# Synthesis of Cyclopropanes via Hydrogen-Borrowing Catalysis

**DOI:** 10.1021/acs.orglett.3c01768

**Published:** 2023-07-10

**Authors:** Jessica L. Crompton, James R. Frost, Sam M. Rowe, Kirsten E. Christensen, Timothy J. Donohoe

**Affiliations:** † Department of Chemistry, University of Oxford, Chemistry Research Laboratory, Mansfield Road, Oxford, OX1 3TA, U.K.; ‡ GSK Medicines Research Centre, Gunnels Wood Road, Stevenage, Hertfordshire SG1 2NY, U.K.

## Abstract

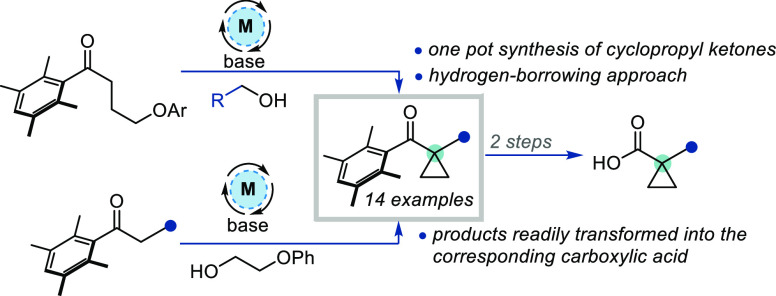

Cyclopropanes are highly useful motifs that are often incorporated into drug candidates to improve potency, metabolic stability, or pharmacokinetic properties. An expedient method for the α-cyclopropanation of ketones using hydrogen borrowing (HB) catalysis is described. The transformation occurs via HB alkylation of a hindered ketone with subsequent intramolecular displacement of a pendant leaving group affording the cyclopropanated product. The leaving group can be installed in either the ketone or alcohol component of the HB system, giving access to α-cyclopropyl ketones via two complementary approaches. Conversion to the corresponding carboxylic acids can be achieved in a simple two-step sequence to afford synthetically useful 1,1-substituted spirocyclopropyl acid building blocks.

Cyclopropanes are often incorporated into drug candidates and FDA-approved drugs (Scheme 1A).^1^ Their incorporation can impose conformational restrictions on the molecule, fixing the positions of the pendant pharmacophores and leading to improved interactions with the target protein. 1,1-Substituted spirocyclopropanes in particular have the potential to introduce a quaternary stereocenter which may enhance 3D shape complementarity with a protein target.^2^ In combination with the intrinsic lipophilicity of the cyclopropane motif, its inclusion can therefore impart a significant boost in potency. Beyond the potential improvement in binding interactions, the introduction of a cyclopropane can enhance both the pharmacokinetic profile and metabolic stability of drug candidates.

**Scheme 1:**
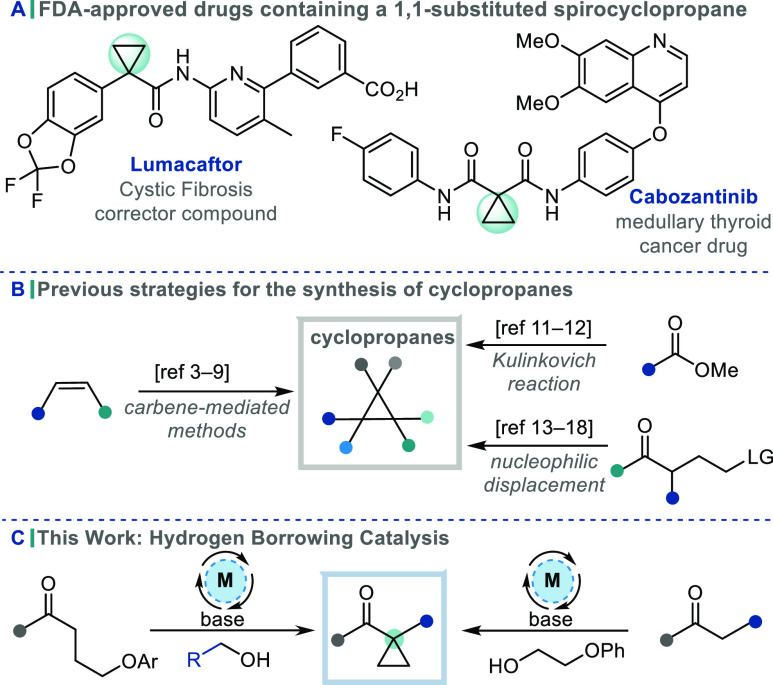
Cyclopropanes in Organic Synthesis

Traditional methods to make cyclopropanes are dominated by carbene-based strategies,^3^ including the Simmons–Smith reaction,^4,5^ the use of free carbenes,^6,7^ and diazo-derived carbenoids (Scheme 1B).^8,9^ The Kulinkovich reaction and cycloisomerization strategies have also been employed for the construction of cyclopropanes in total synthesis.^10−12^ A conceptually distinct classical approach for the synthesis of cyclopropanes is *via* the intramolecular nucleophilic displacement of a leaving group. This was exemplified by Perkin in 1884 and has formed the basis of other powerful transformations, such as the Corey–Chaykovsky reaction.^13−16^ Related approaches have been employed for the synthesis of cyclopropanes in a variety of natural product targets.^17,18^

Although these traditional approaches are extremely powerful methods for cyclopropane formation, they often employ hazardous alkyl halide or pseudohalide-derived reagents, generating copious amounts of toxic waste, or require highly specialized substrates to enable the cyclopropanation to occur.

We have recently reported that *ortho*-disubstituted phenyl ketones can be alkylated *via* the sustainable method of hydrogen borrowing catalysis.^19−22^ The transformation is mediated by a metal catalyst, which removes hydrogen from an alcohol to give the corresponding carbonyl compound and a metal hydride *in situ*. Aldol condensation with the hindered ketone yields the corresponding enone, which is then reduced by the metal-hydride species. Herein, we present the α-cyclopropanation of ketones using a hydrogen borrowing catalysis strategy (Scheme 1C).

Since the α-alkylation of pentamethylphenyl (Ph*) ketones has been well established,^19−21^ we proposed that an α-cyclopropyl group could be formed *via* the displacement of a leaving group from an intermediate α-alkylated ketone. This intermediate could be accessed using HB catalysis by installing a leaving group on either the ketone or alcohol component of the system, offering two complementary approaches to access cyclopropanes using this method.

We began by examining the prefunctionalization of a Ph* ketone with a leaving group (see **1**, **3**, **6a**, Scheme 2). Bromide **1** was shown to be too reactive toward cyclization, giving cyclopropane **2** in high yield before it could be intercepted by a HB process. Sulfur-based leaving groups (see **3**) were better candidates as some conversion to the cyclopropane **4** was observed; however, byproduct **5** was also formed, likely due to the displaced thiolate anion ring-opening the cyclopropane product **4***via* homoconjugate addition.^19^ We surmised that the choice of leaving group was crucial as (i) it must be sufficiently poor to allow for HB alkylation to take place *prior* to intramolecular displacement and (ii) the anion released must not be so nucleophilic as to risk ring opening or other reaction pathways.

**Scheme 2:**
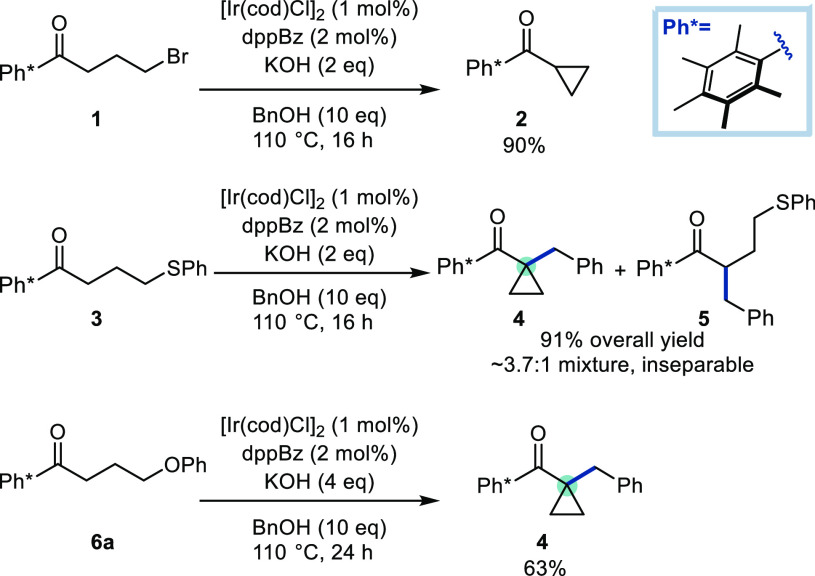
Preliminary Experiments

To this end, we identified ketone **6a**, bearing a pendant phenoxy group, which underwent HB alkylation and subsequent cyclization to give compound **4** in 63% yield (Scheme 2). A selection of electron-rich and -poor substituted phenoxy leaving groups was then screened in an effort to improve the yield (Table 1). The electron-poor substrates **6b**–**6e** gave a reduced yield of cyclopropane **4**, whereas electron-rich *p*-methoxyphenoxy substituted **6f** offered a marginal improvement (67%) over phenoxy substituted **6a** (63%). The reduced yields for the electron-poor substrates were largely due to side reactions (for full details, see Table S1, Supporting Information (SI)). The reaction yield for the *p*-methoxyphenoxy leaving group could be increased further by adding a second portion of base and solvent (Table 1, entry 7; for full optimization table see Table S2, SI). An alternative ruthenium-based catalyst, Ru-MACHO, also gave **4** in a yield identical to that of the iridium-based system for this substrate (Table 1, entry 8).

**Table 1:**
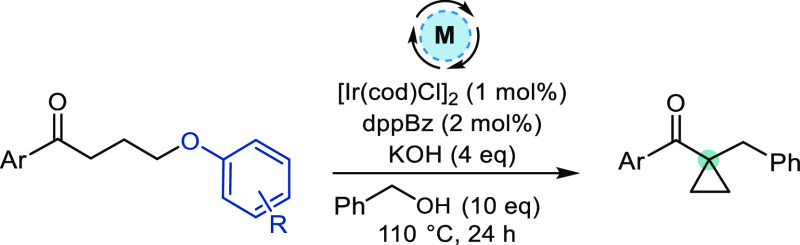

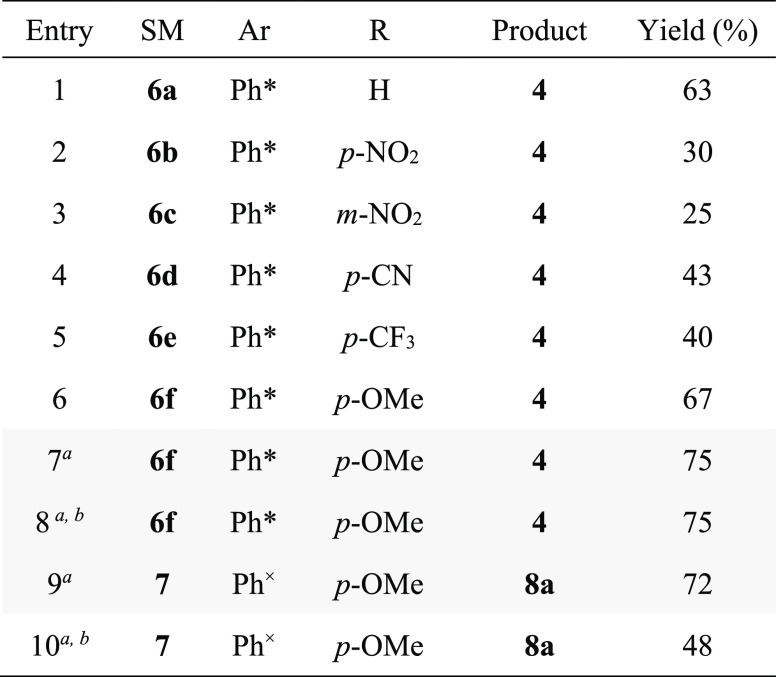
Optimization of the Aryloxy Leaving Group and Ketone Protecting Group

Reactions were performed on 0.30 mmol scale. Isolated yields are shown.

a KOH (2 equiv) and ^*t*^BuOH were added after 24 h and the reaction heated for a further 24 h.

b Ru-MACHO (2 mol %) was used instead of [Ir(cod)Cl]_2_ and dppBz.

Unfortunately, previously developed bromine or acid-mediated conditions for the removal of the Ph* protecting group were unsuccessful due to complete degradation of the newly installed cyclopropane unit.^19,23^ However, the protecting group was easily replaced with the related tetramethylphenyl group (here named as Ph^×^) which could be removed in a mild two-step sequence to give the corresponding carboxylic acid (see Scheme 6).^24^ The Ph^×^ group performed almost identically to the Ph* group under iridium-catalyzed HB conditions (Table 1, entry 9), although a poorer yield was obtained when swapping to the ruthenium-based catalyst (Table 1, entry 10). Note that our attempts to utilize non *ortho*-substituted aryl ketones (e.g., Ph, *p*MeOC_6_H_4_) in cyclopropane forming reactions were unsuccessful and led only to products whereby the ketone had been reduced without cyclopropane formation (see SI). This fits with our earlier work which suggested that the two *ortho*-methyl groups within Ph* effectively shield the carbonyl group from nucleophilic attack, especially reduction.^20^

Having optimized the aryloxy leaving group and ketone protecting group, the scope of the transformation was investigated (Scheme 3). Using **7** as the ketone substrate, a variety of benzylic alcohols were subjected to iridium-catalyzed HB alkylation and subsequent cyclization to give **8a**–**c** in 62–83% yield. Simple alkyl alcohols also proved to be suitable substrates, giving **8d**–**f** in 79–82% yield. Protected heteroatoms were tolerated, forming **8g**–**i** in 42–80% yield. A pyridyl-substituted product **8j** was also isolated in a reasonable yield.

**Scheme 3:**
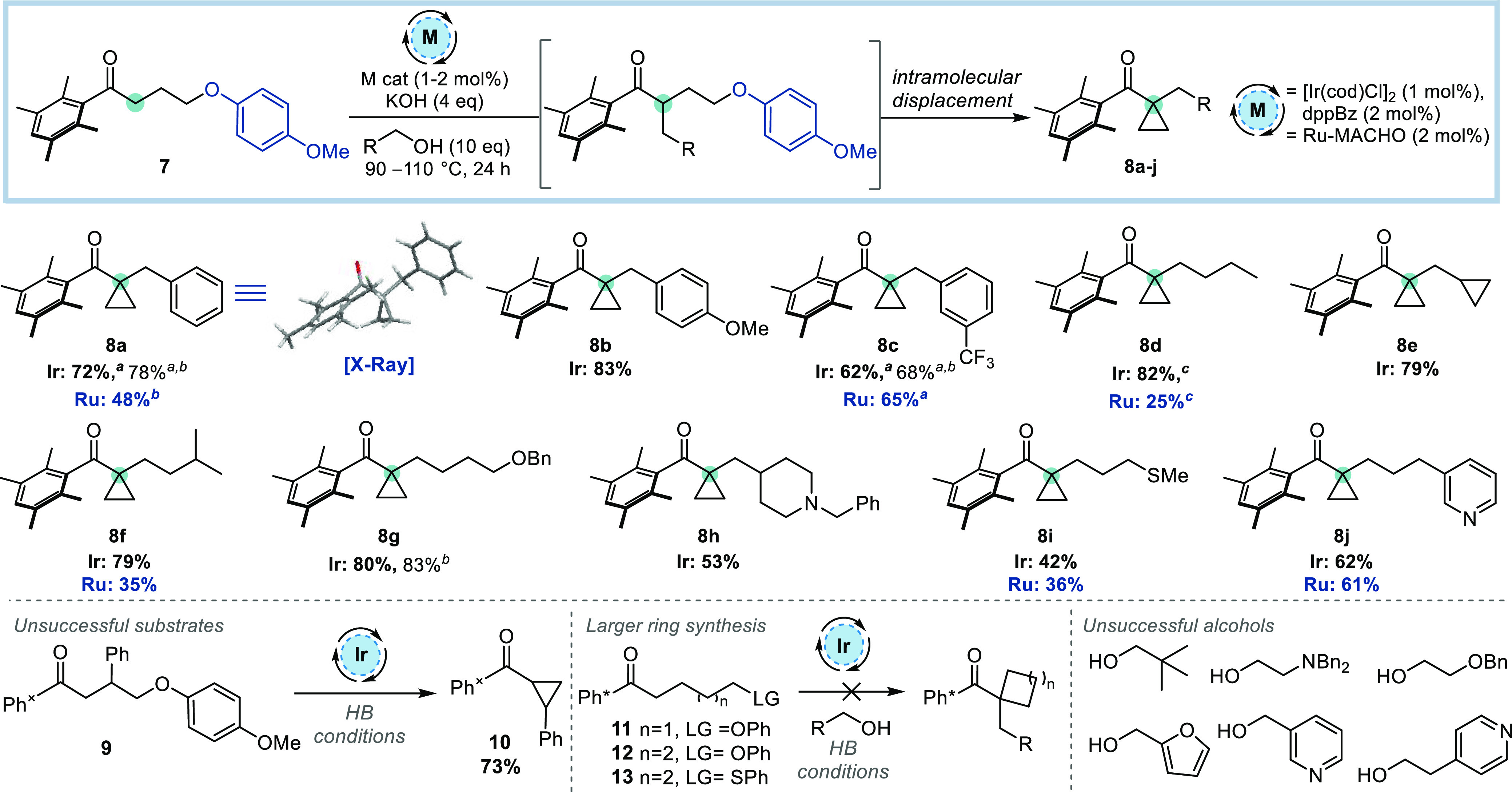
Scope of Cyclopropane Formation *via* HB Alkylation of Ketone Substrate **7** Reactions were performed on a 0.30–0.60 mmol scale unless otherwise stated. Isolated yields are reported. KOH (2 equiv) and ^*t*^BuOH added after 24 h; reaction heated for a further 24 h. Reaction was performed on a 1.00 mmol scale. Reaction performed at 90 °C.

A significant proportion of the alcohols tested did not require a second addition of base and solvent, allowing for a shorter reaction time. The Ru-MACHO catalyst was also employed with several alcohols, and although some products were formed in comparable yields to the Ir-catalyzed system, such as **8c**, **8i**, and **8j**, others were less successful (**8a**, **8d**, **8f**). Unfortunately, attempts to form backbone-substituted cyclopropanes using this method were unsuccessful. When **9** was subjected to the standard HB conditions using cyclopropanemethanol, only cyclopropane **10** was formed. We postulate that adding substituents to the backbone of the prefunctionalized ketone increases the rate of cyclization compared to HB alkylation, preventing the formation of a tertiary center prior to cyclopropanation. Attempts were made to access larger rings using this strategy but these were also unsuccessful; when ketones **11**–**13** were subjected to the reaction conditions, only HB alkylation was observed, even when the leaving group was enhanced to promote subsequent cyclization (see **13**).

Having investigated the formation of cyclopropanes by prefunctionalization of the ketone substrate, attention shifted to the complementary approach, prefunctionalizing the alcohol with a leaving group. The previously optimized conditions were found to be broadly transferrable to this strategy; however, in this scenario the phenoxy leaving group gave the highest yields and optimal conversion was achieved using KO^*t*^Bu for the second addition of base (for full optimization table, see Table S3, SI).

This alcohol prefunctionalization approach was found to be compatible with a variety of Ph^×^ ketones, including an alkyl ketone which gave **8f** in moderate yield (Scheme 4). Adding a phenyl group to the chain was also tolerated to give **8a** and **8k** in 61% and 33% yield, respectively; the decreased yield obtained for **8k** is likely due to increased steric hindrance in the intermediate enolate preventing cyclization on carbon (in this case some cyclization onto the enolate oxygen was observed; see SI). The isolation of compound **8k** highlights the complementarity of the two approaches, as the requirement for a methylene group adjacent to the cyclopropane is removed in this approach, allowing cyclopropane synthesis α- to an aromatic ring. Ketones bearing a pendant heteroatom also proved to be excellent substrates with **8l** and **8m** being formed in 79% and 82% yield respectively. These results further demonstrate the complementarity of the two strategies for the synthesis of cyclopropanes *via* HB catalysis, as compounds analogous to **8l** and **8m** were not successfully formed using the ketone prefunctionalization approach (see unsuccessful alcohols, Scheme 3). Using the alcohol prefunctionalization approach, a ketone bearing a pendant sulfone was also tolerated, giving **8n** in 53% yield. The Ru-MACHO catalyst gave results which were much more comparable to those obtained using the Ir catalyst for this cyclopropanation reaction. Although the yields for this approach are generally slightly lower than their ketone prefunctionalization counterparts (compare **8a** and **8f** across Schemes 3 and 4), the relative ease of starting material synthesis coupled with the commercial availability of the monoprotected diol, which acts as a “cyclopropane surrogate”, renders this approach more synthetically useful.

**Scheme 4:**
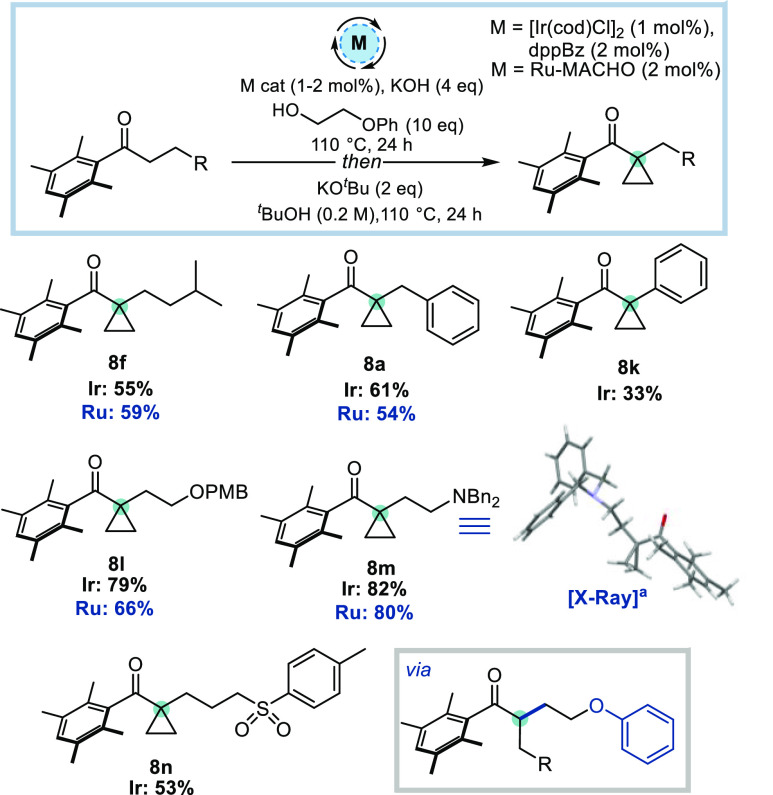
Scope of Alcohol Prefunctionalization Approach Reactions were performed on a 0.30 mmol scale. Isolated yields are reported. Disorder is omitted for clarity.

To support our mechanistic proposal for these cyclopropanation reactions, the cyclization of compound **14** was investigated (Scheme 5A). This ketone should represent an intermediate in the proposed reaction pathway, preceding leaving group displacement. When treated with base in ^*t*^BuOH, **14** cyclized cleanly to give cyclopropane **4** in high yield, which is consistent with cyclization being the final step of the reaction mechanism. This supports the proposed two-step reaction pathway, namely, ketone alkylation by HB catalysis to afford an α-branched intermediate bearing a pendant leaving group which subsequently undergoes intramolecular nucleophilic displacement by the enolate (Scheme 5B).

**Scheme 5:**
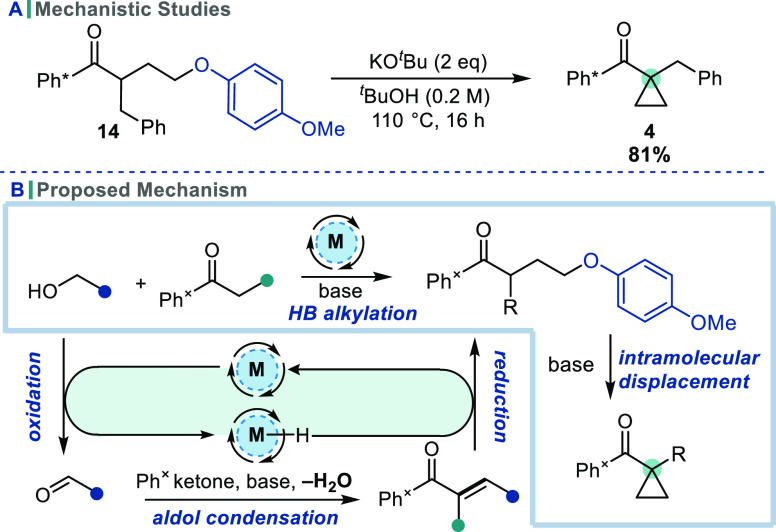
Mechanistic Studies and Mechanistic Proposal

We have previously shown that Ph^×^ ketones can be converted into the corresponding carboxylic acids using a two-step sequence.^24^ Thus, oxidation of the product cyclopropyl ketones with phthaloyl peroxide in HFIP gave the corresponding phenols **15a**–**e** in moderate to good yields (Scheme 6).^25^ These phenols were subsequently treated with CAN to afford carboxylic acids **16a**–**e** in 61–86% yield, leaving the cyclopropyl alcohol ring intact. Facile access to this acid functional handle greatly enhances the synthetic utility of this methodology, enabling installation of cyclopropane groups without the need to resort to classical alkylation conditions using toxic alkyl halides.

**Scheme 6:**
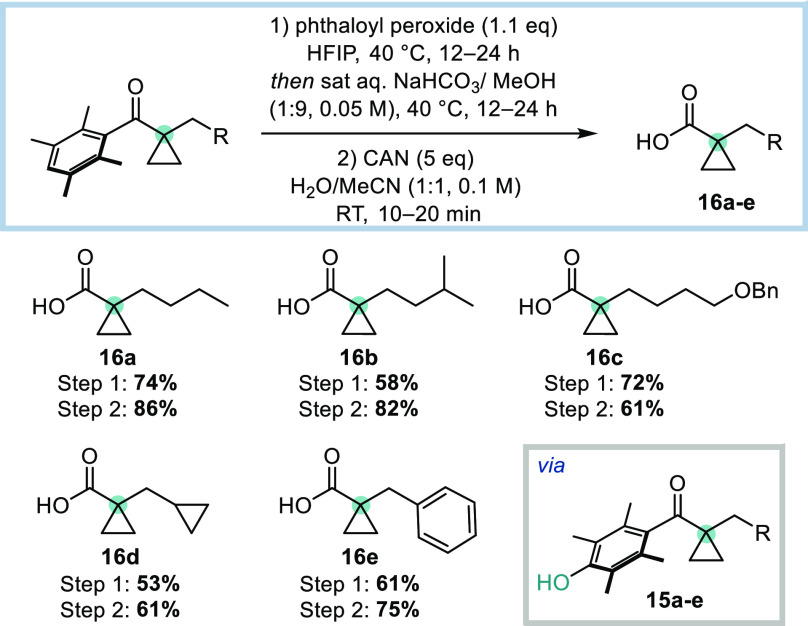
Derivatization of α-Cyclopropyl Ketones Reactions were performed on a 0.20–0.30 mmol scale. Isolated yields are reported.

In summary, we present a new method for the formation of α-cyclopropyl ketones via hydrogen borrowing catalysis and subsequent intramolecular displacement. We have demonstrated the feasibility of two complementary strategies, whereby the leaving group is installed on either the ketone or alcohol substrate, respectively. Furthermore, we have shown that the Ph^×^ ketone protecting group can be easily removed in a mild two-step sequence to allow access to synthetically useful α-cyclopropyl carboxylic acids which can be employed in wider synthetic applications.

## Data Availability

The data underlying this study are available in the published article and its Supporting Information.

## Supporting Information Available

Experimental Procedures and characterization for all new compounds; expanded optimization tables (PDF)
