# Supplementary material for: Synthesis of Cyclopropanes via Hydrogen-Borrowing Catalysis
Source: Org Lett. 2023 Jul 10;25(28):5253–7. doi: 10.1021/acs.orglett.3c01768 (PMC10367074; doi:10.1021/acs.orglett.3c01768)
Supplement: Supplementary file 1 — ol3c01768_si_001.pdf [file ol3c01768_si_001.pdf]

SYNTHESIS OF CYCLOPROPANES VIA HYDROGEN-BORROWING CATALYSIS: SUPPLEMENTARY INFORMATION

Jessica L. Crompton,[a] James R. Frost,[a] Sam M. Rowe,[b] Kirsten E. Christensen[a] and Timothy J. Donohoe [a]*

[a] Department of Chemistry, University of Oxford, Chemistry Research Laboratory, Mansfield Road, Oxford, OX1 3TA, UK. E-mail: timothy.donohoe@chem.ox.ac.uk

[b] GSK Medicines Research Centre, Gunnels Wood Road, Stevenage, Hertfordshire, SG1 2NY, UK.

GENERAL INFORMATION 2

GENERAL PROCEDURES 4

OPTIMISATION TABLES 6

PREPARATION AND CHARACTERISATION OF COMPOUNDS 10

Pre-functionalisation Method 10

Cyclopropane Surrogate Method 39

Cyclopropane Surrogate Optimisation and Mechanistic Studies 51

Derivatisation 53

NMR SPECTRA OF NOVEL COMPOUNDS 61

X-RAY CRYSTALLOGRAPHY SUPPLEMENTARY INFORMATION 120

REFERENCES 122

General Information

Microwave vials and vial caps (containing a resealing Silicone/PTFE septum) were purchased from Kinesis (Cole-Palmer) and were used without flame-drying. All other reactions were carried out in flame-dried glassware under an atmosphere of nitrogen unless stated otherwise. Room temperature (RT) refers to 20–25 °C. Temperatures of 0 °C were obtained using an ice/water bath. Heating was achieved using an oil bath equipped with a contact thermometer.

PhMe, CH_2Cl_2, Et_2O, tetrahydrofuran, MeCN and 1,4-dioxane were purified by filtration through activated alumina columns employing the method of Grubbs et al.[1] Water was purified by an Elix® UV-10 system. All other solvents and reagents were used as supplied without prior purification. All other reagents were used directly as supplied by major chemical suppliers.

Thin layer chromatography was performed on Merck Kieselgel 60 F_{254} 0.25 mm pre-coated aluminium plates. Product spots were visualized under UV light (λ = 254 nm) and/or by staining with potassium permanganate solution, phosphomolybdic acid stain or vanillin in acidic ethanol (Vanillin stain). Flash chromatography was performed using VWR silica gel 60 (40–63 µm particle size) or (15–40 µm particle size) using head pressure by means of a nitrogen line.

NMR spectroscopy was carried out using Bruker 400 MHz, 500 MHz or 600 MHz (with helium-cooled broadband cryoprobe) spectrometers in the deuterated solvent stated, using the residual non-deuterated solvent signal as an internal reference. Chemical shifts are quoted in ppm with signal splittings recorded as singlet (s), doublet (d), triplet (t), quartet (q), pentet (p), multiplet (m). Higher multiplicities are indicated by combinations of these abbreviations, for example: doublet of doublets (dd). The abbreviation br denotes broad. Coupling constants, J, are rounded to the nearest 0.1 Hz and are presented as observed. Unless otherwise stated, all NMR experiments were performed at 298 K. Systematic names were generated by the computer program ChemDraw according to the guidelines specified by the International Union of Pure and S4 Applied Chemistry (IUPAC).

Melting points were determined using a Griffin melting point apparatus and are uncorrected. Infrared spectra were recorded neat or as a thin film on a Bruker Tensor 27 spectrometer equipped with an attenuated total reflectance attachment with internal calibration. Absorption maxima (λ_{max}) are quoted in wavenumbers (cm^{-1}). The abbreviation br denotes broad.

Low-resolution mass spectra were recorded on a Waters LCT Premier XE bench-top orthogonal acceleration time-of-flight LC-MS system (loop injection mode). High Resolution Mass spectrometry was carried out using flow injection analysis and was performed on an ACQUITY I-Class PLUS UPLC System (Waters, Milford, MA, USA) coupled to an ACQUITY RDa mass spectrometer (Waters, Milford, MA, USA) equipped with an ESI probe and a TOF mass analyzer. In positive ion mode, the flow rate was set to 0.300 mL/min using a 50% methanol(aq) + 0.1% formic acid eluent. Scan parameters were set as follows: analyser mode, full scan; scan range, 50 2000 m/z; scan rate, 2 Hz; cone voltage, 30 V; capillary voltage, 1.5 kV; desolvation temperature, 550 °C; and intelligent data capture, on. In negative ion mode, the flow rate was set to 0.300 mL/min using a 50% methanol(aq) + 0.1% formic acid eluent. Scan parameters were set as follows: analyser mode, full scan; scan range, 50 2000 m/z; scan rate, 2 Hz; cone voltage, 40 V; capillary voltage, 0.8 kV; desolvation temperature, 550 °C; and intelligent data capture, on. Electrospray ionisation (ESI) HRMS were also recorded on a Thermo Exactive orbitrap spectrometer equipped with a Waters Equity LC system, with a flow rate of 0.2 mL/min using water:methanol:formic acid (10:89.9:0.1) as eluent. The system uses an orbitrap mass analyzer and a heated electrospray ionisation (HESI-II) probe for ESI^+ and has a resolution of 50,000 FWHM under conditions for maximum sensitivity, with an accuracy of better than 5 ppm for 24 h following external calibration on the day of analysis. The mass reported is that containing the most abundant isotopes, with each value rounded to 4 decimal places and within 5 ppm of the calculated mass.

Single crystal X-ray diffraction data were collected using a Rigaku Synergy-DW diffractometer (EP/V028995/1) and CrysAlisPro. In all cases, Cu-Kα (λ = 1.54184 Å) radiation was used and the instrument was equipped with a nitrogen gas Oxford Cryosystems Cryostream unit. Data were collected at 100 K. Structures were solved using 'Superflip'[2] before refinement with CRYSTALS[3, 4] as per the SI (CIF).

General Procedures

General Procedure 1: Hydrogen borrowing reaction using pre-functionalised ketones to give α-cyclopropyl ketones

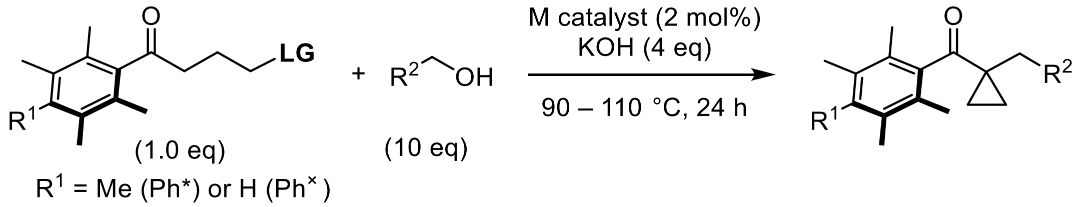

Ph* or Ph$^{\times}$ Ketone (0.30 mmol, 1.0 eq), metal catalyst (2.0 mol% M) and KOH (67 mg, 1.2 mmol, 4.0 eq) were added to a 2-5 mL microwave vial. The vial was sealed, evacuated and backfilled with Ar (three times) and alcohol (3.0 mmol, 10 eq, degassed with Ar (balloon)) was added. The reaction was heated at 90 – 110 °C for 24 h, cooled to RT, filtered through a silica plug (Et_2O elution) and concentrated *in vacuo*. Purification was accomplished *via* flash column chromatography as indicated.

General Procedure 2: Mitsunobu Reaction for the synthesis of 6a-f

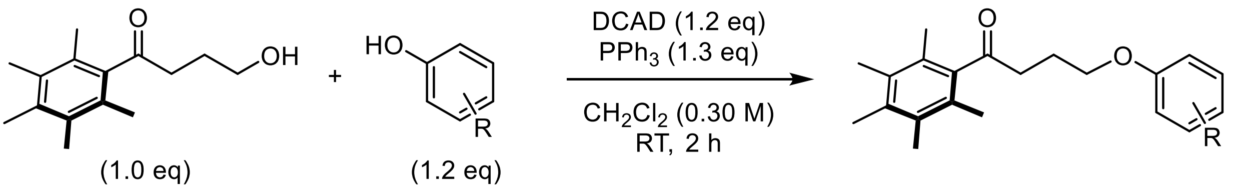

A substituted phenol (1.2 eq) was added to a solution of **S2** (1.0 eq)) in CH_2Cl_2 (0.30 M). PPh_3 (1.3 eq) and di-(4-chlorobenzyl)azodicarboxylate (DCAD, 1.2 eq) were added and the reaction was stirred at RT for 2 h. The reaction mixture was filtered and the filtrate was concentrated *in vacuo*. Purification was accomplished *via* flash column chromatography as indicated**.**

General Procedure 3: Hydrogen borrowing reaction using 2-phenoxyethanol as a cyclopropane surrogate to give α-cyclopropyl ketones

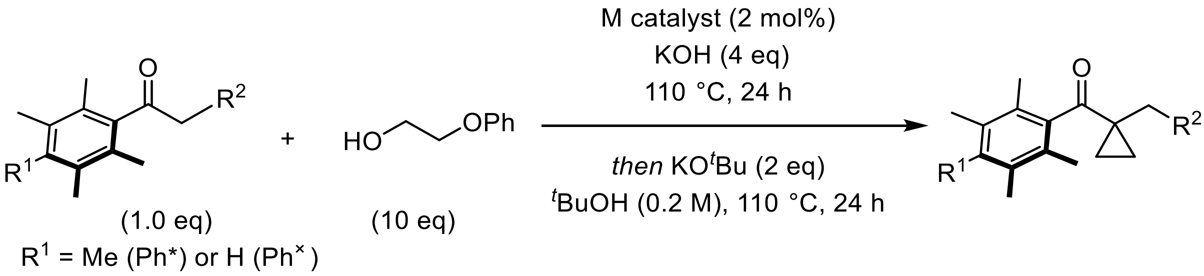

Ph* or Ph$^{\times}$ Ketone (0.30 mmol, 1.0 eq), metal catalyst (2.0 mol% M), KOH (67 mg, 1.2 mmol, 4.0 eq) were added to a 2-5 mL microwave vial. The vial was sealed, evacuated and backfilled with Ar (three times) and 2-phenoxyethanol (0.37 mL, 3.0 mmol, 10 eq, degassed with Ar (balloon)) was added. The

reaction was heated at 110 °C for 24 h, cooled to RT and KO^{t}Bu (68 mg, 0.60 mmol, 2.0 eq) was added. The vial was re-sealed and purged with Ar (balloon for 5 min). ^{t}BuOH (1.5 mL, 0.2 M) was added and the reaction was heated at 110 °C for a further 24 h, cooled to RT, filtered through a silica plug (Et_2O elution) and concentrated *in vacuo*. Purification was accomplished *via* flash column chromatography as indicated.

General Procedure 4: Oxidation of Ph$^{\times}$ group using phthaloyl peroxide

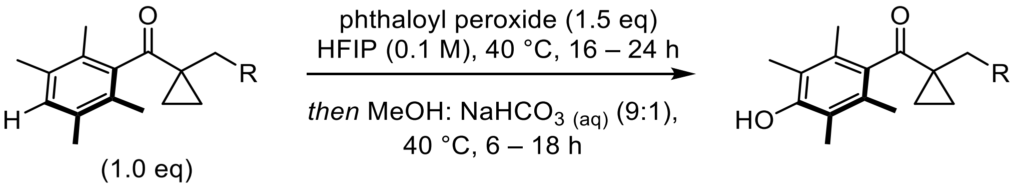

Phthaloyl peroxide **S26** (1.5 eq) was added to a solution of Ph$^{\times}$ ketone (1.0 eq) in 1,1,1,3,3,3-hexafluoro-2-propanol (0.1 M, degassed with Ar (balloon)). The resulting mixture was purged with Ar (balloon) and heated at 40 °C for 16–24 h. The crude reaction mixture was concentrated *in vacuo* and deoxygenated MeOH and sat. $NaHCO_3$ (9:1, 0.1 M, degassed with Ar) were added. The mixture was heated at 40 °C for a further 6–18 h, cooled to RT and diluted with brine and CH_2Cl_2. The layers were separated and the aqueous layer extracted twice with CH_2Cl_2. The combined organic layers were dried over Na_2SO_4 an concentrated in vacuo. Purification was accomplished *via* flash column chromatography as indicated.

Optimisation Tables

Table S1: Leaving Group Screening for the Synthesis of Ph* Cyclopropanes

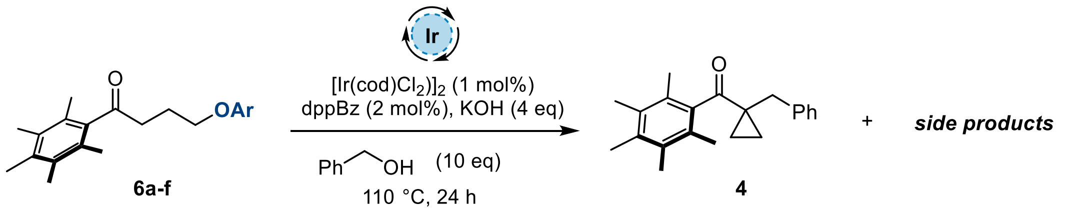

Entry	Compound	Leaving Group (OAr)	Yield 4 (%)	Side Product(s) & Yield(s) (%)
1	6a	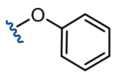	63	N/A
2	6b	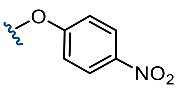	30	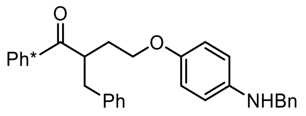 4% (NMR)
3	6c	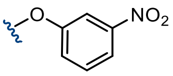	25	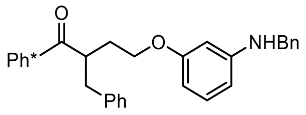 21%
4	6d	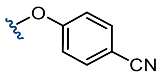	43	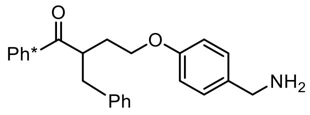 6%
5	6e	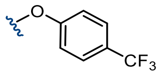	40	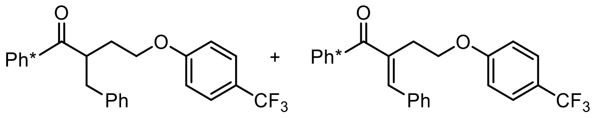 27% (NMR) + 11% (NMR)
6	6f	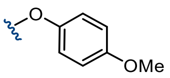	67	N/A
7[a]	6f	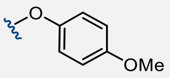	75	N/A
8[ab]	6f	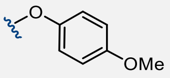	75	N/A

Reactions carried out on a 0.3 mmol scale. NMR yields determined via ^{1}H quantitative NMR using 1,1,2,2-tetrachloroethane as an internal standard. Yields are isolated unless otherwise indicated.

[a] 2 eq. KOH and ^{t}BuOH (0.2 M) added after 24 h, reaction heated for a further 24 h.

[b] Ru-MACHO® (2.0 mol%) was used instead of $[Ir(cod)Cl]_2$ (1.0 mol%) and dppBz (2.0 mol%)

Table S2: Further optimisation of Pre-functionalised Approach using 6f

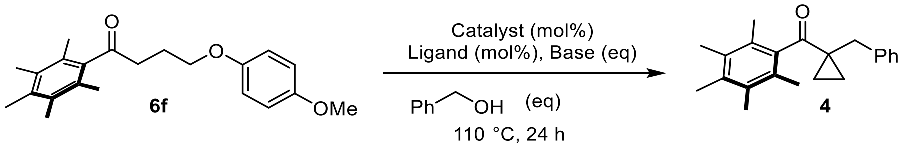

Entry	Catalyst (mol%)	Ligand (mol%)	Benzyl Alcohol Eq	Base (Eq)	Solvent (4 M)	Additive (Eq)	Yield 4 (%)[a]
**1**	$[Ir(cod)Cl]_2$ (1)	dppBz (2)	10	KOH (4)	N/A	N/A	68 *(63)*
**2**	$[Ir(cod)Cl]_2$ (1)	dppBz (2)	10	KOH (2)	N/A	N/A	24
**3**	$[Ir(cod)Cl]_2$ (1)	dppBz (2)	10	KO^{t}Bu (4)	N/A	N/A	58
**4**	$[Ir(cod)Cl]_2$ (1)	dppBz (2)	10	KO^{t}Bu (2)	N/A	N/A	13
**5**	$[Ir(cod)Cl]_2$ (1)	dppBz (2)	10	NaOH (4)	N/A	N/A	51
**6**	$[Ir(cod)Cl]_2$ (1)	dppBz (2)	10	KO^{t}Bu (4)	N/A	N/A	26
**7**	$[Ir(cod)Cl]_2$ (1)	dppBz (2)	10	KO^{t}Bu (4)	PhMe	N/A	43
**8**	$[Ir(cod)Cl]_2$ (1)	dppBz (2)	10	KO^{t}Bu (4)	^{t}BuOH	N/A	26
**9**	$[Ir(cod)Cl]_2$ (1)	dppBz (2)	5	KO^{t}Bu (4)	^{t}BuOH	N/A	53
**10**	$[Ir(cod)Cl]_2$ (1)	dppBz (2)	2	KO^{t}Bu (4)	^{t}BuOH	N/A	50
**11**	$[IrCp^*Cl_2]_2$ (1)	N/A	10	KOH (4)	N/A	N/A	65
**12**	$[RhCp^*Cl_2]_2$ (1)	N/A	10	KOH (4)	N/A	N/A	55
**13**	$[RuCp^*Cl_2]_n$ (2)	N/A	10	KOH (4)	N/A	N/A	43
**14**	Ru-MACHO (2)	N/A	10	KOH (4)	N/A	N/A	72 *(64)*
**15**	$Ni(OAc)_2$ (2)	P^{t}Bu$_3$ (4)	10	KOH (4)	N/A	N/A	39
**16**[b]	Fe Knöllker **Cat1** (2)	P^{t}Bu$_3$ (4)	10	KOH (4)	N/A	N/A	23
**17**	$[Ir(cod)Cl]_2$ (1)	dppBz (2)	10	KOH (4)	N/A	KOH (2), ^{t}BuOH (0.2 M)	75 *(75)*
**18**	$[Ir(cod)Cl]_2$ (1)	dppBz (2)	10	KOH (4)	N/A	KO^{t}Bu (2), ^{t}BuOH (0.2 M)	62
**19**	$[Ir(cod)Cl]_2$ (1)	dppBz (2)	10	KOH (4)	N/A	KOH (4), ^{t}BuOH (0.2 M)	52
**20**	Ru-MACHO (2)	N/A	10	KOH (4)	N/A	KOH (2), ^{t}BuOH (0.2 M)	73 *(75)*
**21**	$[IrCp^*Cl_2]_2$ (1)	N/A	10	KOH (4)	N/A	KOH (4), ^{t}BuOH (0.2 M)	74
**22**	$[Ir(cod)Cl]_2$ (1)	dppBz (2)	10	KOH (4)	N/A	KOH (4), PhMe (0.2 M)	52

[a] Yields determined by ^{1}H quantitative NMR using 1,1,2,2-tetrachloroethane as an internal standard, isolated yield given in parentheses
[b] Me_3NO (4 mol%) was added at the start of the reaction

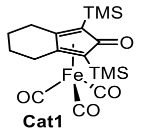

Cat1

Table S3: One-Pot reaction Screening for Cyclopropane Synthesis using mono-activated diol

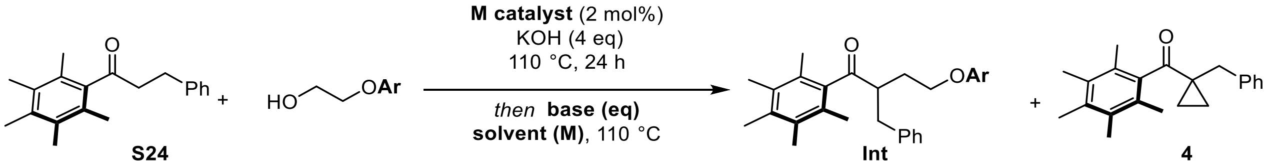

Entry	Catalyst (Loading)	Ar	Alcohol Eq	Base added after 24 h (Eq)	Solvent added (M)	Rxn time after base addition, t (h)	Yield Int (%)[a]	Yield 4 (%)[a]
**1**	$[Ir(cod)Cl]_2$ (1) dppBz (2)	Ph	10	-	-	0 (24 h rxn)	39	33
**2**	$[Ir(cod)Cl]_2$ (1) dppBz (2)	Ph	10	-	-	24 (48 h rxn)	15	20
**3**	$[Ir(cod)Cl]_2$ (1) dppBz (2)	Ph	10	-	^{t}BuOH (0.2)	24	7	22
**4**	$[Ir(cod)Cl]_2$ (1) dppBz (2)	Ph	10	KO^{t}Bu (4)	^{t}BuOH (0.2)	24	52	26
**5**	$[Ir(cod)Cl]_2$ (1) dppBz (2)	Ph	10	KO^{t}Bu (2)	^{t}BuOH (0.2)	24	24	48*(60)*
**6**	$[Ir(cod)Cl]_2$ (1) dppBz (2)	Ph	10	KO^{t}Bu (1)	^{t}BuOH (0.2)	24	25	36 *(50)*
**7**[b]	$[Ir(cod)Cl]_2$ (1) dppBz (2)	Ph	10	KO^{t}Bu (2)	^{t}BuOH (0.2)	24	55	20
**8**	$[Ir(cod)Cl]_2$ (1) dppBz (2)	Ph	10	KO^{t}Bu (2)	^{t}BuOH (0.5)	24	27	44 *(46)*
**9**	$[Ir(cod)Cl]_2$ (1) dppBz (2)	Ph	10	KO^{t}Bu (2)	^{t}BuOH (0.1)	24	24	38 *(41)*
**10**	$[Ir(cod)Cl]_2$ (1) dppBz (2)	Ph	10	KO^{t}Bu (2)	^{t}BuOH (0.2)	6	15	23
**11**[c]	$[Ir(cod)Cl]_2$ (1) dppBz (2)	Ph	10	-	^{t}BuOH (0.2)	24	12	26
**12**	$[Ir(cod)Cl]_2$ (1) dppBz (2)	Ph	5	KO^{t}Bu (2)	^{t}BuOH (0.2)	24	48	31
**13**	$[Ir(cod)Cl]_2$ (1) dppBz (2)	Ph	2	KO^{t}Bu (2)	^{t}BuOH (0.2)	24	72	18
**14**	$[Ir(cod)Cl]_2$ (1) dppBz (2)	Ph	10	KOH (2)	^{t}BuOH (0.2)	24	38	26

**15**	$[Ir(cod)Cl]_2$ (1) dppBz (2)	Ph	10	KO^{t}Bu (2)	PhMe (0.2)	24	37	43
**16**	$[Ir(cod)Cl]_2$ (1) dppBz (2)	PMP	10	KO^{t}Bu (2)	^{t}BuOH (0.2)	24	36 *(33)*	59 *(40)*
**17**	$[Ir(cod)Cl]_2$ (1) dppBz (2)	PMP	5	KO^{t}Bu (2)	^{t}BuOH (0.2)	24	50	34
**18**	$[Ir(cod)Cl]_2$ (1) dppBz (2)	PMP	2	KO^{t}Bu (2)	^{t}BuOH (0.2)	24	51	19
**19**[d]	$[Ir(cod)Cl]_2$ (1) dppBz (2)	PMP	10	KO^{t}Bu (2)	^{t}BuOH (0.2)	24	*(31)*	*(31)*
**20**	Ru-MACHO (2)	Ph	10	KO^{t}Bu (2)	^{t}BuOH (0.2)	24	-	*(62)*

[a] NMR yields deteremined by quantitative ^{1}H NMR using 1,1,2,2-tetrachloroethane as an internal standard. Isolated yields are given in parentheses.

[b] KO^{t}Bu (4 eq.) added in place of KOH in the reaction

[c] KO^{t}Bu (2 eq.) added at the start of the reaction

[d] PhMe (4 M) added at the start of the reaction.

Preparation and Characterisation of Compounds

Pre-functionalisation Method

1 4-Bromo-1-(2,3,4,5,6-pentamethylphenyl)butan-1-one

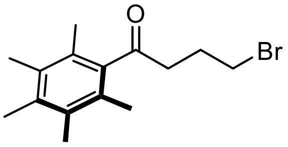

A solution of pentamethylbenzene (4.45 g, 30.0 mmol) and 4-bromobutyryl chloride (3.8 mL, 33 mmol) in CH_2Cl_2 (150 mL) was cooled in an ice bath. $AlCl_3$ (5.00 g, 37.5 mmol) was added portion-wise over 5 minutes, the resulting mixture was allowed to warm to RT and stirred for 2 h. The crude reaction mixture was poured onto ice (approx. 150 g) and the layers were separated. The aqueous layer was extracted with CH_2Cl_2 (3 × 100 mL) and the combined organic layers were washed with satd. aq. $NaHCO_3$ and brine, dried over Na_2SO_4 and concentrated *in vacuo*. Purification *via* flash column chromatography (eluent Pentane/Et_2O, 98:2 → 95:5) gave the *title compound* as a white solid (8.26 g, 93%).

^{1}H NMR (400 MHz, $CDCl_3$) δ 3.57 (t, *J* = 6.4 Hz, 2H), 2.88 (t, *J* = 6.9 Hz, 2H), 2.29 (p, *J* = 6.7 Hz, 2H), 2.24 (s, 3H), 2.19 (s, 6H), 2.11 (s, 6H);

^{13}C NMR (101 MHz, $CDCl_3$) δ 210.8, 140.4, 135.7, 133.3, 127.4, 43.5, 33.5, 26.4, 17.3, 16.8, 16.1.

m/z (ESI$^+$) Found $[M(^{81}Br)+H]^+$ = 299 (48%), $C_{15}H_{22}{}^{81}BrO$ requires 299, $[M(^{79}Br)+H]^+$ = 297 (52%), $C_{15}H_{22}{}^{79}BrO$ requires 297.

The spectroscopic data matched that previously reported in the literature.[5]

2 Cyclopropyl(2,3,4,5,6-pentamethylphenyl)methanone

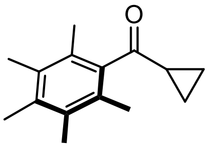

Ketone **1** (89 mg, 0.30 mmol), $[Ir(cod)Cl]_2$ (2.0 mg, 1.0 mol%), dppBz (3.0 mg, 2.0 mol%), KOH (67 mg, 1.2 mmol) and benzyl alcohol (0.31 mL, 3.0 mmol, degassed with Ar (balloon)) were subjected to **General Procedure 1** at 110 °C for 16 h. Purification *via* flash column chromatography (eluent Pentane/Et_2O, 98.5:1.5 → 98:2) gave the *title compound* as a white solid (58 mg, 90%);

m.p. = 68 – 70 °C;

^{1}H NMR (400 MHz, $CDCl_3$) δ 2.24 (s, 3H), 2.20 (s, 6H), 2.17 (s, 7H), 1.33 – 1.18 (m, 2H), 1.13 – 1.00 (m, 2H);

^{13}C NMR (101 MHz, $CDCl_3$) δ 212.1, 141.1, 135.5, 133.1, 127.9, 23.8, 17.6, 16.8, 16.1, 12.3;

IR (film) ν_{max}/cm^{-1} 3007, 2925, 1682, 1365, 1120, 1064, 1030, 993, 937;

m/z (ESI$^+$) Found $[M+H]^+$ = 217, $C_{15}H_{21}O$ requires 217;

HRMS (ESI$^+$) m/z: $[M+H]^+$ Calcd for $C_{15}H_{21}O^+$ 217.1587; found 217.1587, Δ 0.14 ppm.

3 1-(2,3,4,5,6-Pentamethylphenyl)-4-(phenylthio)butan-1-one

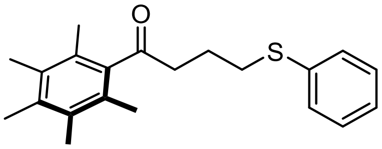

Triethylamine (0.94 mL, 6.7 mmol) was added dropwise to a solution of 4-bromo-1-(2,3,4,5,6-pentamethylphenyl)butan-1-one **1** (1.00 g, 3.36 mmol) and thiophenol (0.41 mL, 4.0 mmol) in EtOAc (35 mL) at RT. The mixture was heated to 65 °C and stirred for 16 h. The mixture was cooled to RT and concentrated *in vacuo*. Purification *via* flash column chromatography (eluent Pentane/Et_2O, 100:0 → 95:5) gave the *title compound* as a white solid (893 mg, 81%).

m.p. = 67 – 69 °C;

^{1}H NMR (400 MHz, $CDCl_3$) δ 7.37 – 7.33 (m, 2H), 7.32 – 7.26 (m, 2H), 7.22 – 7.15 (m, 1H), 3.05 (t, *J* = 7.0 Hz, 2H), 2.85 (t, *J* = 7.1 Hz, 2H), 2.23 (s, 3H), 2.18 (s, 6H), 2.12 – 2.00 (m, 8H);

^{13}C NMR (101 MHz, $CDCl_3$) δ 211.3, 140.6, 136.2, 135.6, 133.2, 129.7, 129.1, 127.4, 126.2, 43.9, 33.3, 22.7, 17.3, 16.8, 16.1;

IR (film) ν_{max}/cm^{-1} 2916, 1699, 1481, 1399, 1112, 739, 692;

HRMS (ESI$^+$) m/z: $[M+Na]^+$ Calcd for $C_{21}H_{26}OSNa$ 349.1600; found 349.1598, Δ 0.38 ppm.

4 (1-Benzylcyclopropyl)(2,3,4,5,6-pentamethylphenyl)methanone

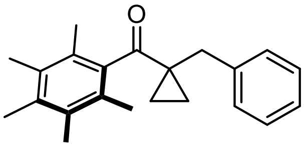

Ir-catalysed: Ketone **6f** (102 mg, 0.300 mmol), $[Ir(cod)Cl]_2$ (2.0 mg, 1.0 mol%), dppBz (3.0 mg, 2.0 mol%), KOH (67 mg, 1.2 mmol) and benzyl alcohol (0.31 mL, 3.0 mmol, degassed with Ar) were added to a 2-5 mL microwave vial. The vial was sealed and evacuated and back-filled with Argon (three times). The reaction was heated at 110 °C for 24 h, cooled to RT and KOH (34 mg, 0.60 mmol) and ^{t}BuOH (1.5 mL) were added. The reaction was heated at 110 °C for a further 24 h, cooled to RT, filtered through a silica plug (Et_2O elution) and concentrated *in vacuo*. Purification *via* flash column chromatography (eluent Pentane/Et_2O, 98:2 → 95:5) gave the *title compound* as a colourless oil (69 mg, 75%).

Ru-catalysed: Ketone **6f** (102 mg, 0.300 mmol), Ru-MACHO® (3.6 mg, 2.0 mol%), KOH (67 mg, 1.2 mmol) and benzyl alcohol (0.31 mL, 3.0 mmol, degassed with Ar) were added to a 2-5 mL microwave vial. The vial was sealed and evacuated and back-filled with Argon (three times). The reaction was heated at 110 °C for 24 h, cooled to RT and KOH (34 mg, 0.60 mmol) and ^{t}BuOH (1.5 mL) were added. The reaction was heated at 110 °C for a further 24 h, cooled to RT, filtered through a silica plug (Et_2O elution) and concentrated *in vacuo*. Purification *via* flash column chromatography (eluent Pentane/Et_2O, 98:2 → 95:5) gave the *title compound* as a colourless oil (69 mg, 75%).

m.p. = 69 – 71 °C;

^{1}H NMR (400 MHz, $CDCl_3$) δ 7.25 – 7.17 (m, 3H), 7.13 – 7.08 (m, 2H), 3.06 (s, 2H), 2.21 (s, 3H), 2.13 (s, 6H), 1.97 (s, 6H), 1.24 – 1.15 (m, 2H), 1.05 – 0.92 (m, 2H).

^{13}C NMR (101 MHz, $CDCl_3$) δ 212.6, 139.1, 137.3, 135.3, 132.9, 130.1, 128.6, 128.0, 126.3, 37.8, 34.4, 17.6, 17.1, 16.8, 16.0;

IR (film) ν_{max}/cm^{-1} 3027, 2921, 1671, 917, 749, 701;

m/z (ESI^{+}) Found $[M+H]^{+}$ = 307, $C_{22}H_{27}O$ requires 307;

HRMS (ESI^{+}) m/z: $[M+H]^{+}$ Calcd for $C_{22}H_{27}O^{+}$ requires 307.2056; found 307.2057, Δ 0.33 ppm.

5 2-Benzyl-1-(2,3,4,5,6-pentamethylphenyl)-4-(phenylthio)butan-1-one (mixture with 4)

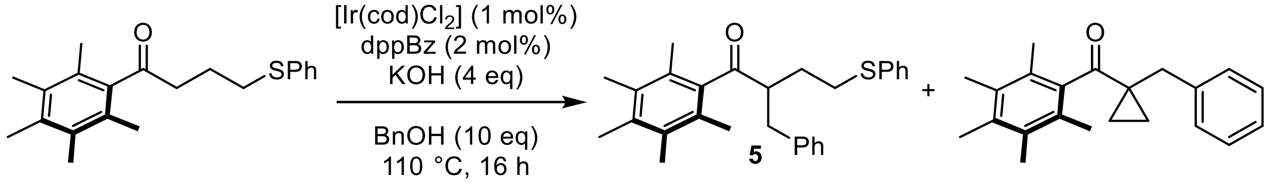

Ketone **3** (89 mg, 0.300 mmol), $[Ir(cod)Cl]_2$ (2.0 mg, 1.0 mol%), dppBz (3.0 mg, 2.0 mol%), KOH (67 mg, 1.2 mmol) and benzyl alcohol (0.31 mL, 3.0 mmol, degassed with Ar) were subjected to **General Procedure 1** at 110 °C for 16 h. Purification *via* flash column chromatography (eluent Pentane/Et_2O, 98:2) gave the *title compound* as a colourless oil (as an inseparable mixture with **4,** ratio 3.7:1, **5:4**)) (107 mg, 91% overall yield);

Selected Data for 5 (from the mixture):

^{1}H NMR (400 MHz, $CDCl_3$) δ 7.17 – 6.88 (m, 10H), 3.24 (dq, *J* = 8.6, 5.8 Hz, 1H), 3.12 (dd, *J* = 13.7, 5.5 Hz, 1H), 2.87 – 2.68 (m, 2H), 2.53 (dd, *J* = 13.7, 8.7 Hz, 1H), 2.16 (s, 3H), 2.13 (d, *J* = 2.2 Hz, 1H), 2.08 (s, 6H), 1.98 – 1.83 (m, 7H, (br s, 6 H and m, 1H, overlapped), 1.61 (ddt, *J* = 14.1, 8.3, 5.9 Hz, 1H);

^{13}C NMR (101 MHz, $CDCl_3$) δ 212.5, 139.7, 139.2, 135.9, 135.8, 133.3, 129.8, 129.4, 128.9, 128.6, 126.4, 126.1, 54.5, 35.9, 32.1, 28.8, 18.0, 16.9, 16.2;

HRMS (ESI^+) m/z: $[M+Na]^+$ Calcd for $C_{28}H_{32}NaOS^+$ 439.2066; found 439.2064, Δ 0.48 ppm.

S1 4-Oxo-4-(2,3,4,5,6-pentamethylphenyl)butanoic acid

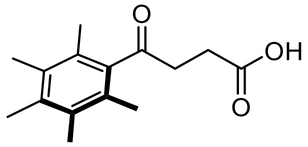

$AlCl_3$ (9.00 g, 67.0 mmol) was added portion-wise (over 5 min) to a stirred solution of succinic anhydride (3.10 g, 30.7 mmol) and pentamethylbenzene (5.00 g, 33.7 mmol) in CH_2Cl_2 (100 mL) at RT. The reaction was stirred for 5 minutes at RT and was then poured into cold 1 M HCl (100 mL). The layers were separated and the aqueous phase extracted with CH_2Cl_2 (2 × 100 mL). The combined organic layers were washed with 1 M HCl (2 × 100 mL), dried over $MgSO_4$ and concentrated *in vacuo*. Purification *via* flash column chromatography (eluent CH_2Cl_2/MeOH, 97:3 → 95:5) gave the *title compound* as a white solid (5.25 g, 68%).

^{1}**H NMR** (400 MHz, $CDCl_3$) δ 3.02 (t, *J* = 6.4 Hz, 2H), 2.78 (t, *J* = 6.4 Hz, 2H), 2.24 (s, 3H), 2.19 (s, 6H), 2.12 (s, 6H);

^{13}**C NMR** (101 MHz, $CDCl_3$) δ 209.6, 178.4 , 139.8, 135.8, 133.3, 127.7, 40.1, 27.6, 17.2, 16.8, 16.1;

m/z (ESI^+) Found $[M+Na]^+$ = 271, $C_{15}H_{20}NaO_3$ requires 271.

The spectroscopic data matched that previously reported in the literature.[6]

S2 4-Hydroxy-1-(2,3,4,5,6-Pentamethylphenyl)butan-1-one

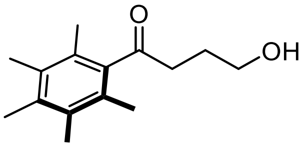

$BH_3{\cdot}SMe_2$ (3.1 mL, 33 mmol) was added dropwise (over 5 min) to a solution of acid **S1** (5.38 g, 21.7 mmol) in THF (100 mL) at 0 °C. The resulting solution allowed to warm slowly to RT overnight (16 h), subsequently cooled to 0 °C and then quenched with MeOH (16 mL) and water (40 mL). The mixture was concentrated *in* vacuo, diluted further with water (100 mL) and extracted with EtOAc (3 × 100 mL). The combined organic layers were washed with satd. aq. $NaHCO_3$, dried over $MgSO_4$ and concentrated *in vacuo*. Purification *via* flash column chromatography (eluent Pentane/Et_2O, 60:40 → 30:70) gave the *title compound* as a white solid (5.05 g, 99%).

^{1}**H NMR** (400 MHz, $CDCl_3$) δ 3.75 (t, *J* = 6.1 Hz, 2H), 2.82 (t, *J* = 6.9 Hz, 2H), 2.23 (s, 3H), 2.19 (s, 6H), 2.10 (s, 6H), 2.06 – 1.94 (m, 2H), 1.78 (s, 1H);

^{13}**C NMR** (101 MHz, $CDCl_3$) δ 212.6, 140.6, 135.6, 133.2, 127.4, 62.5, 42.6, 26.3, 17.3, 16.8, 16.1;

m/z (ESI^+) Found $[M+Na]^+$ = 257, $C_{15}H_{22}NaO_2$ requires 257.

The spectroscopic data matched that previously reported in the literature.[6]

6a 1-(2,3,4,5,6-Pentamethylphenyl)-4-phenoxybutan-1-one

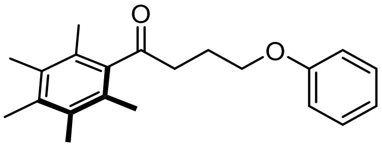

Phenol (1.39 g, 14.8 mmol) was added to a solution of alcohol **S2** (3.00 g, 12.3 mmol) in THF (40 mL). Triphenylphosphine (4.19 g, 16.0 mmol) and DTBAD (di-*tert*-butyl azodicarboxylate, 3.40 g, 14.8 mmol) were added and the reaction was stirred at RT for 2 h. The crude reaction mixture was concentrated *in vacuo* and triturated with Pentane/Et_2O (approx. 5:1), filtered (washed with pentane) and concentrated *in vacuo*. Purification *via* flash column chromatography (eluent Pentane/Et_2O, 98:2 → 95:5) and subsequent recrystallisation from a minimum amount of boiling hexane gave the *title compound* as a white solid (1.73 g, 45%).

m.p. = 74 – 75 °C;

^{1}H NMR (400 MHz, $CDCl_3$) δ 7.27 (dd, *J* = 8.7, 7.3 Hz, 2H), 6.94 (tt, *J* = 7.4, 1.0 Hz, 1H), 6.91 – 6.86 (m, 2H), 4.09 (t, *J* = 6.1 Hz, 2H), 2.91 (t, *J* = 7.0 Hz, 2H), 2.26 – 2.18 (m, 5H), 2.18 (s, 6H), 2.09 (s, 6H);

^{13}C NMR (126 MHz, $CDCl_3$) δ 211.6, 159.0, 140.7, 135.6, 133.2, 129.6, 127.4, 120.8, 114.6, 66.7, 41.8, 23.2, 17.3, 16.8, 16.1;

IR (film) ν_{max}/cm^{-1} 2934, 1699, 1600, 1493, 1473, 1245;

m/z (ESI$^+$) Found $[M+Na]^+$ = 333; $C_{21}H_{26}NaO_2$ requires 333;

HRMS (ESI$^+$) m/z: Calcd for $C_{21}H_{27}O_2^+$ 311.2006; found 311.2007, Δ 0.31 ppm.

6b 4-(4-Nitrophenoxy)-1-(2,3,4,5,6-pentamethylphenyl)butan-1-one

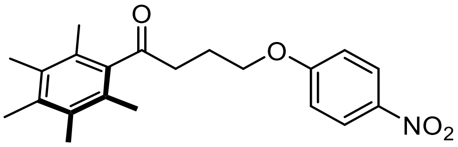

4-Nitrophenol (713 mg, 5.10 mmol), alcohol **S2** (1.00 g, 4.27 mmol), PPh_3 (1.47 g, 5.61 mmol) and di-(4 chlorobenzyl)azodicarboxylate (1.87 g, 5.13 mmol) were subjected to **General Procedure 2.** Purification *via* flash column chromatography (eluent Pentane/Et_2O, 90:10 → 80:20) gave a mixture of the *title compound* and 4-nitrophenol, which was purified

further *via* recrystallisation from a minimum amount of boiling MeOH to give the *title compound* as a white solid (554 mg, 36%).

m.p. = 89 – 90 °C;

^{1}H NMR (400 MHz, $CDCl_3$) δ 8.37 – 8.04 (m, 2H), 7.02 – 6.80 (m, 2H), 4.21 (t, *J* = 6.1 Hz, 2H), 2.90 (t, *J* = 6.8 Hz, 2H), 2.27 (q, *J* = 6.4 Hz, 2H), 2.23 (s, 3H), 2.18 (s, 6H), 2.08 (s, 6H);

^{13}C NMR (101 MHz, $CDCl_3$) δ 210.9, 163.9, 141.6, 140.3, 135.7, 133.2, 127.2, 126.0, 114.4, 67.6, 41.3, 22.8, 17.1, 16.7, 15.9;

IR (film) ν_{max}/cm^{-1} 2929, 1698, 1593, 1511, 1498, 1471, 1405, 1338, 1299, 1260;

m/z (ESI$^+$) Found $[M+H]^+$ = 356, $C_{21}H_{26}NO_4$ requires 356;

HRMS (ESI$^+$) m/z: $[M+H]^+$ Calcd for $C_{21}H_{26}NO_4^+$ 356.1856; found 356.1859, Δ 0.84 ppm.

6c 4-(3-Nitrophenoxy)-1-(2,3,4,5,6-pentamethylphenyl)butan-1-one

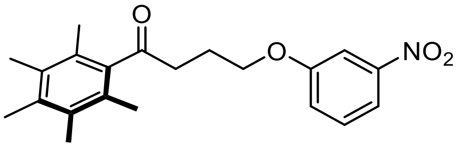

3-Nitrophenol (357 mg, 2.55 mmol), alcohol **S2** (500 mg, 2.14 mmol), PPh_3 (735 mg, 2.81 mmol) and di-(4-chlorobenzyl)azodicarboxylate (935 mg, 2.55 mmol) were subjected to **General Procedure 2.** Purification *via* flash column chromatography (eluent Pentane/Et_2O, 92:8 → 90:10) gave a mixture of the *title compound* and 3-nitrophenol, which was purified further *via* recrystallisation from a minimum amount of boiling MeOH to give the *title compound* as an off-white solid (275 mg, 38%).

m.p. = 87 – 89 °C;

^{1}H NMR (400 MHz, $CDCl_3$) δ 7.82 (ddd, *J* = 8.1, 2.1, 0.9 Hz, 1H), 7.72 (t, *J* = 2.3 Hz, 1H), 7.42 (t, *J* = 8.2 Hz, 1H), 7.22 (ddd, *J* = 8.3, 2.5, 0.9 Hz, 1H), 4.18 (t, *J* = 6.1 Hz, 2H), 2.91 (t, *J* = 7.0 Hz, 2H), 2.30 – 2.24 (m, 2H), 2.23 (s, 3H), 2.18 (s, 6H), 2.09 (s, 6H);

^{13}C NMR (151 MHz, $CDCl_3$) δ 211.0, 159.4, 149.3, 140.3, 135.6, 133.2, 130.0, 127.2, 121.4, 115.8, 108.9, 67.5, 41.4, 22.8, 17.1, 16.7, 16.0;

IR (film) ν_{max}/cm^{-1} 2935, 1699, 1529, 1471, 1405, 1350, 1321, 1286, 1247;

m/z (ESI$^+$) Found $[M+H]^+$ = 356, $C_{21}H_{26}NO_4$ requires 356;

HRMS (ESI$^+$) m/z: $[M+H]^+$ Calcd for $C_{21}H_{26}NO_4^+$ 356.1856; found 356.1858, Δ 0.56 ppm.

6d 4-(4-oxo-4-(2,3,4,5,6-pentamethylphenyl)butoxy)benzonitrile

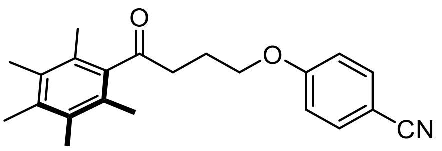

4-Cyanophenol (305 mg, 2.56 mmol), alcohol **S2** (500 mg, 2.15 mmol), PPh_3 (725 mg, 2.77 mmol) and di-(4-chlorobenzyl)azodicarboxylate (974 mg, 2.56 mmol) were subjected to **General Procedure 2.** Purification *via* flash column chromatography (eluent Pentane/Et_2O, 80:20 → 70:30) gave a mixture of the *title compound* and 4-cyanophenol, which was purified further *via* recrystallisation from a minimum amount of boiling MeOH to give the *title compound* as an off-white solid (265 mg, 35%).

m.p. = 117 – 120 °C;

^{1}H NMR (400 MHz, $CDCl_3$) δ 7.72 – 7.37 (m, 2H), 7.03 – 6.78 (m, 2H), 4.15 (t, *J* = 6.1 Hz, 2H), 2.89 (t, *J* = 6.9 Hz, 2H), 2.29 – 2.20 (m, 5H), 2.18 (s, 6H), 2.08 (s, 6H);

^{13}C NMR (101 MHz, $CDCl_3$) δ 211.1, 162.3, 140.4, 135.8, 134.2, 133.3, 127.3, 119.4, 115.3, 104.1, 67.2, 41.5, 22.9, 17.2, 16.8, 16.1;

IR (film) ν_{max}/cm^{-1} 2938, 2224, 1698, 1606, 1509, 1257, 1172, 836;

m/z (ESI$^+$) Found $[M+H]^+$ = 336, $C_{22}H_{26}NO_2$ requires 336;

HRMS (ESI$^+$) m/z: $[M+H]^+$ Calcd for $C_{22}H_{26}NO_2^+$ 336.1958; found 336.1960, Δ 0.59 ppm.

6e 1-(2,3,4,5,6-Pentamethylphenyl)-4-(4-(trifluoromethyl)phenoxy)butan-1-one

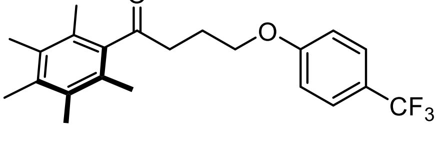

4-(Trifluoromethyl)phenol (415 mg, 2.56 mmol), alcohol **S2** (500 mg, 2.15 mmol), PPh_3 (725 mg, 2.77 mmol) and di-(4-chlorobenzyl)azodicarboxylate (974 mg, 2.65 mmol) were subjected to **General Procedure 2.** Purification *via* flash column chromatography (eluent Pentane/Et_2O, 97:3 → 95:5) gave a mixture of the *title compound* and

4-(trifluoromethyl)phenol, which was purified further *via* recrystallisation from a minimum amount of boiling MeOH to give the *title compound* as an off-white solid (213 mg, 26%).

m.p. = 88 – 90 °C;

^{1}H NMR (500 MHz, $CDCl_3$) δ 7.63 – 7.46 (m, 2H), 6.95 (d, *J* = 8.7 Hz, 2H), 4.14 (t, *J* = 6.1 Hz, 2H), 2.90 (t, *J* = 6.9 Hz, 2H), 2.30 – 2.21 (m, 5H), 2.18 (s, 6H), 2.09 (s, 6H);

^{13}C NMR ({^{19}F} 126 MHz, $CDCl_3$) δ 211.3, 161.4, 140.5, 135.7, 133.3, 127.4, 127.0, 124.6, 123.0, 114.5, 67.0, 41.6, 23.0, 17.2, 16.8, 16.1;

^{19}F NMR (471 MHz, $CDCl_3$) δ -61.5;

IR (film) ν_{max}/cm^{-1} 3020, 2936, 1699, 1616, 1328, 1257, 1111, 1069, 943, 837;

m/z (ESI$^+$) Found $[M+H]^+$ = 379, $C_{22}H_{26}F_3O_2$ requires 379;

HRMS (ESI$^+$) m/z: $[M+H]^+$ Calcd for $C_{22}H_{26}F_3O_2^+$ 379.1890; found 379.1880, Δ 2.63 ppm.

S3 4-(4-Methoxyphenoxy)butanoic acid

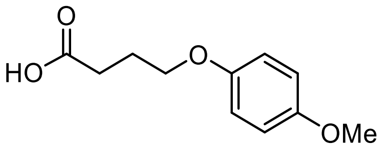

KOH (2.92 g, 52.0 mmol) was added to a stirred suspension of 4-methoxyphenol (6.20 g, 50.0 mmol) in water (2 mL). γ-Butyrolactone (7.7 mL, 100 mmol) was added, a Dean-Stark trap was attached and the mixture was heated at 150 °C for 2 h under N_2 and then at 190 °C for 18 h. After cooling to RT, 50 mL of water was added and the mixture was heated until all of the solid had dissolved. The resulting hot brown solution was poured into ice-cold HCl (1.5 M, 200 mL) and the mixture was cooled at 0 °C for 2 h. The crystals were collected by filtering under reduced pressure (washing with cold water) and then re-dissolved in CH_2Cl_2 (500 mL). The organic phase was dried over $MgSO_4$ and concentrated *in vacuo* to give the *title compound* as a white solid which required no further purification (7.97 g, 76%).

^{1}H NMR (400 MHz, $CDCl_3$) δ 6.83 (s, 4H), 3.97 (t, *J* = 6.1 Hz, 2H), 3.77 (s, 3H), 2.59 (t, *J* = 7.3 Hz, 2H), 2.17 – 2.01 (m, 2H);

^{13}C NMR (101 MHz, $CDCl_3$) δ 179.5, 154.0, 153.0, 115.6, 114.8, 67.3, 55.9, 30.7, 24.6;

m/z (ESI$^-$) Found $[M-H]^-$ = 209, $C_{11}H_{13}O_4$ requires 209.

The spectroscopic data matched that previously reported in the literature.[7]

6f 4-(4-Methoxyphenoxy)-1-(2,3,4,5,6-pentamethylphenyl)butan-1-one

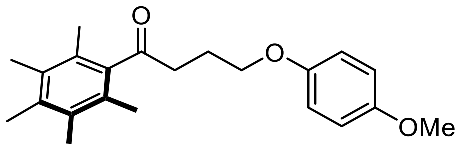

3-Methoxyphenol (320 mg, 2.58 mmol), alcohol **S2** (500 mg, 2.15 mmol), PPh_3 (735 mg, 2.81 mmol) and di-(4-chlorobenzyl)azodicarboxylate (935 mg, 2.55 mmol) were subjected to **General Procedure 2.** Purification *via* flash column chromatography (eluent Pentane/Et_2O, 95:5 → 92:8) gave the *title compound* as a white solid (180 mg, 25%).

Alternative Method: Carboxylic acid **S3** (4.50 g, 21.4 mmol) was dissolved in thionyl chloride (3.1 mL, 43 mmol) and the mixture was refluxed for 2 h. After cooling to RT, the reaction mixture was concentrated *in vacuo*. The crude acyl chloride was dissolved in CH_2Cl_2 (10 mL) and added to a solution of pentamethylbenzene (4.76 g, 32.1 mmol) in CH_2Cl_2 (100 mL) at 0 °C. $AlCl_3$ (5.71 g, 42.8 mmol) was added portion-wise and to mixture was stirred at RT for 1 h. The crude reaction mixture was poured onto ice (approx. 150 g) and the layers were separated. The aqueous layer was extracted with CH_2Cl_2 (2 × 150 mL) and the combined organic layers were washed with brine, dried over Na_2SO_4 and concentrated *in vacuo*. Purification *via* flash column chromatography (eluent Pentane: Et_2O, 96:4 → 90:10) gave a pale yellow solid, which was recrystallised from a minimum amount of boiling hexane to give the *title compound* as a white solid (3.58 g, 49%).

m.p. = 72 – 74 °C;

^{1}H NMR (400 MHz, $CDCl_3$) δ 6.83 (s, 4H), 4.04 (t, *J* = 6.1 Hz, 2H), 3.77 (s, 3H), 2.90 (t, *J* = 7.0 Hz, 2H), 2.24 (s, 3H), 2.22 – 2.15 (m, 8H), 2.10 (s, 6H);

^{13}C NMR (101 MHz, $CDCl_3$) δ 211.5, 154.0, 153.2, 140.7, 135.6, 133.2, 127.4, 115.6, 114.8, 67.5, 55.9, 41.8, 23.3, 17.2, 16.8, 16.1;

IR (film) ν_{max}/cm^{-1} 2935, 1699, 1508, 1231, 1041, 824;

m/z (ESI$^+$) Found [M+H]$^+$ = 336, $C_{22}H_{29}O_3$ requires 341;

HRMS (ESI$^+$) m/z: [M+H]$^+$ Calcd for $C_{22}H_{29}O_3^+$ 341.2109; found 341.2112, Δ 0.88 ppm.

7 4-(4-Methoxyphenoxy)-1-(2,3,5,6-tetramethylphenyl)butan-1-one

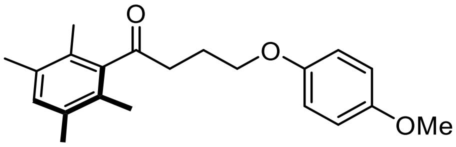

Carboxylic acid **S3** (5.00 g, 23.8 mmol) was dissolved in thionyl chloride (3.5 mL, 48 mmol) and the mixture was refluxed for 3 h. After cooling to RT, the reaction mixture was concentrated *in vacuo*. The crude acyl chloride was dissolved in CH_2Cl_2 (20 mL) and added to a solution of durene (4.83 g, 36.0 mmol) in CH_2Cl_2 (100 mL) at 0 °C. $AlCl_3$ (6.38 g, 48.0 mmol) was added portion-wise and to mixture was stirred at RT for 1 h. The crude reaction mixture was poured onto ice (approx. 150 g) and the layers were separated. The aqueous layer was extracted with CH_2Cl_2 (2 × 150 mL) and the combined organic layers were washed with brine, dried over Na_2SO_4 and concentrated *in vacuo*. Purification *via* flash column chromatography (eluent Pentane: Et_2O, 96:4 → 90:10) gave a pale yellow solid, which was recrystallised from a minimum amount of boiling hexane to give the *title compound* as a white solid (3.75 g, 48%).

m.p. = 59 – 61 °C;

^{1}H NMR (400 MHz, $CDCl_3$) δ 6.95 (s, 1H), 6.83 (s, 4H), 4.03 (t, *J* = 6.1 Hz, 2H), 3.77 (s, 3H), 2.90 (t, *J* = 7.0 Hz, 2H), 2.24 – 2.15 (m, 8H), 2.05 (s, 6H);

^{13}C NMR (101 MHz, $CDCl_3$) δ 211.4, 154.0, 153.1, 142.8, 134.5, 131.7, 128.1, 115.6, 114.8, 67.5, 55.9, 41.6, 23.2, 19.6, 16.0;

IR (film) ν_{max}/cm^{-1} 2936, 1698, 1508, 1470, 1291, 1231, 1040, 825;

m/z (ESI$^+$) Found $[M+H]^+$ = 327, $C_{21}H_{27}O_3$ requires 327;

HRMS (ESI$^+$) m/z: $[M+H]^+$ Calcd for $C_{21}H_{27}O_3^+$ 327.1955; found 327.1954, Δ 0.21 ppm.

8a (1-Benzylcyclopropyl)(2,3,5,6-tetramethylphenyl)methanone

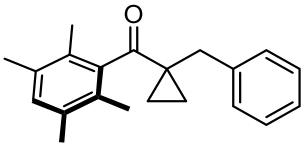

Ir-catalysed: Ketone **7** (196 mg, 0.600 mmol), $[Ir(cod)Cl]_2$ (4.0 mg, 1.0 mol%), dppBz (6.0 mg, 2.0 mol%) and KOH (135 mg, 2.40 mmol) were added to a 2-5 mL microwave vial. The vial was sealed, evacuated and backfilled with Ar (three times) and benzyl alcohol (0.62 mL, 6.00

mmol, degassed with Ar (balloon)) was added. The reaction was heated at 110 °C for 24 h, cooled to RT and KOH (67 mg, 1.2 mmol) and ^{t}BuOH (3.0 mL) were added. The reaction was heated at 110 °C for a further 24 h, cooled to RT, filtered through a silica plug (Et_2O elution) and concentrated *in vacuo*. Purification *via* flash column chromatography (eluent Pentane/Et_2O, 98:2 → 95:5) gave the *title compound* as a colourless oil (133 mg, 72%).

Ru-catalysed: Ketone **7** (98 mg, 0.30 mmol), Ru-MACHO® (3.6 mg, 2.0 mol%) and KOH (67 mg, 1.2 mmol) were added to a 2-5 mL microwave vial. The vial was sealed, evacuated and backfilled with Ar (three times) and and benzyl alcohol (0.31 mL, 3.00 mmol, degassed with Ar (balloon)) was added. The reaction was heated at 110 °C for 24 h, cooled to RT and KOH (34 mg, 0.6 mmol) and ^{t}BuOH (1.5 mL) were added. The reaction was heated at 110 °C for a further 24 h, cooled to RT, filtered through a silica plug (Et_2O elution) and concentrated *in vacuo*. Purification *via* flash column chromatography (eluent Pentane/Et_2O, 98:2 → 95:5) gave the *title compound* as a colourless oil (42 mg, 48%).

Scale-up: Ketone **7** (326 mg, 1.00 mmol), $[Ir(cod)Cl]_2$ (6.7 mg, 1.0 mol%), dppBz (8.9 mg, 2.0 mol%) and KOH (224 mg, 4.00 mmol) were added to a 10-20 mL microwave vial. The vial was sealed, evacuated and backfilled with Ar (three times) and benzyl alcohol (1.03 mL, 10.0 mmol, degassed with Ar (balloon)) was added. The reaction was heated at 110 °C for 24 h, cooled to RT and KOH (112 mg, 2.00 mmol) and ^{t}BuOH (5.0 mL) were added. The reaction was heated at 110 °C for a further 24 h, cooled to RT, filtered through a silica plug (Et_2O elution) and concentrated *in vacuo*. Purification *via* flash column chromatography (eluent Pentane/Et_2O, 98:2) gave the *title compound* as a colourless oil (229 mg, 78%).

^{1}H NMR (400 MHz, $CDCl_3$) δ 7.24 – 7.14 (m, 3H), 7.12 – 7.01 (m, 2H), 6.91 (s, 1H), 3.03 (s, 2H), 2.14 (s, 6H), 1.90 (s, 6H), 1.28 – 1.17 (m, 2H), 1.05 – 0.97 (m, 2H);

^{13}C NMR (101 MHz, $CDCl_3$) δ 212.5, 139.6, 139.1, 134.2, 131.5, 130.0, 129.2, 128.0, 126.4, 37.9, 34.2, 19.6, 17.5, 16.4;

IR (film) ν_{max}/cm^{-1} 2922, 2862, 1671, 1469, 700;

m/z (ESI^+) Found $[M+H]^+$ = 293, $C_{21}H_{25}O$ requires 294;

HRMS (ESI^+) m/z: $[M+H]^+$ Calcd for $C_{21}H_{25}O^+$ 293.1900; found 293.1900, Δ 0.04 ppm.

Single Crystal Data for **8a**: C21H24O, Mr =292.42. 100 K – monoclinic, P 2_1/n, a = 12.6727(4) Å, b = 8.6802(3) Å, c = 14.8889(4) Å, β = 91.672(3)°, V = 1637.11(9) Å^3, Data/restraints/parameters – 4056/0/199, Rint = 0.028, Final R1 = 0.0393, wR2 = 0.0924 (I>2σ(I)).

8b (1-(4-Methoxybenzyl)cyclopropyl)(2,3,5,6-tetramethylphenyl)methanone

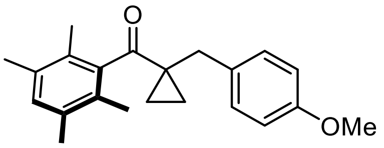

Ketone **7** (98 mg, 0.30 mmol), $[Ir(cod)Cl]_2$ (2.0 mg, 1.0 mol%), dppBz (3.0 mg, 2.0 mol%), KOH (67 mg, 1.2 mmol) and anisyl alcohol (0.37 mL, 3.0 mmol) were subjected to **General Procedure 1** at 110 °C. Purification *via* flash column chromatography (eluent Pentane/Et_2O, 97:3 → 95:5) gave the *title compound* as a colourless oil which solidified on standing (80 mg, 83%).

m.p. = 92 – 94 °C;

^{1}H NMR (400 MHz, $CDCl_3$) δ 7.03 – 6.93 (m, 2H), 6.90 (s, 1H), 6.78 – 6.66 (m, 2H), 3.78 (s, 3H), 2.96 (s, 2H), 2.15 (s, 6H), 1.91 (s, 6H), 1.26 – 1.16 (m, 2H), 1.10 – 0.88 (m, 2H).

^{13}C NMR (101 MHz, $CDCl_3$) δ 212.6, 158.2, 139.8, 134.2, 131.5, 131.1, 131.0, 129.2, 113.4, 55.4, 37.0, 34.4, 19.6, 17.4, 16.4;

IR (film) ν_{max}/cm^{-1} 3006, 2917, 1671, 1611 , 1511, 1247;

m/z (ESI$^+$) Found $[M+Na]^+$ = 345, $C_{22}H_{26}O_2Na$ requires 345;

HRMS (ESI$^+$) m/z: $[M+Na]^+$ Calcd for $C_{22}H_{26}O_2Na^+$ 345.1825; found 345.1831, Δ 1.70 ppm.

8c (2,3,5,6-Tetramethylphenyl)(1-(3-(trifluoromethyl)benzyl)cyclopropyl)methanone

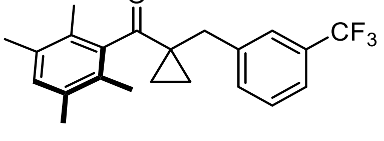

Ir-catalysed: Ketone **7** (98 mg, 0.30 mmol), $[Ir(cod)Cl]_2$ (2.0 mg, 1.0 mol%), dppBz (3.0 mg, 2.0 mol%) and KOH (67 mg, 1.2 mmol) were added to a 2-5 mL microwave vial. The vial was

sealed, evacuated and backfilled with Ar (three times) and (3-(trifluoromethyl)phenyl)methanol (0.40 mL, 3.0 mmol, degassed with Ar (balloon)) was added. The reaction was heated at 110 °C for 24 h, cooled to RT, and KOH (34 mg, 0.15 mmol) and ^{t}BuOH (1.5 mL) were added. The reaction was heated at 110 °C for a further 24 h, cooled to RT, filtered through a silica plug (Et_2O elution) and concentrated *in vacuo*. Purification *via* flash column chromatography (eluent Pentane/Et_2O, 98.5:1.5 → 98:2) gave the *title compound* as a colourless oil (67 mg, 62%).

Ru-catalysed: Ketone **7** (98 mg, 0.30 mmol), Ru-MACHO® (3.6 mg, 2.0 mol%) and KOH (67 mg, 1.2 mmol) were added to a 2-5 mL microwave vial. The vial was sealed, evacuated and backfilled with Ar (three times) and (3-(trifluoromethyl)phenyl)methanol (0.40 mL, 3.0 mmol, degassed with Ar (balloon)) was added. The reaction was heated at 110 °C for 24 h, cooled to RT, and KOH (34 mg, 0.15 mmol) and ^{t}BuOH (1.5 mL) were added. The reaction was heated at 110 °C for a further 24 h, cooled to RT, filtered through a silica plug (Et_2O elution) and concentrated *in vacuo*. Purification *via* flash column chromatography (eluent Pentane/Et_2O, 98.5:1.5 → 98:2) gave the *title compound* as a colourless oil (70 mg, 65%).

Scale-up: Ketone **7** (326 mg, 1.00 mmol), $[Ir(cod)Cl]_2$ (6.7 mg, 1.0 mol%), dppBz (8.9 mg, 2.0 mol%) and KOH (224 mg, 4.00 mmol) were added to a 10-20 mL microwave vial. The vial was sealed, evacuated and backfilled with Ar (three times) and (3-(trifluoromethyl)phenyl)methanol (1.36 mL, 10.0 mmol, degassed with Ar (balloon)) was added. The reaction was heated at 110 °C for 24 h, cooled to RT, and KOH (112 mg, 2.00 mmol) and ^{t}BuOH (5.0 mL) were added. The reaction was heated at 110 °C for a further 24 h, cooled to RT, filtered through a silica plug (Et_2O elution) and concentrated *in vacuo*. Purification *via* flash column chromatography (eluent Pentane/Et_2O, 98:2) gave the *title compound* as a colourless oil (243 mg, 68%).

^{1}H NMR (500 MHz, $CDCl_3$) δ 7.45 – 7.42 (m, 1H), 7.36 – 7.30 (m, 3H), 6.90 (s, 1H), 3.05 (s, 2H), 2.12 (s, 6H), 1.87 (s, 6H), 1.30 (q, *J* = 4.1 Hz, 2H), 1.12 – 1.02 (m, 2H);

^{13}C NMR ({^{19}F} 126 MHz, $CDCl_3$) δ 211.8, 140.5, 139.2, 134.4, 133.2, 131.8, 130.4, 129.1, 128.5, 126.3, 124.3, 123.2, 38.4, 34.3, 19.5, 17.9, 16.3;

^{19}F NMR (471 MHz, $CDCl_3$) δ -62.6;

IR (film) ν_{max}/cm^{-1} 2925, 2866, 1672, 1450, 1329, 1198, 1163, 1125;

m/z (ESI^+) Found $[M+H]^+$ = 361, $C_{22}H_{24}F_3O$ requires 361;

HRMS (ESI^+) m/z: $[M+Na]^+$ Calcd for $C_{22}H_{23}F_3NaO^+$ 383.1593; found 383.1607, Δ 3.59 ppm.

8d (1-Butylcyclopropyl)(2,3,5,6-tetramethylphenyl)methanone

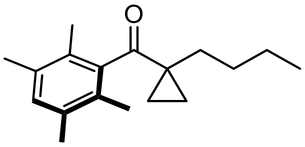

Ir-catalysed: Ketone **7** (98 mg, 0.30 mmol), $[Ir(cod)Cl]_2$ (2.0 mg, 1.0 mol%), dppBz (3.0 mg, 2.0 mol%), KOH (67 mg, 1.2 mmol) and *n*-butanol (0.27 mL, 3.0 mmol) were subjected to **General Procedure 1** at 90 °C. Purification *via* flash column chromatography (eluent Pentane/Et_2O, 99:1 → 98.5:1.5) gave the *title compound* as a colourless oil (64 mg, 82%).

Ru-catalysed: Ketone **7** (98 mg, 0.30 mmol) Ru-MACHO® (3.6 mg, 2.0 mol%), KOH (67 mg, 1.2 mmol) and *n*-butanol (0.27 mL, 3.0 mmol) were subjected to **General Procedure 1** at 90 °C. Purification *via* flash column chromatography (eluent Pentane/Et_2O, 99:1 → 98.5:1.5) gave the *title compound* as a colourless oil (19 mg, 25%).

^{1}H NMR (400 MHz, $CDCl_3$) δ 6.91 (s, 1H), 2.17 (s, 6H), 2.05 (s, 6H), 1.68 – 1.59 (m, 2H), 1.47 – 1.35 (m, 2H), 1.35 – 1.23 (m, 2H), 1.10 (q, *J* = 3.8 Hz, 2H), 0.96 – 0.90 (m, 2H), 0.86 (t, *J* = 7.3 Hz, 3H);

^{13}C NMR (101 MHz, $CDCl_3$) δ 212.6, 139.3, 134.2, 131.4, 129.2, 34.1, 33.4, 29.6, 23.2, 19.6, 17.9, 16.6, 14.1;

IR (film) ν_{max}/cm^{-1} 2956, 2932, 2861, 1670, 1468, 964;

m/z (ESI$^+$) Found $[M+H]^+$ = 259, $C_{18}H_{27}O$ requires 259;

HRMS (ESI$^+$) m/z: $[M+H]^+$ Calcd for $C_{18}H_{27}O^+$ 259.2054; found 259.2057, Δ 0.95 ppm.

8e (1-(Cyclopropylmethyl)cyclopropyl)(2,3,5,6-tetramethylphenyl)methanone

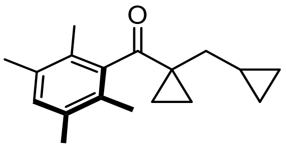

Ketone **7** (196 mg, 0.600 mmol), $[Ir(cod)Cl]_2$ (4.0 mg, 1.0 mol%), dppBz (6.0 mg, 2.0 mol%) and KOH (135 mg, 1.20 mmol) were added to a 2-5 mL microwave vial. The vial was sealed, evacuated and backfilled with Ar (three times) and cyclopropanemethanol (0.55 mL, 6.0 mmol, degassed with Ar (balloon)) was added. The reaction was heated at 110 °C for 24 h, cooled to RT, filtered through a silica plug (Et_2O elution) and concentrated *in vacuo*.

Purification *via* flash column chromatography (eluent Pentane/Et_2O, 98:2) gave the *title compound* as a colourless oil (121 mg, 79%).

^{1}H NMR (400 MHz, $CDCl_3$) δ 6.91 (s, 1H), 2.18 (s, 6H), 2.07 (s, 6H), 1.64 (d, *J* = 6.9 Hz, 2H), 1.19 – 1.10 (m, 2H), 1.10 – 1.01 (m, 2H), 0.84 – 0.67 (m, 1H), 0.46 – 0.26 (m, 2H), 0.03 (dd, *J* = 5.0, 1.4 Hz, 2H).

^{13}C NMR (101 MHz, $CDCl_3$) δ 212.9, 139.6, 134.2, 131.5, 129.2, 37.1, 34.0, 19.6, 17.1, 16.5, 8.6, 4.5;

IR (film) ν_{max}/cm^{-1} 3078, 3003, 2923, 1671;

m/z (ESI^+) Found $[M+H]^+$ = 257, $C_{18}H_{24}O$ requires 257;

HRMS (ESI^+) m/z: $[M+H]^+$ Calcd for $C_{18}H_{24}O^+$ 257.1900; found 257.1900, Δ 0.08 ppm.

8f (1-Isopentylcyclopropyl)(2,3,5,6-tetramethylphenyl)methanone

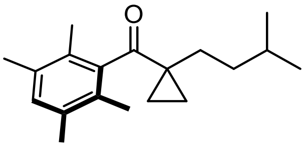

Ir-catalysed: Ketone **7** (98 mg, 0.30 mmol), $[Ir(cod)Cl]_2$ (2.0 mg, 1.0 mol%), dppBz (3.0 mg, 2.0 mol%), KOH (67 mg, 1.2 mmol) and 3-methylbutan-1-ol (0.35 mL, 3.0 mmol) were subjected to **General Procedure 1** at 90 °C. Purification *via* flash column chromatography (eluent Pentane/Et_2O, 99.5:0.5 → 99:1) gave the *title compound* as a white solid (64 mg, 79%).

Ru-catalysed: Ketone **7** (98 mg, 0.30 mmol), Ru-MACHO® (3.6 mg, 2.0 mol%), KOH (67 mg, 1.2 mmol) and 3-methylbutan-1-ol (0.35 mL, 3.0 mmol) were subjected to **General Procedure 1** at 90 °C. Purification *via* flash column chromatography (eluent Pentane/Et_2O, 99.5:0.5 → 99:1) gave the *title compound* as a white solid (28 mg, 35%).

m.p. = 48 – 50 °C;

^{1}H NMR (500 MHz, $CDCl_3$) δ 6.91 (s, 1H), 2.17 (s, 6H), 2.05 (s, 6H), 1.67 – 1.58 (m, 2H), 1.51 – 1.39 (m, 1H), 1.37 – 1.23 (m, 2H), 1.11 (q, *J* = 4.0 Hz, 2H), 0.91 (q, *J* = 4.0 Hz, 2H), 0.83 (d, *J* = 6.6 Hz, 6H);

^{13}C NMR (126 MHz, $CDCl_3$) δ 212.6, 139.3, 134.2, 131.4, 129.2, 36.4, 34.1, 31.5, 28.4, 22.6, 19.6, 18.0, 16.6;

IR (film) ν_{max}/cm^{-1} 2953, 2869, 1670, 1468, 966;

m/z (ESI$^+$) Found [M+H]$^+$ = 273, $C_{19}H_{29}O$ requires 273;

HRMS (ESI$^+$) m/z: [M+H]$^+$ Calcd for $C_{19}H_{29}O^+$ 273.2211; found 273.2213 Δ 0.59 ppm.

8g (1-(4-(Benzyloxy)butyl)cyclopropyl)(2,3,5,6-tetramethylphenyl)methanone

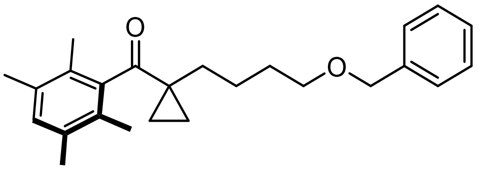

Ketone **7** (98 mg, 0.30 mmol), $[Ir(cod)Cl]_2$ (2.0 mg, 1.0 mol%), dppBz (3.0 mg, 2.0 mol%), KOH (67 mg, 1.2 mmol) and 4-benzyloxybutan-1-ol (0.53 mL, 3.0 mmol) were subjected to **General Procedure 1** at 110 °C. Purification *via* flash column chromatography (eluent Pentane/Et_2O, 95:5 → 94:6) gave the *title compound* as a colourless oil (87 mg, 80%).

Scale-up: Ketone **7** (326 mg, 1.00 mmol), $[Ir(cod)Cl]_2$ (6.7 mg, 1.0 mol%), dppBz (8.9 mg, 2.0 mol%), KOH (224 mg, 4.0 mmol) were added to a 2-5 mL microwave vial. The vial was sealed, evacuated and backfilled with Ar (three times) and 4-benzyloxybutanol (1.75 mL, 10.0 mmol, degassed with Ar (balloon)) was added. The reaction was heated at 110 °C for 24 h, cooled to RT, filtered through a silica plug (Et_2O elution) and concentrated *in vacuo*. Purification *via* flash column chromatography (eluent Pentane/Et_2O, 95:5) gave the *title compound* as a colourless oil (269 mg, 83%).

^{1}H NMR (400 MHz, $CDCl_3$) δ 7.38 – 7.27 (m, 5H), 6.90 (s, 1H), 4.49 (s, 2H), 3.44 (t, *J* = 6.4 Hz, 2H), 2.16 (s, 6H), 2.05 (s, 6H), 1.69 – 1.45 (m, 6H), 1.10 (q, *J* = 3.6 Hz, 2H), 1.01 – 0.88 (m, 2H);

^{13}C NMR (101 MHz, $CDCl_3$) δ 212.5, 139.1, 138.8, 134.2, 131.5, 129.2, 128.5, 127.8, 127.6, 73.0, 70.4, 34.0, 33.5, 30.2, 24.0, 19.6, 17.8, 16.6;

IR (film) ν_{max}/cm^{-1} 3029, 2939, 2861, 1669, 1469, 1105, 736, 698;

m/z (ESI$^+$) Found [M+H]$^+$ = 365, $C_{25}H_{33}O_2$ requires 365;

HRMS (ESI$^+$) m/z: [M+H]$^+$ Calcd for $C_{25}H_{33}O_2^+$ 365.2475; found 365.2474, Δ 0.36 ppm.

8h (1-((1-Benzylpiperidin-4-yl)methyl)cyclopropyl)(2,3,5,6-tetramethylphenyl)methanone

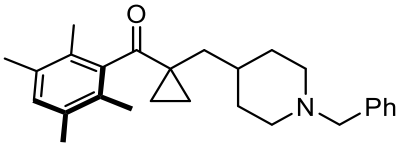

Ketone **7** (98 mg, 0.30 mmol), $[Ir(cod)Cl]_2$ (2.0 mg, 1.0 mol%), dppBz (3.0 mg, 2.0 mol%), KOH (67 mg, 1.2 mmol) and 1-benzyl-4-piperidyl methanol (616 mg, 3.00 mmol) were subjected to **General Procedure 1** at 110 °C. Purification *via* flash column chromatography (eluent Pentane/Et_2O, 50:50) gave the *title compound* as a colourless oil which solidified on standing to give an off-white solid (62 mg, 53%).

m.p. = 85 – 87 °C;

^{1}H NMR (400 MHz, $CDCl_3$) δ 7.35 – 7.21 (m, 5H), 6.90 (s, 1H), 3.48 (s, 2H), 2.86 (d, *J* = 11.1 Hz, 2H), 2.17 (s, 6H), 2.05 (s, 6H), 1.95 – 1.86 (m, 2H), 1.84 – 1.61 (m, 3H), 1.57 (d, *J* = 6.3 Hz, 2H), 1.33 – 1.18 (m, 2H), 1.10 (q, *J* = 4.0 Hz, 2H), 0.93 (m, 2H);

^{13}C NMR (151 MHz, $CDCl_3$) δ 212.3, 139.2, 138.6 br, 134.3, 131.5, 129.4, 129.2, 128.3, 127.1, 63.6, 54.0, 40.8, 34.5, 33.3, 32.2, 19.6, 18.0, 16.7;

IR (film) ν_{max}/cm^{-1} 2922, 2799, 2756, 1669, 738, 699;

m/z (ESI$^+$) Found $[M+H]^+$ = 390, $C_{27}H_{36}NO$ requires 390;

HRMS (ESI$^+$) m/z: $[M+Na]^+$ Calcd for $C_{27}H_{35}NNaO^+$ 412.2611; found 412.2625, Δ 3.40 ppm.

8i (1-(4-(Methylthio)butyl)cyclopropyl)(2,3,5,6-tetramethylphenyl)methanone

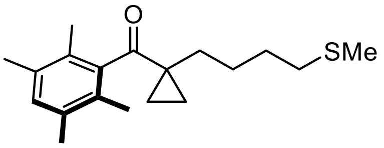

Ir-catalysed: Ketone **7** (98 mg, 0.30 mmol), $[Ir(cod)Cl]_2$ (2.0 mg, 1.0 mol%), dppBz (3.0 mg, 2.0 mol%), KOH (67 mg, 1.2 mmol) and 4-(methylthio)butan-1-ol (0.36 mL, 3.0 mmol) were subjected to **General Procedure 1** at 110 °C. Purification *via* flash column chromatography (eluent Pentane/Et_2O, 98:2 → 96:4) gave the *title compound* as a colourless oil (39 mg, 43%).

Ru-catalysed: Ketone **7** (98 mg, 0.30 mmol), Ru-MACHO® (3.6 mg, 2.0 mol%), KOH (67 mg, 1.2 mmol) and 4-(methylthio)butan-1-ol (0.36 mL, 3.0 mmol) were subjected to **General**

Procedure 1 at 110 °C. Purification *via* flash column chromatography (eluent Pentane/Et_2O, 98:2 → 96:4) gave the *title compound* as a colourless oil (31 mg, 36%).

^{1}H NMR (400 MHz, $CDCl_3$) δ 6.91 (s, 1H), 2.46 (t, *J* = 6.9 Hz, 2H), 2.17 (s, 6H), 2.07 (s, 3H), 2.05 (s, 6H), 1.68 – 1.61 (m, 2H), 1.61 – 1.49 (m, 4H), 1.14 – 1.06 (m, 2H), 0.98 – 0.89 (m, 2H).

^{13}C NMR (101 MHz, $CDCl_3$) δ 212.5, 139.0, 134.3, 131.5, 129.2, 34.2, 34.0, 33.3, 29.6, 26.6, 19.6, 17.9, 16.6, 15.7;

IR (film) ν_{max}/cm^{-1} 2938, 2861, 1670, 1469, 1097, 965, 872, 760;

m/z (ESI$^+$) Found $[M+Na]^+$ = 327, $C_{19}H_{28}NaOS$ requires 327;

HRMS (ESI$^+$) m/z: $[M+H]^+$ Calcd for $C_{19}H_{29}OS^+$ 305.1934; found 305.1943, Δ 3.06 ppm.

8j (1-(3-(Pyridin-3-yl)propyl)cyclopropyl)(2,3,5,6-tetramethylphenyl)methanone

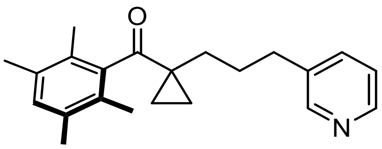

Ir-catalysed: Ketone **7** (98 mg, 0.30 mmol), $[Ir(cod)Cl]_2$ (2.0 mg, 1.0 mol%), dppBz (3.0 mg, 2.0 mol%), KOH (67 mg, 1.2 mmol) and 3-pyridinepropanol (0.39 mL, 3.0 mmol) were subjected to **General Procedure 1** at 110 °C. Purification *via* flash column chromatography (eluent Pentane/Et_2O, 40:60 → 20:80) gave the *title compound* as a colourless oil (60 mg, 62%).

Ru-catalysed: Ketone **7** (98 mg, 0.30 mmol), Ru-MACHO® (3.6 mg, 2.0 mol%), KOH (67 mg, 1.2 mmol) and 3-pyridinepropanol (0.39 mL, 3.0 mmol) were subjected to **General Procedure 1** at 110 °C. Purification *via* flash column chromatography (eluent Pentane/Et_2O, 40:60 → 20:80) gave the *title compound* as a colourless oil (60 mg, 62%).

^{1}H NMR (500 MHz, $CDCl_3$) δ 8.43 (d, *J* = 4.9 Hz, 1H), 8.39 (d, *J* = 2.3 Hz, 1H), 7.43 (dd, *J* = 7.8, 2.5 Hz, 1H), 7.19 (dd, *J* = 7.8, 4.8 Hz, 1H), 6.91 (s, 1H), 2.58 (t, *J* = 7.6 Hz, 2H), 2.16 (s, 6H), 2.03 (s, 6H), 1.78 (td, *J* = 9.7, 6.1 Hz, 2H), 1.69 – 1.61 (m, 2H), 1.12 (q, *J* = 4.1 Hz, 2H), 0.90 (q, *J* = 4.2 Hz, 2H).

^{13}C NMR (126 MHz, $CDCl_3$) δ 212.3, 149.9, 147.2, 139.0, 137.6, 136.0, 134.3, 131.6, 129.1, 123.5, 33.8, 33.5, 33.3, 28.7, 19.6, 18.0, 16.6;

IR (film) ν_{max}/cm^{-1} 2938, 2862, 1667, 1470, 1377, 1343, 1308, 715;

m/z (ESI$^+$) Found $[M+H]^+$ = 322, $C_{22}H_{28}NO$ requires 322;

HRMS (ESI$^+$) m/z: $[M+H]^+$ Calcd for $C_{22}H_{28}NO^+$ 322.2165; found 322.2164, Δ 0.44 ppm.

S4 4-Phenyldihydrofuran-2(3*H*)-one

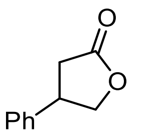

$[Rh(cod)Cl]_2$ (21 mg, 1.5 mol%) was dissolved in PhMe (65 mL) and stirred for 5 mins. KOH (2.0 M, 7.0 mL, 14 mmol) was added and the mixture was stirred vigorously for 30 min. Phenylboronic acid (4.47 g, 36.0 mmol) and furan-2(5*H*)-one (2.0 mL, 28 mmol) was added and the mixture was heated at 50 °C for 24 h. After cooling to RT, the reaction mixture was diluted with sat. aq. $NaHCO_3$ (150 mL) and CH_2Cl_2 (100 mL), the layers separated and the aqueous layer extracted with CH_2Cl_2 (2 × 100 mL). The combined organic layers were washed with brine, dried over Na_2SO_4 and concentrated *in vacuo*. Purification *via* flash column chromatography (eluent Pentane/Et_2O, 65:35 → 50:50) gave the *title compound* as an off-white solid (2.87 g, 63%).

^{1}H NMR (400 MHz, $CDCl_3$) δ 7.40 – 7.34 (m, 2H), 7.33 – 7.28 (m, 1H), 7.26 – 7.21 (m, 2H), 4.67 (dd, *J* = 9.1, 7.8 Hz, 1H), 4.27 (dd, *J* = 9.1, 7.9 Hz, 1H), 3.79 (p, *J* = 8.4 Hz, 1H), 2.93 (dd, *J* = 17.5, 8.7 Hz, 1H), 2.68 (dd, *J* = 17.5, 9.1 Hz, 1H);

^{13}C NMR (101 MHz, $CDCl_3$) δ 176.5, 139.5, 129.3, 127.8, 126.8, 74.2, 41.2, 35.8;

m/z (ESI$^+$) Found $[M+H]^+$ = 163, $C_{10}H_{11}O$ requires 163.

The spectral data matched that previously reported in the literature.[8]

S5 4-(4-Methoxyphenoxy)-3-phenylbutanoic acid

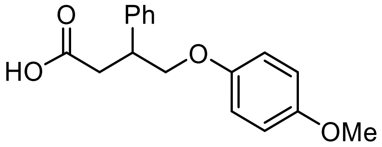

KOH (561 mg, 10.0 mmol) was added to a stirred suspension of 4-methoxyphenol (1.13 g, 9.10 mmol) in water (0.4 mL). Lactone **S4** (1.62 g, 10.0 mmol) was added and the reaction was heated to 150 °C for 2 h, then 190 °C for 16 h. After cooling to RT, the reaction mixture was

diluted with 1.5 M HCl (20 mL) and CH_2Cl_2 (20 mL) and the layers separated. The organic layer was basified using 3 M NaOH and the layers separated. The aqueous layer was acidified using 3 M HCl, and extracted with CH_2Cl_2 (3 × 100 mL). The combined organic extracts were washed with brine, dried over Na_2SO_4 and concentrated *in vacuo*. Purification *via* flash column chromatography (eluent CH_2Cl_2/EtOAc, 95:5 → CH_2Cl_2/MeOH 98:2) gave the *title compound* as a tan solid (290 mg, 10%);

m.p. = 102 – 103 °C;

^{1}H NMR (400 MHz, $CDCl_3$) δ 7.39 – 7.26 (m, 5H), 6.80 (s, 4H), 4.10 (dd, *J* = 9.3, 5.2 Hz, 1H), 3.97 (dd, *J* = 9.3, 8.1 Hz, 1H), 3.76 (s, 3H), 3.67 – 3.53 (m, 1H), 3.07 (dd, *J* = 16.2, 6.5 Hz, 1H), 2.77 (dd, *J* = 16.2, 8.2 Hz, 1H);

^{13}C NMR (101 MHz, $CDCl_3$) δ 176.9, 154.0, 152.7, 140.5, 128.7, 127.8, 127.3, 115.5, 114.6, 72.0, 55.7, 41.6, 37.0;

IR (film) ν_{max}/cm^{-1} 3031, 2919, 1708, 1509, 1230;

m/z (ESI$^-$) Found [M-H]$^-$ = 285, $C_{17}H_{17}O_4$ requires 285;

HRMS (ESI$^-$) m/z: [M-H]$^-$ Calcd for $C_{17}H_{17}O_4^-$ 285.1132; found 285.1133, Δ 0.24 ppm.

9 4-(4-Methoxyphenoxy)-3-phenyl-1-(2,3,5,6-tetramethylphenyl)butan-1-one

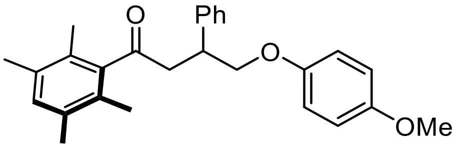

Carboxylic acid **S5** (250 mg, 0.900 mmol) was dissolved in thionyl chloride (0.15 mL, 2.0 mmol) and the mixture was refluxed for 3 h. After cooling to RT, the reaction mixture was concentrated *in vacuo*. The crude acyl chloride was dissolved in CH_2Cl_2 (3.0 mL), durene (238 mg, 1.40 mmol) was added and the mixture cooled to 0 °C. $AlCl_3$ (240 mg, 1.80 mmol) was added portion-wise and to mixture was stirred at RT for 2 h. The crude reaction mixture was poured onto ice (approx. 10 g) and the layers were separated. The aqueous layer was extracted with CH_2Cl_2 (2 × 10 mL) and the combined organic layers were washed with brine, dried over Na_2SO_4 and concentrated *in vacuo*. Purification *via* flash column chromatography (eluent Pentane/CH_2Cl_2, 20:80→ Pentane: Et_2O, 90:10) gave the *title compound* as an off-white solid (101 mg, 35%).

m.p. = 84 – 86 °C;

^{1}H NMR (400 MHz, $CDCl_3$) δ 7.40 – 7.28 (m, 5H), 6.93 (s, 1H), 6.81 (s, 4H), 4.20 (dd, *J* = 9.2, 5.2 Hz, 1H), 4.08 (dd, *J* = 9.2, 6.7 Hz, 1H), 3.87 (q, *J* = 6.2 Hz, 1H), 3.76 (s, 3H), 3.52 – 3.37 (m, 1H), 3.18 – 3.00 (m, 1H), 2.16 (s, 6H), 1.90 (s, 6H);
^{13}C NMR (126 MHz, $CDCl_3$) δ 209.8, 154.1, 153.0, 142.5, 142.1, 134.5, 131.7, 128.6, 128.4, 128.2, 127.0, 115.7, 114.8, 71.7, 55.9, 47.9, 39.9, 19.6, 15.8;
IR (film) ν_{max}/cm^{-1} 2938, 1701, 1508, 1289, 1230;
m/z (ESI$^+$) Found [M+H]$^+$ = 403, $C_{27}H_{31}O_3$ requires 403;
HRMS (ESI$^+$) m/z: [M+H]$^+$ Calcd for $C_{27}H_{31}O_3^+$ 403.2266; found 403.2268, Δ 0.37 ppm.

10 (2-Phenylcyclopropyl)(2,3,5,6-tetramethylphenyl)methanone

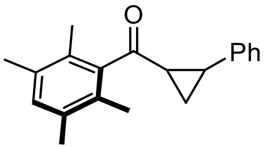

Ketone **9** (75 mg, 0.19 mmol), $[Ir(cod)Cl]_2$ (1.3 mg, 1.0 mol%), dppBz (1.8 mg, 2.0 mol%), KOH (45 mg, 0.8 mmol) and cyclopropanemethanol (0.17 mL, 2.0 mmol) were added to a 2-5 mL microwave vial. The vial was sealed and evacuated and backfilled with Ar (three times). The reaction was heated at 90 °C for 20 h, cooled to room temperature, filtered through a silica plug (elution with Et_2O) and concentrated *in vacuo*. Purification *via* flash column chromatography (eluent Pentane/Et_2O, 99.5:0.5 → 99:1) gave the *title compound* as an off-white solid (38 mg, 73%).
m.p. = 108 – 110 °C;
^{1}H NMR (400 MHz, $CDCl_3$) δ 7.34 – 7.24 (m, 2H), 7.24 – 7.15 (m, 1H), 7.14 – 7.06 (m, 2H), 6.95 (s, 1H), 2.77 (ddd, *J* = 9.1, 6.7, 4.0 Hz, 1H), 2.42 (ddd, *J* = 8.0, 5.3, 4.0 Hz, 1H), 2.20 (s, 6H), 2.13 (s, 6H), 1.94 (ddd, *J* = 9.3, 5.3, 4.1 Hz, 1H), 1.63 – 1.52 (m, 1H);
^{13}C NMR (101 MHz, $CDCl_3$) δ 209.7, 143.3, 140.1, 134.5, 131.8, 128.7, 128.5, 126.8, 126.2, 35.3, 30.9, 20.0, 19.6, 16.4.
IR (film) ν_{max}/cm^{-1} 2922, 1686, 1470, 1456, 1398, 980, 669;
m/z (ESI$^+$) Found [M+H]$^+$ = 279, $C_{20}H_{23}O$ requires 279;
HRMS (ESI$^+$) m/z: [M+H]$^+$ Calcd for $C_{20}H_{23}O^+$ 279.1743; found 279.1743, Δ 0.20 ppm.

S6 5-Oxo-5-(2,3,4,5,6-pentamethylphenyl)pentanoic acid

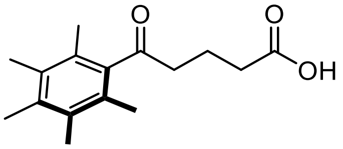

$AlCl_3$ (5.12 g, 39.0 mmol) was added portion-wise (over 5 min) to a stirred solution of glutaric anhydride (2.00 g, 18.0 mmol) and pentamethylbenzene (2.85 g, 19.0 mmol) in CH_2Cl_2 (70 mL) at RT. The reaction was stirred for 5 minutes at RT and was then poured into cold 1 M HCl (100 mL). The layers were separated and the aqueous phase extracted with CH_2Cl_2 (2 × 100 mL). The combined organic layers were washed with 1 M HCl (2 × 100 mL), dried over $MgSO_4$ and concentrated *in vacuo*. The residue was dissolved in MeCN (1 L) and washed with pentane (4 × 300 mL). The MeCN layer was concentrated *in vacuo* to give the *title compound* as a white solid which was used without further purification (3.31 g, 47%);

m.p. = 142 – 145 °C;

^{1}H NMR (400 MHz, $CDCl_3$) δ 2.76 (t, *J* = 7.1 Hz, 2H), 2.52 (t, *J* = 7.3 Hz, 2H), 2.23 (s, 3H), 2.18 (s, 6H), 2.0 (d, *J* = 10.9 Hz, 8H);

^{13}C NMR (101 MHz, $CDCl_3$) δ 211.2, 178.7, 140.5, 135.7, 133.3, 127.4, 44.3, 33.0, 18.4, 17.3, 16.8, 16.1;

IR (film) ν_{max}/cm^{-1} 3029, 2975, 1694, 1405, 1310, 1233, 1199, 1118, 912, 735;

m/z (ESI$^-$) Found [M-H]$^-$ = 261, $C_{16}H_{21}O_3$ requires 261;

HRMS (ESI$^-$) m/z: [M-H]$^-$ Calcd for $C_{16}H_{21}O_3^-$ 261.1496; found 261.1493, Δ 1.15 ppm

S7 5-Hydroxy-1-(2,3,4,5,6-pentamethylphenyl)pentan-1-one

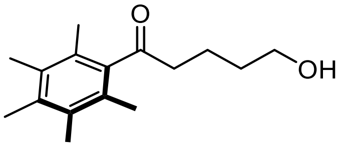

$BH_3{\cdot}SMe_2$ (1.56 mL, 16.5 mmol) was added dropwise to a solution of **S6** (2.88 g, 11.0 mmol) in THF (55 mL) at 0 °C. The mixture was allowed to warm to RT slowly over 16 h. The reaction was cooled to 0 °C, and quenched with MeOH (8 mL) and H_2O (26 mL). The mixture was extracted with EtOAc (3 × 100 mL) and the combined organics dried ($MgSO_4$) and concentrated *in vacuo*. Purification *via* flash column chromatography (eluent Pentane/Et_2O, 60:40) gave the *title compound* as a white solid (2.52 g, 92%).

m.p. = 76 – 77 °C.

^{1}H NMR (400 MHz, $CDCl_3$) δ 3.69 (m, 2H), 2.72 (t, *J* = 7.1 Hz, 2H), 2.23 (s, 3H), 2.18 (s, 6H), 2.09 (s, 6H), 1.85–1.76 (m, 2H), 1.71–1.56 (m, 3H);

^{13}C NMR (101 MHz, $CDCl_3$) δ 212.2, 140.7, 135.6, 133.2, 127.4, 62.7, 45.3, 32.3, 19.5, 17.3, 16.8, 16.1;

IR (film) ν_{max}/cm^{-1} 3265, 2921, 2868, 1696, 1460, 1406, 1120, 1067, 938, 846, 694;

HRMS (ESI$^+$) m/z: $[M+H]^+$ Calcd for $C_{16}H_{25}O_2^+$ 249.1849; found 249.1851, Δ 0.79 ppm.

11 1-(2,3,4,5,6-Pentamethylphenyl)-5-phenoxypentan-1-one

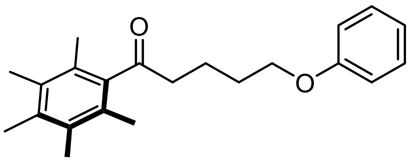

Phenol (458 mg, 4.87 mmol), PPh_3 (1.34 g, 5.12 mmol) and di-*tert*-butyl azodicarboxylate (1.12 g, 4.87 mmol) were added sequentially to a solution of alcohol **S7** (1.00 g, 4.03 mmol) in THF (10.3 mL) at RT. The mixture was stirred at RT for 30 min and then concentrated *in vacuo*. Purification *via* column chromatography (eluent Pentane/Et_2O, 97:3 → 95:5) gave the *title compound* as a white solid (206 mg, 16%).

m.p. = 130 – 132 °C.

^{1}H NMR (400 MHz, $CDCl_3$) δ 7.31–7.26 (m, 2H), 6.96–6.91 (m, 1H), 6.91–6.87 (m, 2H), 4.00 (t, *J* = 5.8 Hz, 2H), 2.76 (t, *J* = 7.0 Hz, 2H), 2.23 (s, 3H), 2.18 (s, 6H), 2.10 (s, 6H), 1.97–1.84 (m, 4H);

^{13}C NMR (101 MHz, $CDCl_3$) δ 211.8, 159.1, 140.7, 135.5, 133.2, 129.6, 127.4, 120.7, 114.6, 67.7, 45.3, 28.9, 20.1, 17.3, 16.8, 16.1;

IR (film) ν_{max}/cm^{-1} 2933, 2875, 1689, 1602, 1584, 1500, 1475, 1265, 1176, 1013, 991, 971, 747;

HRMS (ESI$^+$) m/z: $[M+Na]^+$ Calcd for $C_{22}H_{28}O_2Na^+$ 347.1981; found 347.1981, Δ 0.09 ppm.

S8 1-(2,3,4,5,6-Pentamethylphenyl)-2-(3-phenoxypropyl)hexan-1-one

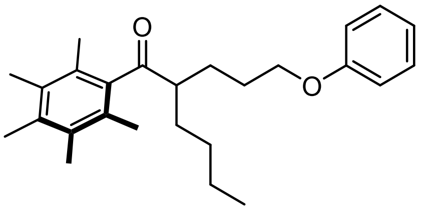

Ketone **11** (97 mg, 0.30 mmol), $[Ir(cod)Cl]_2$ (2.0 mg, 1.0 mol%), dppBz (3.0 mg, 2.0 mol%), KOH (67 mg, 1.2 mmol) and *n*-BuOH (0.27 mL, 3.00 mmol) were subjected to **General Procedure 1** at 85 °C. Purification *via* column chromatography (eluent, Pentane/Et_2O, 98:2 → 95:5) gave the *title compound* as a white solid (93 mg, 81%).

m.p. = 78 – 80 °C;

^{1}H NMR (400 MHz, $CDCl_3$,) δ 7.32–7.25 (m, 2H), 6.98–6.92 (m, 1H), 6.91–6.86 (m, 2H), 4.03–3.91 (m, 2H), 2.88–2.79 (m, 1H), 2.25 (s, 3H), 2.20 (s, 6H), 2.12 (s, 6H), 1.94–1.83 (m, 3H), 1.82–1.64 (m, 2H), 1.56–1.44 (m, 1H), 1.44–1.24 (m, 4H), 0.90 (t, *J* = 7.0 Hz, 3H);

^{13}C NMR (101 MHz, $CDCl_3$) δ 213.9, 159.1, 139.9, 135.6, 133.2, 129.5, 128.6, 120.7, 114.5, 67.9, 53.1, 29.5, 29.2, 27.0, 25.6, 23.1, 18.0, 16.9, 16.2, 14.1;

IR (film) ν_{max}/cm^{-1} 2950, 2930, 2867, 1689, 1601, 1585, 1498, 1248, 1035, 748, 690;

HRMS (ESI^+) m/z: $[M+H]^+$ Calcd for $C_{26}H_{37}O_2^+$ 381.2788; found 381.2788, Δ 0.16 ppm.

S9 Methyl 6-oxo-6-(2,3,4,5,6-pentamethylphenyl)hexanoate

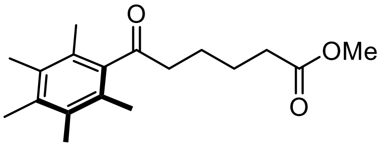

Pentamethylbenzene (1.57 g, 11.0 mmol) and methyl adipoyl chloride (1.2 mL, 7.7 mmol) were dissolved in CH_2Cl_2 (30 mL) and the mixture was cooled to 0 °C. $AlCl_3$ (1.92 g, 14.0 mmol) was added portion-wise and the reaction was allowed to warm to RT and stirred for 15 min. The crude reaction mixture was poured onto ice (approx. 50 g) and the layers were separated. The aqueous layer was extracted with CH_2Cl_2 (2 × 50 mL) and the combined organic layers were washed sequentially with satd. aq. $NaHCO_3$ and brine, dried over Na_2SO_4 and concentrated *in vacuo*. Purification via flash column chromatography (eluent Pentane: Et_2O, 95:5 → 85:15) gave the *title compound* as a white solid (1.74 g, 78%).

m.p. = 54 – 56 °C;

^{1}H NMR (400 MHz, $CDCl_3$) δ 3.68 (s, 3H), 2.76 – 2.64 (m, 2H), 2.41 – 2.29 (m, 2H), 2.23 (s, 3H), 2.18 (s, 6H), 2.09 (s, 6H), 1.84 – 1.64 (m, 4H);

^{13}C NMR (101 MHz, $CDCl_3$) δ 211.6, 174.0, 140.7, 135.5, 133.2, 127.4, 51.7, 45.3, 34.1, 24.6, 22.9, 17.3, 16.8, 16.1.

IR (neat) ν_{max}/cm^{-1} 2952, 1729, 1683, 1465, 1235, 1168;

m/z (ESI$^+$) Found $[M+H]^+$ = 291, $C_{18}H_{27}O_3$ requires 291;

HRMS (ESI$^+$) m/z: $[M+H]^+$ Calcd for $C_{18}H_{27}O_3^+$ 291.1955; found 291.1955, Δ 0.08 ppm.

S10 6-Hydroxy-1-(2,3,4,5,6-pentamethylphenyl)hexan-1-one

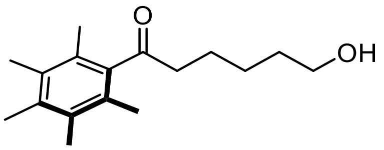

A solution of ester **S9** (1.50 g, 5.20 mmol) in Et_2O (10 mL) was added dropwise to a stirred suspension of $LiAlH_4$ (196 mg, 5.20 mmol) in Et_2O (25 mL) dropwise at –78 °C. The resulting mixture was stirred at –78 °C for 2.5 h and then at 0 °C for 10 min. The reaction was quenched with sequential dropwise addition of water (0.2 mL), aq. NaOH (15% w/v, 0.2 mL) and water (0.6 mL) at 0 °C. The resulting mixture was diluted with Et_2O and $MgSO_4$ was added. The mixture was vigorously stirred for 15 min, then filtered and concentrated *in vacuo*. Purification via flash column chromatography (eluent Pentane: Et_2O, 55:45 → 45:55) gave the *title compound* as a white solid (1.11 g, 82%).

m.p. = 74 – 75 °C;

^{1}H NMR (400 MHz, $CDCl_3$) δ 3.68 (td, *J* = 6.5, 5.3 Hz, 2H), 2.69 (t, *J* = 7.3 Hz, 2H), 2.23 (s, 3H), 2.18 (s, 6H), 2.09 (s, 6H), 1.75 (dt, *J* = 15.1, 7.3 Hz, 2H), 1.68 – 1.59 (m, 2H), 1.55 – 1.40 (m, 2H), 1.32 (t, *J* = 5.4 Hz, 1H);

^{13}C NMR (101 MHz, $CDCl_3$) δ 212.1, 140.9, 135.5, 133.2, 127.4, 62.9, 45.7, 32.7, 25.5, 23.1, 17.3, 16.8, 16.1;

IR (film) ν_{max}/cm^{-1} 3394, 2935, 2864, 1698, 1122, 1071, 1053;

m/z (ESI$^+$) Found $[M+H]^+$ = 263, $C_{17}H_{27}O_2$ requires 263;

HRMS (ESI$^+$) m/z: $[M+H]^+$ Calcd for $C_{17}H_{27}O_2^+$ 263.2006; found 263.1993; Δ 4.9 ppm.

12 1-(2,3,4,5,6-Pentamethylphenyl)-6-phenoxyhexan-1-one

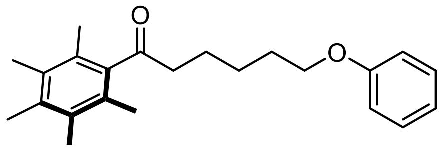

Phenol (343 mg, 3.64 mmol), PPh_3 (1.00 g, 3.82 mmol) and di-*tert*-butyl azodicarboxylate (838 mg, 3.64 mmol) were added sequentially to a solution of **S10** (789 mg, 3.01 mmol) in THF (7.7 mL) at RT. The mixture was stirred at RT for 1 h 30 min and then concentrated *in vacuo*. Purification *via* column chromatography (eluent Pentane/Et_2O, 97:3→95:5) gave the *title compound* as a white solid (400 mg, 40%).

m.p. = 56 – 58 °C;

^{1}H NMR (400 MHz, $CDCl_3$) δ 7.31–7.24 (m, 2H), 6.96–6.87 (m, 3H), 3.98 (t, *J* = 6.4 Hz, 2H), 2.71 (t, *J* = 7.3 Hz, 2H), 2.23 (s, 3H), 2.18 (s, 6H), 2.10 (s, 6H), 1.88–1.75 (m, 4H), 1.61–1.51 (m, 2H).

^{13}C NMR (101 MHz, $CDCl_3$,) δ 212.0, 159.2, 140.9, 135.5, 133.2, 129.6, 127.4, 120.7, 114.6, 67.7, 45.7, 29.4, 25.9, 23.2, 17.3, 16.8, 16.1;

IR (film) ν_{max}/cm^{-1} 2928, 2866, 1697, 1603, 1587, 1502, 1469, 1252, 1069, 758, 694;

HRMS (ESI^+) m/z: $[M+H]^+$ Calcd for $C_{23}H_{31}O_2$ 339.2319; found 339.2320, Δ 0.32 ppm.

S11 2-Butyl-1-(2,3,4,5,6-pentamethylphenyl)-6-phenoxyhexan-1-one

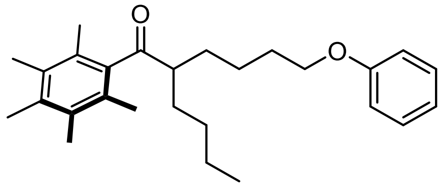

Ketone **12** (102 mg, 0.300 mmol), $[Ir(cod)Cl]_2$ (2.0 mg, 1.0 mol%), dppBz (3.0 mg, 2.0 mol%), KOH (67 mg, 1.20 mmol) and *n*-BuOH (0.27 mL, 3.00 mmol) were subjected to **General Procedure 1** at 85 °C. Purification *via* column chromatography (eluent, Pentane/Et_2O, 98:2 → 95:5) gave the *title compound* as a white solid (115 mg, 97%).

m.p. = 69 – 70 °C;

^{1}H NMR (400 MHz, $CDCl_3$) δ 7.32–7.23 (m, 2H), 6.97–6.91 (m, 1H), 6.90–6.83 (m, 2H), 3.94 (t, *J* = 6.5 Hz, 2H), 2.81–2.72 (m, 1H), 2.24 (s, 3H), 2.18 (s, 6H), 2.10 (s, 6H), 1.86–1.67 (m, 4H), 1.60–1.41 (m, 4H), 1.39–1.24 (m, 4H), 0.88 (t, *J* = 7.1 Hz, 3H);

^{13}C NMR (101 MHz, $CDCl_3$) δ 214.0, 159.2, 140.1, 135.6, 133.2, 129.5, 128.6, 120.7, 114.6, 67.7, 53.5, 29.7, 29.6, 29.1, 29.1, 24.0, 23.1, 18.0, 16.9, 16.2, 14.1;

IR (film) ν_{max}/cm^{-1} 2932, 2869, 1690, 1600, 1496, 1469, 1300, 1243, 752, 691;

HRMS (ESI$^+$) m/z: [M+H]$^+$ Calcd for $C_{27}H_{39}O_2$ 395.2945; found 395.2945, Δ 0.12 ppm.

S12 6-Bromo-1-(2,3,4,5,6-pentamethylphenyl)hexan-1-one

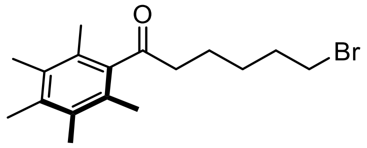

A solution of pentamethylbenzene (3.00 g, 20.0 mmol), and 6-hexanoyl chloride (3.4 mL, 22 mmol) in CH_2Cl_2 (100 mL) was cooled in an ice bath, $AlCl_3$ (3.37 g, 25.0 mmol) was added portion-wise over 10 mins. The mixture was allowed to warm to RT and stirred for 30 min, then poured onto ice (approx. 100 g) and stirred rapidly until the ice melted. The organic phase was separated and the aqueous phase extracted with CH_2Cl_2 (2 × 100 mL). The combined organic phases were washed with satd. aq. $NaHCO_3$ and concentrated *in vacuo*. Purification *via* flash column chromatography (eluent Pentane/Et_2O, 97:3 → 95:5) gave the *title compound* as a white solid (5.65 g, 84%).

m.p. = 46 – 47 °C;

^{1}H NMR (400 MHz, $CDCl_3$) δ 3.43 (t, *J* = 6.8 Hz, 2H), 2.69 (t, *J* = 7.3 Hz, 2H), 2.23 (s, 3H), 2.19 (s, 6H), 2.10 (s, 6H), 1.91 (dq, *J* = 8.1, 6.8 Hz, 2H), 1.83 – 1.67 (m, 2H), 1.60 – 1.43 (m, 2H);

^{13}C NMR (101 MHz, $CDCl_3$) δ 211.8, 140.8, 135.5, 133.2, 127.4, 45.5, 33.7, 32.9, 27.9, 22.6, 17.3, 16.8, 16.1;

IR (film) ν_{max}/cm^{-1} 2936, 1699, 1454, 1262, 1242, 1219, 1111;

m/z (ESI$^+$) Found [M(^{81}Br)+Na]$^+$ = 327 (47%), $C_{17}H_{26}{}^{81}BrO$ requires 327, [M(^{79}Br)+H]$^+$ = 325 (53%), $C_{17}H_{26}{}^{79}BrO$ requires 325.

HRMS (ESI$^+$) m/z: [M+H]$^+$ Calcd for $C_{17}H_{26}{}^{79}BrO^+$ 325.1162; found 325.1174, Δ 3.83 ppm.

13 1-(2,3,4,5,6-Pentamethylphenyl)-6-(phenylthio)hexan-1-one

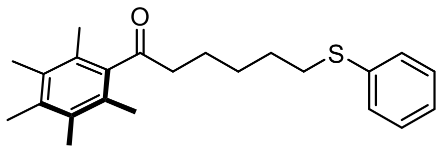

Triethylamine (2.8 mL, 20 mmol) was added dropwise to a solution of **S12** (3.24 g, 10.0 mmol) and thiophenol (1.3 mL, 12 mmol) in EtOAc (100 mL). The reaction mixture was stirred at 65 °C

for 18 h, cooled to RT and concentrated *in vacuo.* Purification *via* flash column chromatography (eluent Pentane/Et_2O, 98:2 → 95:5) gave the *title compound* as a white solid (3.51 g, 99%).

m.p. 48 – 50 °C;

^{1}H NMR (400 MHz, $CDCl_3$) δ 7.27 – 7.23 (m, 2H), 7.22 – 7.15 (m, 2H), 7.11 – 7.06 (m, 1H), 2.91 – 2.79 (m, 2H), 2.59 (t, *J* = 7.3 Hz, 2H), 2.15 (s, 3H), 2.10 (s, 6H), 2.02 (s, 6H), 1.74 – 1.57 (m, 4H), 1.45 (m, 2H);

^{13}C NMR (101 MHz, $CDCl_3$) δ 211.9, 140.8, 136.9, 135.5, 133.2, 129.2, 129.0, 127.4, 125.9, 45.5, 33.6, 29.3, 28.5, 22.9, 17.3, 16.8, 16.1;

IR (film) ν_{max}/cm^{-1} 2931, 1699, 1584, 1480, 1439, 1402, 1382, 1303, 1265, 1141, 1025, 998, 929, 738, 691;

m/z (ESI$^+$) Found [M+Na]$^+$ = 377 $C_{23}H_{30}NaOS$ requires 377;

HRMS (ESI$^+$) $C_{23}H_{30}NaOS^+$ ([M+Na]$^+$) requires 377.1910; found 377.1913, Δ 0.80 ppm.

S13 2-Benzyl-1-(2,3,4,5,6-pentamethylphenyl)-6-(phenylthio)hexan-1-one

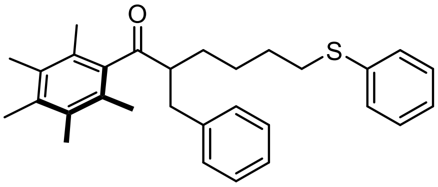

Ketone **13** (106 mg, 0.30 mmol), $[Ir(cod)Cl]_2$ (2.0 mg, 1.0 mol%), dppBz (3.0 mg, 2.0 mol%), KOH (67 mg, 1.2 mmol) and Benzyl alcohol (0.31 mL, 3.0 mmol) were subjected to **General Procedure 1** at 115 °C. Purification *via* flash column chromatography (eluent Pentane/Et_2O, 98:2 → 97.5:2.5) gave the *title compound* as a (viscous) colourless oil (109 mg, 84%);

^{1}H NMR (400 MHz, $CDCl_3$) δ 7.26 – 7.20 (m, 5H), 7.20 – 7.11 (m, 5H), 3.15 (dd, *J* = 13.5, 6.0 Hz, 1H), 3.03 (dq, *J* = 7.9, 5.9 Hz, 1H), 2.79 (t, *J* = 7.1 Hz, 2H), 2.64 (dd, *J* = 13.5, 7.9 Hz, 1H), 2.21 (s, 3H), 2.15 (s, 6H), 2.00 – 1.90 (br s, 6H), 1.69 (m, 2H), 1.61 – 1.32 (m, 4H);

^{13}C NMR (101 MHz, $CDCl_3$) δ 212.8, 140.3, 139.6, 136.9, 135.8, 133.3, 129.4, 129.1, 129.0, 128.7, 128.4, 126.2, 125.9, 55.8, 35.7, 33.4, 29.4, 29.1, 26.5, 17.9, 16.9, 16.2;

IR (film) ν_{max}/cm^{-1} 2981, 1691, 1382, 1302, 1259, 1154, 955, 819, 738, 700;

m/z (ESI$^+$) Found [M+Na]$^+$ = 467 $C_{30}H_{36}NaOS$ requires 467;

HRMS (ESI$^+$) m/z: [M+Na]$^+$ Calcd for $C_{30}H_{36}ONaS^+$ 467.2379; found 467.2379, Δ 0.05 ppm.

Cyclopropane Surrogate Method

S14 3-Phenyl-1-(2,3,5,6-tetramethylphenyl)propan-1-one

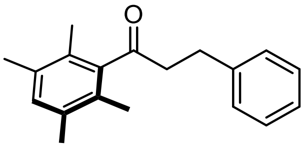

3-Phenylpropanoic acid (4.50 g, 30 mmol) was dissolved in thionyl chloride (5.3 mL, 75 mmol) and the mixture was refluxed for 16 h. Once cooled to RT, the reaction mixture was concentrated *in vacuo*. The crude acyl chloride was dissolved in CH_2Cl_2 (10 mL) and added to a solution of durene (4.40 g, 32.8 mmol) in CH_2Cl_2 (90 mL) at 0 °C. $AlCl_3$ (5.00 g, 37.5 mmol) was added portion-wise and the mixture was stirred at RT for 1 h. The crude reaction mixture was poured onto ice (approx. 150 g) and the layers were separated. The aqueous layer was extracted with CH_2Cl_2 (2 × 100 mL) and the combined organic layers were washed sequentially with satd. aq. $NaHCO_3$ and brine, dried over Na_2SO_4 and concentrated *in vacuo*. Purification via flash column chromatography (eluent Pentane/Et_2O, 98:2) gave the title compound as an off-white solid (1.30 g, 16%).

^{1}H NMR (400 MHz, $CDCl_3$) δ 7.33 – 7.17 (m, 5H), 6.95 (s, 1H), 3.07 (ddd, *J* = 7.7, 6.4, 1.9 Hz, 2H), 3.04 – 2.94 (m, 2H), 2.19 (s, 6H), 2.00 (s, 6H);

^{13}C NMR (101 MHz, $CDCl_3$) δ 210.8, 142.7, 141.1, 134.5, 131.7, 128.6, 128.6, 128.1, 126.2, 46.9, 29.4, 19.6, 16.0;

m/z (ESI$^+$) Found $[M+Na]^+$ = 289, $C_{19}H_{22}NaO$ requires 289.

The spectral data matched that previously reported in the literature.[9]

8a (1-Benzylcyclopropyl)(2,3,5,6-tetramethylphenyl)methanone

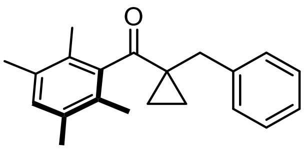

Ir-catalysed: Ketone **S15** (80 mg, 0.30 mmol), $[Ir(cod)Cl]_2$ (4.0 mg, 1.0 mol%), dppBz (6.0 mg, 2.0 mol%), KOH (67 mg, 1.2 mmol) and 2-phenoxyethanol (0.37 mL, 3.0 mmol) were subjected to **General Procedure 3**. Purification *via* flash column chromatography (eluent Pentane/Et_2O, 98.5:1.5) gave the *title compound* as a colourless oil (54 mg, 61%).

Ru-catalysed: Ketone **S15** (80 mg, 0.30 mmol), Ru-MACHO® (3.6 mg, 2.0 mol%) KOH (67 mg, 1.2 mmol) and 2-phenoxyethanol (0.37 mL, 3.0 mmol) were subjected to **General Procedure 3**. Purification *via* flash column chromatography (eluent Pentane/Et_2O, 98.5:1.5) gave the *title compound* as a colourless oil (48 mg, 54%).

The spectral data matched that reported for the prefunctionalisation method.

S15 5-Methyl-1-(2,3,5,6-tetramethylphenyl)hexan-1-one

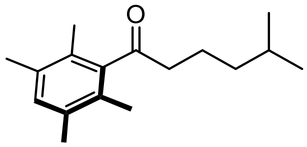

5-Methylhexanoic acid (2.2 mL, 15 mmol) was dissolved in thionyl chloride (2.3 mL, 31 mmol) and the mixture was refluxed for 3 h. Once cooled to RT, the reaction mixture was concentrated *in vacuo*. The crude acyl chloride was dissolved in CH_2Cl_2 (10 mL) and added to a solution of durene (3.10 g, 23.1 mmol) in CH_2Cl_2 (50 mL) at 0 °C. $AlCl_3$ (3.28 g, 24.6 mmol) was added portion-wise and the mixture was stirred at RT for 1 h. The crude reaction mixture was poured onto ice (approx. 50 g) and the layers were separated. The aqueous layer was extracted with CH_2Cl_2 (2 × 50 mL) and the combined organic layers were washed sequentially with satd. aq. $NaHCO_3$ and brine, dried over Na_2SO_4 and concentrated *in vacuo*. Purification *via* flash column chromatography (eluent Pentane: Et_2O, 99:1) gave the *title compound* as a white solid (1.31 g, 35%).

m.p. = 43 – 46 °C;

^{1}H NMR (400 MHz, $CDCl_3$) δ 6.95 (s, 1H), 2.75 – 2.55 (m, 2H), 2.20 (s, 6H), 2.06 (s, 6H), 1.83 – 1.64 (m, 2H), 1.65 – 1.50 (m, 1H), 1.36 – 1.16 (m, 2H), 0.91 (d, *J* = 6.6 Hz, 6H);

^{13}C NMR (101 MHz, $CDCl_3$) δ 212.1, 143.1, 134.5, 131.6, 128.0, 45.7, 38.5, 28.1, 22.7, 21.2, 19.6, 16.1;

IR (film) ν_{max}/cm^{-1} 2955, 2871, 1704, 1544, 1469, 1385;

m/z (ESI$^+$) Found ($[M+H]^+$) = 247, $C_{17}H_{27}O$ requires 247;

HRMS (ESI$^+$) m/z: $[M+H]^+$ Calcd for $C_{17}H_{27}O^+$ 247.2056; found 247.2057, Δ 0.22 ppm.

8f (1-Isopentylcyclopropyl)(2,3,5,6-tetramethylphenyl)methanone

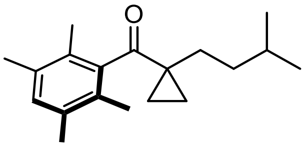

Ir-catalysed: Ketone **S15** (74 mg, 0.30 mmol), $[Ir(cod)Cl]_2$ (2.0 mg, 1.0 mol%), dppBz (3.0 mg, 2.0 mol%), KOH (67 mg, 1.2 mmol) and 2-phenoxyethanol (0.37 mL, 3.0 mmol) were subjected to **General Procedure 3**. Purification *via* flash column chromatography (eluent Pentane/Et_2O, 98.5:1.5) gave the *title compound* as a colourless oil which solidified on standing (45 mg, 55%).

Ru-catalysed: Ketone **S15** (74 mg, 0.30 mmol), Ru-MACHO® (3.6 mg, 2.0 mol%), KOH (67 mg, 1.2 mmol) and 2-phenoxyethanol (0.37 mL, 3.0 mmol) were subjected to **General Procedure 3**. Purification *via* flash column chromatography (eluent Pentane/Et_2O, 98.5:1.5) gave the *title compound* as a colourless oil which solidified on standing (48 mg, 59%).

The spectral data matched that reported for the prefunctionalisation method.

S16 2-Phenyl-1-(2,3,5,6-tetramethylphenyl)ethan-1-one

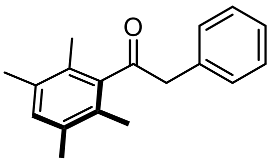

Durene (2.50 g, 18.6 mmol) and phenylacetylchloride (2.7 mL, 20.5 mmol) were dissolved in CH_2Cl_2 (65 mL) and the mixture was cooled to 0 °C. $AlCl_3$ (3.11 g, 23.2 mmol) was added portion-wise over 15 min, the reaction warmed to RT and stirred for 30 min. The crude reaction mixture was poured onto ice (approx. 75 g) and the layers were separated. The aqueous layer was extracted with CH_2Cl_2 (2 × 75 mL) and the combined organic layers were washed with satd. aq. $NaHCO_3$, dried over Na_2SO_4 and concentrated *in vacuo*. Purification *via* recrystallisation from a minimum amount of boiling hexane gave the *title compound* as a white solid (3.70 g, 79%).

m.p. = 104 – 106 °C;

^{1}H NMR (400 MHz, $CDCl_3$) δ 7.31 – 7.13 (m, 5H), 6.91 (s, 1H), 3.92 (s, 2H), 2.14 (s, 6H), 1.96 (s, 6H);

^{13}**C NMR** (101 MHz, $CDCl_3$) δ 208.7, 142.4, 134.5, 133.3, 131.9, 130.1, 128.7, 128.3, 127.2, 52.3, 19.6, 16.2;

IR (film) ν_{max}/cm^{-1} 2968, 1703, 1320, 1104, 979, 710;

m/z (ESI$^+$) Found $[M+Na]^+$ = 275, $C_{18}H_{20}NaO$ requires 275;

HRMS (ESI$^+$) m/z: $[M+H]^+$ Calcd for $C_{18}H_{21}O^+$ 253.1587; found 253.1575 Δ 4.72 ppm.

8k (1-Phenylcyclopropyl)(2,3,5,6-tetramethylphenyl)methanone

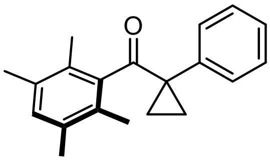

Ketone **S16** (76 mg, 0.30 mmol), $[Ir(cod)Cl]_2$ (2.0 mg, 1.0 mol%), dppBz (3.0 mg, 2.0 mol%), KOH (67 mg, 1.2 mmol) and 2-phenoxyethanol (0.37 mL, 3.0 mmol) were subjected to **General Procedure 3**. Purification *via* flash column chromatography (eluent Pentane/Et_2O, 99.5:0.5 → 98:2) gave the *title compound* as a colourless oil (27 mg, 33%).

m.p. = 94 – 96 °C;

^{1}**H NMR** (400 MHz, $CDCl_3$) δ 7.33 – 7.27 (m, 2H), 7.24 – 7.14 (m, 3H), 6.81 (s, 1H), 2.12 (s, 6H), 2.11 (s, 6H), 1.68 (q, *J* = 3.7 Hz, 2H), 1.47 – 1.35 (m, 2H);

^{13}**C NMR** (101 MHz, $CDCl_3$) δ 211.6, 140.3, 139.9, 134.1, 131.4, 130.7, 128.5, 128.0, 127.3, 39.7, 19.5, 19.5, 17.1;

IR (film) ν_{max}/cm^{-1} 2939, 1676, 1306, 1257, 975, 701;

m/z (ESI$^+$) Found $[M+H]^+$ = 279, $C_{20}H_{23}O$ requires 279;

HRMS (ESI$^+$) m/z: $[M+Na]^+$ Calcd for $C_{20}H_{22}NaO^+$ 301.1563; found 301.1570 Δ 2.36 ppm.

8k′ 4-Phenyl-5-(2,3,5,6-tetramethylphenyl)-2,3-dihydrofuran

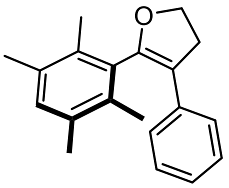

Ketone **S16** (76 mg, 0.30 mmol), $[Ir(cod)Cl]_2$ (2.0 mg, 1.0 mol%), dppBz (3.0 mg, 2.0 mol%), KOH (67 mg, 1.2 mmol) and 2-phenoxyethanol (0.37 mL, 3.0 mmol) were subjected to **General Procedure 3**. Purification *via* flash column chromatography (eluent Pentane/Et_2O, 99.5:0.5 → 98:2) gave the *title compound* as a colourless solid (20 mg, 24%, isolated in conjunction with 27 mg **8k**, 33%).

m.p. = 105 – 107 °C;

^{1}H NMR (400 MHz, $CDCl_3$) δ 7.13 (dd, *J* = 8.4, 7.0 Hz, 2H), 7.08 – 6.96 (m, 2H), 6.96 – 6.86 (m, 2H), 4.55 (t, *J* = 9.3 Hz, 2H), 3.25 (t, *J* = 9.3 Hz, 2H), 2.23 (s, 6H), 2.10 (s, 6H);

^{13}C NMR (101 MHz, $CDCl_3$) δ 152.3, 135.2, 134.1, 133.0, 132.3, 132.2, 128.3, 125.0, 124.7, 110.8, 68.7, 33.4, 20.1, 16.2;

IR (film) ν_{max}/cm^{-1} 2924, 1651, 1601, 1471, 1152, 1011, 760, 694;

HRMS (ESI$^+$) m/z: $[M+H]^+$ Calcd for $C_{20}H_{23}O^+$ 279.1743; found 279.1730 Δ 4.82 ppm.

S17 4-Oxo-4-(2,3,5,6-tetramethylphenyl)butanoic acid

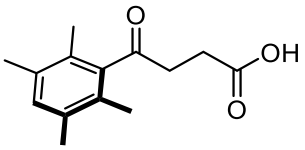

$AlCl_3$ (16.0 g, 120 mmol) was added portion-wise (over 5 min) to a stirred solution of succinic anhydride (6.00 g, 60 mmol) and 1,2,4,5-tetramethylbenzene (8.86 g, 66.0 mmol) in CH_2Cl_2 (200 mL) at RT. The reaction was stirred for 45 minutes at RT and was then poured onto ice (approx. 200 g) and diluted with 1 M HCl (100 mL). The layers were separated and the aqueous phase extracted with CH_2Cl_2 (2 × 300 mL). The combined organic layers were washed sequentially with 1 M HCl (200 mL) and brine, dried over $MgSO_4$ and concentrated *in vacuo*. Purification *via* flash column chromatography (eluent CH_2Cl_2 → CH_2Cl_2/MeOH, 90:10) gave the *title compound* as a white solid (12.2 g, 87%);

m.p. = 155 – 157 °C;

^{1}H NMR (400 MHz, $CDCl_3$) δ 6.97 (s, 1H), 2.94 (t, *J* = 6.4 Hz, 2H), 2.71 (t, *J* = 6.4 Hz, 2H), 2.13 (s, 6H), 2.00 (s, 6H);

^{13}C NMR (101 MHz, $CDCl_3$) δ 209.3, 178.6, 141.9, 134.6, 131.9, 128.3, 39.8, 27.6, 19.6, 16.0;

IR (film) ν_{max}/cm^{-1} 3046, 2941, 1716, 1699;

m/z (ESI^+) Found $[M+H]^+$ = 235, $C_{14}H_{19}O_3$ requires 235;

HRMS (ESI^+) m/z: $[M+Na]^+$ Calcd for $C_{14}H_{18}NaO_3^+$ 257.1148; found 257.1148, Δ 0.03 ppm.

S18 4-Hydroxy-1-(2,3,5,6-tetramethylphenyl)butan-1-one

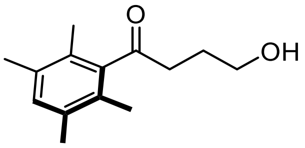

$BH_3{\cdot}SMe_2$ (3.6 mL, 37.5 mmol) was added dropwise (over 5 min) to a solution of acid **S17** (5.85 g, 25.0 mmol) in THF (125 mL) at 0 °C. The resulting solution was allowed to warm slowly to RT overnight (16 h), subsequently cooled to 0 °C and then quenched with MeOH (16 mL) and water (40 mL). The mixture was concentrated *in* vacuo, diluted further with water (200 mL) and extracted with EtOAc (3 × 200 mL). The combined organic layers were washed with brine, dried over $MgSO_4$ and concentrated *in vacuo*. Purification *via* flash column chromatography (eluent Pentane/Et_2O, 55:45 → Et_2O) gave the *title compound* as a white solid (5.09 g, 93%).

m.p. = 78 – 80 °C;

^{1}H NMR (400 MHz, $CDCl_3$) δ 6.95 (s, 1H), 3.75 (q, *J* = 5.8 Hz, 2H), 2.83 (t, *J* = 6.9 Hz, 2H), 2.20 (s, 6H), 2.06 (s, 6H), 2.04 – 1.94 (m, 2H), 1.83 (t, *J* = 5.1 Hz, 1H);

^{13}C NMR (101 MHz, $CDCl_3$) δ 212.3, 142.8, 134.5, 131.7, 128.0, 62.4, 42.2, 26.3, 19.6, 16.1;

IR (film) ν_{max}/cm^{-1} 3370, 2939, 1695, 1469, 1054, 1020;

m/z (ESI^+) Found $[M+H]^+$ = 221, $C_{14}H_{21}O_2$ requires 221;

HRMS (ESI^+) m/z: $[M+H]^+$ Calcd for $C_{14}H_{21}O_2^+$ 221.1536; found 221.1536, Δ 0.16 ppm

S19 4-((4-Methoxybenzyl)oxy)-1-(2,3,5,6-tetramethylphenyl)butan-1-one

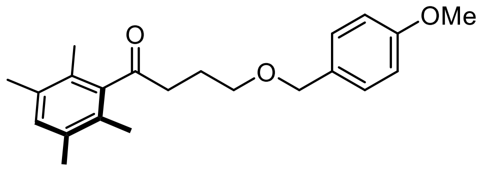

Alcohol **S18** (396 mg, 1.80 mmol), 4-methoxybenzyl chloride (0.50 mL, 3.6 mmol), *N,N*-diisopropylethylamine (0.66 mL, 3.8 mmol) and KI (60 mg, 0.36 mmol) were heated at 110 °C for 3 h. The mixture was cooled to RT and diluted with EtOAc (10 mL) and water (10 mL). The layers were separated and the aqueous layer was extracted with EtOAc (2 × 10 mL). The combined organic phases were washed with brine, dried over Na_2SO_4 and concentrated *in vacuo*. Purification *via* flash column chromatography (eluent Pentane/Et_2O, 93:7 → 92:8) gave the *title compound* as a white solid (511 mg, 83%).

m.p. = 48 – 50 °C;

^{1}H NMR (400 MHz, $CDCl_3$) δ 7.33 – 7.20 (m, 2H), 6.95 (s, 1H), 6.90 – 6.83 (m, 2H), 4.43 (s, 2H), 3.80 (s, 3H), 3.55 (t, *J* = 6.2 Hz, 2H), 2.80 (t, *J* = 7.2 Hz, 2H), 2.20 (s, 6H), 2.10 – 1.98 (obscured m, 8H (s, 6H and m, 2H overlapped);

^{13}C NMR (101 MHz, $CDCl_3$) δ 211.7, 159.3, 142.9, 134.5, 131.6, 130.6, 129.4, 128.0, 113.9, 72.7, 69.1, 55.4, 41.9, 23.5, 19.6, 16.1;

IR (film) ν_{max}/cm^{-1} 2937, 2862, 1699, 1613, 1514, 1248, 1098, 1036, 820;

m/z (ESI$^+$) Found [M+Na]$^+$ = 363, $C_{22}H_{28}NaO_3$ requires 363;

HRMS (ESI$^+$) m/z: [M+Na]$^+$ Calcd for $C_{22}H_{28}NaO_3^+$ 363.1931; found 363.1933, Δ 0.63 ppm.

8l (1-(2-((4-Methoxybenzyl)oxy)ethyl)cyclopropyl)(2,3,5,6-tetramethylphenyl)methanone

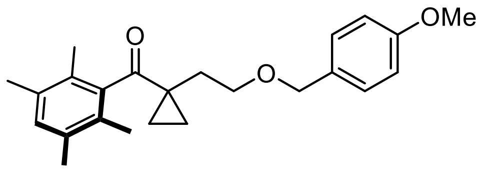

Ir-catalysed: Ketone **S19** (102 mg, 0.300 mmol), $[Ir(cod)Cl]_2$ (2.0 mg, 1.0 mol%), dppBz (3.0 mg, 2.0 mol%), KOH (67 mg, 1.2 mmol) and 2-phenoxyethanol (0.37 mL, 3.0 mmol) were subjected to **General Procedure 3**. Purification *via* flash column chromatography (eluent Pentane/Et_2O, 92:8) gave the *title compound* as an off-white solid (85 mg, 79%).

Ru-catalysed: Ketone **S19** (102 mg, 0.300 mmol), Ru-MACHO® (3.6 mg, 2.0 mol%), KOH (67 mg, 1.2 mmol) and 2-phenoxyethanol (0.37 mL, 3.0 mmol) were subjected to **General Procedure 3**. Purification *via* flash column chromatography (eluent Pentane/Et_2O, 92:8) gave the *title compound* as an off-white solid (72 mg, 66%).

m.p. = 81 – 83 °C;

^{1}H NMR (400 MHz, $CDCl_3$) δ 7.31 – 7.19 (m, 2H), 6.91 (s, 1H), 6.89 – 6.84 (m, 2H), 4.40 (s, 2H), 3.81 (s, 3H), 3.61 (t, *J* = 6.8 Hz, 2H), 2.16 (s, 6H), 2.03 (s, 6H), 1.99 (t, *J* = 6.8 Hz, 2H), 1.15 – 1.09 (m, 2H), 1.09 – 1.02 (m, 2H);

^{13}C NMR (101 MHz, $CDCl_3$) δ 212.4, 159.2, 138.9, 134.3, 131.6, 130.8, 129.3, 129.2, 113.9, 72.7, 68.6, 55.4, 33.4, 31.7, 19.6, 17.8, 16.6;

IR (film) ν_{max}/cm^{-1} 2938, 2863, 1669, 1514, 1249;

m/z (ESI$^+$) Found [M+Na]$^+$ = 389, $C_{24}H_{30}NaO_3$ requires 389;

HRMS (ESI$^+$) m/z: [M+Na]$^+$ Calcd for $C_{24}H_{30}NaO_3^+$ 389.2087; found 389.2088 Δ 0.20 ppm.

S20 4-Bromo-1-(2,3,5,6-tetramethylphenyl)butan-1-one

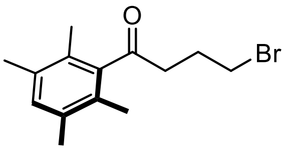

Durene (3.36 g, 25.0 mmol) and 4-bromobutyryl chloride (3.2 mL, 27.5 mmol) were dissolved in CH_2Cl_2 (85 mL) and the mixture was cooled to 0 °C. $AlCl_3$ (4.16 g, 31.3 mmol) was added portion-wise over 15 min, the reaction warmed to RT and stirred for 1 h. The crude reaction mixture was poured onto ice (approx. 100 g) and the layers were separated. The aqueous layer was extracted with CH_2Cl_2 (2 × 100 mL) and the combined organic layers were washed with satd. aq. $NaHCO_3$, dried over Na_2SO_4 and concentrated *in vacuo*. Purification *via* flash column chromatography (eluent Pentane/Et_2O, 98.5:1.5 → 97.5:2.5) gave the *title compound* as a white solid (6.64 g, 94%).

m.p. = 50 – 52 °C;

^{1}H NMR (400 MHz, $CDCl_3$) δ 6.96 (s, 1H), 3.58 (t, *J* = 6.3 Hz, 2H), 2.88 (t, *J* = 6.9 Hz, 2H), 2.29 (m, 2H), 2.21 (s, 6H), 2.05 (s, 6H);

^{13}C NMR (101 MHz, $CDCl_3$) δ 210.7, 142.5, 134.6, 131.8, 128.0, 43.2, 33.5, 26.3, 19.6, 16.1;

IR (film) ν_{max}/cm^{-1} 2922, 1703, 1471, 1106, 874;

m/z (ESI$^+$) Found [M+H]$^+$ = 283, $C_{14}H_{20}{}^{79}BrO$ requires 283;

HRMS (ESI$^+$) m/z: [M+H]$^+$ Calcd for $C_{14}H_{20}{}^{79}BrO^+$ 283.0692; found 283.0681 Δ 3.91 ppm.

S21 4-(Dibenzylamino)-1-(2,3,5,6-tetramethylphenyl)butan-1-one

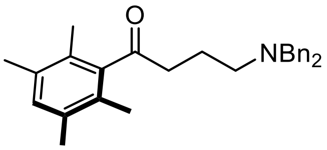

Bromide **S20** (1.42 g, 5.00 mmol), KI (83 mg, 0.50 mmol) and K_2CO_3 (1.38 g, 10.0 mmol) were added sequentially to a solution of Bn_2NH (1.0 mL, 5.25 mmol) in DMF (2.9 mL). The resulting mixture was heated to 85 °C for 18 h, cooled to RT and diluted with EtOAc (30 mL) and water (30 mL). The layers were separated and the aqueous layer was extracted with EtOAc (2 × 30 mL). The combined organic phases were washed with brine, dried over $MgSO_4$ and concentrated *in vacuo*. Purification *via* flash column chromatography (eluent Pentane/Et_2O, 98:2 → 94:6) gave the *title compound* as a white solid (1.49 g, 75%).

m.p. = 67 – 69 °C;

^{1}H NMR (400 MHz, $CDCl_3$) δ 7.38 – 7.26 (m, 8H), 7.26 – 7.18 (m, 2H), 6.95 (s, 1H), 3.58 (s, 4H), 2.69 (t, *J* = 7.5 Hz, 2H), 2.51 (t, *J* = 6.9 Hz, 2H), 2.20 (s, 6H), 2.00 (s, 6H), 1.95 (p, *J* = 7.2 Hz, 2H);

^{13}C NMR (101 MHz, $CDCl_3$) δ 212.0, 143.1, 139.8, 134.5, 131.6, 128.9, 128.3, 128.1, 127.0, 58.4, 52.8, 43.1, 20.8, 19.6, 16.1;

IR (film) ν_{max}/cm^{-1} 2941, 1699, 1453, 746, 699;

m/z (ESI$^+$) Found [M+H]$^+$ = 400, $C_{28}H_{34}NO$ requires 400;

HRMS (ESI$^+$) m/z: [M+Na]$^+$ Calcd for $C_{28}H_{33}NNaO^+$ 422.2454; found 422.2445 Δ 2.22 ppm.

8m (1-(2-(Dibenzylamino)ethyl)cyclopropyl)(2,3,5,6-tetramethylphenyl)methanone

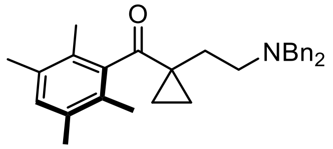

Ir-catalysed: Ketone **S21** (120 mg, 0.300 mmol), $[Ir(cod)Cl]_2$ (2.0 mg, 1.0 mol%), dppBz (3.0 mg, 2.0 mol%), KOH (67 mg, 1.2 mmol) and 2-phenoxyethanol (0.37 mL, 3.0 mmol) were subjected to **General Procedure 3**. Purification *via* flash column chromatography (eluent Pentane/Et_2O, 95:5) gave the *title compound* as a colourless oil which solidified on standing (105 mg, 82%).

Ru-catalysed: Ketone **S21** (120 mg, 0.300 mmol), Ru-MACHO® (3.6 mg, 2.0 mol%), KOH (67 mg, 1.2 mmol) and 2-phenoxyethanol (0.37 mL, 3.0 mmol) were subjected to **General Procedure 3**. Purification *via* flash column chromatography (eluent Pentane/Et_2O, 95:5) gave the *title compound* as a colourless oil which solidified on standing (102 mg, 80%).

m.p. = 81 – 83 °C;

^{1}H NMR (400 MHz, $CDCl_3$) δ 7.35 – 7.27 (m, 8H), 7.25 – 7.16 (m, 2H), 6.87 (s, 1H), 3.52 (s, 4H), 2.63 – 2.49 (m, 2H), 2.14 (s, 6H), 1.97 (s, 6H), 1.90 – 1.80 (m, 2H), 1.07 (q, *J* = 4.0 Hz, 2H), 0.86 (q, *J* = 4.1 Hz, 2H);

^{13}C NMR (101 MHz, $CDCl_3$) δ 212.4, 139.9, 134.2, 131.5, 129.1, 128.8, 128.3, 126.9, 58.3, 51.6, 32.5, 31.0, 19.6, 18.1, 16.6;

IR (film) ν_{max}/cm^{-1} 2925, 1671, 1495, 1454, 737, 699;

m/z (ESI$^+$) Found $[M+H]^+$ = 426, $C_{30}H_{36}NO$ requires 426;

HRMS (ESI$^+$) m/z: $[M+H]^+$ Calcd for $C_{30}H_{36}NO^+$ 426.2791; found 426.2794 Δ 0.60 ppm.

Single Crystal Data for **8m**: C30H35NO, Mr =425.61. 100 K – monoclinic, C 2/c, a = 19.4632(5) Å, b = 6.9774(2) Å, c = 35.9129(8) Å, β = 92.285(2)°, V = 4873.2(2) Å^3, Data/restraints/parameters – 6035/584/333, Rint = 0.050, Final R1 = 0.0418, wR2 = 0.0981 (I>2σ(I)).

S22 5-Bromo-1-(2,3,5,6-tetramethylphenyl)pentan-1-one

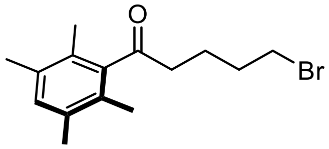

5-bromovaleric acid (2.72 g, 15.0 mmol) was dissolved in thionyl chloride (2.2 mL, 30 mmol) and the mixture was heated at 85 °C for 2 h. Once cooled to RT, the reaction mixture was concentrated *in vacuo*. The crude acyl chloride was dissolved in CH_2Cl_2 (10 mL) and added to a solution of durene (2.62 g, 19.5 mmol) in CH_2Cl_2 (40 mL) at 0 °C. $AlCl_3$ (2.59 g, 19.5 mmol) was added portion-wise and to mixture was stirred at RT for 10 min. The crude reaction mixture was poured onto ice (approx. 50 g) and the layers were separated. The aqueous layer was extracted with CH_2Cl_2 (2 × 50 mL) and the combined organic layers were washed sequentially with satd. aq. $NaHCO_3$ and brine, dried over Na_2SO_4 and concentrated *in vacuo*. Purification *via* flash column chromatography (eluent Pentane: Et_2O, 98:2 → 95:5) gave the title compound as a white solid (4.21 g, 94%).

m.p. = 54 – 56 °C;

^{1}H NMR (400 MHz, $CDCl_3$) δ 6.96 (s, 1H), 3.45 (t, *J* = 6.5 Hz, 2H), 2.71 (t, *J* = 7.1 Hz, 2H), 2.20 (s, 6H), 2.05 (s, 6H), 2.02 – 1.93 (m, 2H), 1.93 – 1.84 (m, 2H);

^{13}C NMR (101 MHz, $CDCl_3$) δ 211.1, 142.8, 134.6, 131.7, 128.0, 44.3, 33.4, 32.2, 22.0, 19.6, 16.1;

IR (film) ν_{max}/cm^{-1} 2941, 1702, 1470, 1402, 1302, 1256, 1229, 1107;

m/z (ESI$^+$) Found $[M+Na]^+$ = 319, $C_{15}H_{21}{}^{79}BrNaO$ requires 319;

HRMS (ESI$^+$) m/z: $[M+H]^+$ Calcd for $C_{15}H_{22}{}^{79}BrO^+$ 297.0849; found 297.0843, Δ 1.87 ppm.

S23 1-(2,3,5,6-Tetramethylphenyl)-5-tosylpentan-1-one

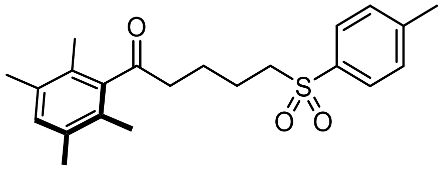

Bromide **S22** (1.49 g, 5.00 mmol) and sodium *p*-toluene sulfonate were dissolved in *N,N*-dimethylformamide (8.0 mL) and the reaction was heated at 80 °C for 4 h. The mixture was cooled to RT and diluted with CH_2Cl_2 (100 mL). The organic phase was washed with brine

(5 × 100 mL), dried over $MgSO_4$ and concentrated *in vacuo*. Purification *via* flash column chromatography (eluent Pentane/CH_2Cl_2/Et_2O, 70:20:10) gave the *title compound* as a white solid (1.40 g, 75%).

m.p. = 111 – 113 °C;

^{1}H NMR (400 MHz, $CDCl_3$) δ 7.94 – 7.66 (m, 2H), 7.44 – 7.31 (m, 2H), 6.94 (s, 1H), 3.21 – 3.00 (m, 2H), 2.77 – 2.57 (m, 2H), 2.45 (s, 3H), 2.19 (s, 6H), 2.00 (s, 6H), 1.88 – 1.70 (m, 4H);

^{13}C NMR (101 MHz, $CDCl_3$) δ 210.7, 144.9, 142.6, 136.3, 134.6, 131.8, 130.1, 128.2, 127.9, 56.4, 44.6, 22.5, 22.1, 21.8, 19.6, 16.1;

IR (film) ν_{max}/cm^{-1} 2925, 1698, 1300, 1148, 1088, 818, 732;

m/z (ESI$^+$) Found [M+H]$^+$ = 373, $C_{22}H_{29}O_3S$ requires 373;

HRMS (ESI$^+$) m/z: [M+H]$^+$ Calcd for $C_{22}H_{29}O_3S^+$ 373.1832; found 373.1831 Δ 0.26 ppm.

8n (2,3,5,6-Tetramethylphenyl)(1-(3-tosylpropyl)cyclopropyl)methanone

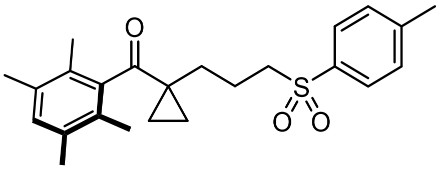

Ketone **S23** (112 mg, 0.300 mmol), $[Ir(cod)Cl]_2$ (2.0 mg, 1.0 mol%), dppBz (3.0 mg, 2.0 mol%), KOH (67 mg, 1.2 mmol) and 2-phenoxyethanol (0.37 mL, 3.0 mmol) were subjected to **General Procedure 3**. Purification *via* sequential flash column chromatography (eluent PhMe/Et_2O, 98.5:1.5 → 97:3) and preparative TLC (eluent PhMe/Et_2O, 80:20) gave the *title compound* as a white solid (63 mg, 53%).

m.p. = 88 – 90 °C;

^{1}H NMR (600 MHz, $CDCl_3$) δ 7.83 – 7.72 (m, 2H), 7.41 – 7.31 (m, 2H), 6.90 (s, 1H), 3.15 – 2.99 (m, 2H), 2.45 (s, 3H), 2.15 (s, 6H), 1.99 (s, 6H), 1.92 (m, 2H), 1.76 – 1.66 (m, 2H), 1.14 – 1.04 (m, 2H), 0.97 – 0.88 (m, 2H);

^{13}C NMR (151 MHz, $CDCl_3$) δ 211.8, 144.8, 138.3, 136.4, 134.4, 131.7, 130.0, 129.1, 128.2, 56.6, 33.8, 32.5, 21.8, 21.0, 19.5, 17.7, 16.6;

IR (film) ν_{max}/cm^{-1} 2923, 1667, 1301, 1149, 1089, 735;

m/z (ESI$^+$) Found [M+H]$^+$ = 399, $C_{24}H_{31}O_3S$ requires 399;

HRMS (ESI$^+$) m/z: $[M+Na]^+$ Calcd for $C_{24}H_{30}NaO_3S^+$ 421.1808; found 421.1824 Δ 3.82 ppm.

Cyclopropane Surrogate Optimisation and Mechanistic Studies

S24 1-(2,3,4,5,6-Pentamethylphenyl)-3-phenylpropan-1-one

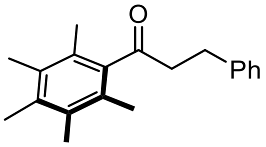

3-Phenylpropanoic acid (4.50 g, 30.0 mmol) was dissolved in thionyl chloride (5.3 mL, 73 mmol) and the mixture was refluxed for 16 h. Once cooled to RT, the reaction mixture was concentrated *in vacuo*. The crude acyl chloride was dissolved in CH_2Cl_2 (10 mL) and added to a solution of pentamethylbenzene (4.40 g, 30.0 mmol) in CH_2Cl_2 (90 mL) at 0 °C. $AlCl_3$ (5.00 g, 37.5 mmol) was added portion-wise and to mixture was stirred at RT for 1 h. The crude reaction mixture was poured onto ice (approx. 150 g) and the layers were separated. The aqueous layer was extracted with CH_2Cl_2 (2 × 100 mL) and the combined organic layers were washed sequentially with satd. aq. $NaHCO_3$ and brine, dried over Na_2SO_4 and concentrated *in vacuo*. Purification *via* flash column chromatography (eluent Pentane: Et_2O, 98:2) gave the title compound as a pale yellow solid (3.88 g, 46%).

^{1}H NMR (400 MHz, $CDCl_3$) δ 7.37 – 7.12 (m, 5H), 3.11 – 3.04 (m, 2H), 3.04 – 2.95 (m, 2H), 2.24 (s, 3H), 2.18 (s, 6H), 2.06 (s, 6H);

^{13}C NMR (101 MHz, $CDCl_3$) δ 210.9, 141.0, 140.5, 135.5, 133.1, 128.5, 128.5, 127.4, 126.1, 47.1, 29.4, 17.1, 16.7, 15.9;

m/z (ESI$^+$) Found $[M+H]^+$ = 281, $C_{20}H_{25}O$ requires 281.

The spectroscopic data matched that previously reported in the literature.[6]

S25 2-(4-Methoxyphenoxy)ethan-1-ol

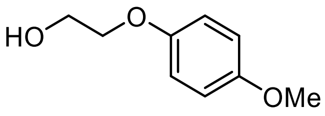

4-Methoxyphenol (2.48 g, 20.0 mmol) was dissolved in aqueous NaOH (50 mL, 30.0 mmol). 2-Bromoethanol (2.2 mL, 30.0 mmol) was added and the reaction was heated at 105 °C for

40 h. The reaction mixture was cooled to RT, diluted with 15 mL water and extracted with CH_2Cl_2 (3 × 50 mL). The combined organic layers were washed sequentially with 10% aq. NaOH, water and brine, dried over Na_2SO_4, and concentrated *in vacuo* to give the *title compound* as an off-white solid (2.72 g, 81%) which was used without further purification.

^{1}H NMR (400 MHz, $CDCl_3$) δ 6.91 – 6.77 (m, 4H), 4.08 – 3.99 (m, 2H), 3.94 (ddd, *J* = 6.5, 5.3, 3.7 Hz, 2H), 3.77 (s, 3H), 2.08 (t, *J* = 6.2 Hz, 1H).

^{13}C NMR (101 MHz, $CDCl_3$) δ 154.3, 152.9, 115.8, 114.9, 70.1, 61.7, 55.9;

m/z (ESI^+) Found $[M+Na]^+$ = 191, $C_9H_{12}NaO_3$ requires 191.

The spectroscopic data matched that previously reported in the literature.[10]

14 2-Benzyl-4-(4-methoxyphenoxy)-1-(2,3,4,5,6-pentamethylphenyl)butan-1-one

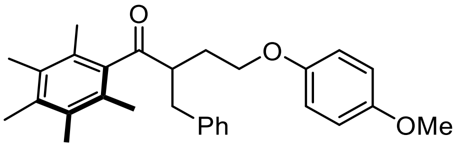

Ketone **S24** (84 mg, 0.30 mmol), alcohol **S25** (504 mg, 3.00 mmol), $[Ir(cod)Cl]_2$ (2.0 mg, 1.0 mol%), dppBz (3.0 mg, 2.0 mol%), and KOH (67 mg, 1.20 mmol) were added to a 2-5 mL microwave vial. The vial was sealed and evacuated and backfilled with Ar (three times). PhMe (75 µL) was added and the reaction was heated for 48 h at 110 °C, cooled to RT, filtered through a silica plug (Et_2O elution) and concentrated *in vacuo*. Purification *via* flash column chromatography (eluent Pentane/Et_2O, 95:5 → 92:8) gave the *title compound* as a yellow solid (55 mg, 43%).

m.p. = 95 – 97 °C;

^{1}H NMR (600 MHz, $CDCl_3$) δ 7.26 – 7.22 (m, 2H), 7.17 (td, *J* = 7.4, 1.6 Hz, 3H), 6.82 – 6.73 (m, 2H), 6.71 – 6.59 (m, 2H), 3.92 – 3.82 (m, 2H), 3.75 (s, 3H), 3.37 (ddt, *J* = 9.1, 7.0, 5.4 Hz, 1H), 3.22 (dd, *J* = 13.7, 5.2 Hz, 1H), 2.63 (dd, *J* = 13.8, 9.1 Hz, 1H), 2.26 – 2.19 (m, 4H), 2.16 (s, 6H), 2.12 – 1.92 (m, 6H), 1.83 (dtd, *J* = 12.3, 6.7, 5.5 Hz, 1H).

^{13}C NMR (151 MHz, $CDCl_3$) δ 212.8, 153.9, 152.9, 139.7, 139.3, 135.9, 133.3, 129.3, 128.6 (br.) 128.5, 126.4, 115.6, 114.7, 66.3, 55.8, 52.3, 36.4, 28.9, 17.9, 16.9, 16.2;

IR (film) ν_{max}/cm^{-1} 3026, 2932, 1691, 1508, 1231;

m/z (ESI$^+$) Found $[M+H]^+$ = 431, $C_{29}H_{35}O_3$ requires 431;

HRMS (ESI$^+$) m/z: $[M+Na]^+$ Calcd for $C_{29}H_{34}NaO_3^+$ 453.2400; found 453.2401, Δ 0.22 ppm.

Derivatisation

S26 Phthaloyl peroxide

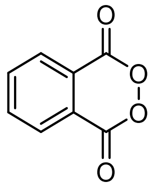

Phthalyol chloride (1.45 mL, 10.0 mmol) was added to a vigorously stirred suspension of $Na_2CO_3{\cdot}1.5H_2O_2$ (2.36 g, 15.0 mmol) and Na_2CO_3 (530 mg, 5.0 mmol) in water-saturated CH_2Cl_2* (50 mL) and the resulting mixture was stirred vigorously for 3 h. The crude reaction mixture was filtered through a Celite® pad, eluted with CH_2Cl_2 and the filtrate was concentrated *in vacuo* (ensuring that the water bath temperature did not exceed 30 °C). The residue was re-dissolved in a minimum amount of warm benzene and poured into ice-cold pentane (100 mL). The resulting solid was filtered under reduced pressure and washed with ice-cold pentane (3 × 30 mL) to give the *title compound* as a white solid (1.37 g, 83%);

^{1}H NMR (400 MHz, $CDCl_3$) δ 8.35 – 8.25 (m, 2H), 8.07 – 7.98 (m, 2H);

^{13}C NMR (101 MHz, $CDCl_3$) δ 162.1, 136.6, 130.3, 123.8;

m/z (ESI$^+$) Found $[M+H]^+$ = 165, $C_8H_5O_4$ requires 165.

The spectroscopic data matched that previously reported in the literature.[11]

---

* Water-saturated CH_2Cl_2 can be prepared by simply putting CH_2Cl_2 and DI H_2O in a separating funnel and shaking it vigorously, then removing only the organic phase and avoiding any obvious water droplets.

15a (1-Butylcyclopropyl)(4-hydroxy-2,3,5,6-tetramethylphenyl)methanone

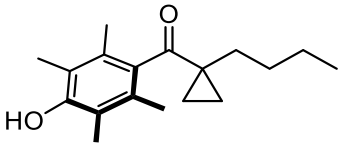

Phthaloyl peroxide **S26** (58 mg, 0.35 mmol) and ketone **8d** (70 mg, 0.27 mmol) in 1,1,1,3,3,3-hexafluoro-2-propanol (2.7 mL) were subjected to **General Procedure 4** (heated at 40 °C for 16 h, after the addition of MeOH (2.7 mL) and satd. $NaHCO_3$ (0.29 mL) the mixture was heated at 40 °C for a further 6 h). Purification *via* flash column chromatography (eluent Pentane/Et_2O, 85:15) gave the *title compound* as an off-white solid (55 mg, 74%);

m.p. = 111 – 113 °C, *via* oxidation to the corresponding quinone (yellow solid);

^{1}H NMR (600 MHz, $CDCl_3$) δ 4.67 (br s, 1H), 2.11 (s, 6H), 2.08 (s, 6H), 1.66 – 1.59 (m, 2H), 1.44 – 1.32 (m, 2H), 1.32 – 1.21 (m, 2H), 1.08 (q, *J* = 4.0 Hz, 2H), 0.94 – 0.88 (m, 2H), 0.87 (t, *J* = 7.3 Hz, 3H);

^{13}C NMR (151 MHz, $CDCl_3$) δ 212.6, 151.7, 132.1, 130.2, 119.6, 34.3, 33.5, 29.6, 23.2, 17.8, 17.3, 14.1, 11.8;

IR (film) ν_{max}/cm^{-1} 3433, 2955, 2932, 2860, 1650, 1124, 1098;

m/z (ESI$^+$) Found [M+H]$^+$ = 275, $C_{18}H_{27}O_2$ requires 275;

HRMS (ESI$^+$) m/z: [M+H]$^+$ Calcd for $C_{18}H_{27}O_2^+$ 275.2006; found 275.2006, Δ 0.02 ppm.

16a 1-Butylcyclopropane-1-carboxylic acid

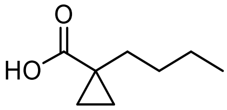

A solution of CAN (ceric ammonium nitrate) (548 mg, 1.00 mmol) in water (1.0 mL) was added to a stirred suspension of phenol **15a** (55 mg, 0.20 mmol) in MeCN (1.0 mL) and the resulting mixture was stirred at RT for 5 min. Water (6.0 mL) was added and the reaction was stirred for a further 10 min. The reaction was diluted with CH_2Cl_2 (10 mL) and water (4 mL) was added. The layers were separated and the aqueous phase was extracted with CH_2Cl_2 (10 mL). NaOH (3 M, 30 mL) was added to the combined organic layers, the layers separated and the aqueous layer acidified with HCl (3 M, 35 mL) and extracted with CH_2Cl_2 (3 × 60 mL). The

combined organic extracts were washed with brine, dried over Na_2SO_4 and concentrated *in vacuo* to give the *title compound* as a yellow oil which was not purified further (24.5 mg, 86%).

^{1}H NMR (600 MHz, $CDCl_3$) δ 1.54 – 1.47 (m, 2H), 1.47 – 1.39 (m, 2H), 1.33 – 1.28 (m, 2H), 1.28 – 1.22 (m, 2H), 0.89 (t, *J* = 7.3 Hz, 3H), 0.81 – 0.67 (m, 2H);

^{13}C NMR (151 MHz, $CDCl_3$) δ 182.2, 33.5, 29.9, 23.5, 23.0, 16.6, 14.2;

m/z (ESI^-) Found $[M-H]^-$ = 141, $C_8H_{13}O_2$ requires 141.

The spectroscopic data matched that previously reported in the literature.[12]

15b (4-Hydroxy-2,3,5,6-tetramethylphenyl)(1-isopentylcyclopropyl)methanone

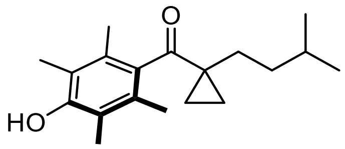

Phthaloyl peroxide **S26** (111 mg, 0.675 mmol) and ketone **8f** (122 mg, 0.450 mmol) in 1,1,1,3,3,3-hexafluoro-2-propanol (4.5 mL) were subjected to **General Procedure 4** (heated at 40 °C for 24 h, after the addition of MeOH (4.0 mL) and satd. $NaHCO_3$ (0.5 mL), the mixture was heated at 40 °C for a further 18 h). Purification *via* flash column chromatography (eluent Pentane/Et_2O, 85:15) gave the title compound as a white solid (75 mg, 58%).

m.p. = 83 – 84 °C;

^{1}H NMR (600 MHz, $CDCl_3$) δ 4.81 – 4.44 (m, 1H), 2.11 (s, 6H), 2.08 (s, 6H), 1.64 – 1.57 (m, 2H), 1.50 – 1.42 (m, 1H), 1.34 – 1.24 (m, 2H), 1.09 (q, *J* = 3.6 Hz, 2H), 0.92 – 0.87 (m, 2H), 0.84 (d, *J* = 6.6 Hz, 6H);

^{13}C NMR (151 MHz, $CDCl_3$) δ 212.5, 151.7, 132.2, 130.2, 119.6, 36.4, 34.3, 31.5, 28.4, 22.6, 17.9, 17.3, 11.8;

IR (film) ν_{max}/cm^{-1} 3480, 2955, 2870, 1654, 1458, 1124, 1102, 762, 737;

m/z (ESI^+) Found $[M+H]^+$ = 289, $C_{19}H_{29}O_2$ requires 289;

HRMS (ESI^+) m/z: $[M+Na]^+$ Calcd for $C_{19}H_{28}NaO_2^+$ 311.1982; found 311.1984, Δ 0.79 ppm.

16b 1-Isopentylcyclopropane-1-carboxylic acid

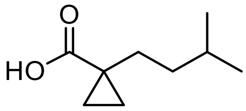

A solution of CAN (ceric ammonium nitrate) (548 mg, 1.00 mmol) in water (1.0 mL) was added to a stirred suspension of phenol **15b** (58 mg, 0.20 mmol) in MeCN (1.0 mL) and the resulting mixture was stirred at RT for 5 min. Water (6.0 mL) was added and the reaction was stirred for a further 10 min. The reaction was diluted with CH_2Cl_2 (10 mL) and water (4 mL) was added. The layers were separated and the aqueous phase was extracted with CH_2Cl_2 (10 mL). NaOH (3 M, 30 mL) was added to the combined organic layers, the layers separated and the aqueous layer acidified with HCl (3 M, 35 mL) and extracted with CH_2Cl_2 (3 × 60 mL). The combined organic extracts were washed with brine, dried over Na_2SO_4 and concentrated *in vacuo*. Purification *via* flash column chromatography (eluent $CH_2Cl_2 \rightarrow CH_2Cl_2$/EtOAc/$HCO_2H$, 97.95:2:0.05) gave the *title compound* as a colourless oil (25.5 mg, 82%).

^{1}H NMR (400 MHz, $CDCl_3$) δ 1.55 – 1.45 (m, 3H), 1.38 – 1.30 (m, 2H), 1.29 – 1.22 (m, 2H), 0.87 (d, *J* = 6.6 Hz, 6H), 0.77 – 0.71 (m, 2H);

^{13}C NMR (101 MHz, $CDCl_3$) δ 182.7, 36.7, 31.5, 28.4, 23.6, 22.7, 16.7;

IR (film) ν_{max}/cm^{-1} 3017, 2957, 1693, 1458, 1346, 1293, 1229, 1190;

HRMS (ESI$^-$) m/z: [M-H]$^-$ Calcd for $C_9H_{15}O_2^-$ 155.1078; not found.

15c (1-(4-(Benzyloxy)butyl)cyclopropyl)(4-hydroxy-2,3,5,6-tetramethylphenyl)methanone

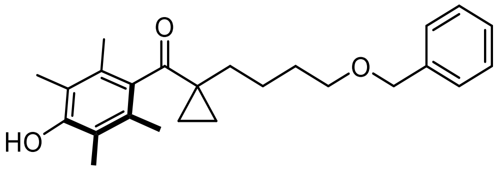

Phthaloyl peroxide **S26** (175 mg, 1.07 mmol) and ketone **8g** (259 mg, 0.710 mmol) in 1,1,1,3,3,3-hexafluoro-2-propanol (7.0 mL) were subjected to **General Procedure 4** (heated at 40 °C for 24 h, after the addition of MeOH (2.7 mL) and satd. $NaHCO_3$ (0.29 mL) the mixture was heated at 40 °C for a further 18 h). Purification *via* flash column chromatography (eluent Pentane/Et_2O, 80:20 → 70:30) gave the title compound as an off-white solid (195 mg, 72%).

m.p. = 83 – 84 °C;

^{1}H NMR (600 MHz, $CDCl_3$) δ 7.37 – 7.27 (m, 5H), 4.49 (s, 2H), 3.44 (t, *J* = 6.5 Hz, 2H), 2.10 (s, 6H), 2.07 (s, 6H), 1.68 – 1.55 (m, 4H), 1.55 – 1.47 (m, 2H), 1.08 (q, *J* = 4.0 Hz, 2H), 0.95 – 0.88 (m, 2H);

^{13}C NMR (151 MHz, $CDCl_3$) δ 212.5, 151.7, 138.8, 132.0, 130.2, 128.5, 127.8, 127.6, 119.7, 73.0, 70.4, 34.3, 33.6, 30.2, 24.1, 17.8, 17.3, 11.8;

IR (film) ν_{max}/cm^{-1} 3449, 2942, 2863, 1719, 1120, 737, 699;

m/z (ESI$^+$) Found $[M+Na]^+$ = 403, $C_{25}H_{32}NaO_3$ requires 403;

HRMS (ESI$^+$) m/z: $[M+Na]^+$ Calcd for $C_{25}H_{32}NaO_3^+$ 403.2244; found 403.2243, Δ 0.17 ppm.

16c 1-(4-(Benzyloxy)butyl)cyclopropane-1-carboxylic acid

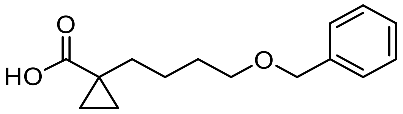

A solution of CAN (ceric ammonium nitrate) (822 mg, 1.50 mmol) in water (1.5 mL) was added to a stirred solution of phenol **15c** (114 mg, 0.300 mmol) in MeCN (1.5 mL) and the resulting mixture was stirred at RT for 15 min. Water (6.0 mL) was added and the reaction was stirred for a further 15 min. The reaction was diluted with CH_2Cl_2 (20 mL) and water (15 mL) was added. The layers were separated and the aqueous phase was extracted with CH_2Cl_2 (2 × 20 mL). The combined organic extracts were washed with brine, dried over Na_2SO_4 and concentrated *in vacuo.* Purification *via* flash column chromatography (eluent CH_2Cl_2/EtOAc/AcOH, 94:5:1) gave the *title compound* as a yellow oil which solidified on standing (46 mg, 61%).

m.p. = 46 – 48 °C;

^{1}H NMR (400 MHz, $CDCl_3$) δ 7.38 – 7.27 (m, 5H), 4.50 (s, 2H), 3.46 (t, *J* = 6.3 Hz, 2H), 1.70 – 1.47 (m, 6H), 1.26 (q, *J* = 3.9 Hz, 2H), 0.81 – 0.64 (m, 2H);

^{13}C NMR (101 MHz, $CDCl_3$) δ 182.0, 138.7, 128.5, 127.8, 127.6, 73.1, 70.4, 33.5, 30.0, 24.4, 23.4, 16.6;

IR (film) ν_{max}/cm^{-1} 3089, 2942, 2864, 1687, 1456, 1213, 1104, 737, 698;

HRMS (ESI$^-$) m/z: $[M-H]^-$ Calcd for $C_{15}H_{19}O_3^-$ 247.1340; found 247.1339 Δ 0.26 ppm.

15d 1-(Cyclopropylmethyl)cyclopropyl)(4-hydroxy-2,3,5,6-tetramethylphenyl)methanone

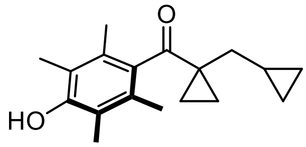

Phthaloyl peroxide **S26** (148 mg, 0.900 mmol) and ketone **8e** (166 mg, 0.650 mmol) in 1,1,1,3,3,3-hexafluoro-2-propanol (6.0 mL) were subjected to **General Procedure 4** (heated at 40 °C for 24 h, after the addition of MeOH (5.4 mL) and satd. $NaHCO_3$ (0.6 mL) the mixture was heated at 40 °C for a further 18 h). Purification *via* flash column chromatography (eluent Pentane/Et_2O, 85:15) gave the title compound as a white solid (94 mg, 53%).

m.p. = 89 – 91 °C;

^{1}H NMR (400 MHz, $CDCl_3$) δ 4.80 (br s, 1H), 2.10 (s, 6H), 2.09 (s, 6H), 1.63 (d, *J* = 6.9 Hz, 2H), 1.14 – 1.08 (m, 2H), 1.07 – 1.00 (m, 2H), 0.76 (m, 1H), 0.45 – 0.31 (m, 2H), 0.06 – -0.01 (m, 2H);

^{13}C NMR (101 MHz, $CDCl_3$) δ 213.0, 151.7, 132.3, 130.1, 119.8, 37.2, 34.2, 17.2, 17.1, 11.8, 8.6, 4.5.

IR (film) ν_{max}/cm^{-1} 3433, 2922, 1653, 1350, 1299, 1219, 1103, 1014, 930, 762;

m/z (ESI$^+$) Found $[M+H]^+$ = 273, $C_{18}H_{25}O_2$ requires 273;

HRMS (ESI$^+$) m/z: $[M+H]^+$ Calcd for $C_{18}H_{25}O_2^+$ 273.1849; found 273.1844, Δ 1.69 ppm.

16d 1-(Cyclopropylmethyl)cyclopropane-1-carboxylic acid

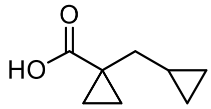

A solution of CAN (ceric ammonium nitrate) (822 mg, 1.50 mmol) in water (1.5 mL) was added to a stirred suspension of phenol **15d** (82 mg, 0.30 mmol) in MeCN (1.5 mL) and the resulting mixture was stirred at RT for 5 min. Water (6.0 mL) was added and the reaction was stirred for a further 10 min. The reaction was diluted with CH_2Cl_2 (10 mL) and water (4 mL) was added. The layers were separated and the aqueous phase was extracted with CH_2Cl_2 (10 mL). NaOH (3 M, 30 mL) was added to the combined organic layers, the layers separated and the aqueous layer acidified with HCl (3 M, 35 mL) and extracted with CH_2Cl_2 (3 × 60 mL). The

combined organic extracts were washed with brine, dried over Na_2SO_4 and concentrated *in vacuo*. Purification *via* flash column chromatography (eluent $CH_2Cl_2 \rightarrow CH_2Cl_2$/EtOAc/$HCO_2H$, 97.95:2:0.05) gave the *title compound* as a colourless oil (25.5 mg, 61%).

^{1}H NMR (400 MHz, MeOD) δ 1.52 (d, *J* = 6.8 Hz, 2H), 1.14 (d, *J* = 2.9 Hz, 2H), 0.91 – 0.84 (m, 1H), 0.83 – 0.79 (m, 2H), 0.47 – 0.26 (m, 2H), 0.08 (m, 2H);

^{13}C NMR (101 MHz, MeOD) δ 179.6, 38.9, 24.3, 15.2, 9.7, 4.6;

IR (film) ν_{max}/cm^{-1} 3080, 3010, 2911, 2840, 1689, 1425, 1340, 1248, 1202, 1031, 929;

HRMS (ESI$^-$) m/z: [M-H]$^-$ Calcd for $C_8H_{11}O_2^-$ 139.0765; not found.

15e (1-Benzylcyclopropyl)(4-hydroxy-2,3,5,6-tetramethylphenyl)methanone

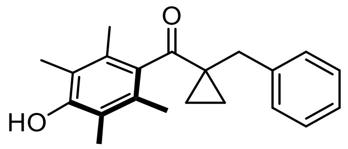

Phthaloyl peroxide **S26** (111 mg, 0.675 mmol) and ketone **8a** (131 mg, 0.45 mmol) in 1,1,1,3,3,3-hexafluoro-2-propanol (4.5 mL) were subjected to **General Procedure 4** (heated at 40 °C for 18 h, after the addition of MeOH (4.0 mL) and satd. $NaHCO_3$ (0.5 mL) the mixture was heated at 40 °C for a further 18 h.) Purification *via* flash column chromatography (eluent Pentane/Et_2O, 85:15) gave the *title compound* as a colourless oil (85 mg, 61%);

^{1}H NMR (400 MHz, $CDCl_3$) δ 7.23 – 7.13 (m, 3H), 7.11 – 7.03 (m, 2H), 4.66 (s, 1H), 3.02 (s, 2H), 2.08 (s, 6H), 1.92 (s, 6H), 1.20 (q, *J* = 4.0 Hz, 2H), 1.05 – 0.87 (m, 2H);

^{13}C NMR (101 MHz, $CDCl_3$) δ 212.4, 151.8, 139.2, 132.4, 130.1, 130.0, 128.0, 126.3, 119.7, 38.1, 34.6, 17.4, 17.0, 11.8;

IR (film) ν_{max}/cm^{-1} 3567, 2921, 2861, 1699, 1653, 1455, 1296, 1107, 745, 701;

m/z (ESI$^+$) Found [M+H]$^+$ = 309, $C_{21}H_{25}O_2$ requires 309;

HRMS (ESI$^+$) m/z: [M+H]$^+$ Calcd for $C_{21}H_{25}O_2^+$ 309.1849; found 309.1849, Δ 0.10 ppm.

16e 1-Benzylcyclopropane-1-carboxylic acid

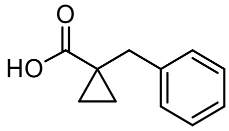

A solution of CAN (ceric ammonium nitrate) (1.01 g, 1.85 mmol) in water (1.85 mL) was added to a stirred solution of phenol **15e** (115 mg, 0.37 mmol) in MeCN (1.85 mL) and the resulting mixture was stirred at RT for 15 min. Water (6.0 mL) was added and the reaction was stirred for a further 15 min. The reaction was diluted with CH_2Cl_2 (20 mL) and water (15 mL) was added. The layers were separated and the aqueous phase was extracted with CH_2Cl_2 (2 × 20 mL). The combined organic extracts were washed with brine, dried over Na_2SO_4 and concentrated *in vacuo.* Purification *via* flash column chromatography (eluent $CH_2Cl_2 \rightarrow CH_2Cl_2$/EtOAc/AcOH, 94.95:5:0.05) gave the *title compound* as a yellow solid (48.4 mg, 75%).

^{1}H NMR (400 MHz, $CDCl_3$) δ 7.33 – 7.14 (m, 5H), 2.99 (s, 2H), 1.35 (q, *J* = 4.0 Hz, 2H), 0.90 – 0.85 (m, 2H);

^{13}C NMR (101 MHz, $CDCl_3$) δ 181.6, 139.3, 129.4, 128.3, 126.4, 37.9, 23.6, 16.1;

m/z (ESI^-) Found $[M\text{-}H]^-$ = 175, $C_{11}H_{11}O_2$ requires 175.

The spectroscopic data matched that previously reported in the literature.[13]

NMR Spectra of Novel Compounds

2 Cyclopropyl(2,3,4,5,6-pentamethylphenyl)methanone

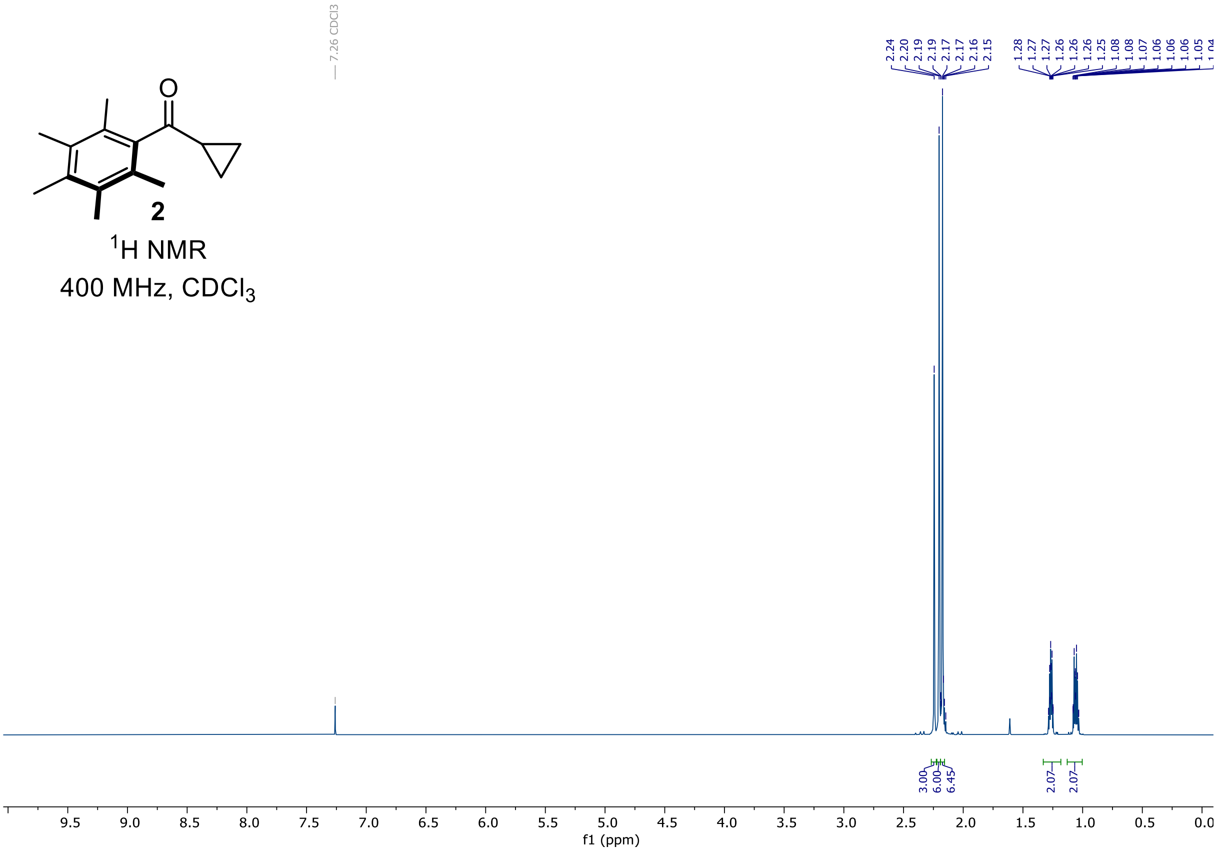

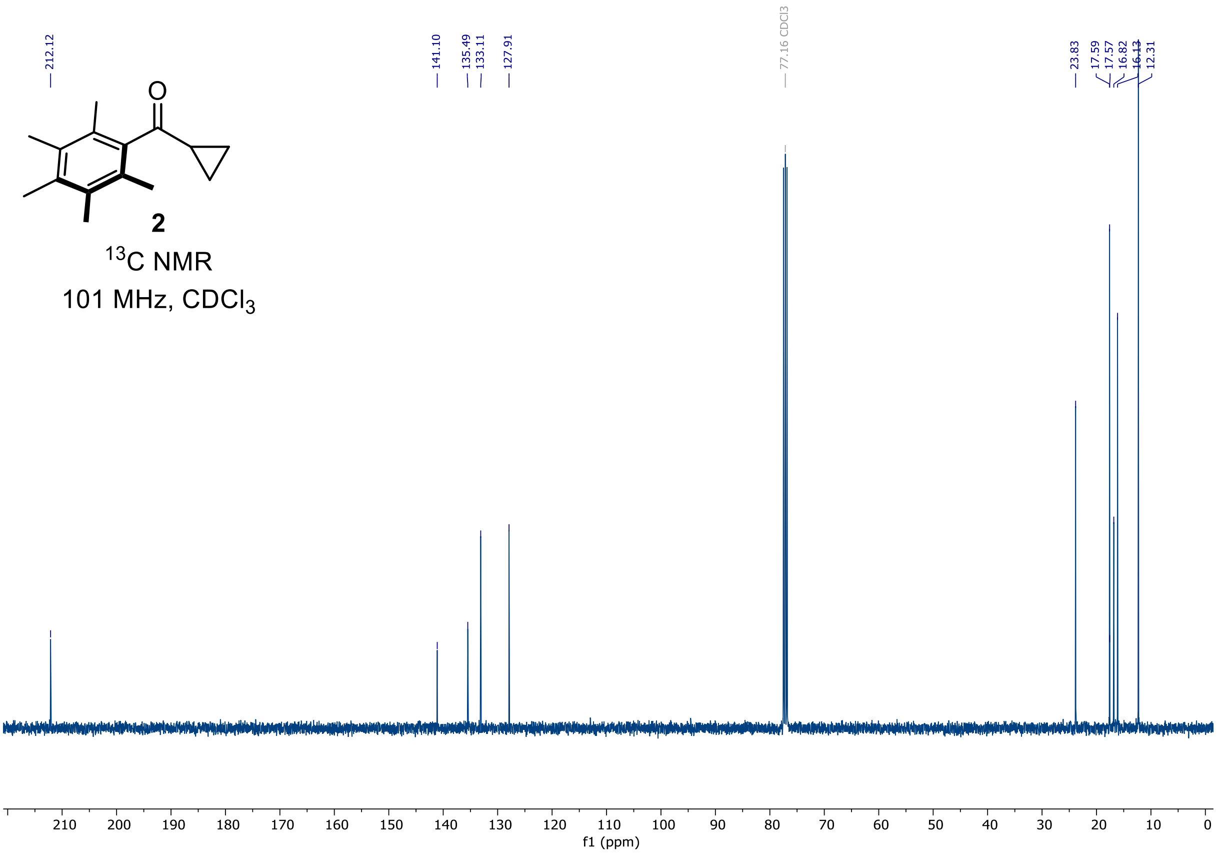

3 1-(2,3,4,5,6-Pentamethylphenyl)-4-(phenylthio)butan-1-one

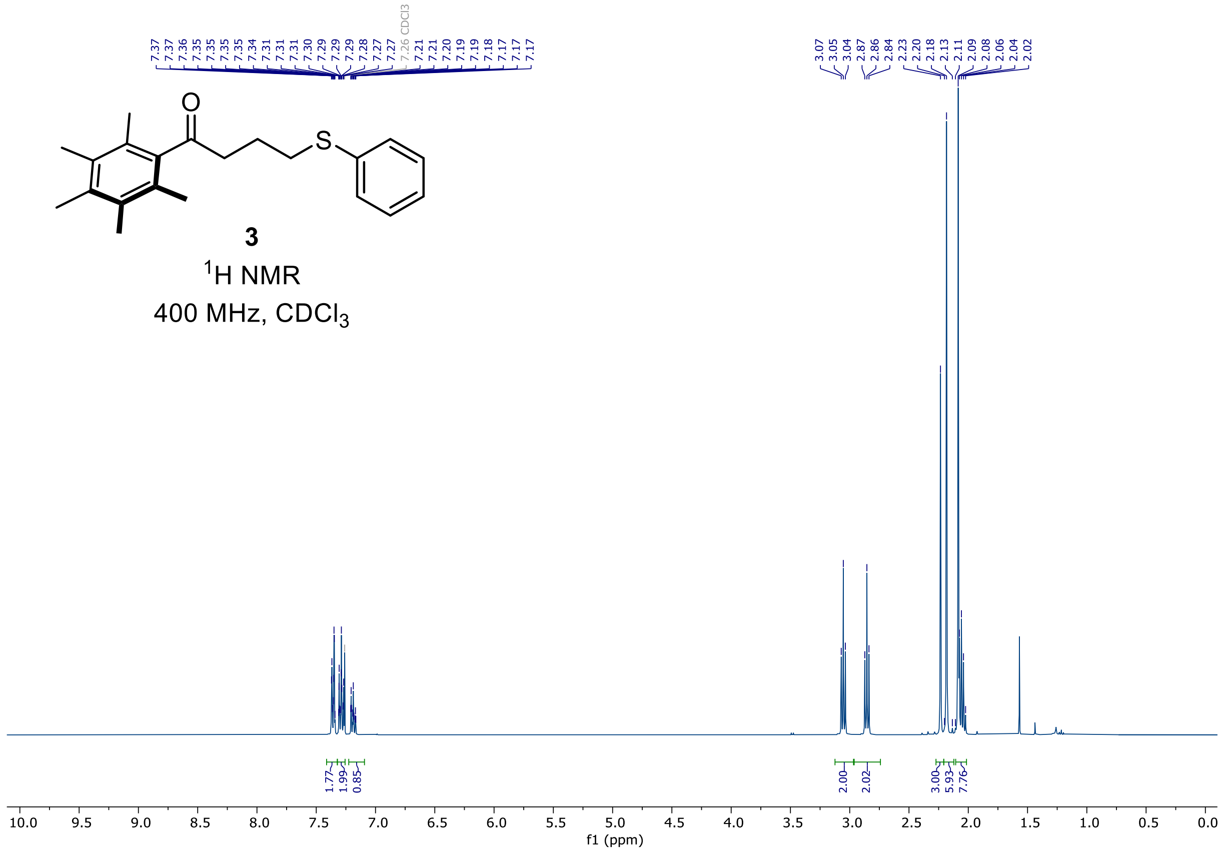

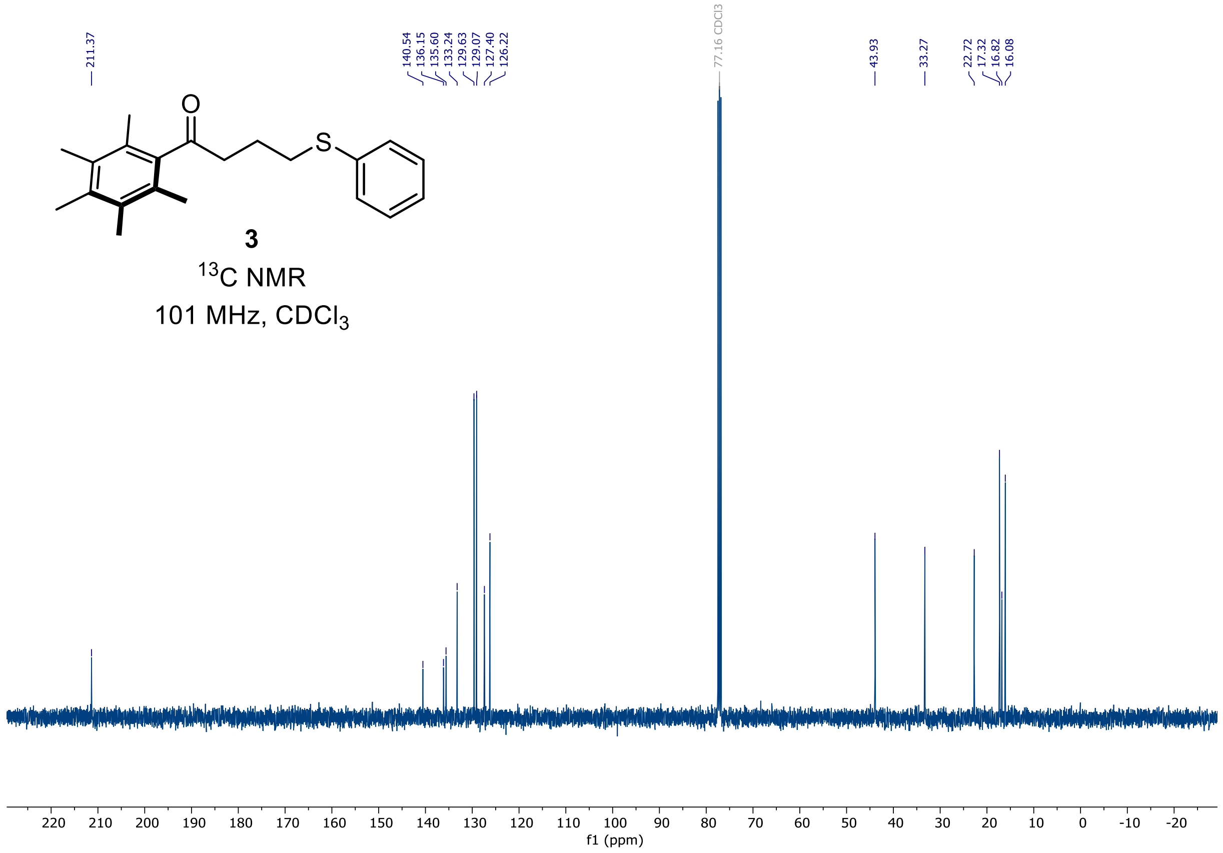

4 (1-Benzylcyclopropyl)(2,3,4,5,6-pentamethylphenyl)methanone

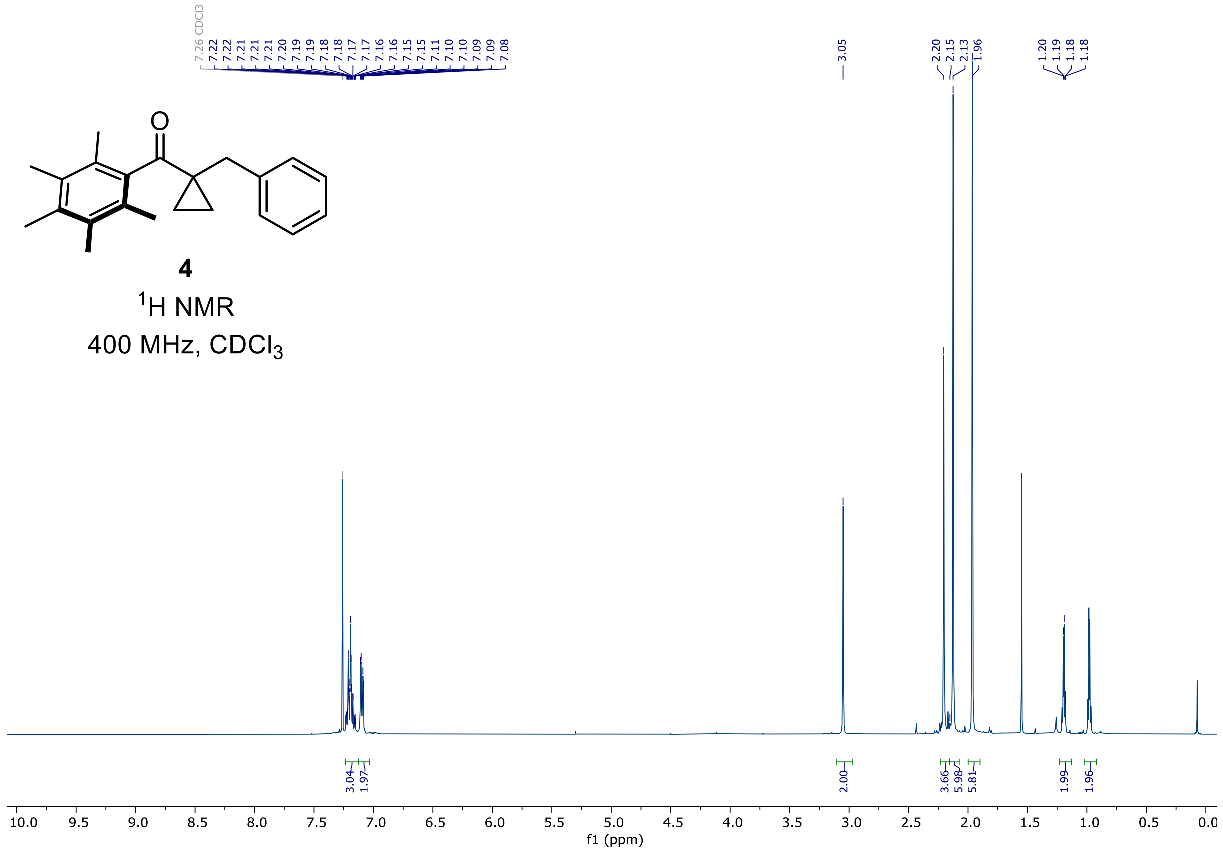

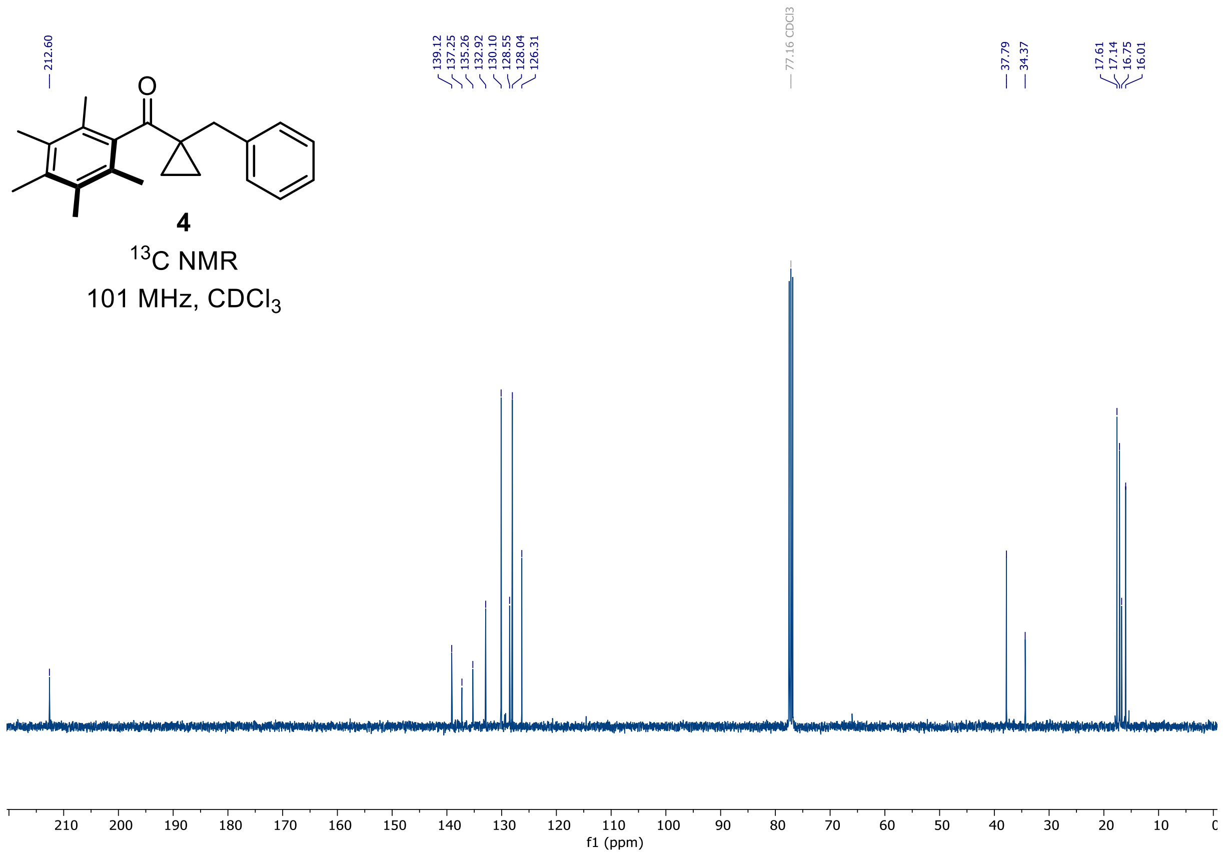

6a 1-(2,3,4,5,6-Pentamethylphenyl)-4-phenoxybutan-1-one

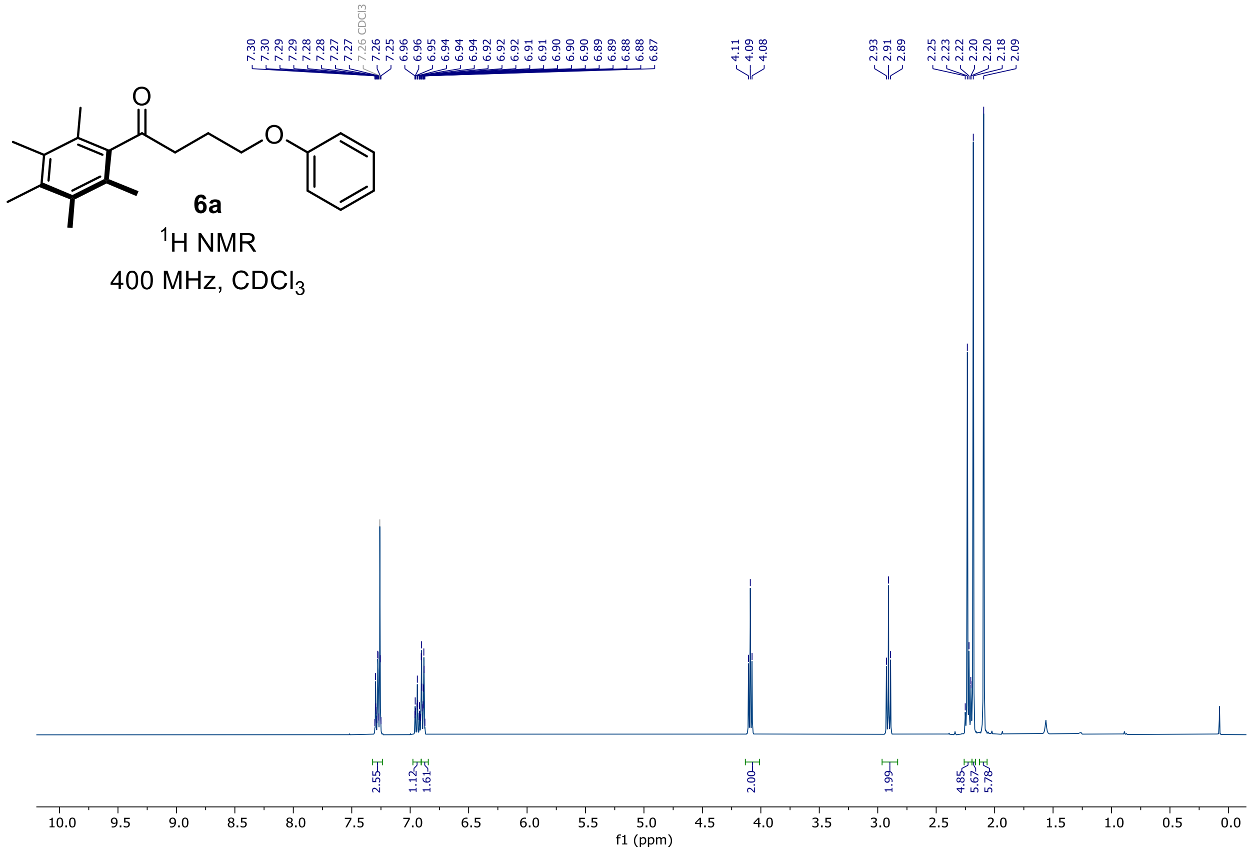

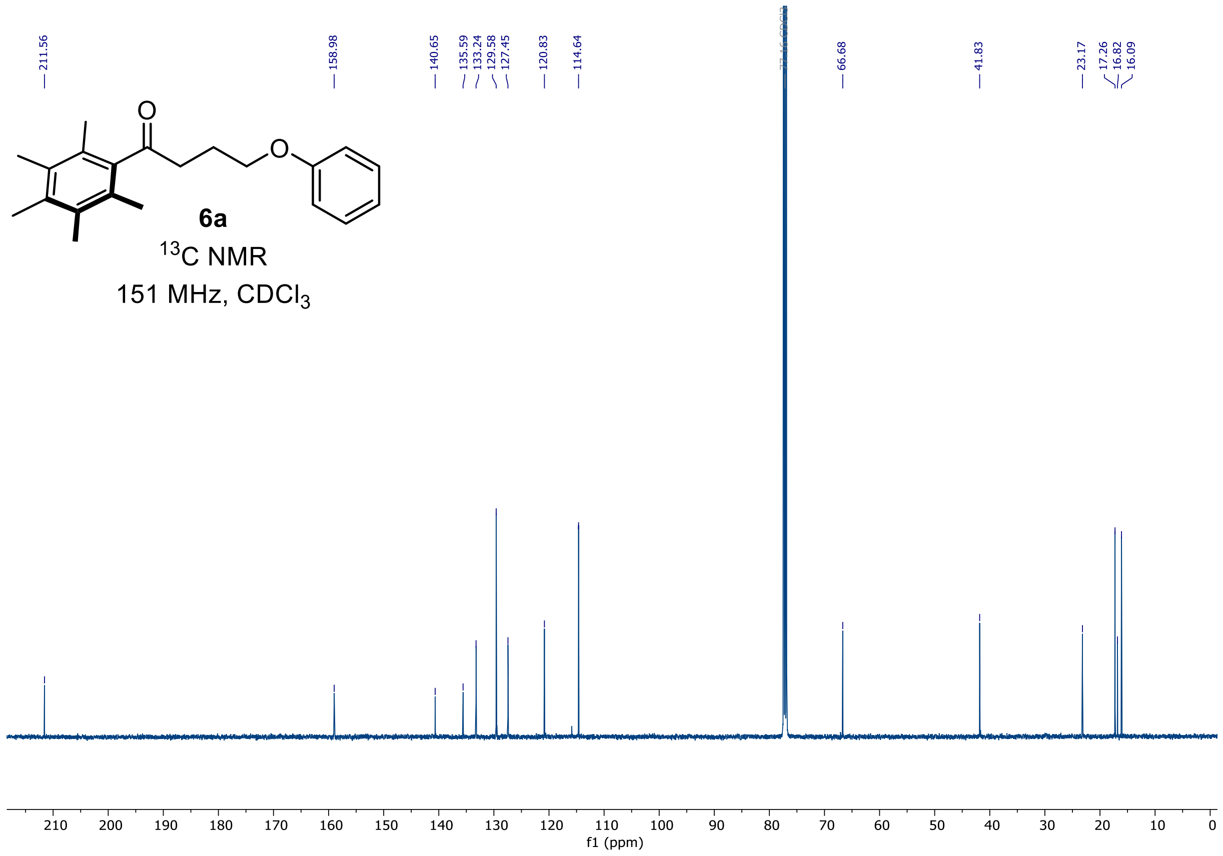

6b 4-(4-Nitrophenoxy)-1-(2,3,4,5,6-pentamethylphenyl)butan-1-one

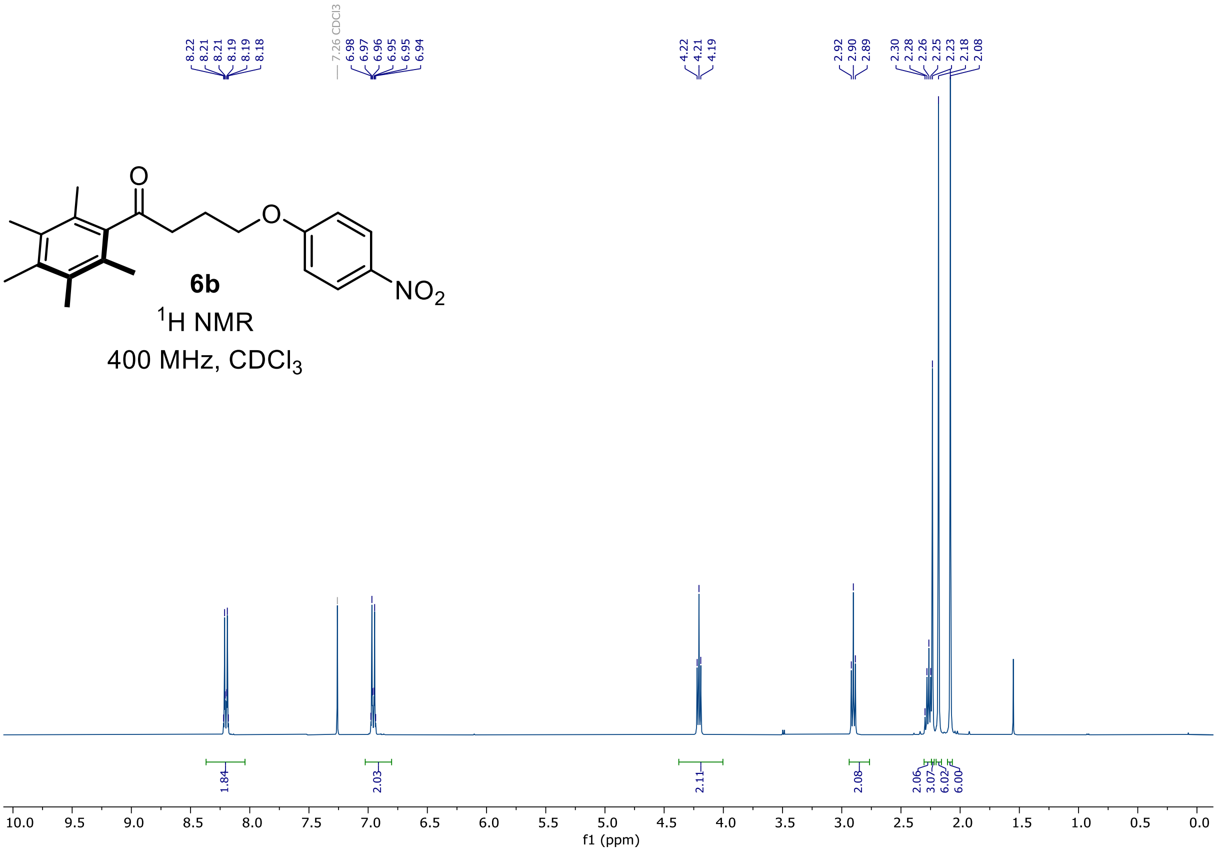

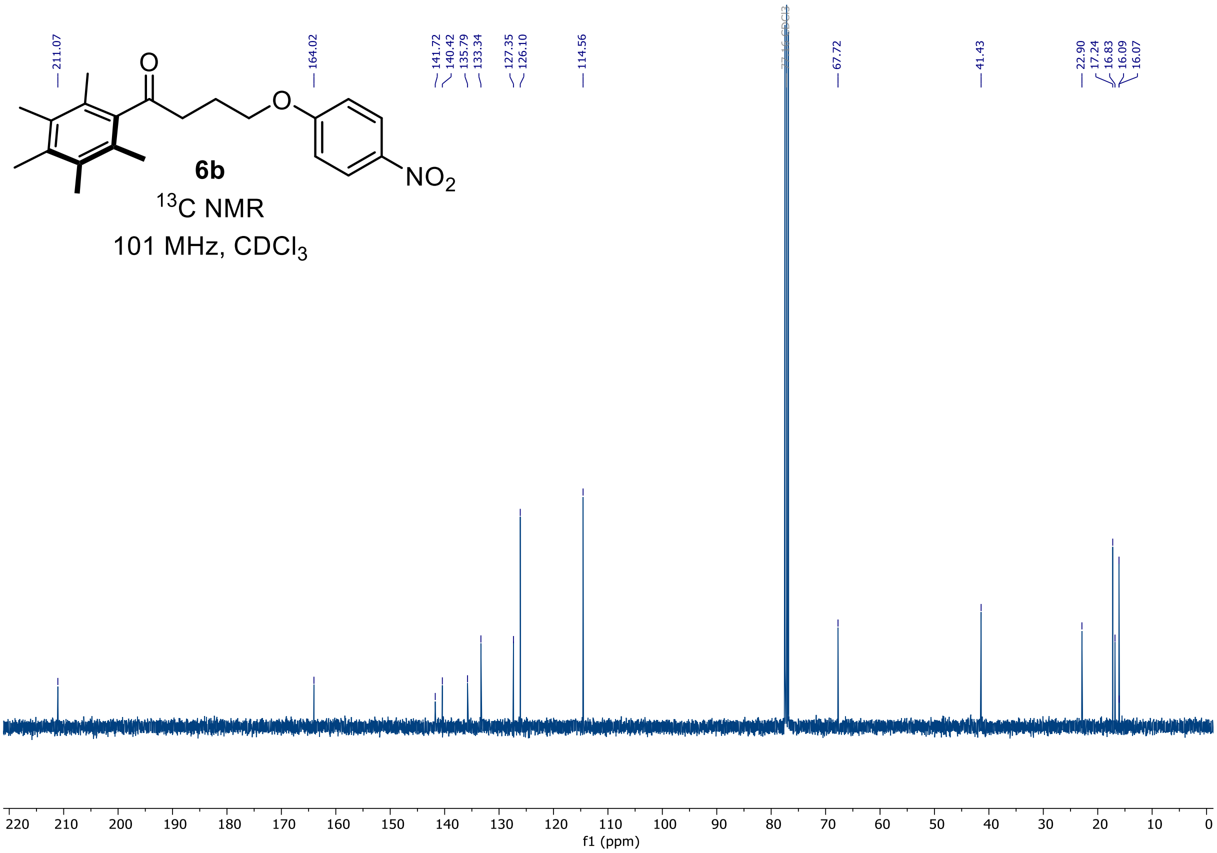

6c 4-(3-Nitrophenoxy)-1-(2,3,4,5,6-pentamethylphenyl)butan-1-one

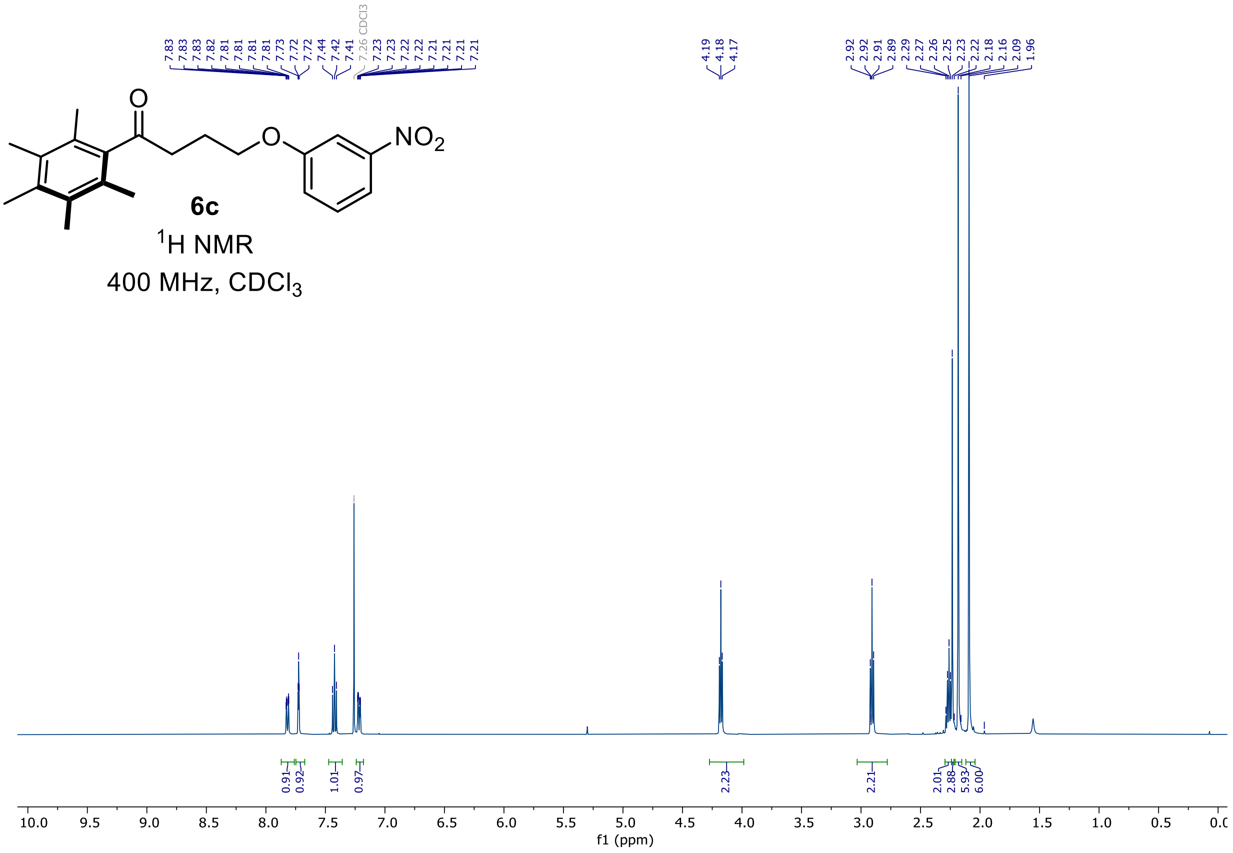

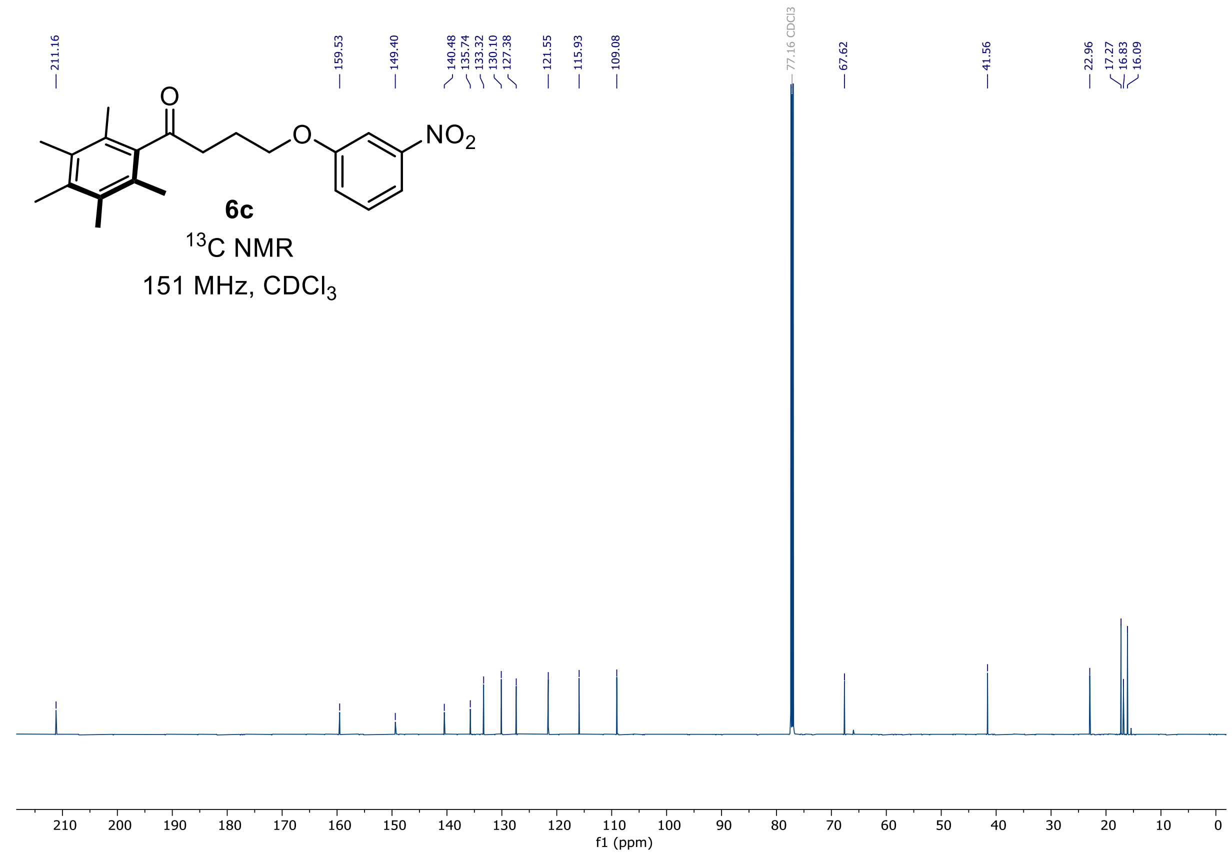

6d 4-(4-oxo-4-(2,3,4,5,6-pentamethylphenyl)butoxy)benzonitrile

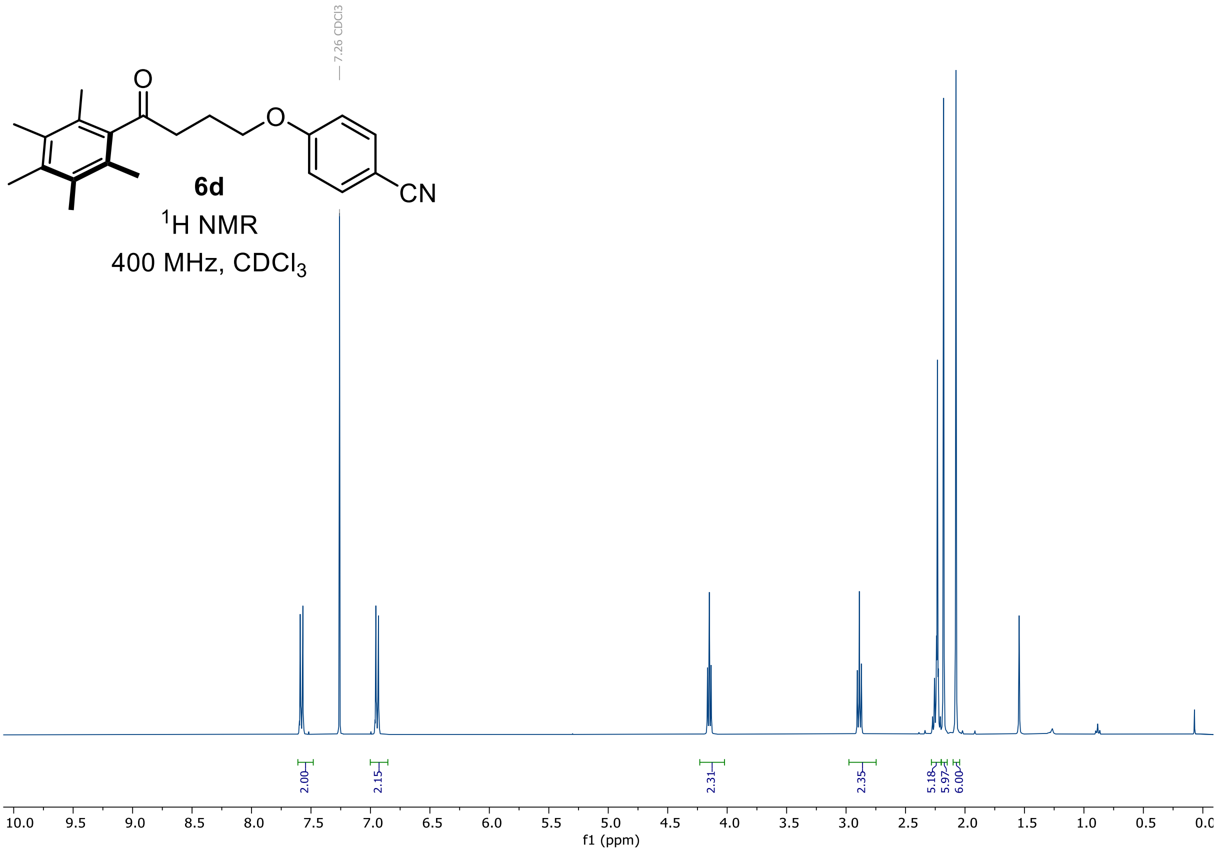

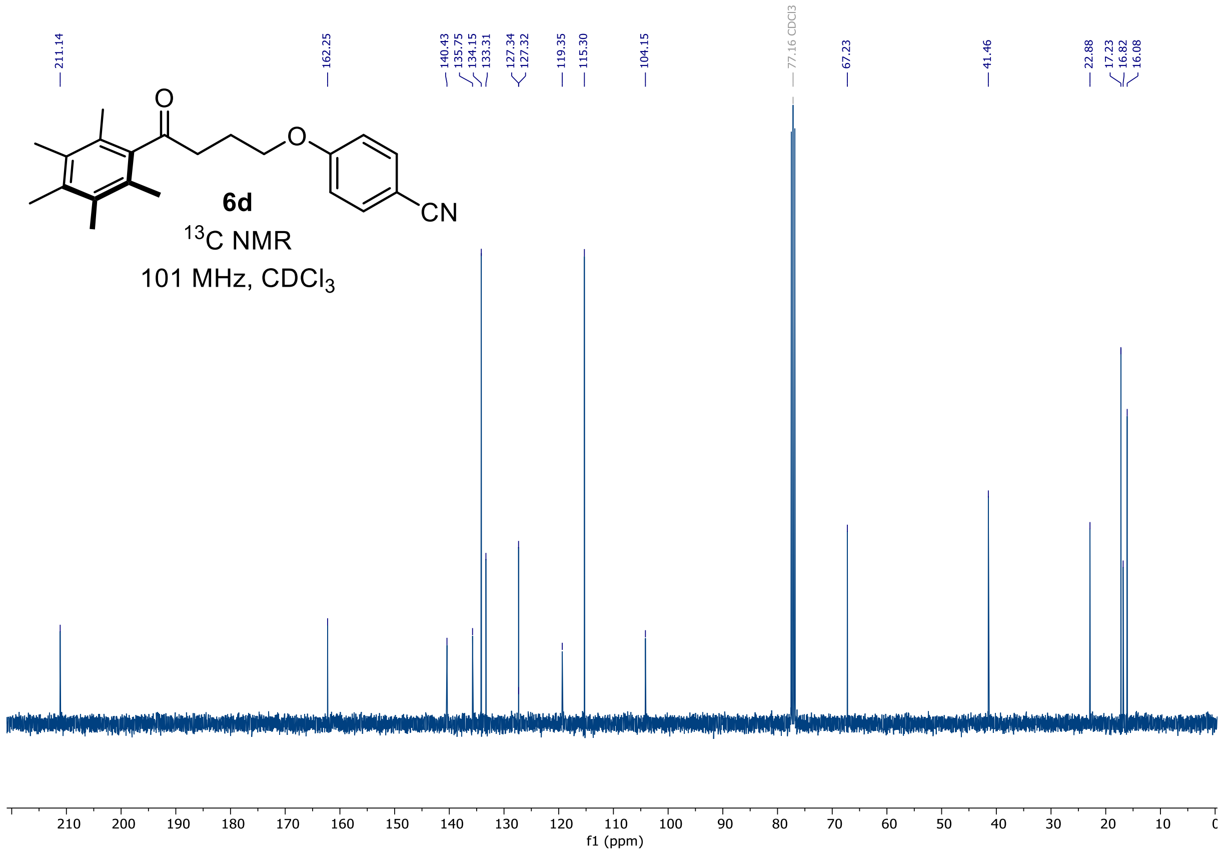

6e 1-(2,3,4,5,6-Pentamethylphenyl)-4-(4-(trifluoromethyl)phenoxy)butan-1-one

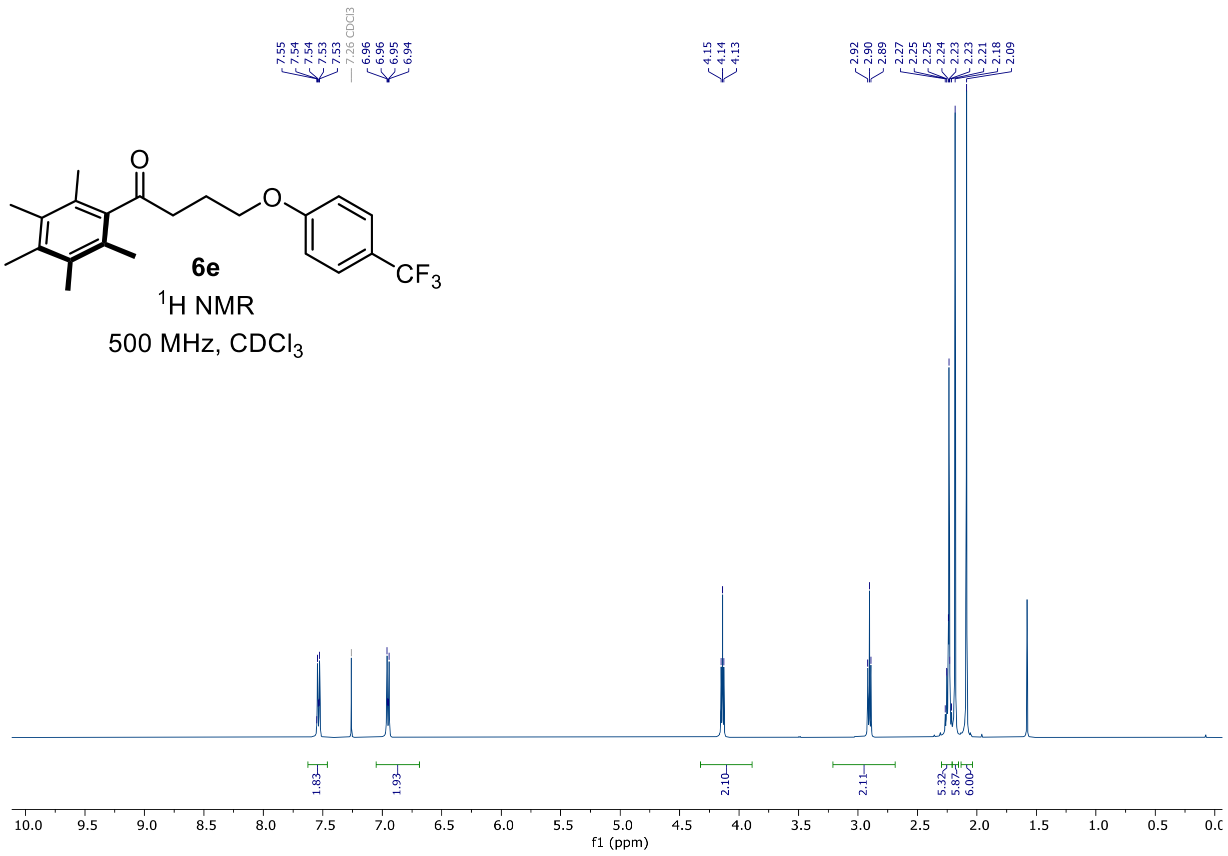

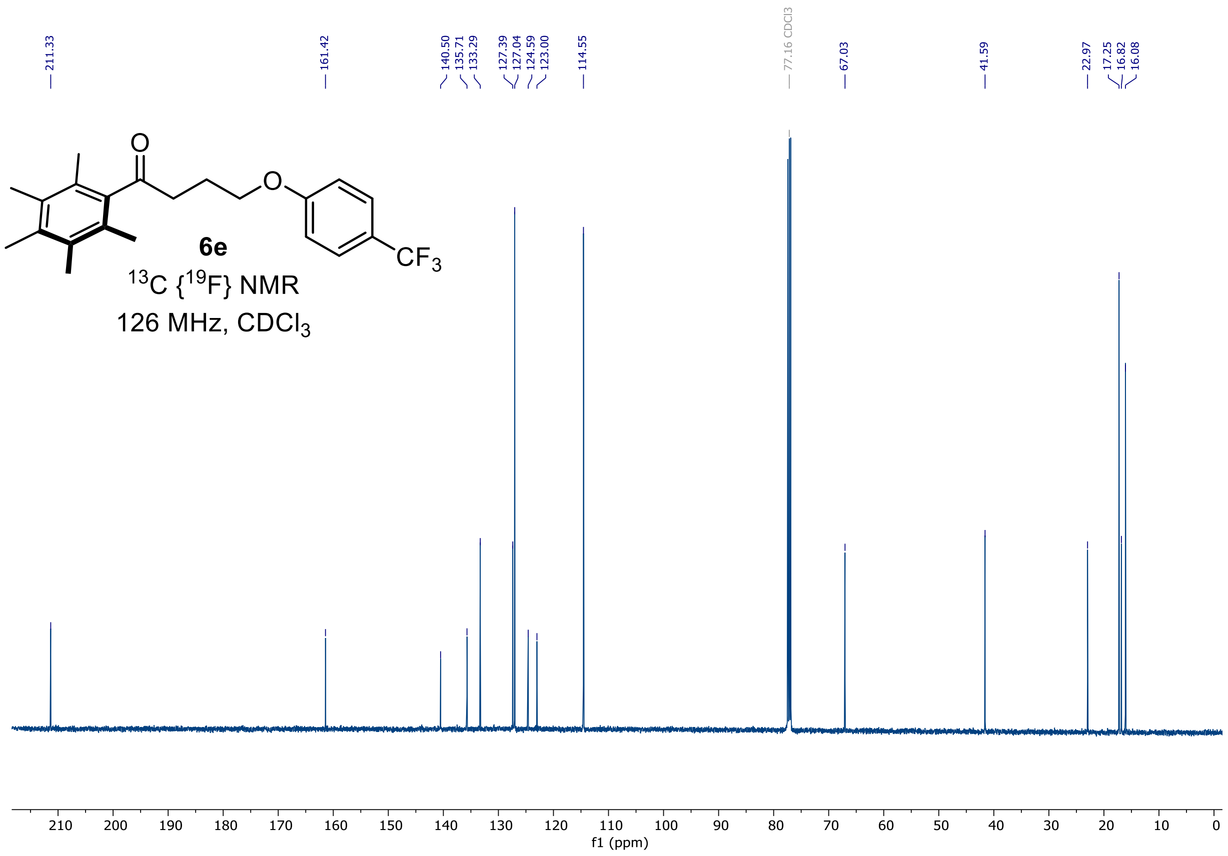
211.33
161.42
140.50
135.71
133.29
127.39
127.04
124.59
123.00
114.55
77.16 CDCl3
67.03
41.59
22.97
17.25
16.82
16.08
6e
^{13}C {^{19}F} NMR
126 MHz, $CDCl_3$
f1 (ppm)

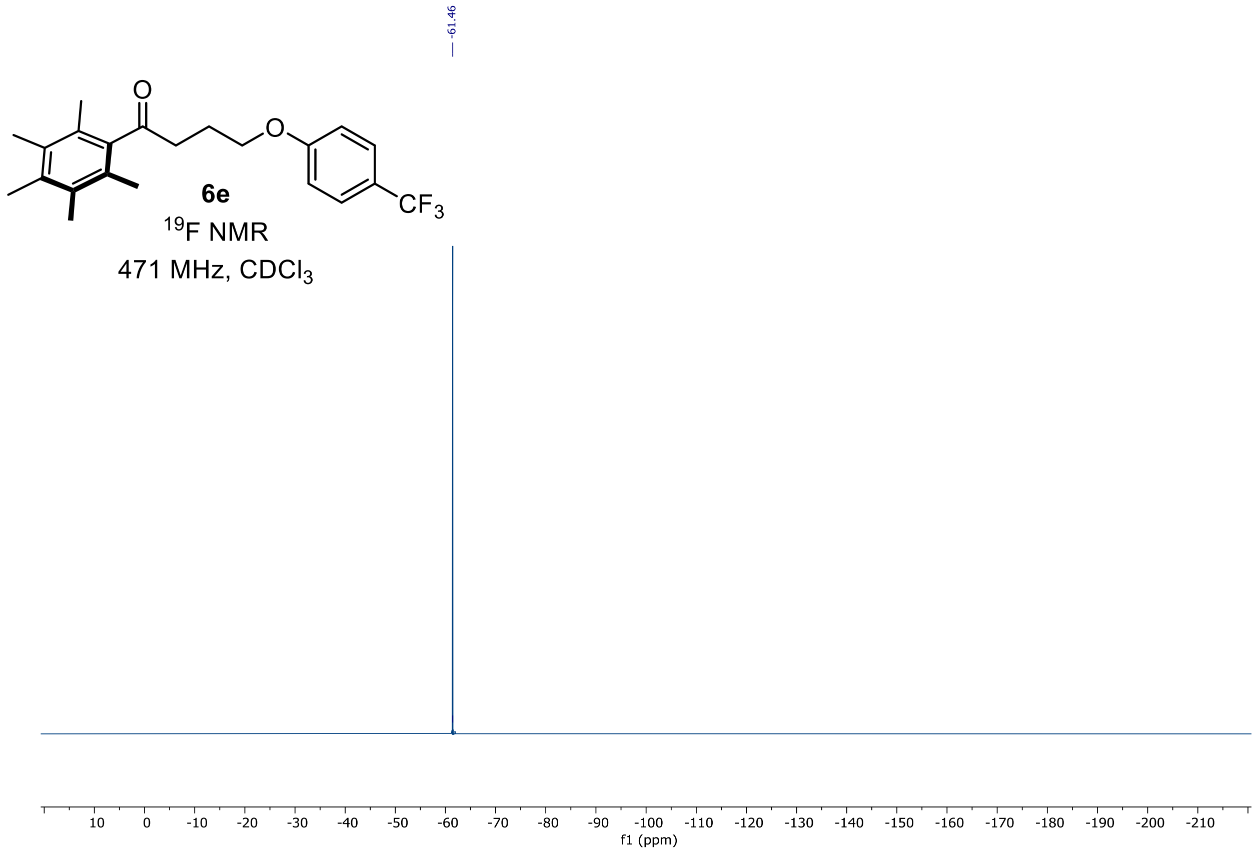
-61.46
6e
^{19}F NMR
471 MHz, $CDCl_3$
f1 (ppm)

6f 4-(4-Methoxyphenoxy)-1-(2,3,4,5,6-pentamethylphenyl)butan-1-one

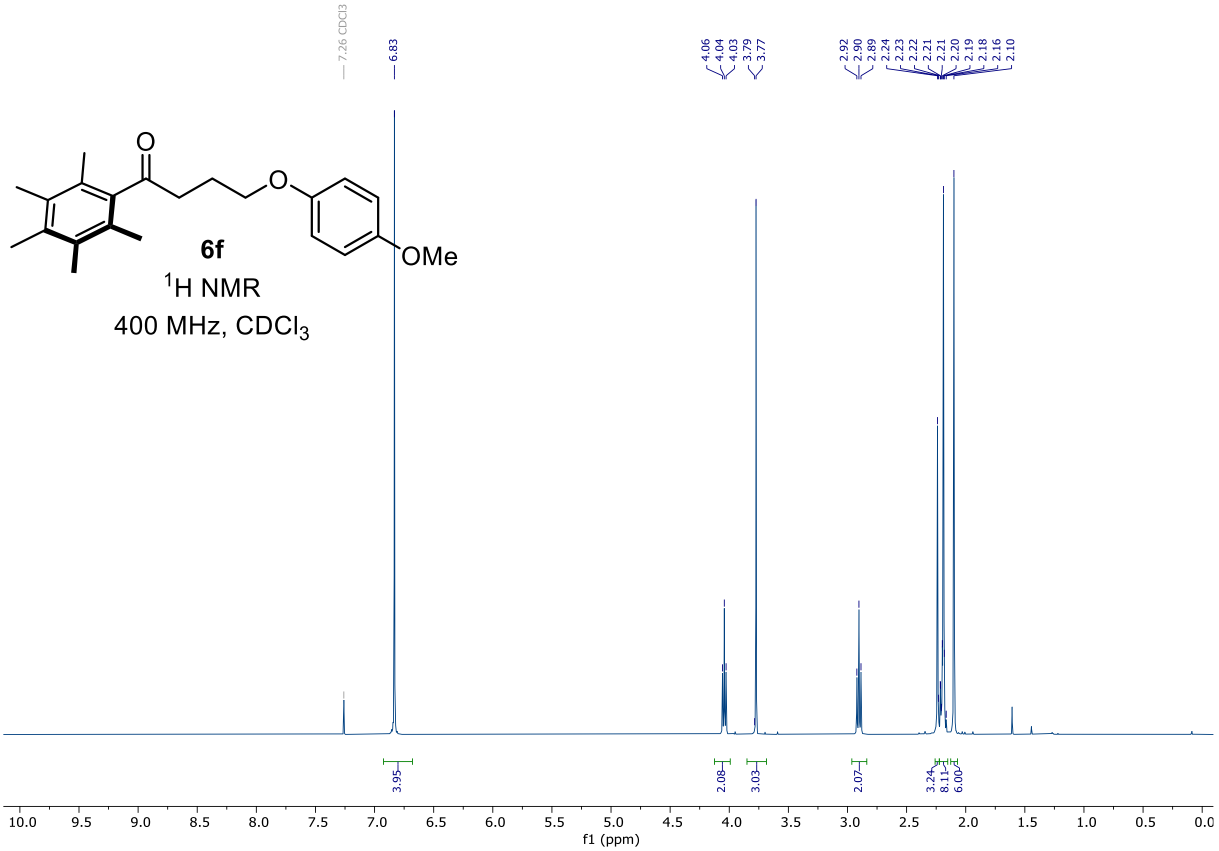

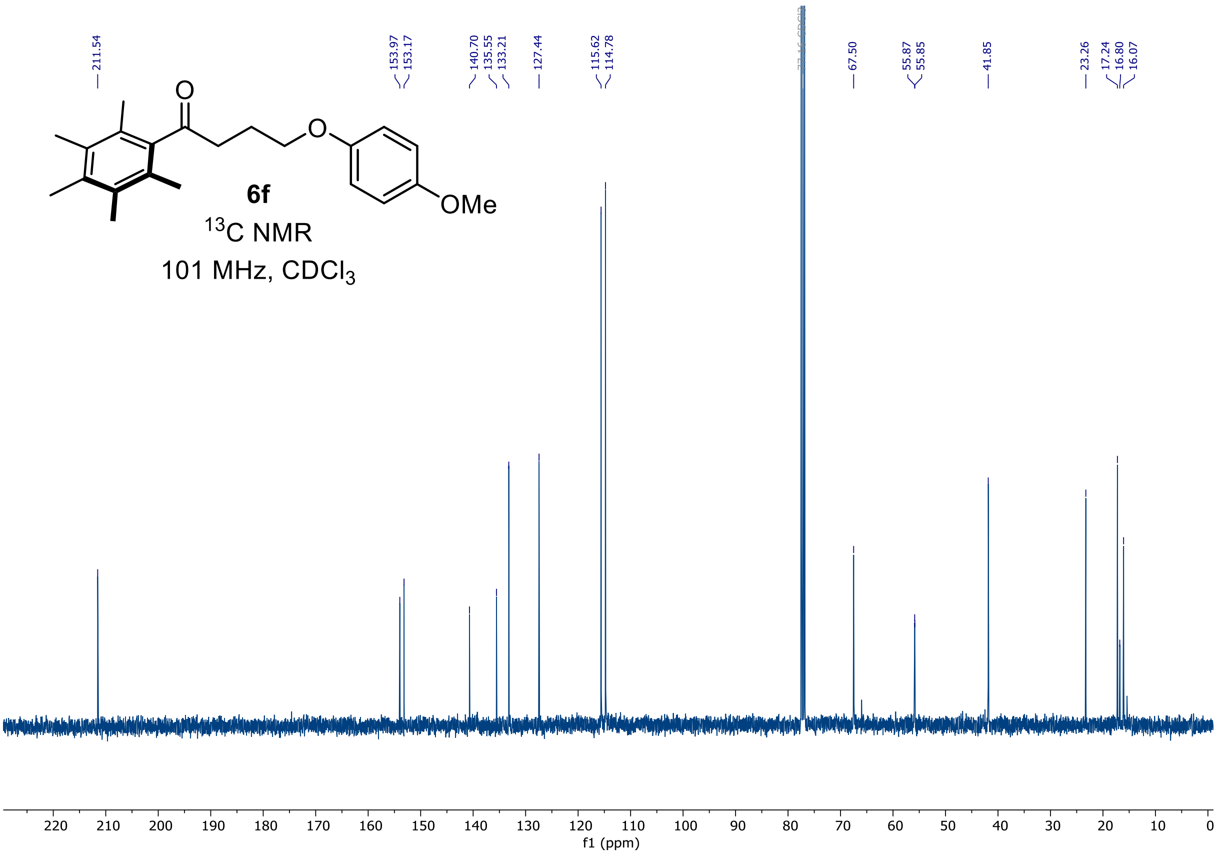

7 4-(4-Methoxyphenoxy)-1-(2,3,5,6-tetramethylphenyl)butan-1-one

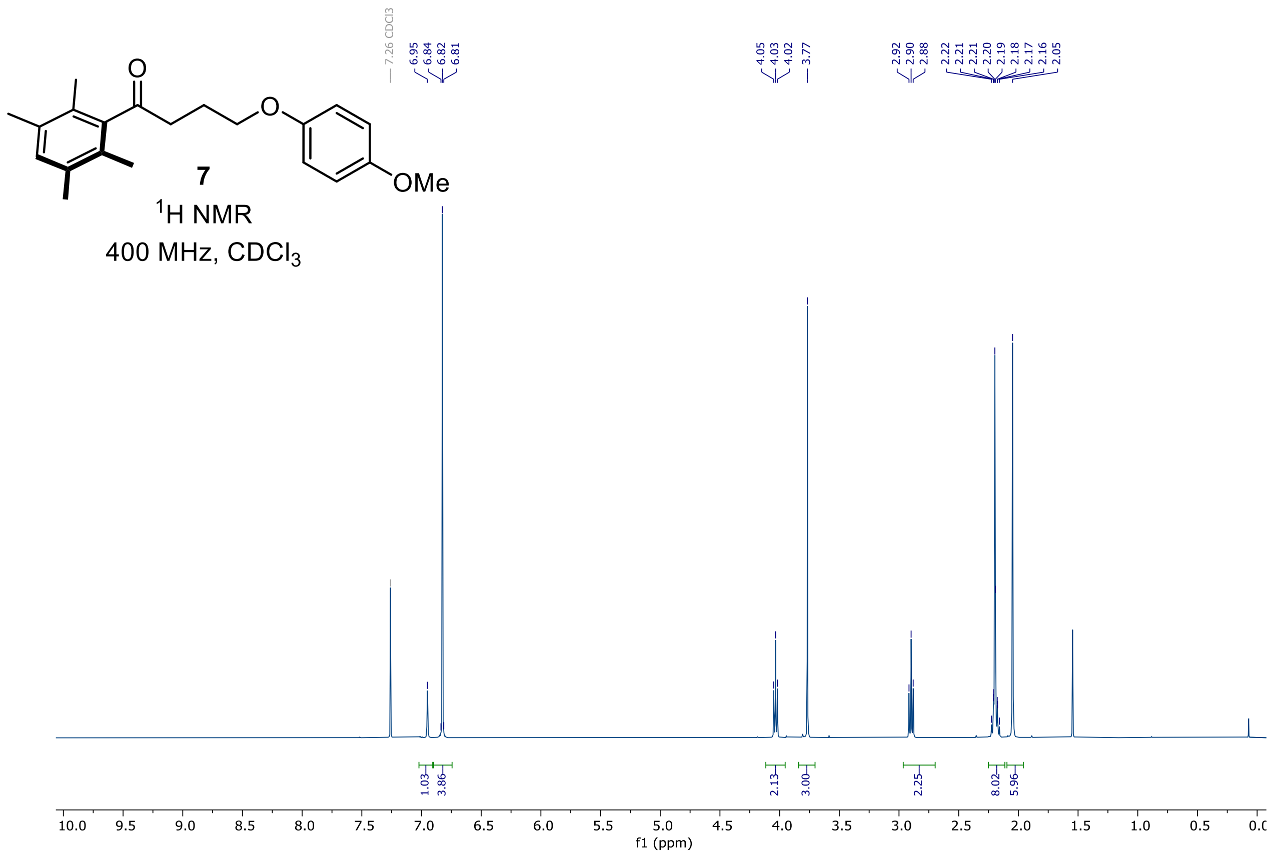

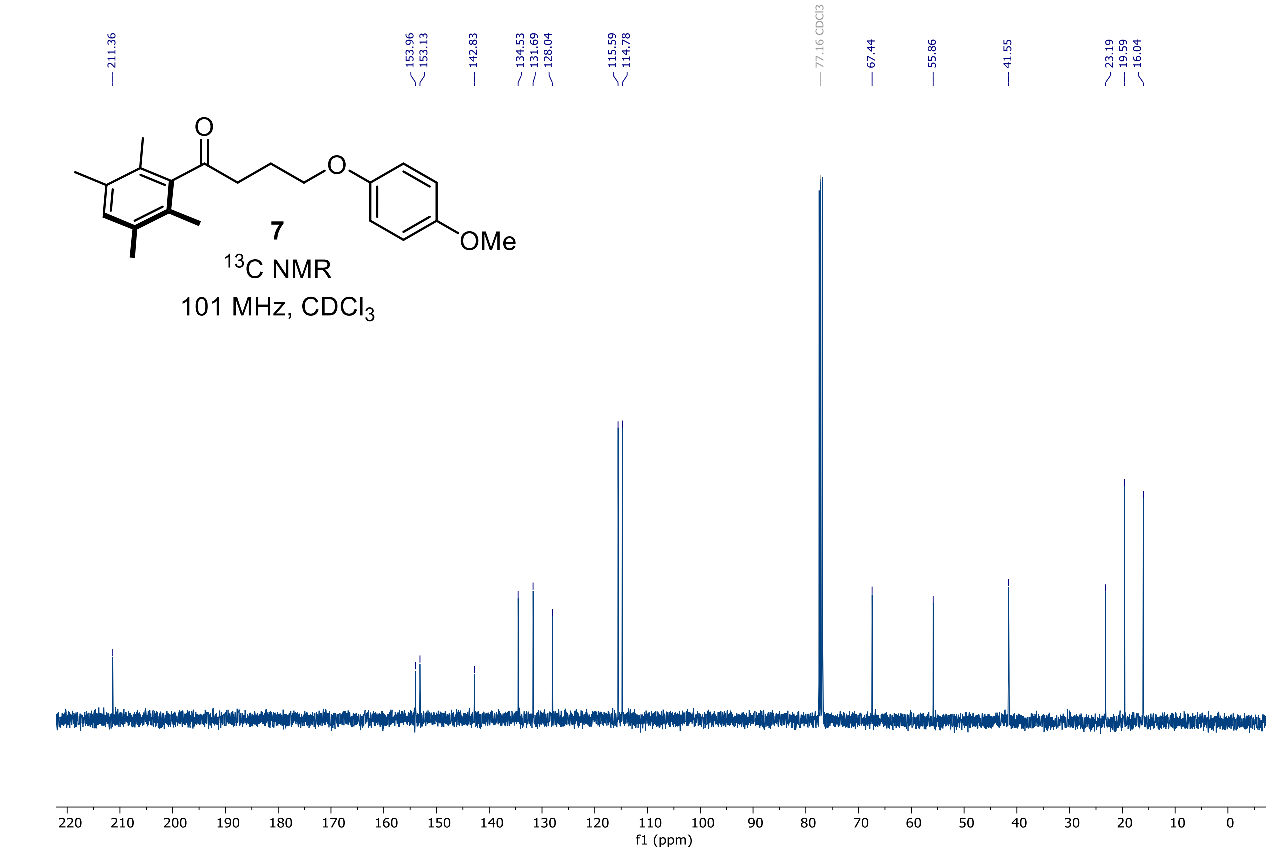

8a (1-Benzylcyclopropyl)(2,3,5,6-tetramethylphenyl)methanone

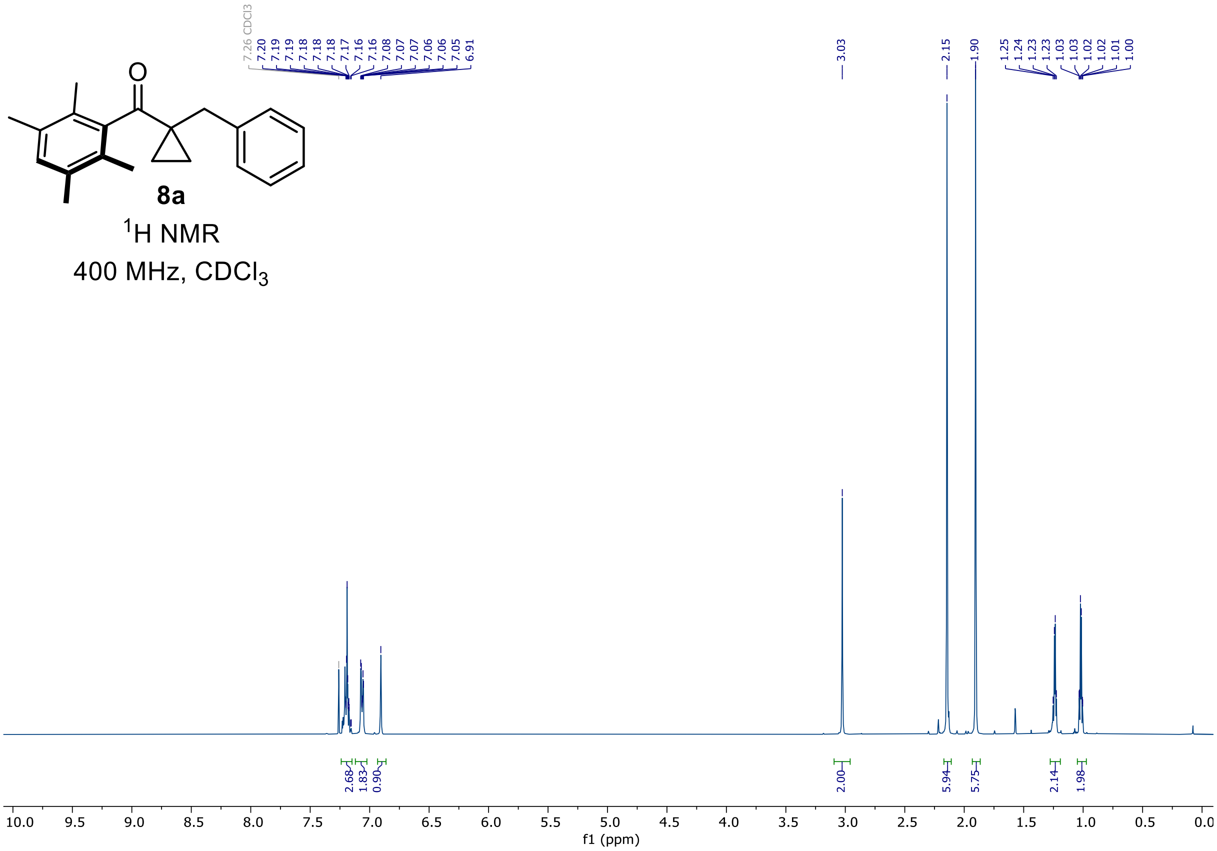

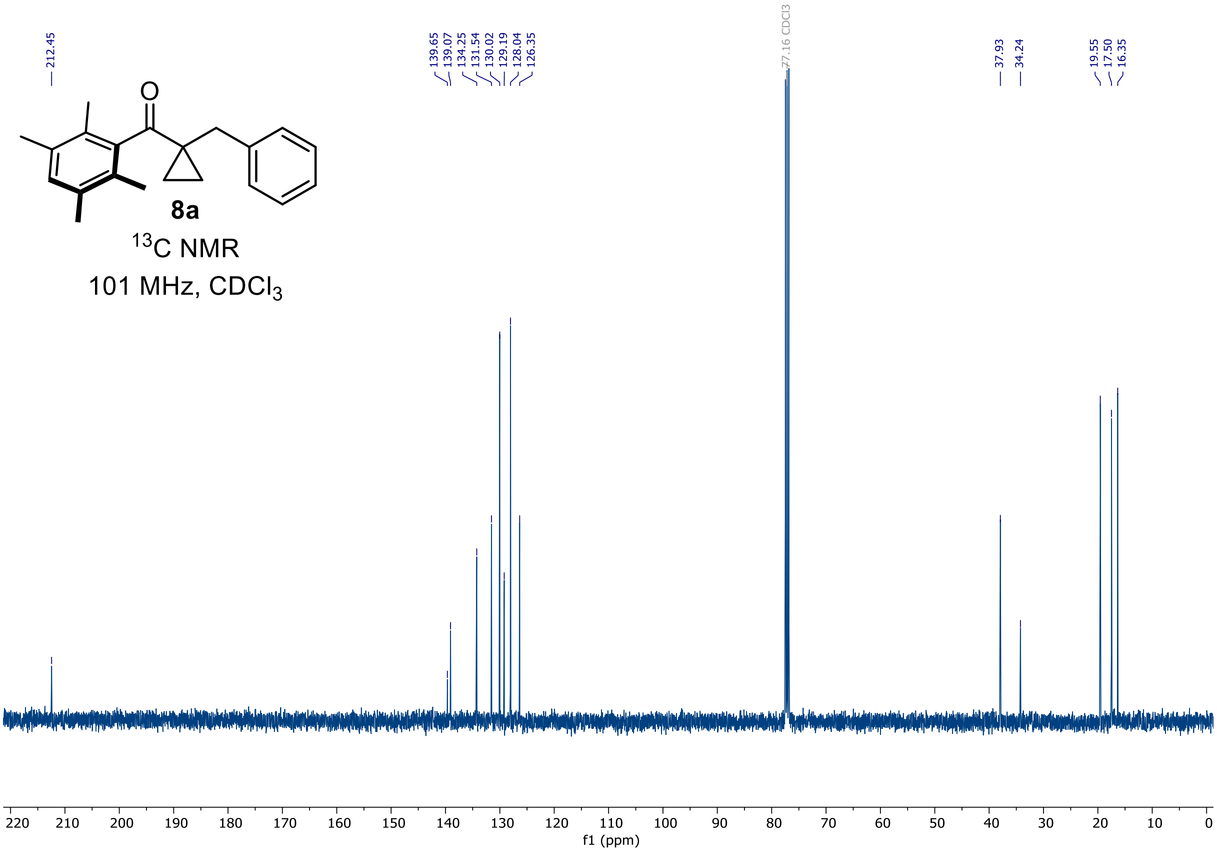

8b (1-(4-Methoxybenzyl)cyclopropyl)(2,3,5,6-tetramethylphenyl)methanone

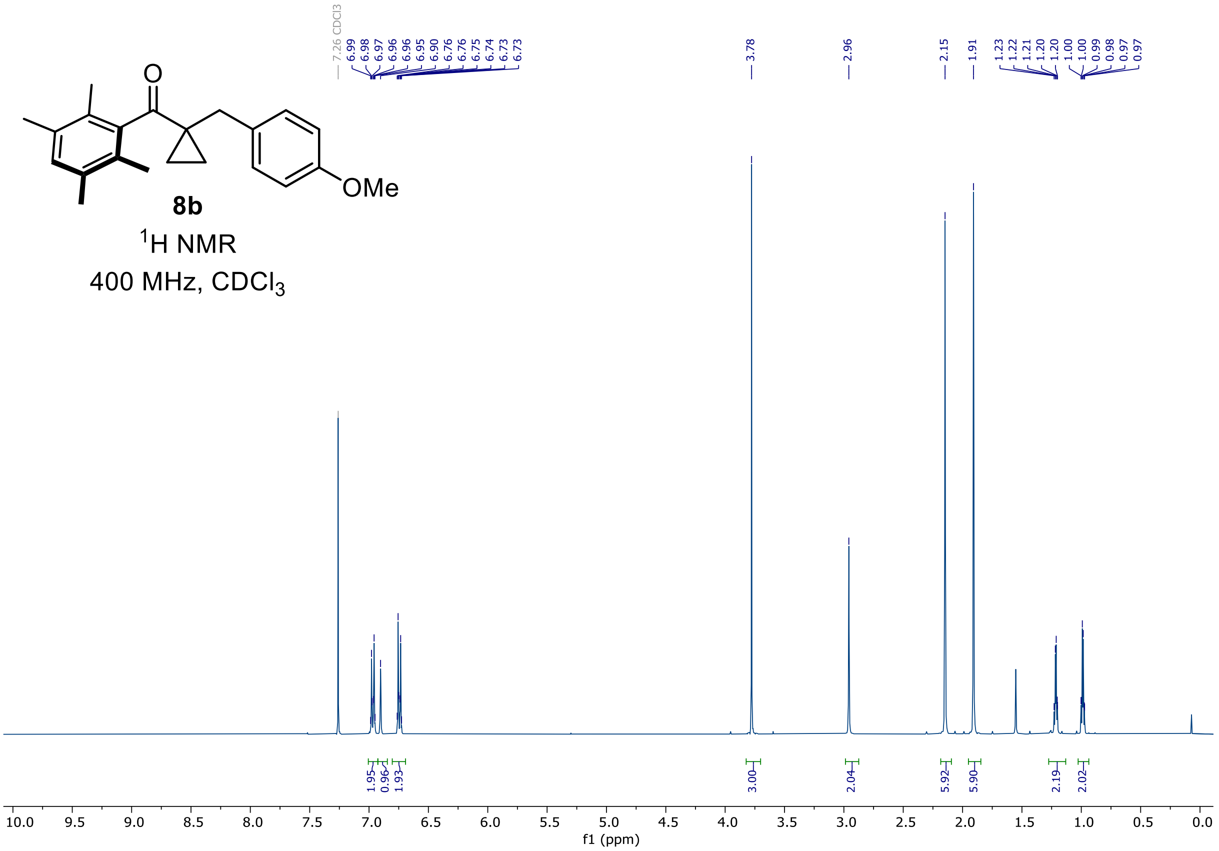

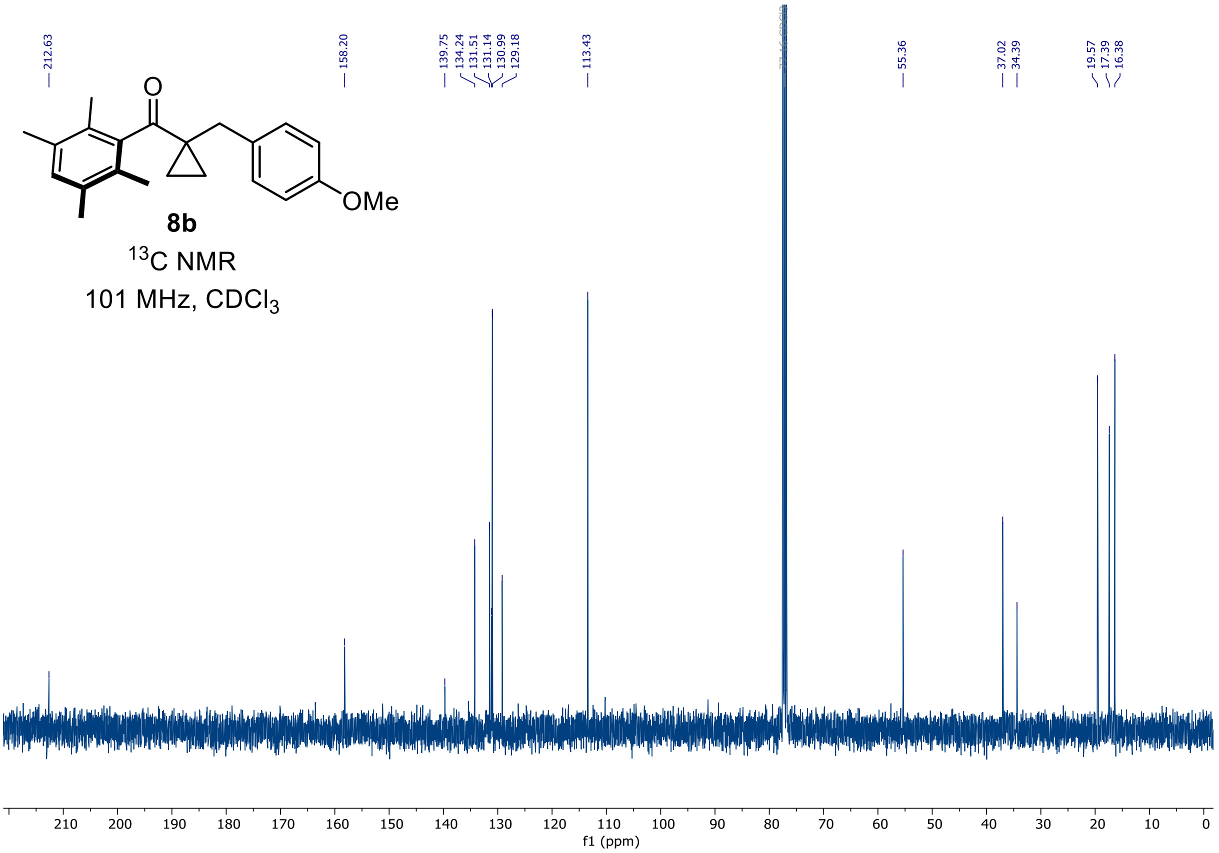

8c (2,3,5,6-Tetramethylphenyl)(1-(3-(trifluoromethyl)benzyl)cyclopropyl)methanone

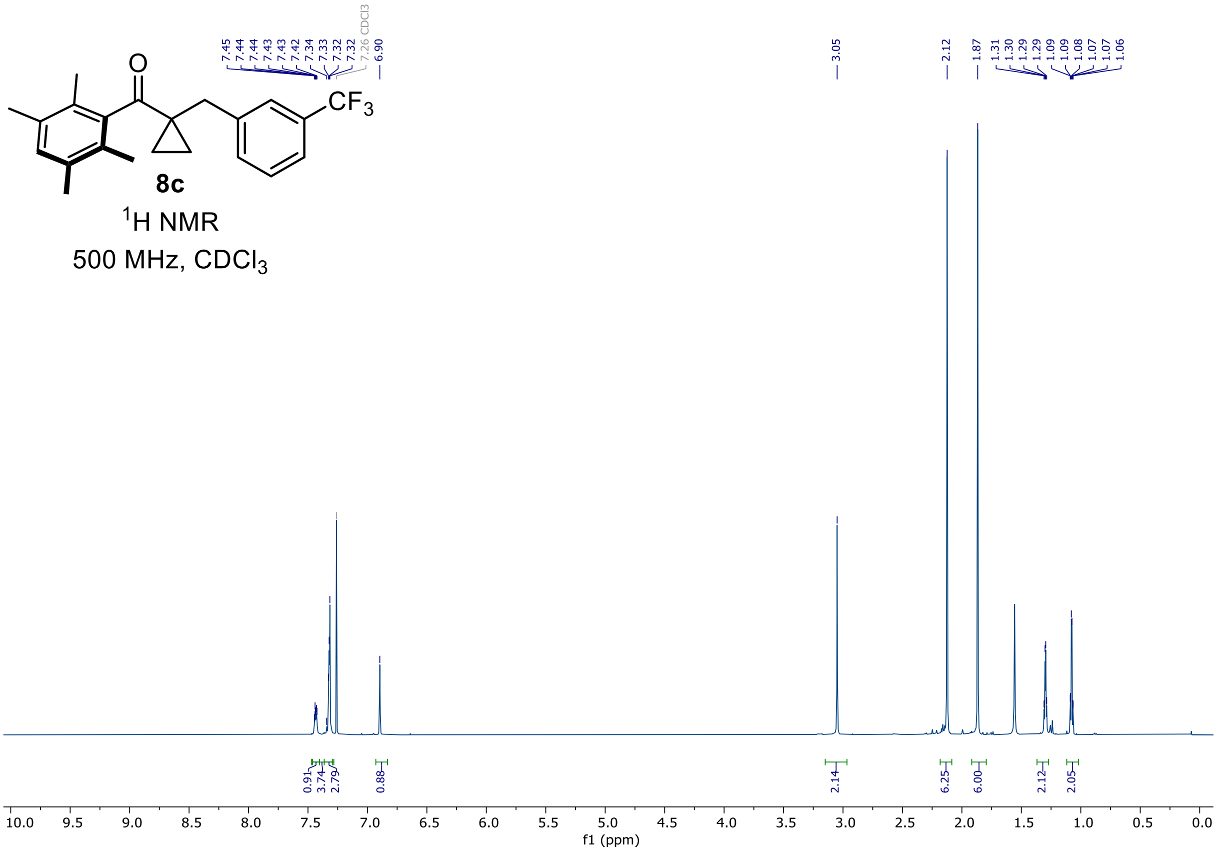

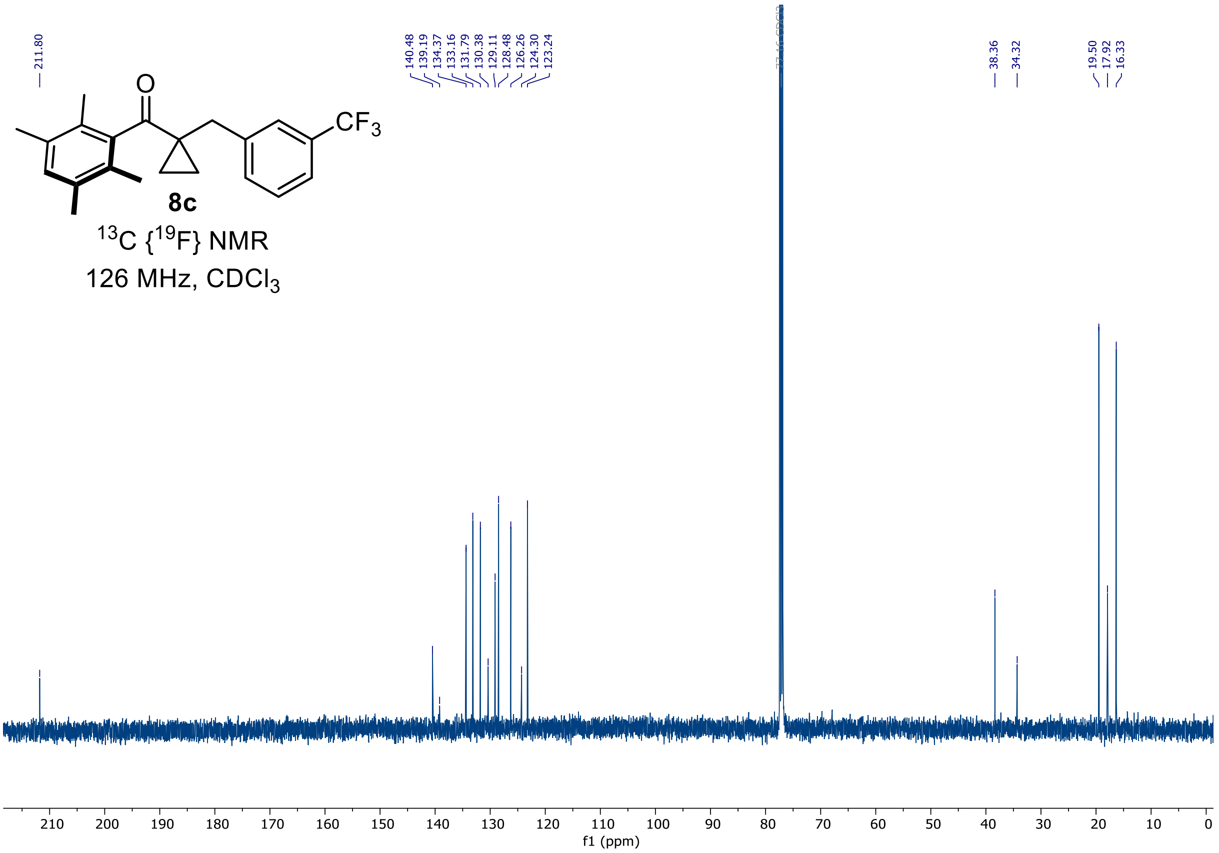

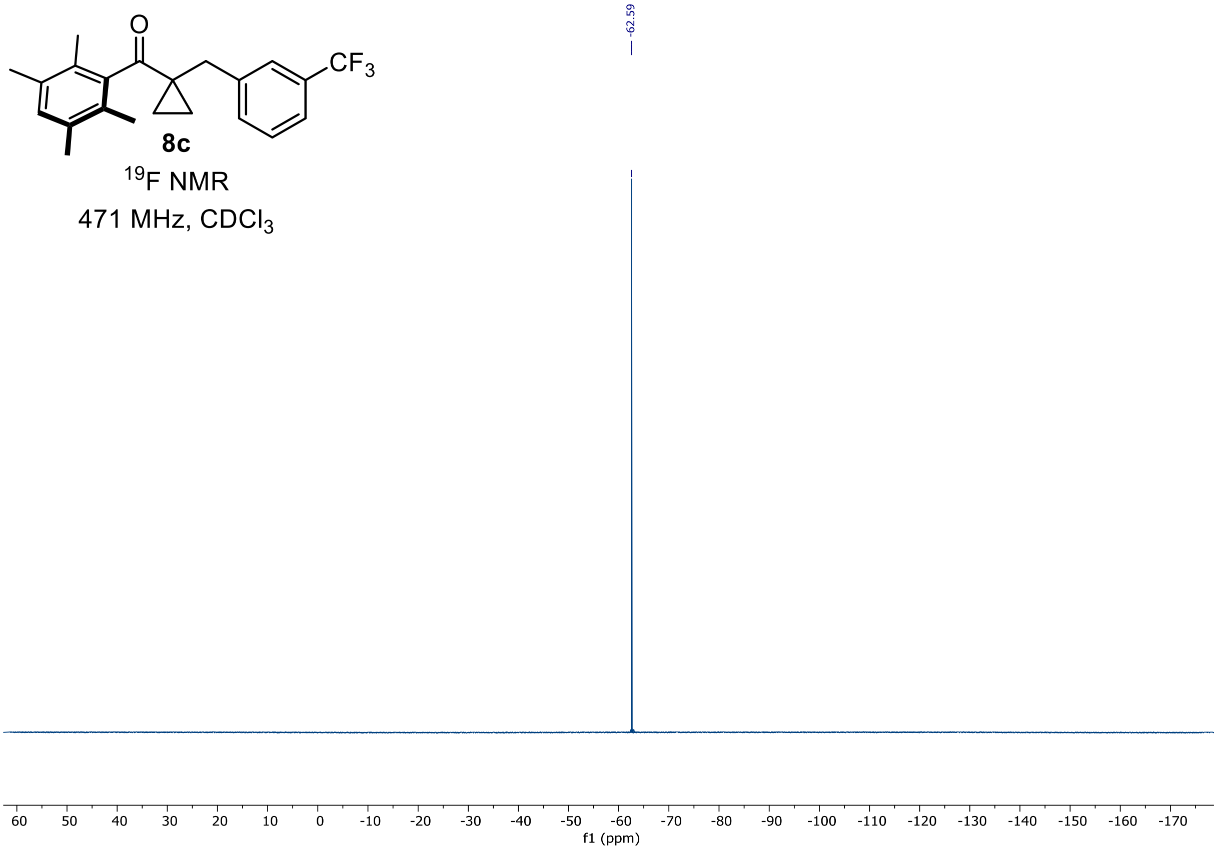

8c

^{19}F NMR

471 MHz, $CDCl_3$

8d (1-Butylcyclopropyl)(2,3,5,6-tetramethylphenyl)methanone

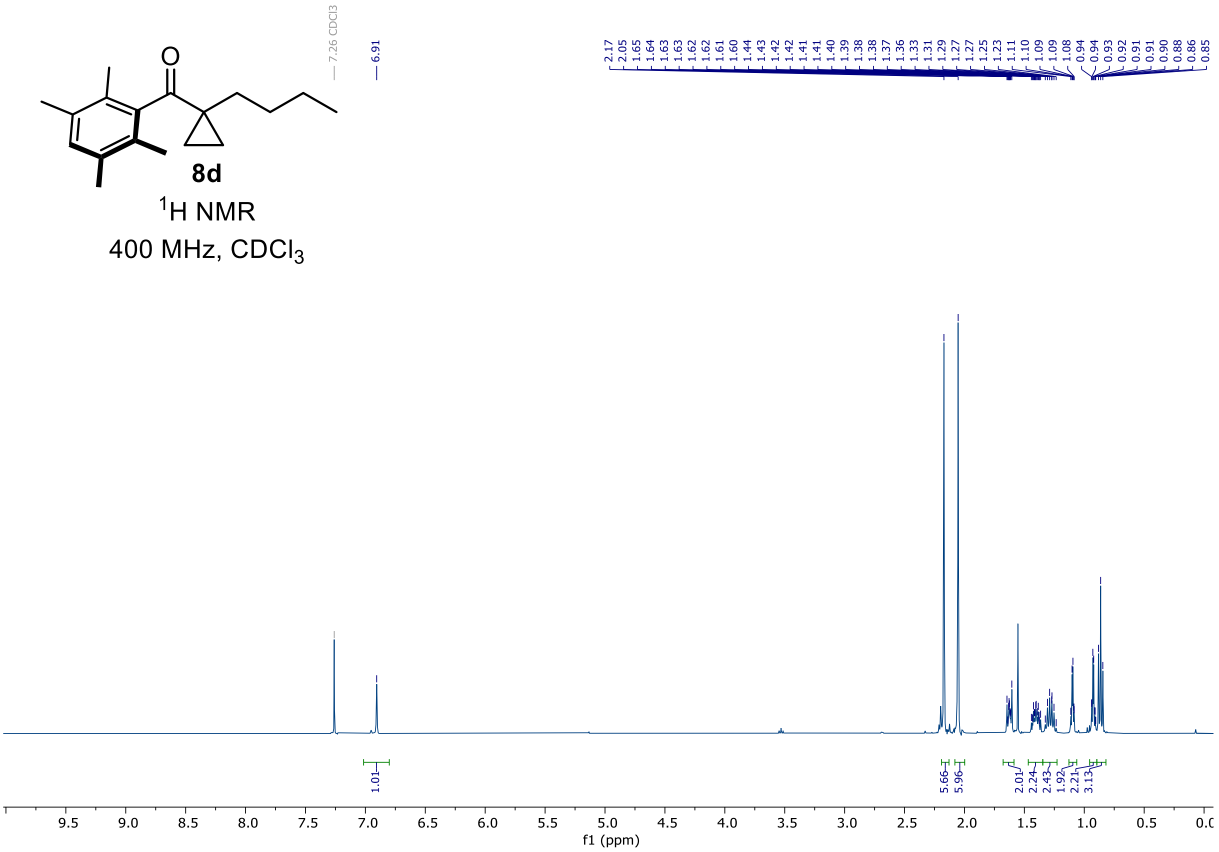

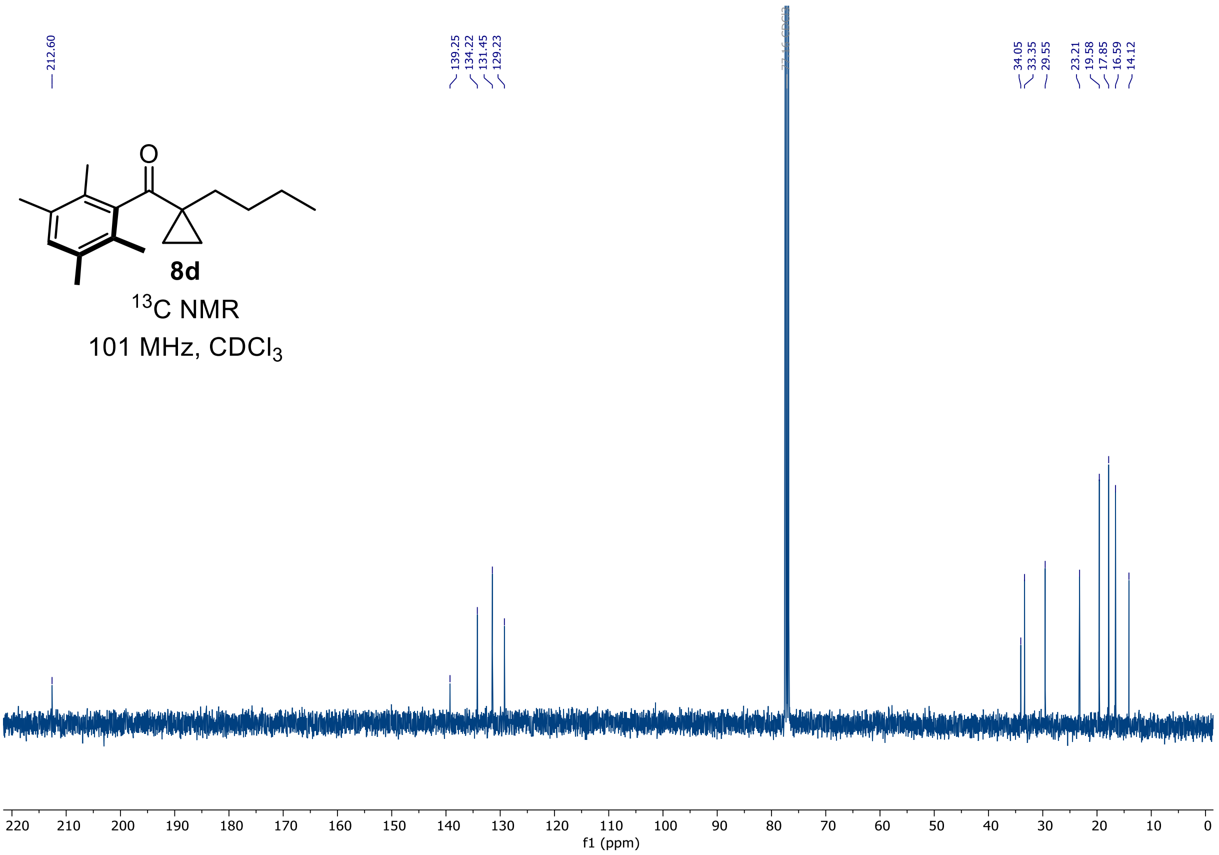

8e (1-(Cyclopropylmethyl)cyclopropyl)(2,3,5,6-tetramethylphenyl)methanone

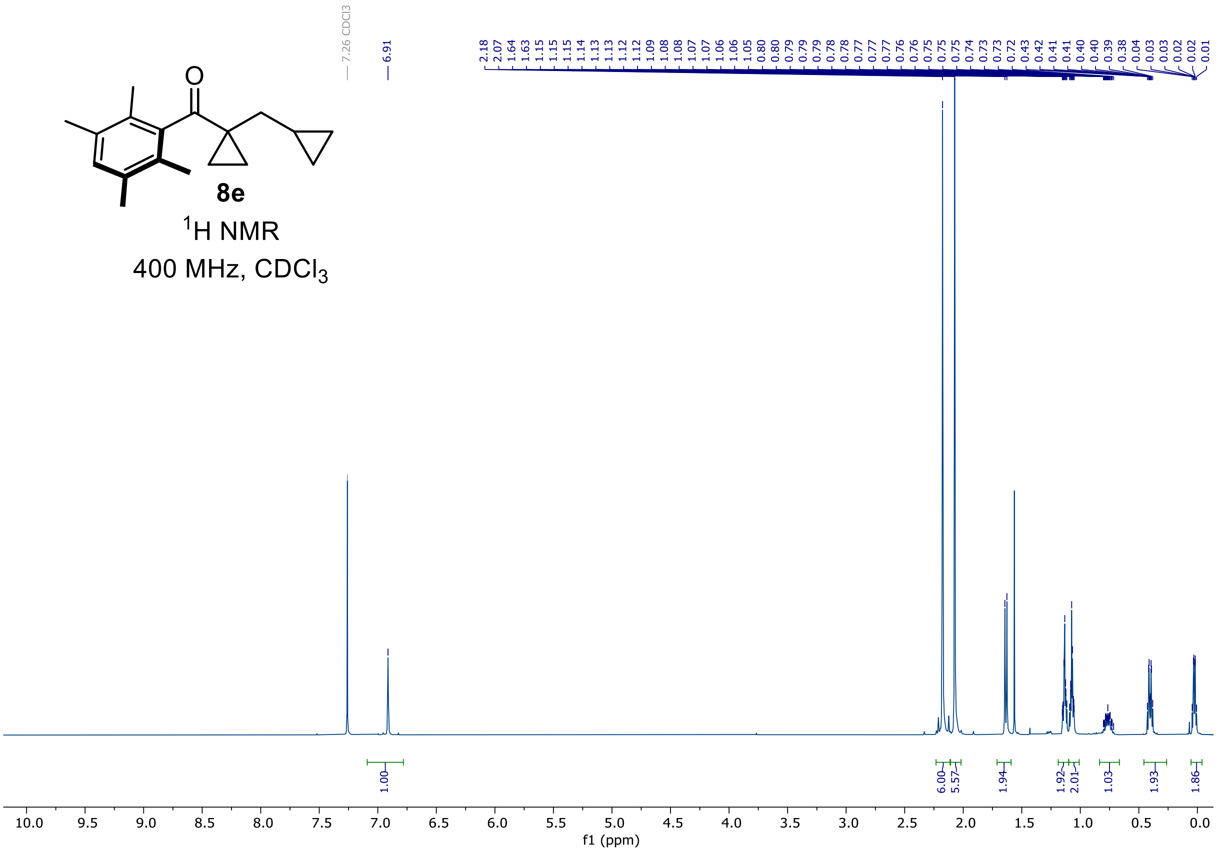

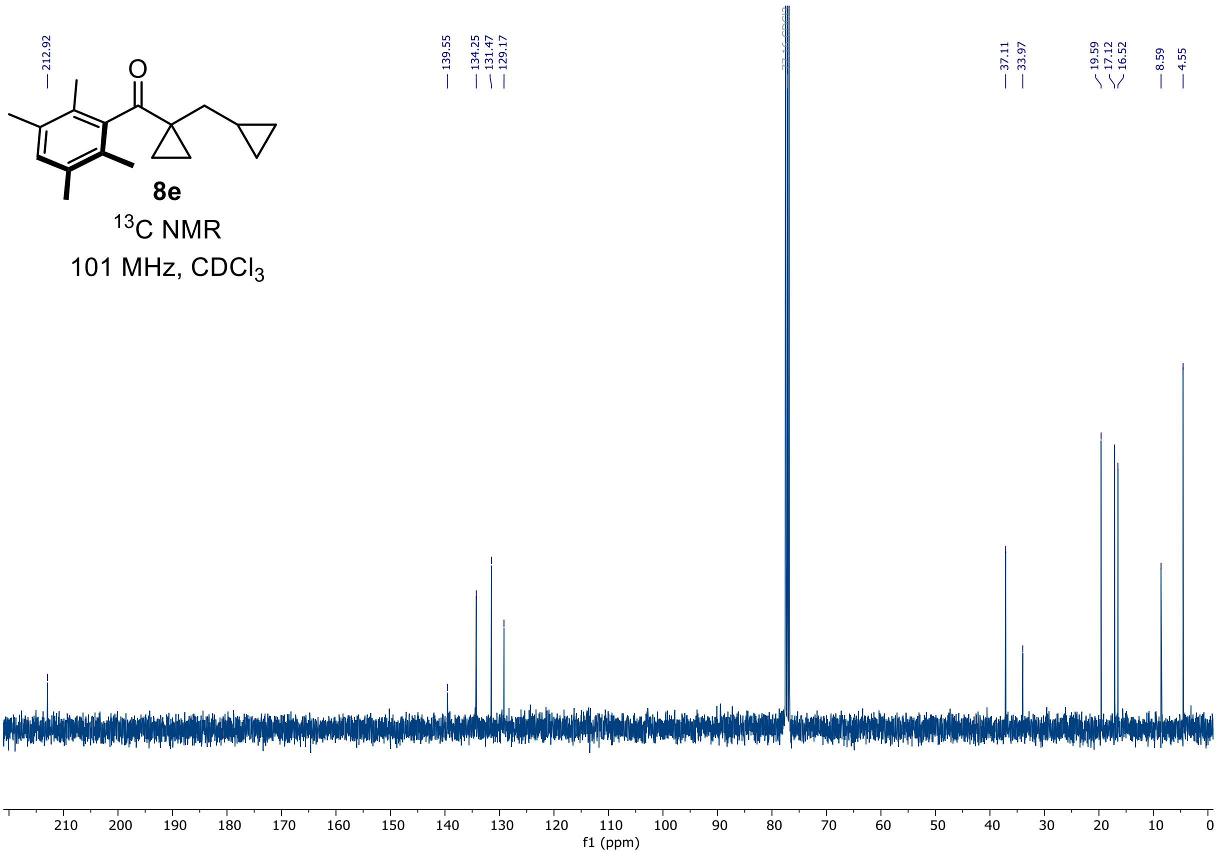

8f (1-Isopentylcyclopropyl)(2,3,5,6-tetramethylphenyl)methanone

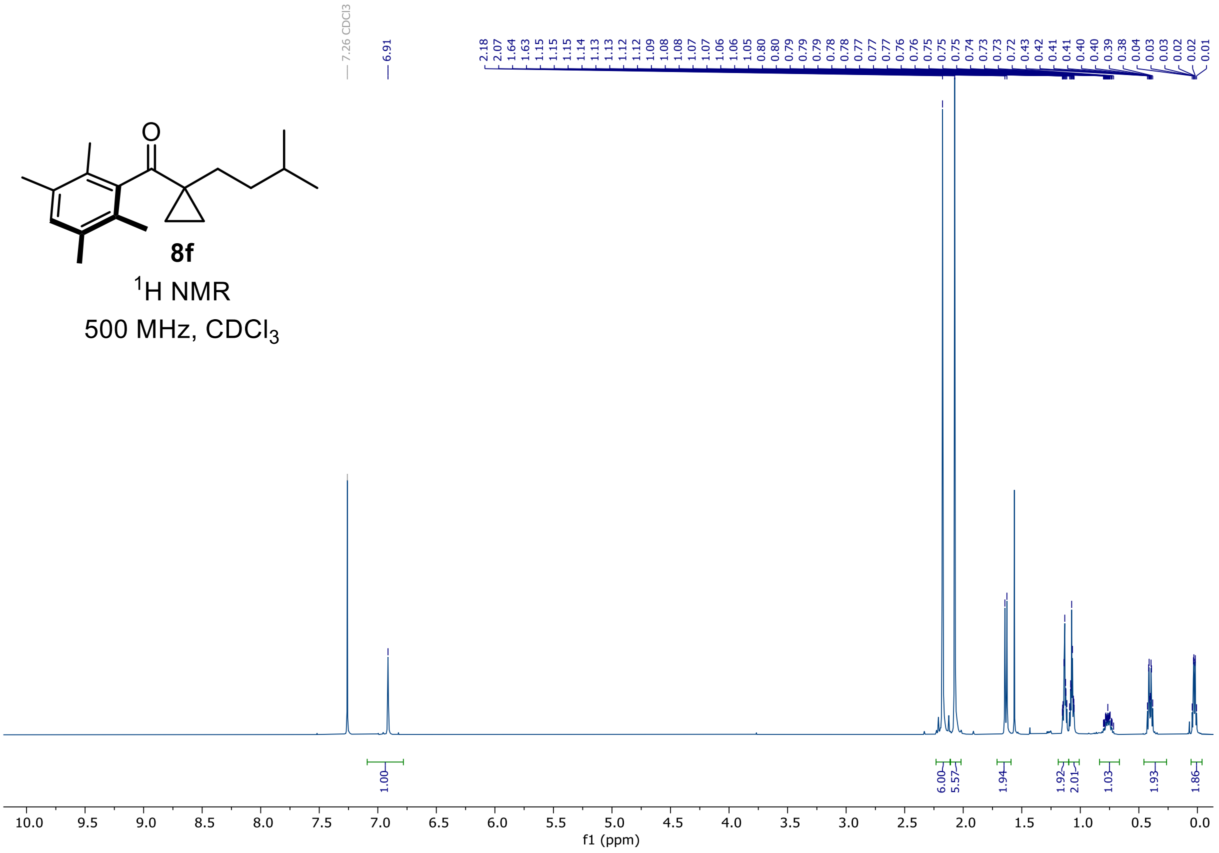

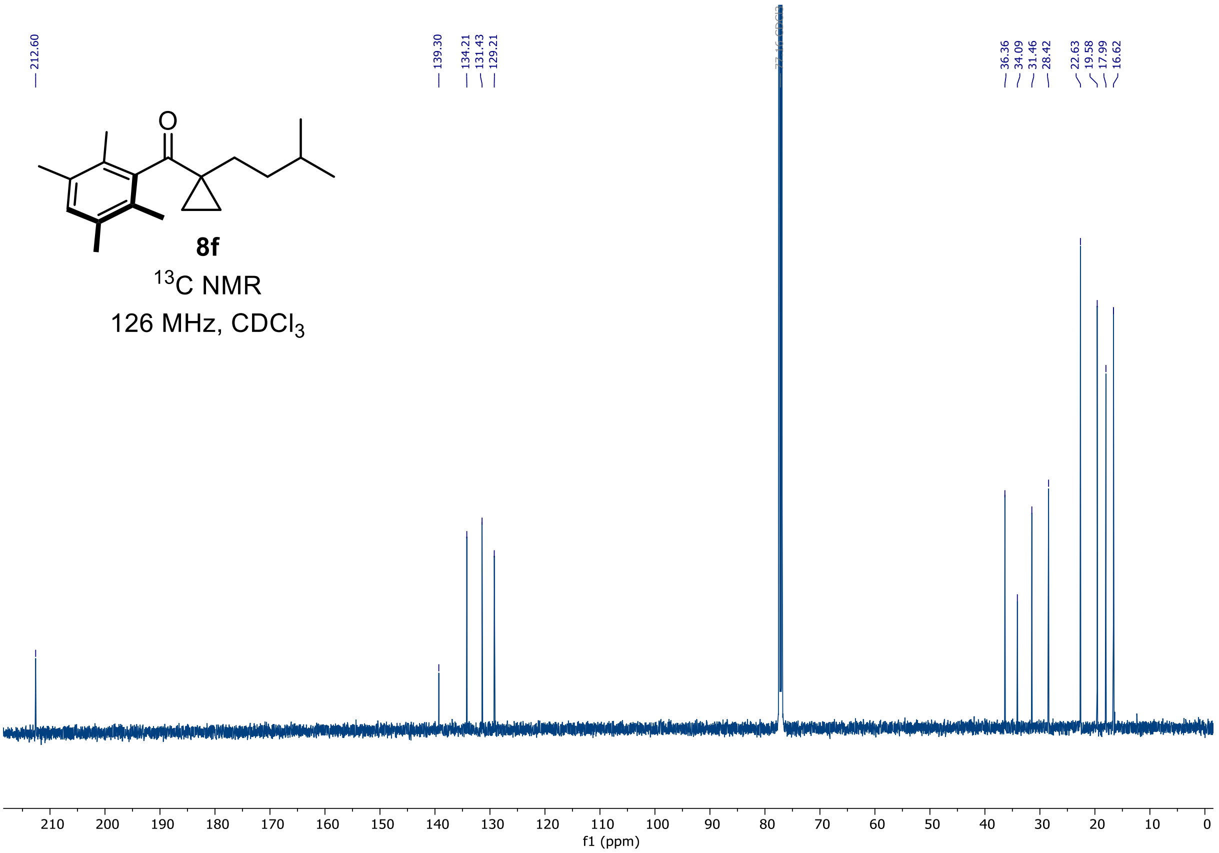

8g (1-(4-(Benzyloxy)butyl)cyclopropyl)(2,3,5,6-tetramethylphenyl)methanone

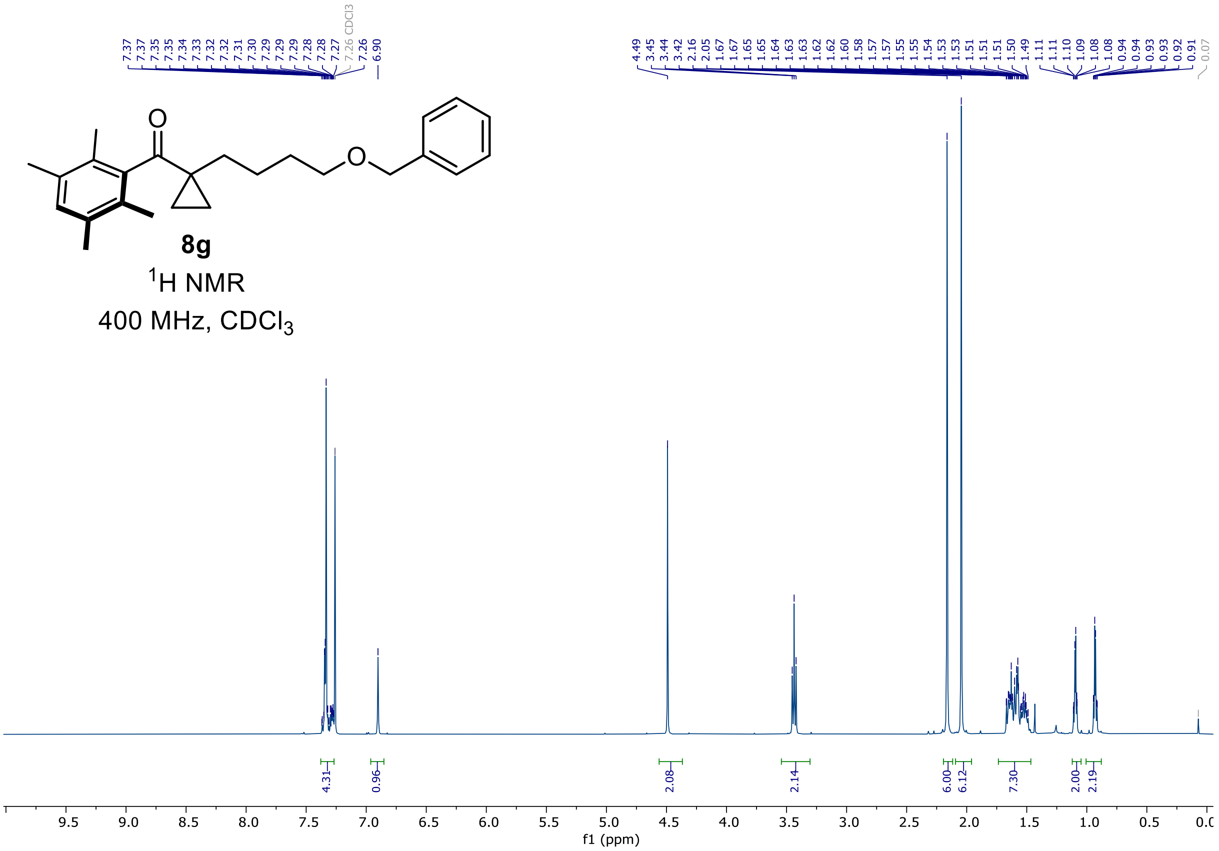

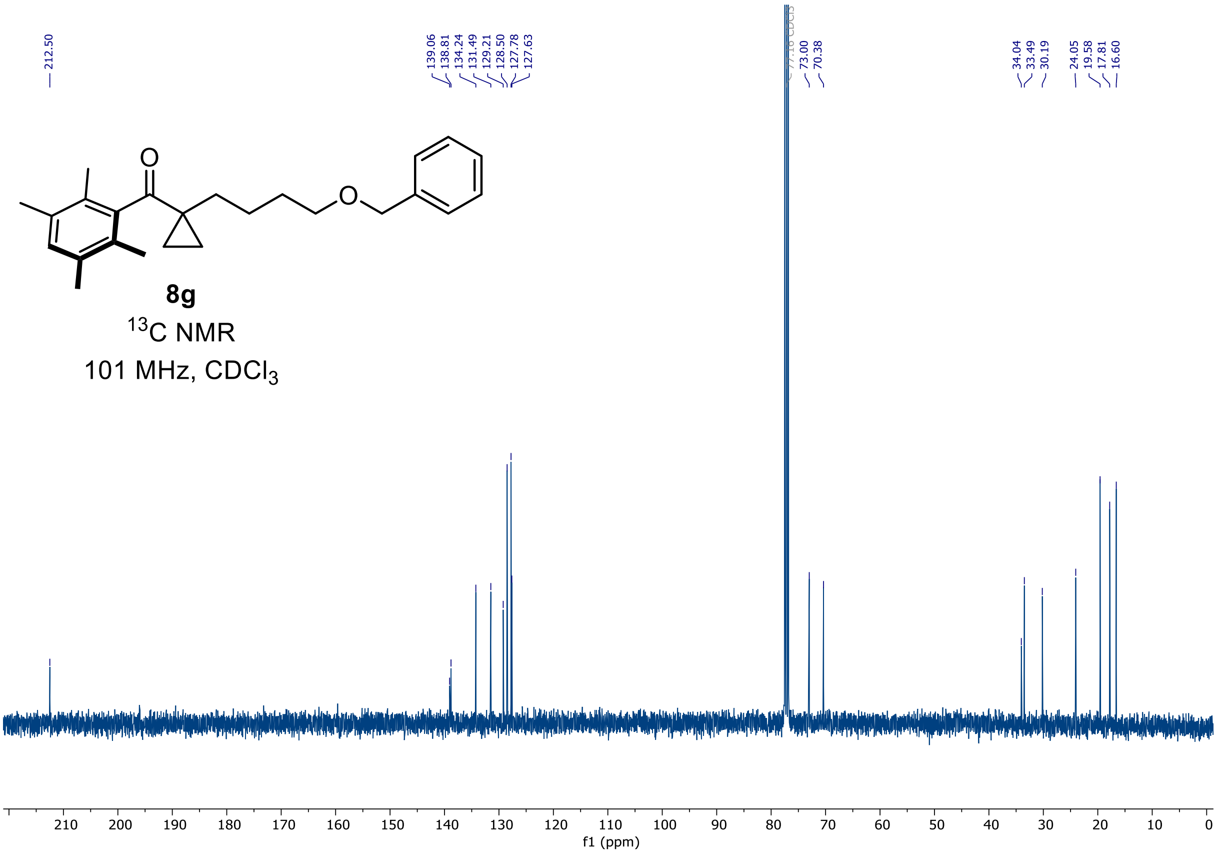

8h (1-((1-Benzylpiperidin-4-yl)methyl)cyclopropyl)(2,3,5,6-tetramethylphenyl)methanone

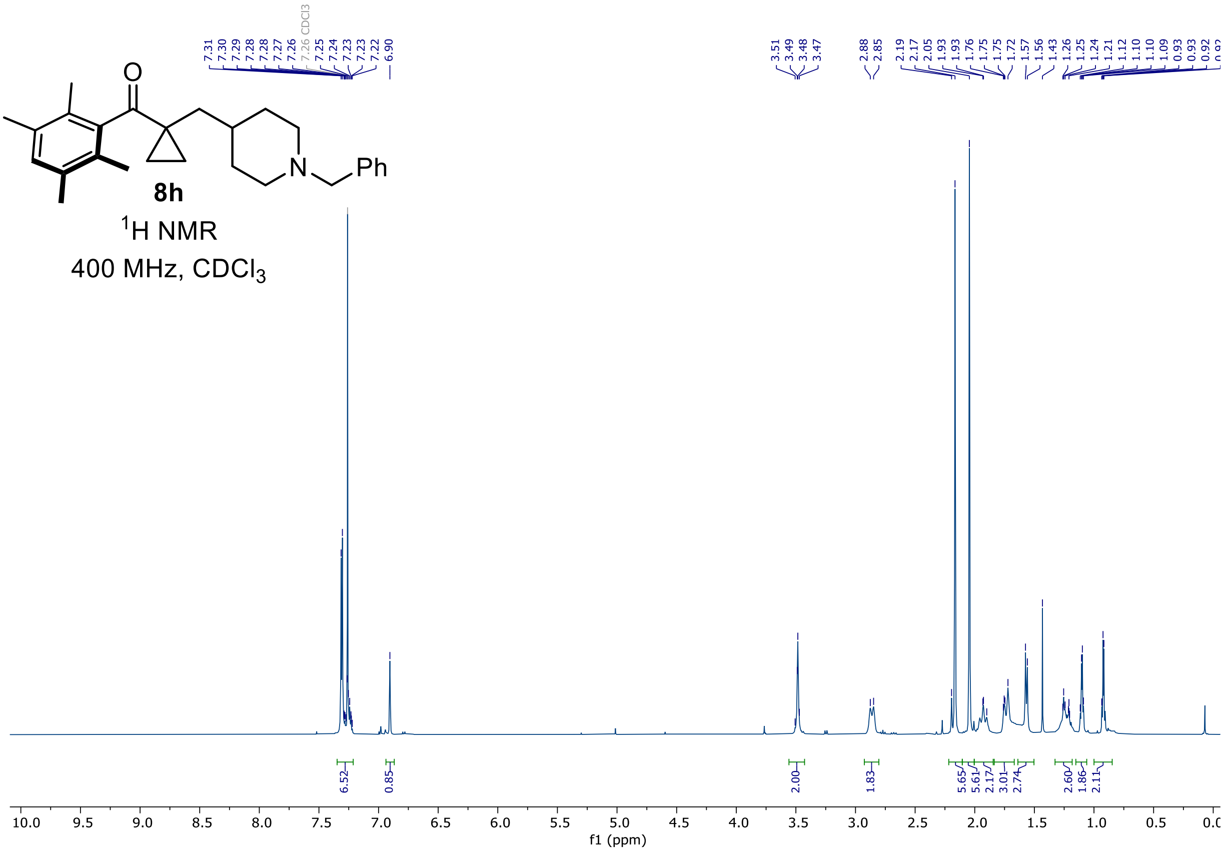

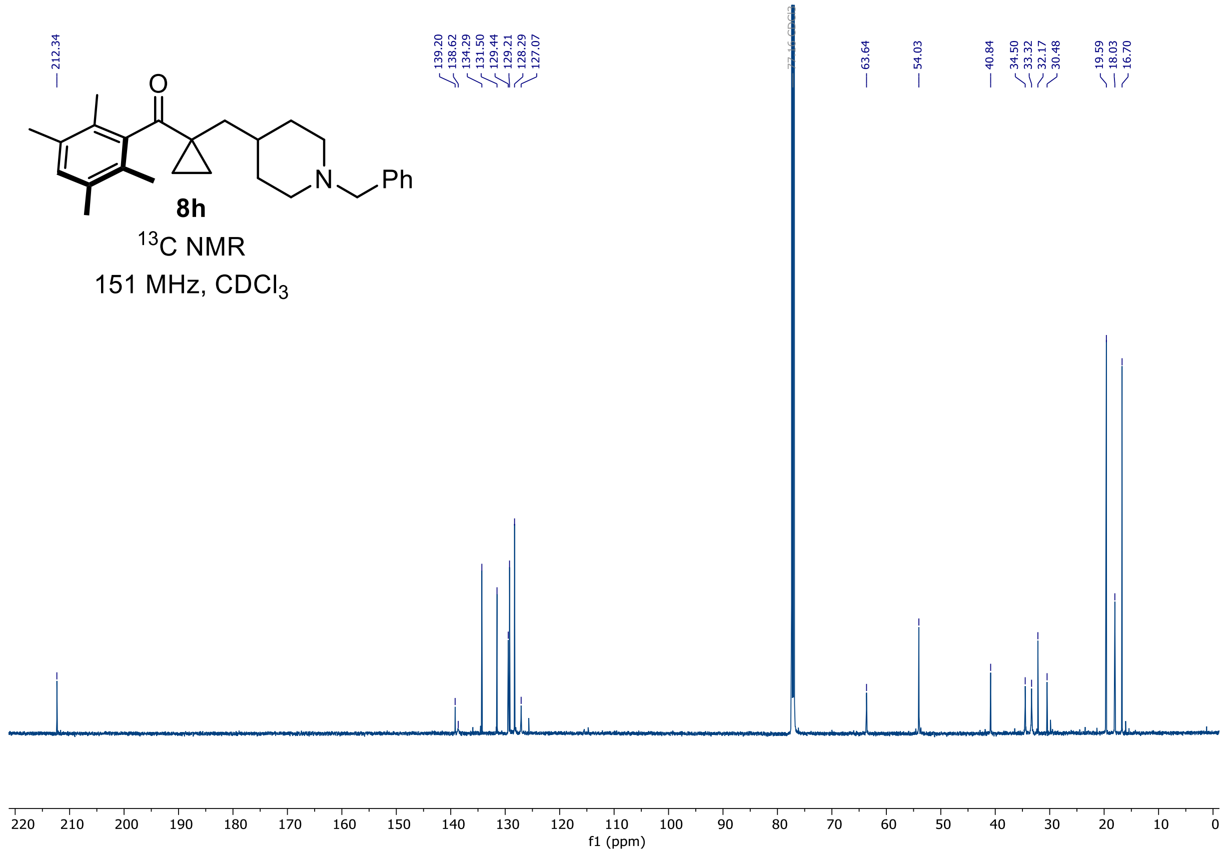

8i (1-(4-(Methylthio)butyl)cyclopropyl)(2,3,5,6-tetramethylphenyl)methanone

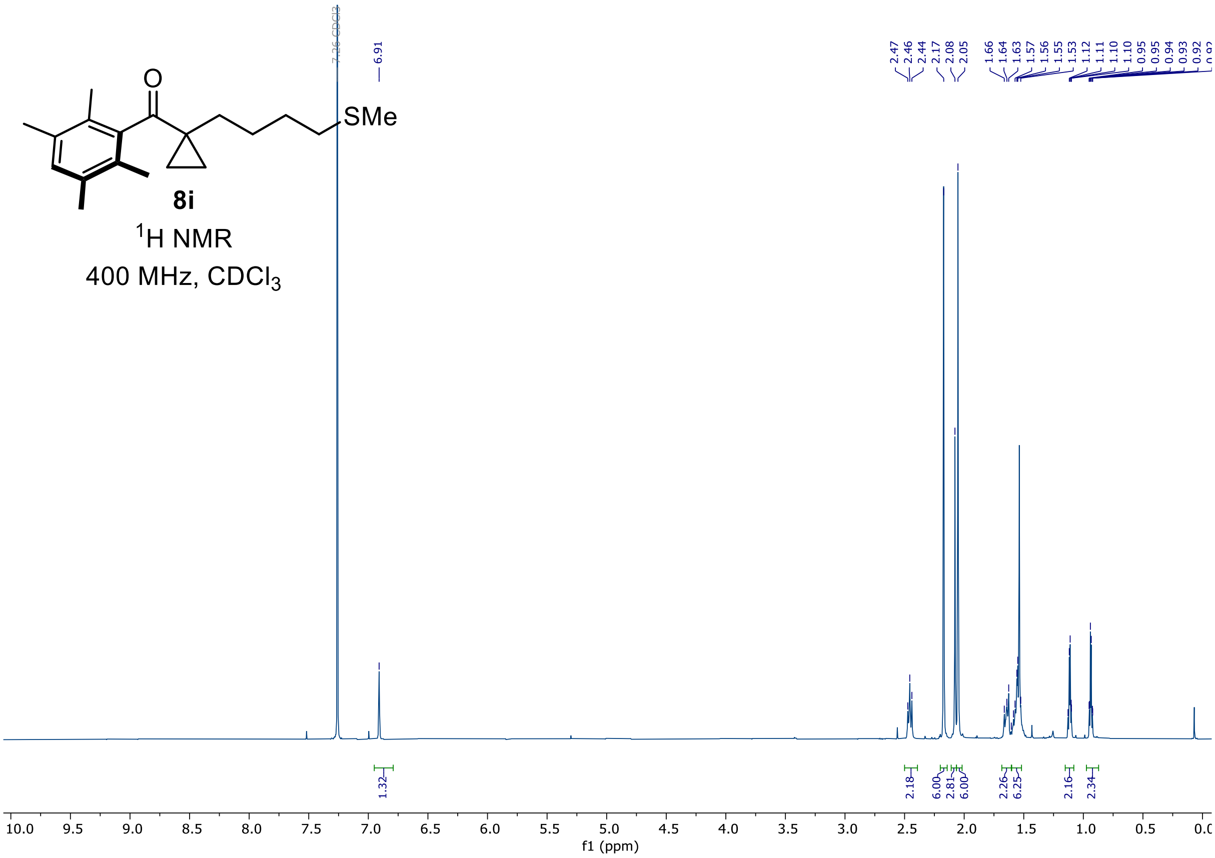

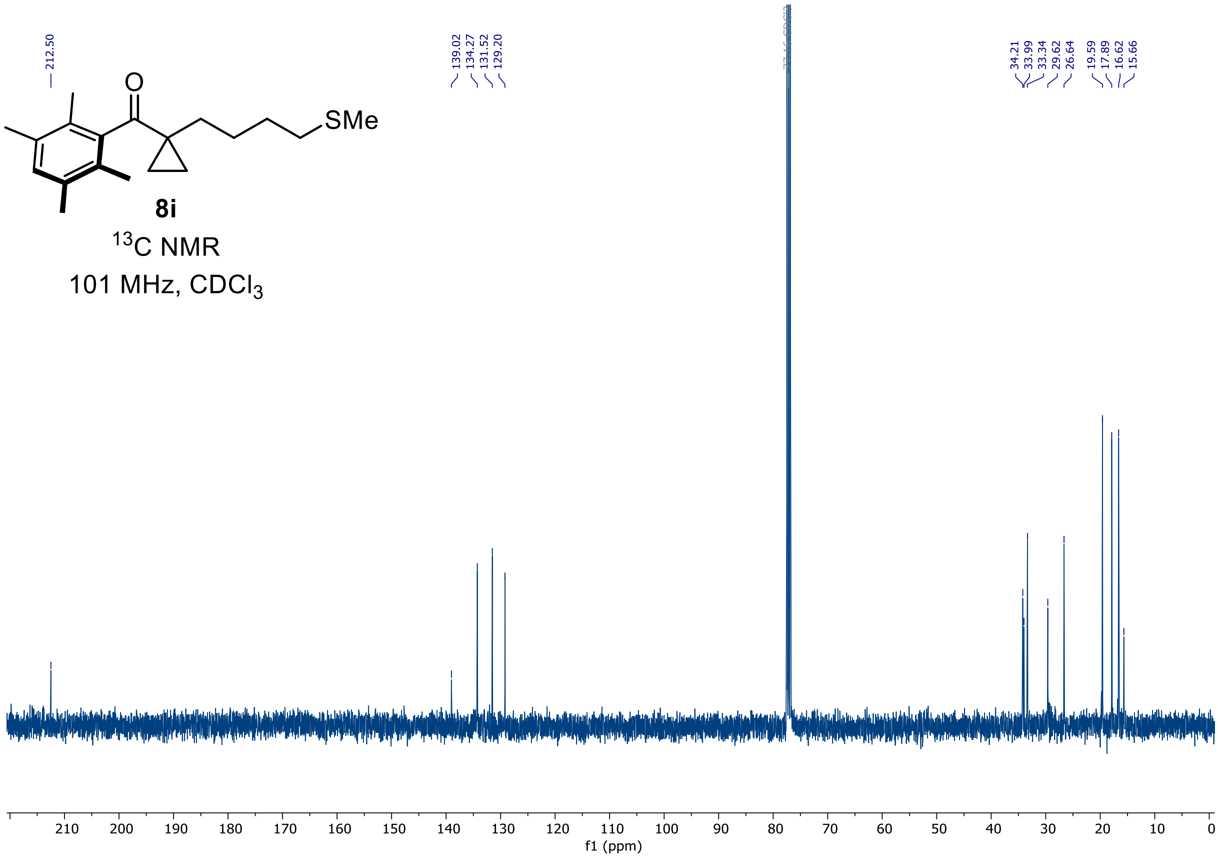

8j (1-(3-(Pyridin-3-yl)propyl)cyclopropyl)(2,3,5,6-tetramethylphenyl)methanone

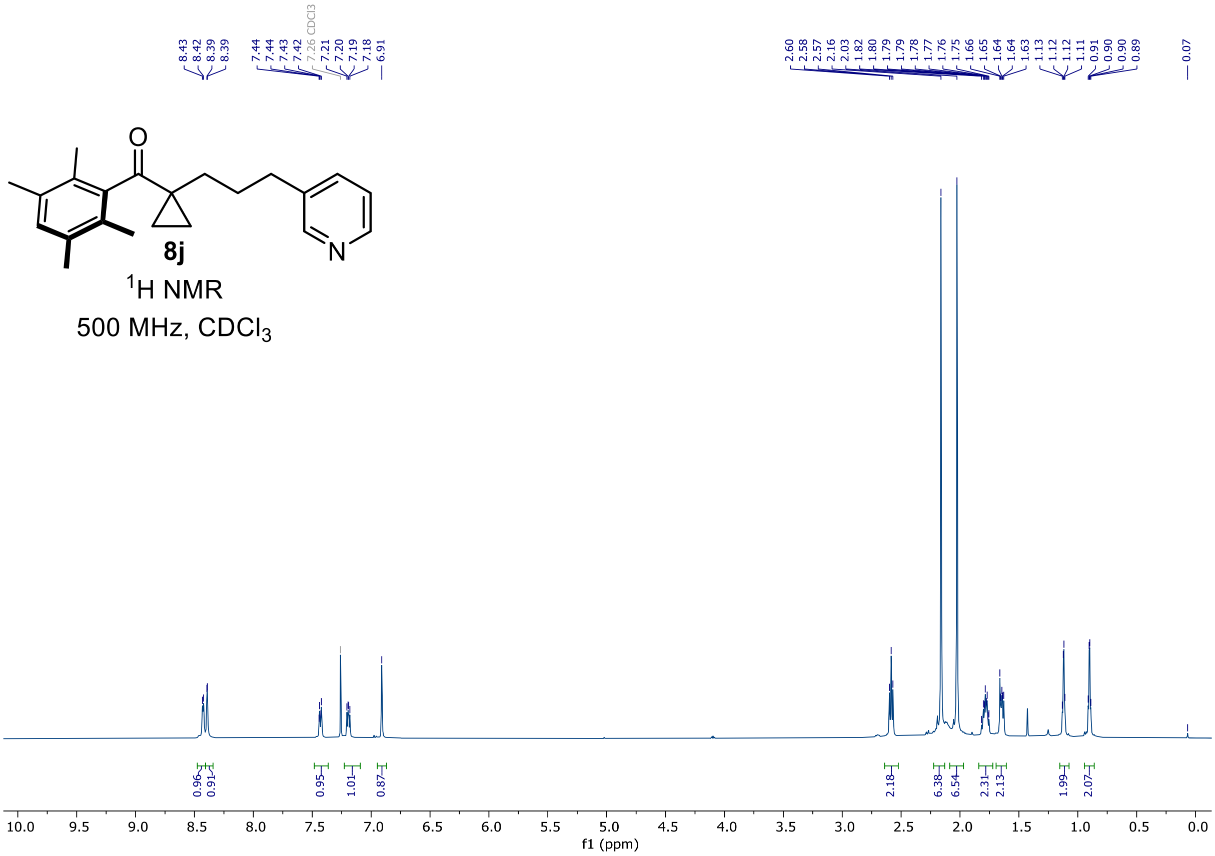

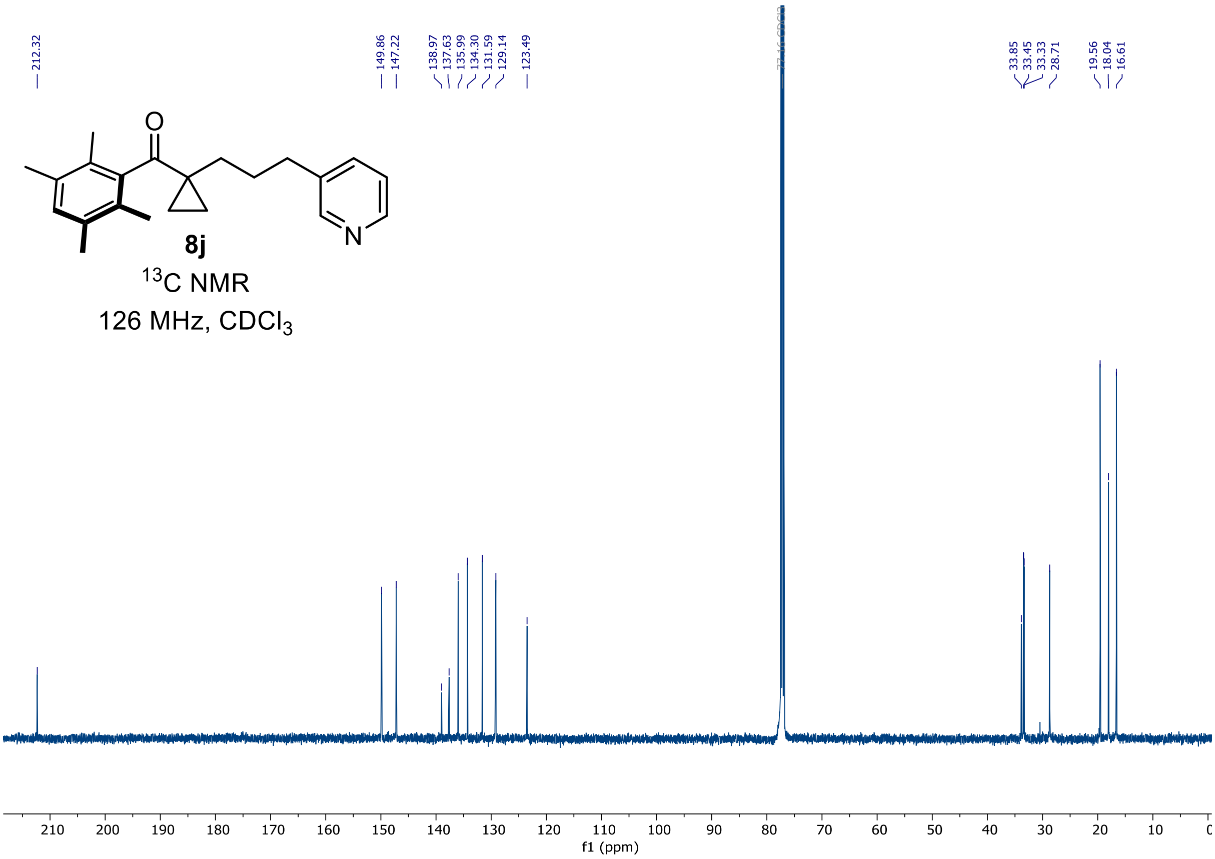

S5 4-(4-Methoxyphenoxy)-3-phenylbutanoic acid

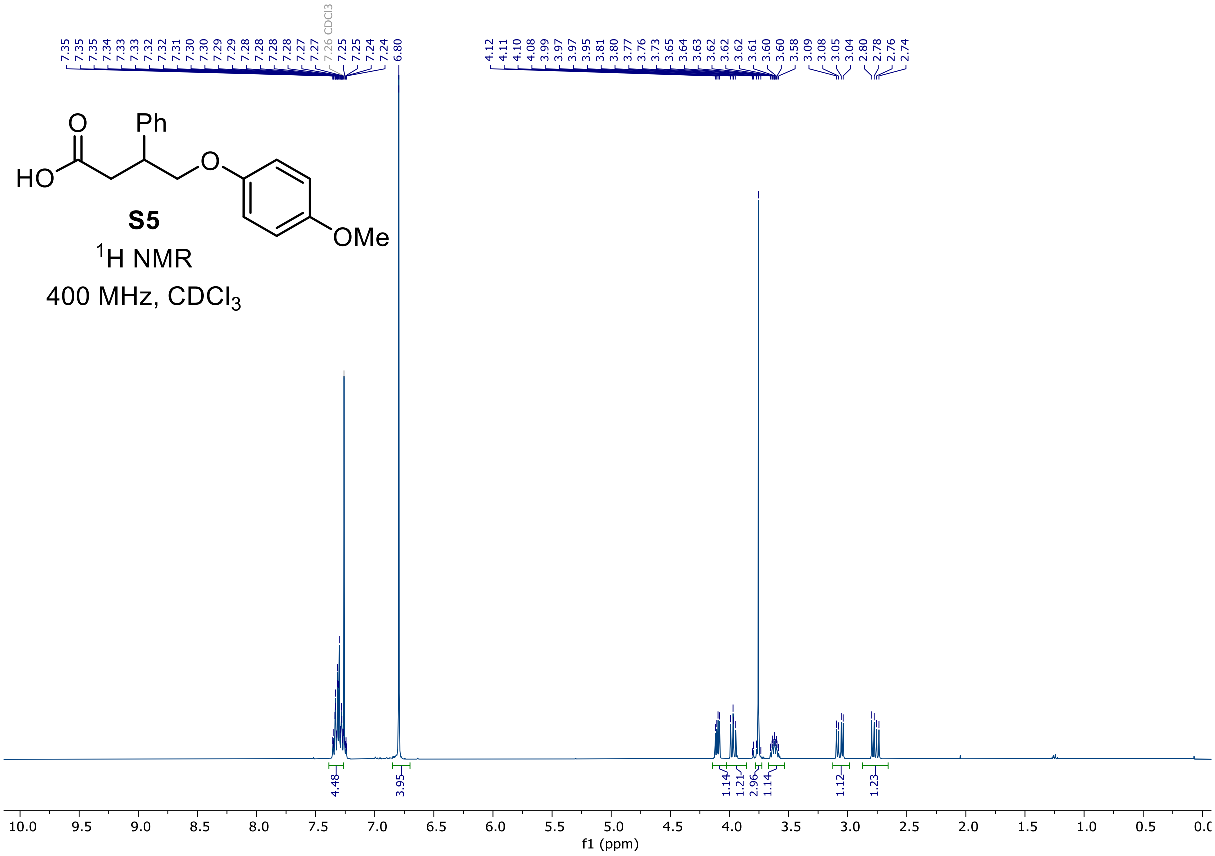

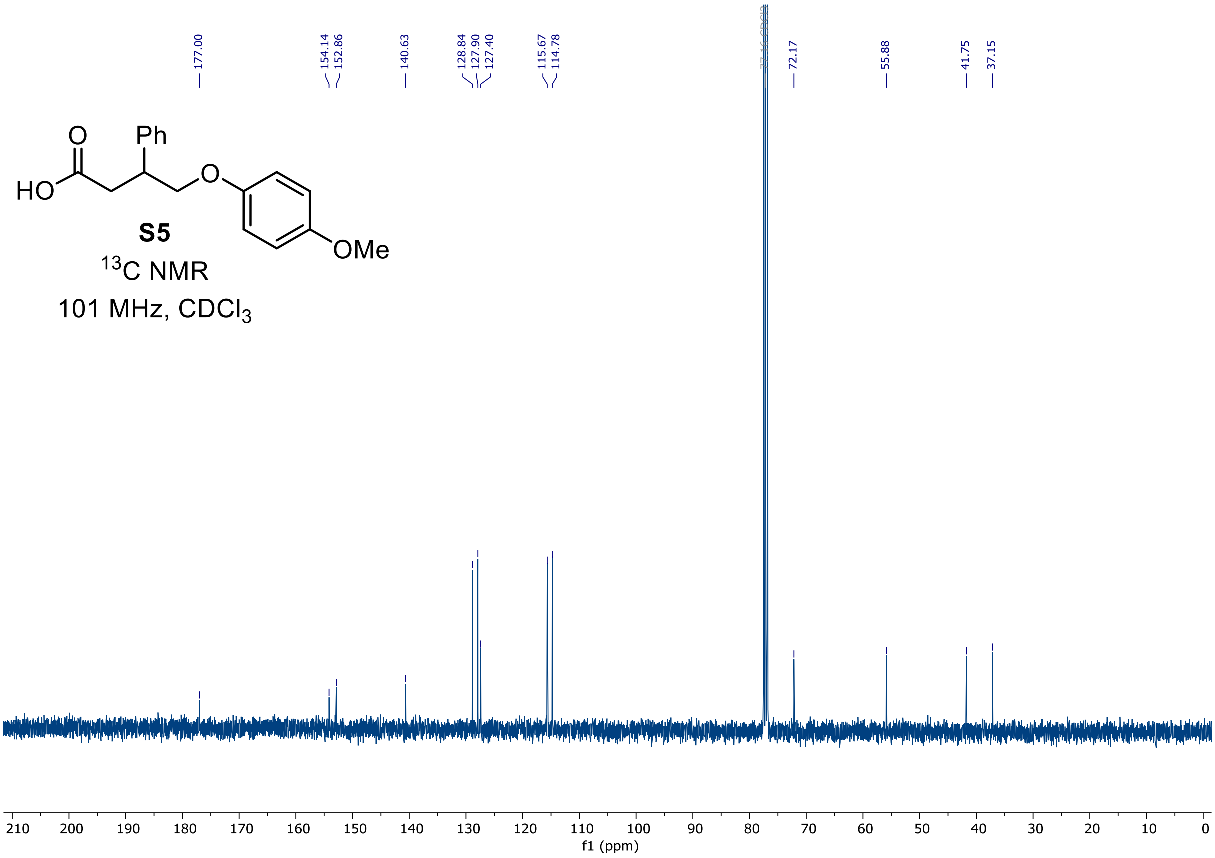

9 4-(4-Methoxyphenoxy)-3-phenyl-1-(2,3,5,6-tetramethylphenyl)butan-1-one

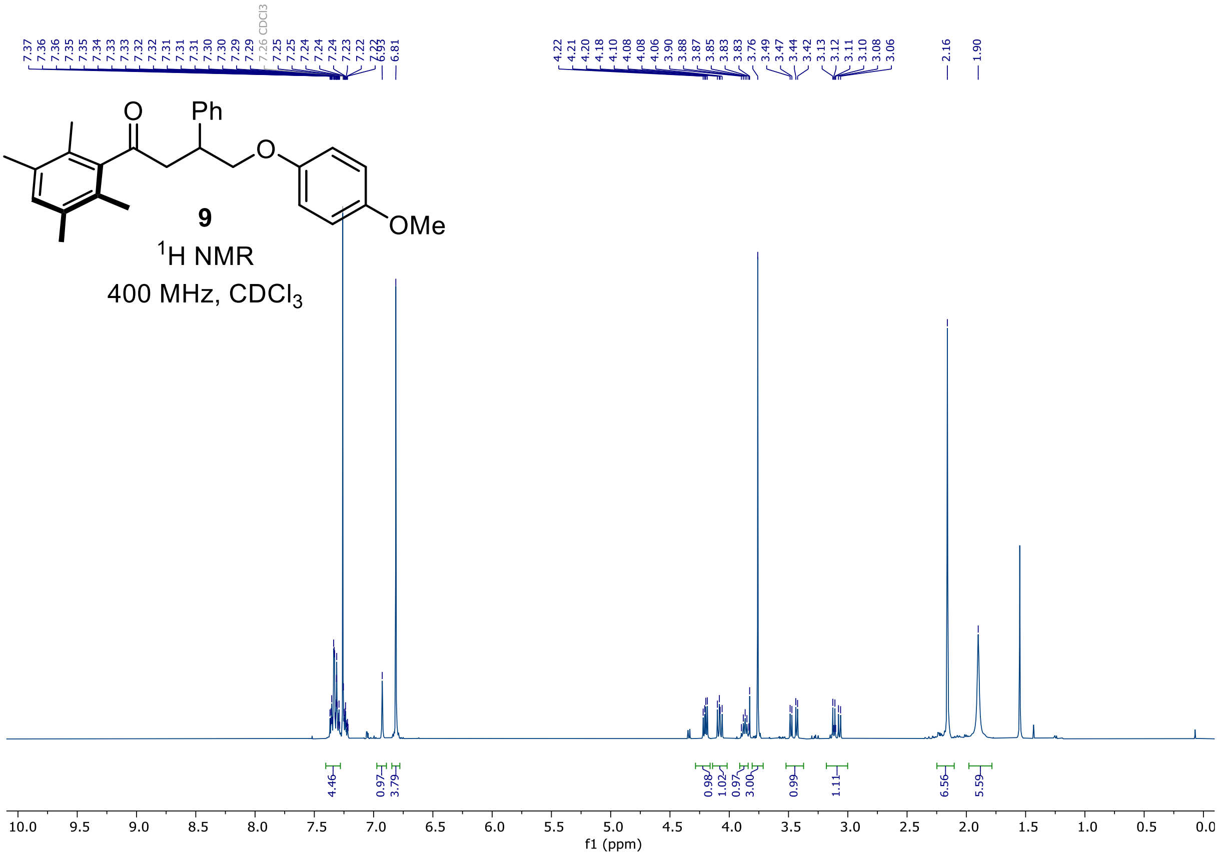

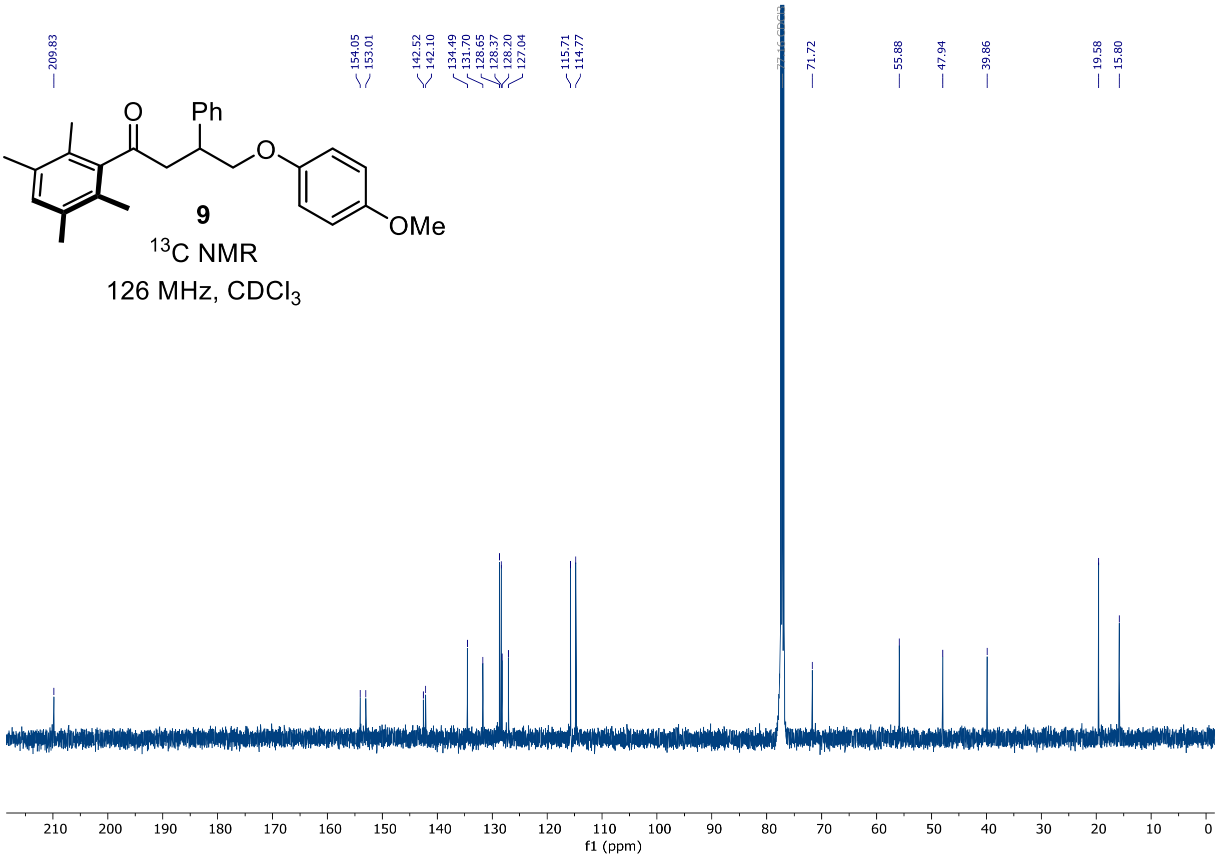

10 (2-Phenylcyclopropyl)(2,3,5,6-tetramethylphenyl)methanone

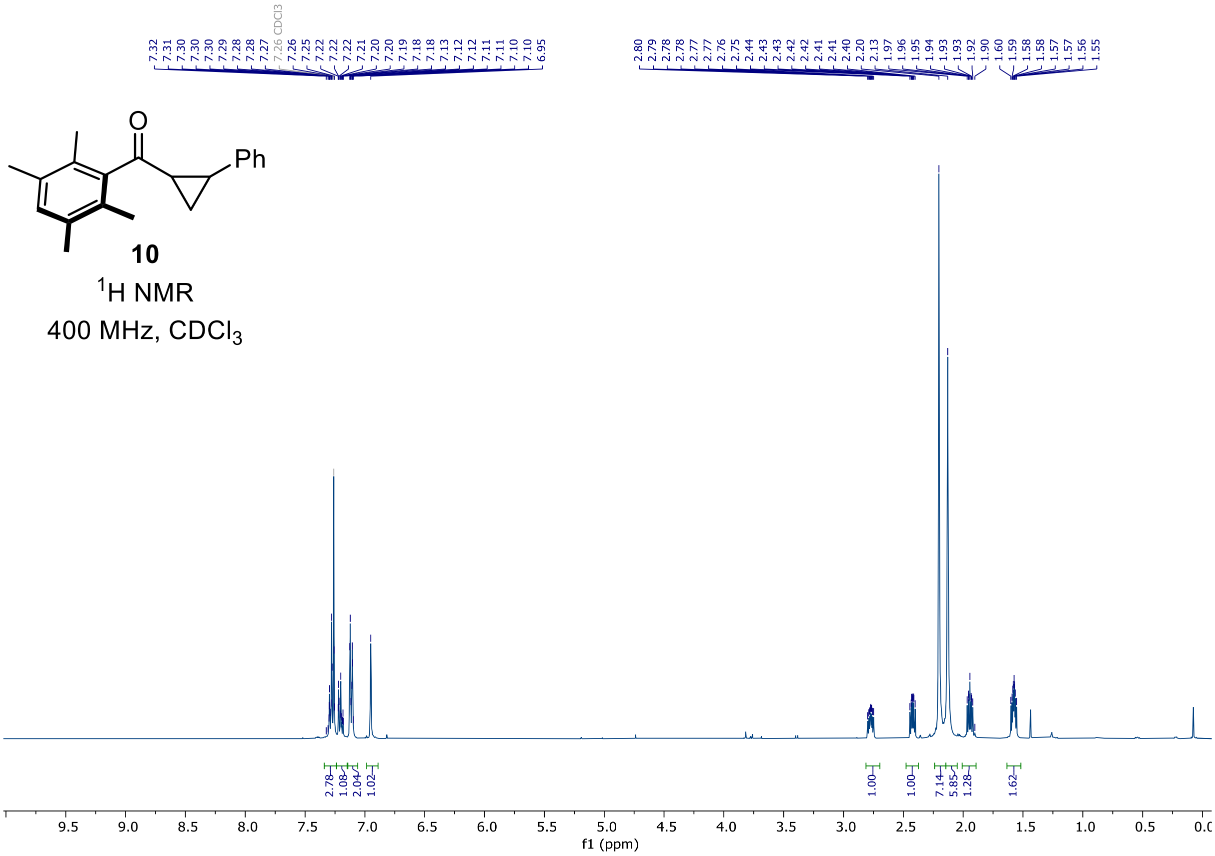

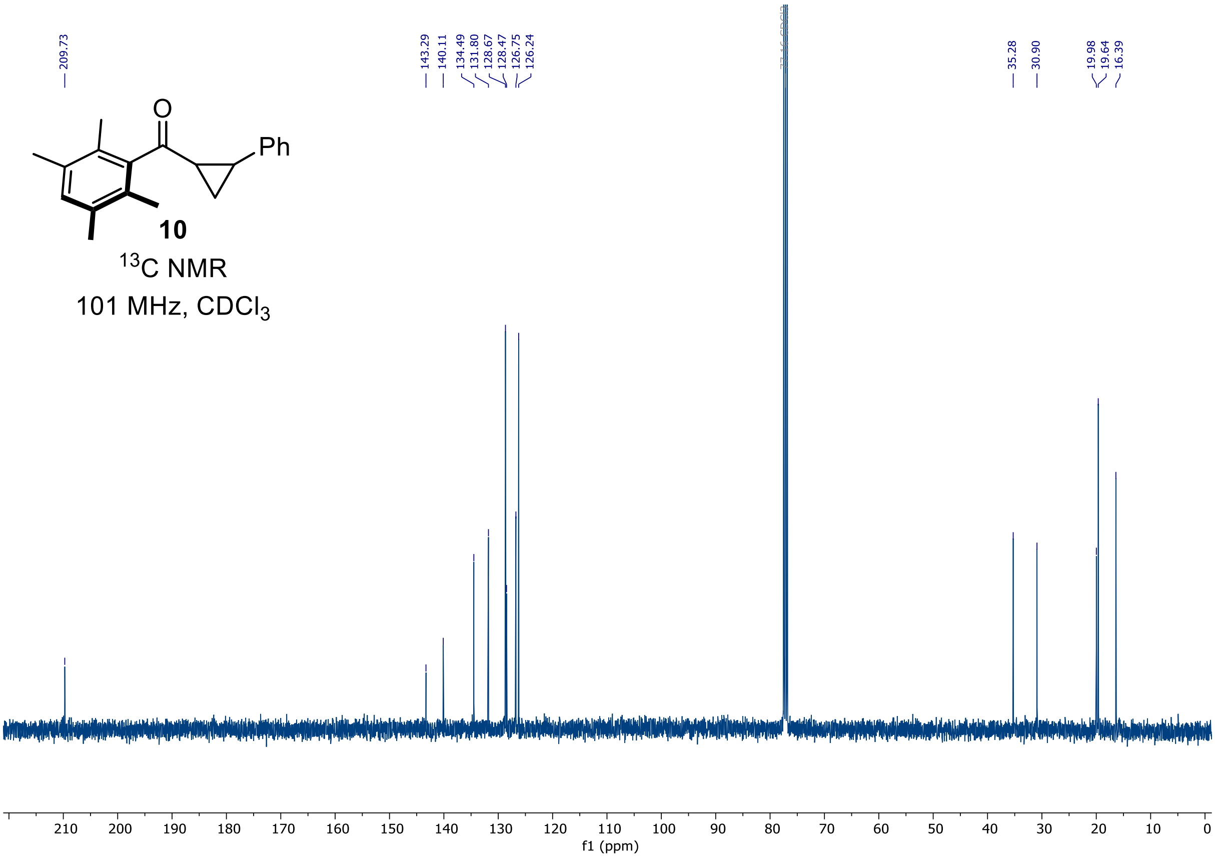

S6 5-Oxo-5-(2,3,4,5,6-pentamethylphenyl)pentanoic acid

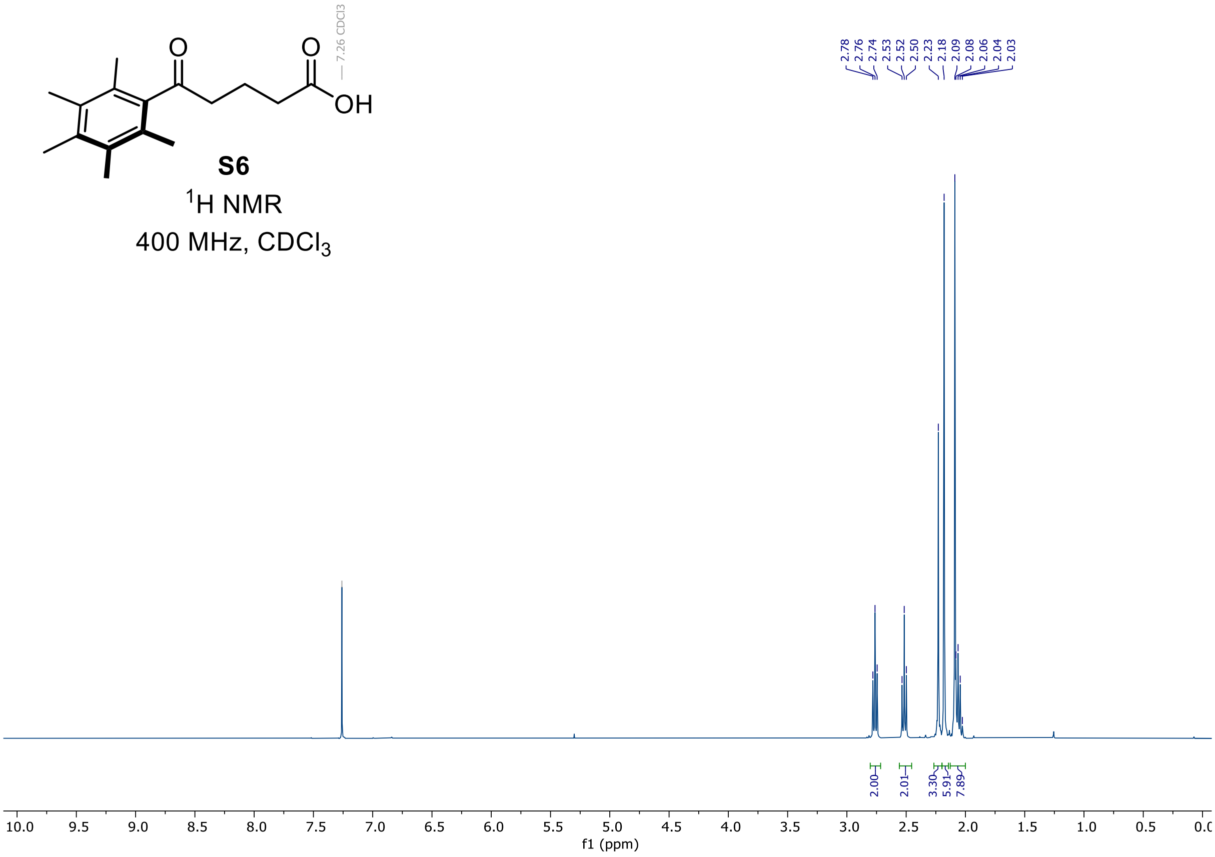

S6
^{1}H NMR
400 MHz, $CDCl_3$

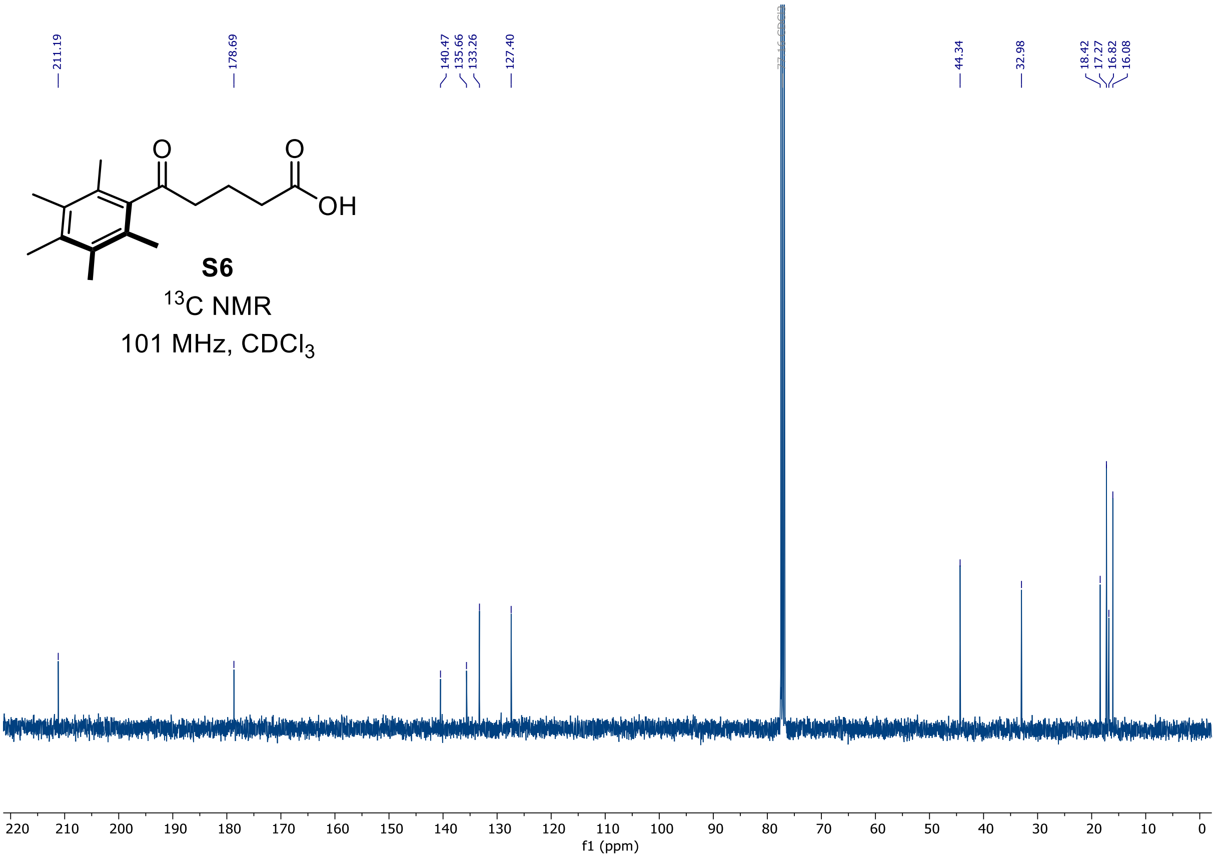

S6
^{13}C NMR
101 MHz, $CDCl_3$

S7 5-Hydroxy-1-(2,3,4,5,6-pentamethylphenyl)pentan-1-one

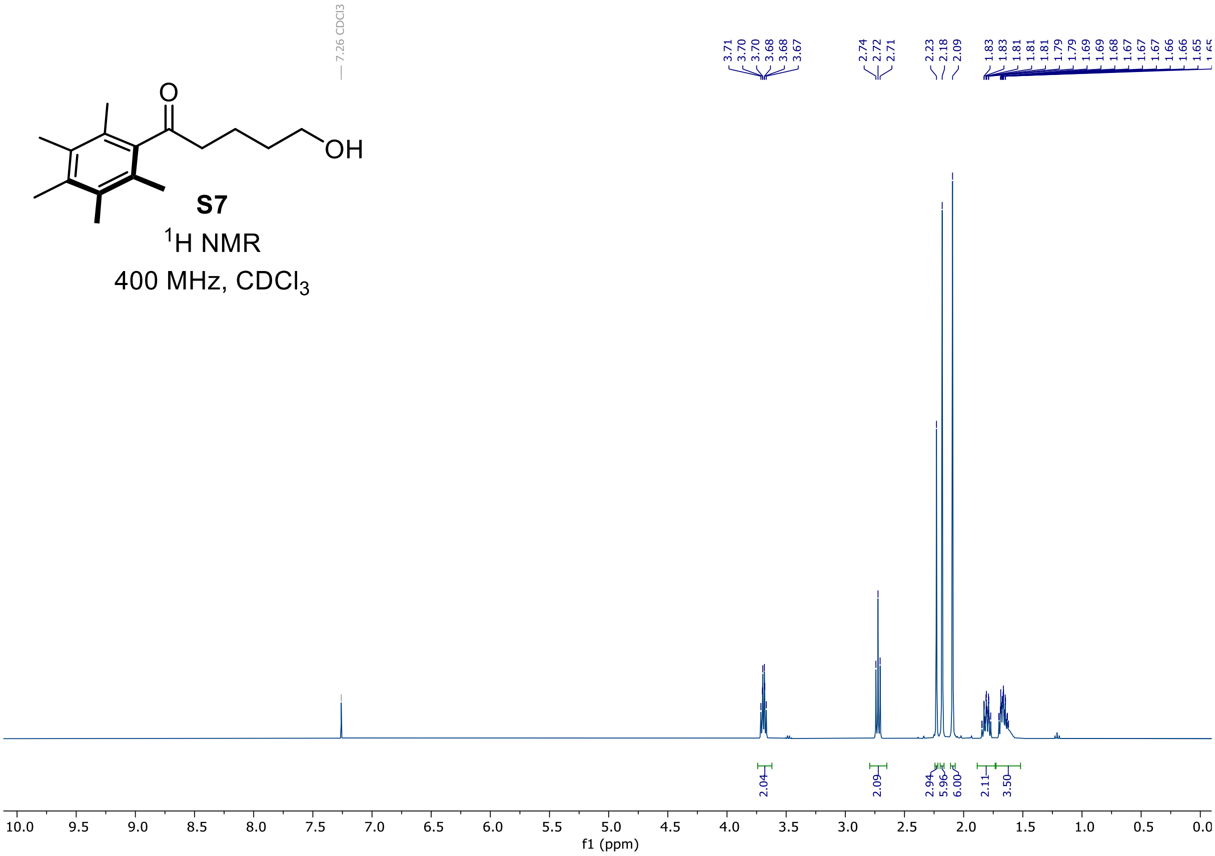

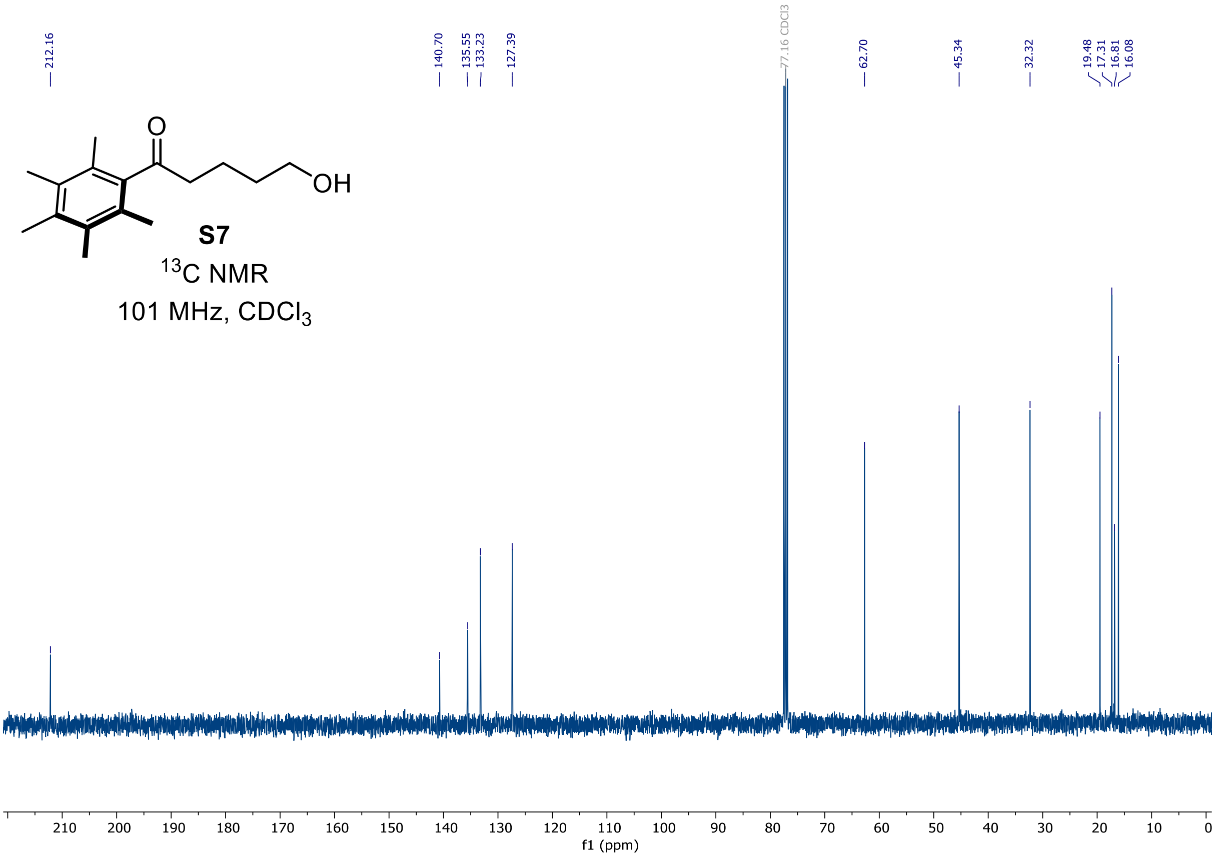

11 1-(2,3,4,5,6-Pentamethylphenyl)-5-phenoxypentan-1-one

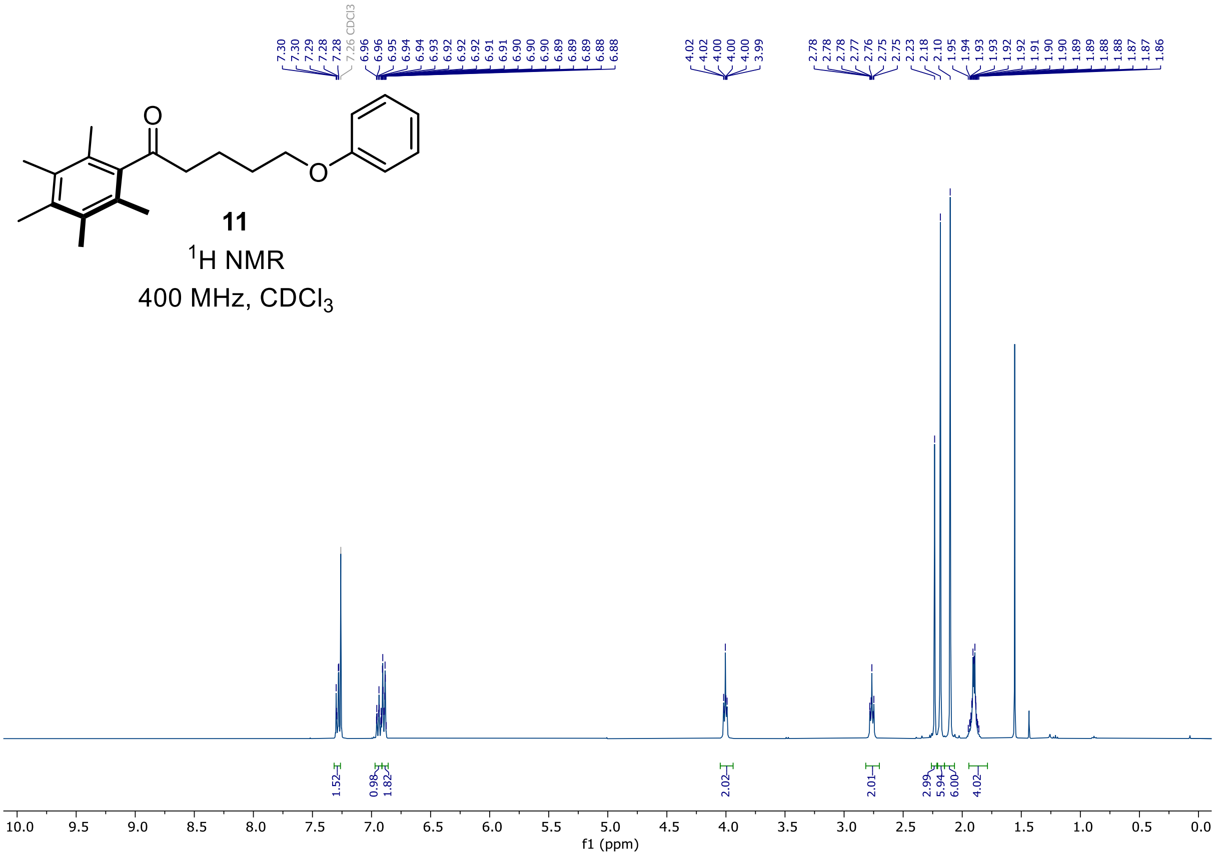

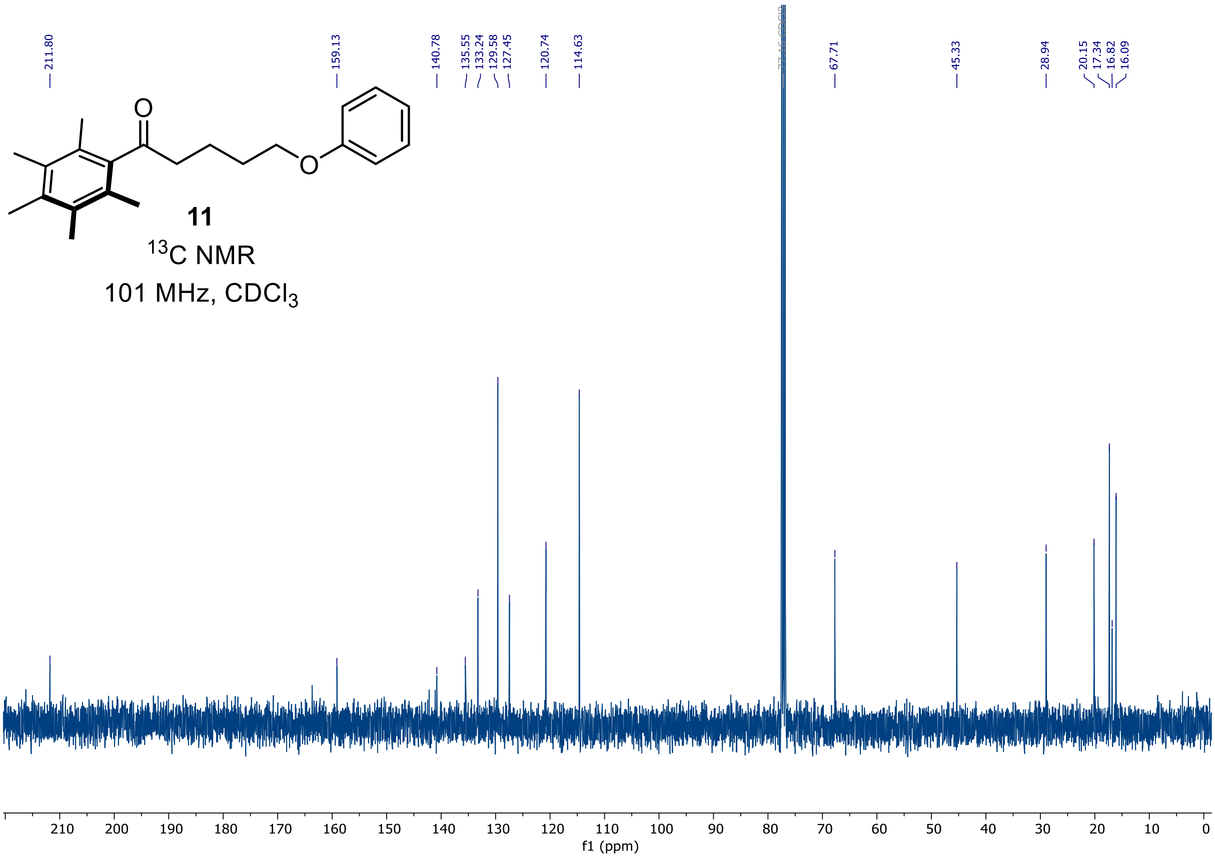

S8 1-(2,3,4,5,6-Pentamethylphenyl)-2-(3-phenoxypropyl)hexan-1-one

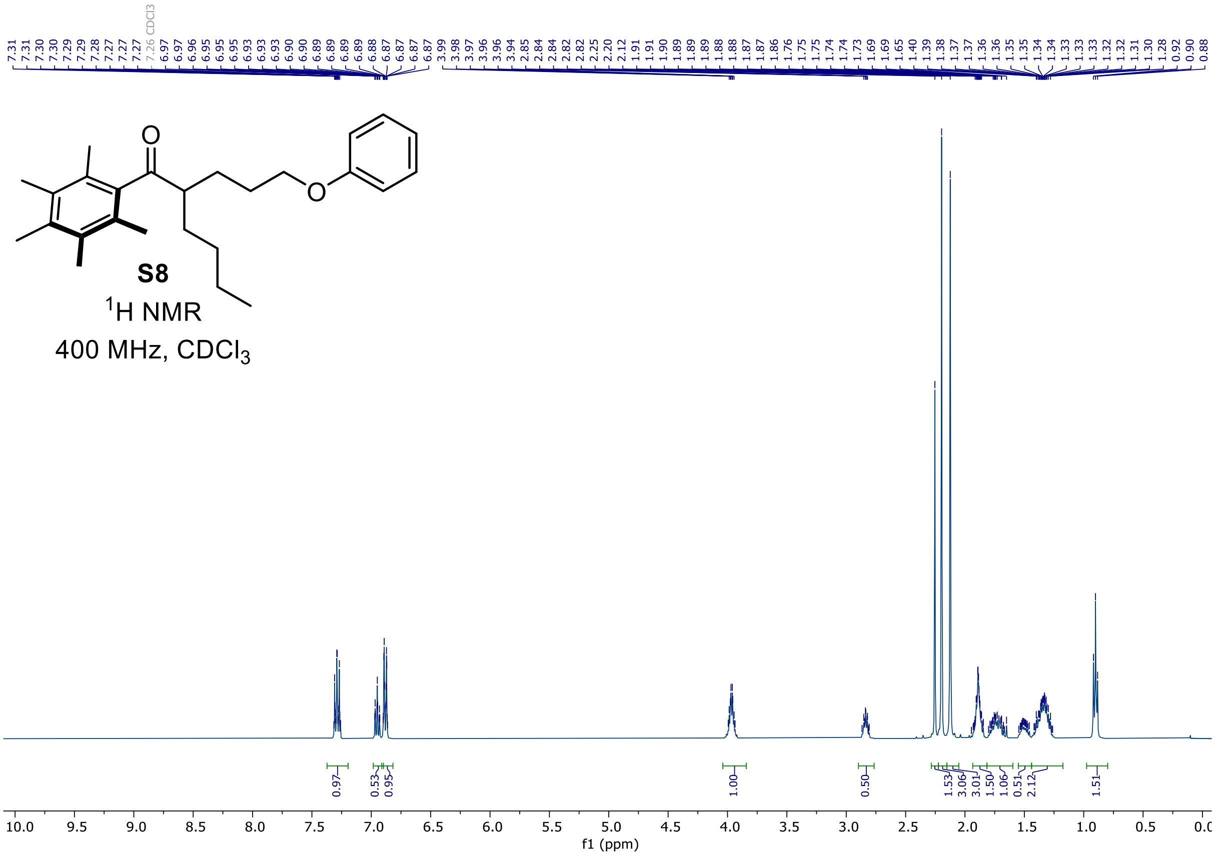

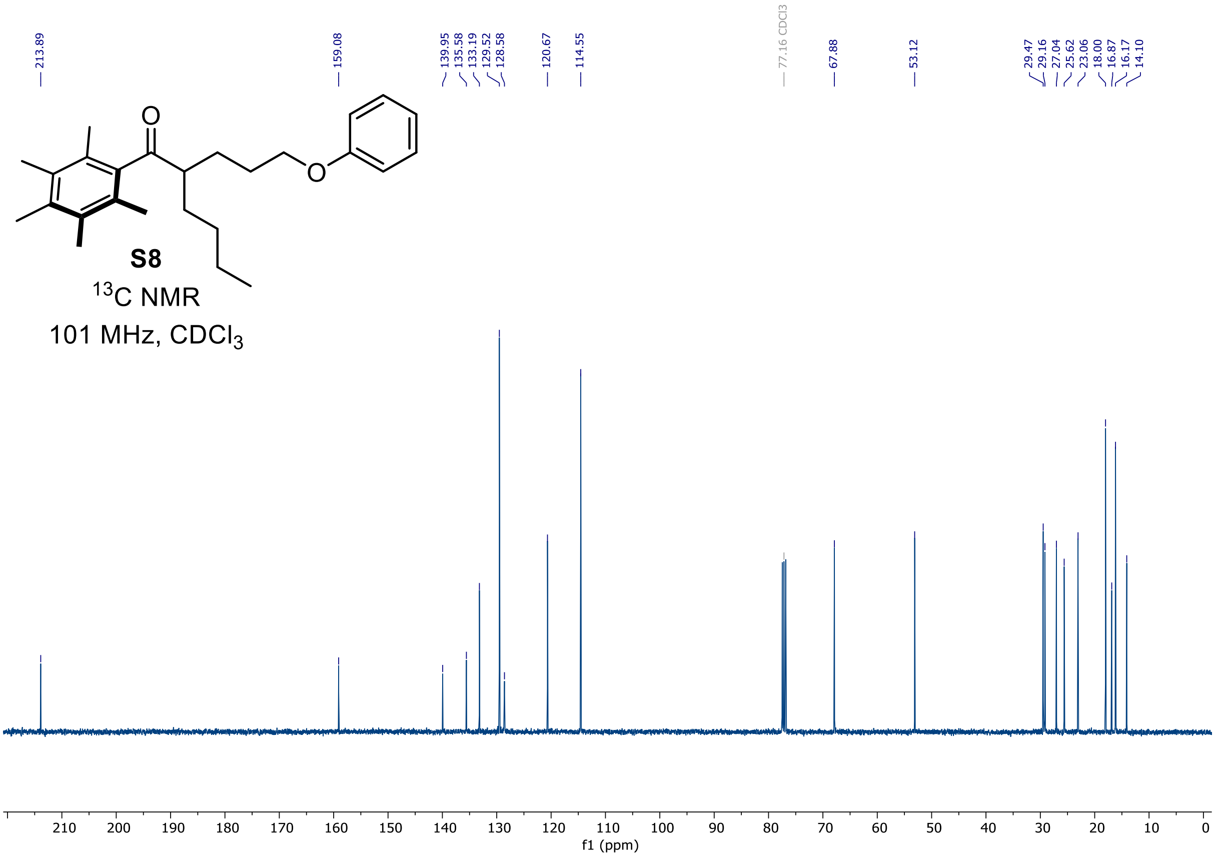

S9 Methyl 6-oxo-6-(2,3,4,5,6-pentamethylphenyl)hexanoate

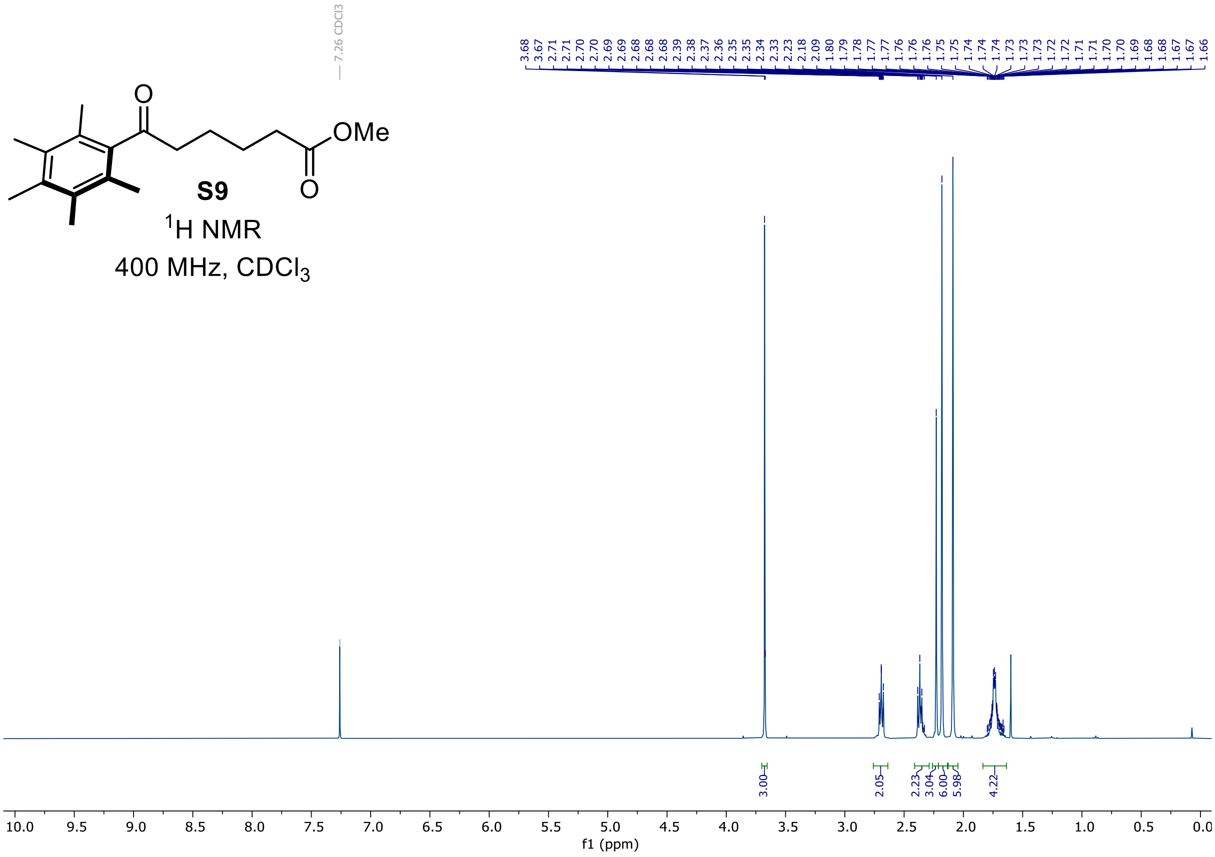

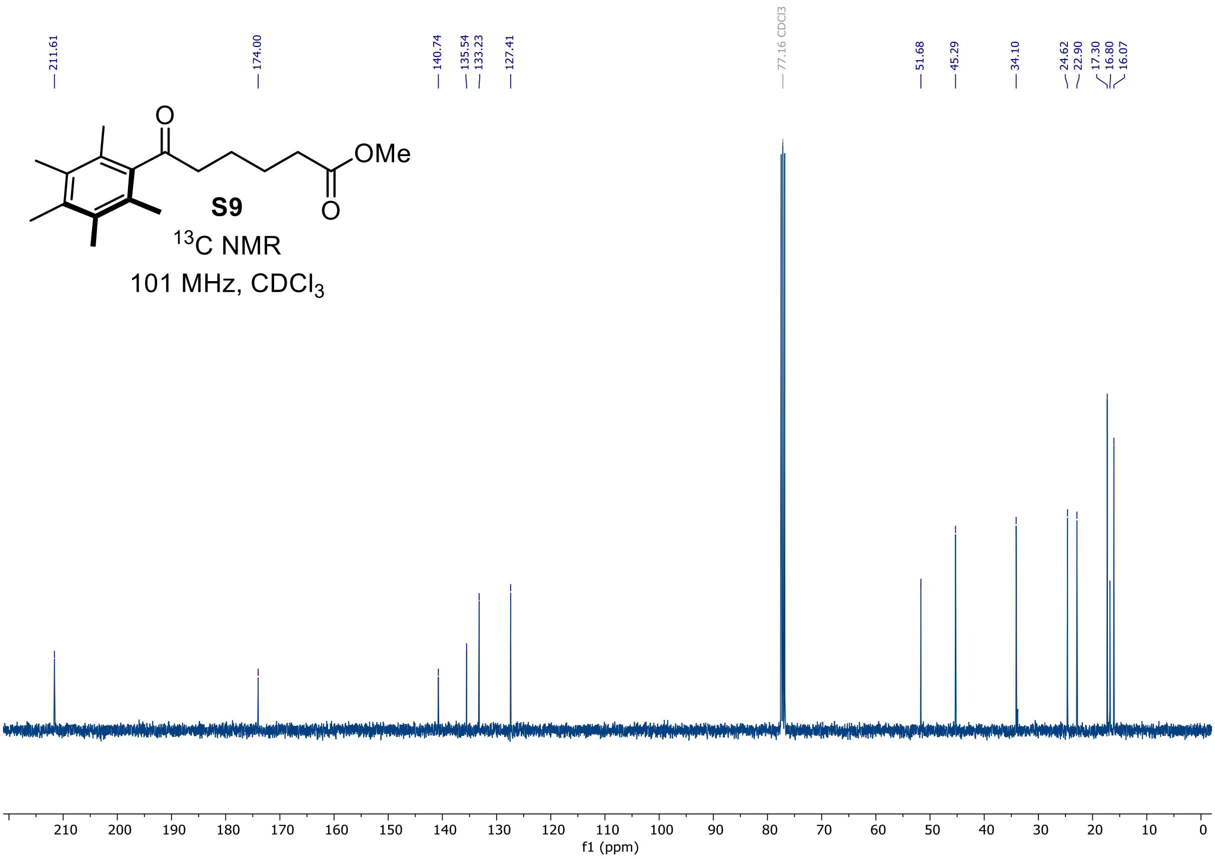

S10 6-Hydroxy-1-(2,3,4,5,6-pentamethylphenyl)hexan-1-one

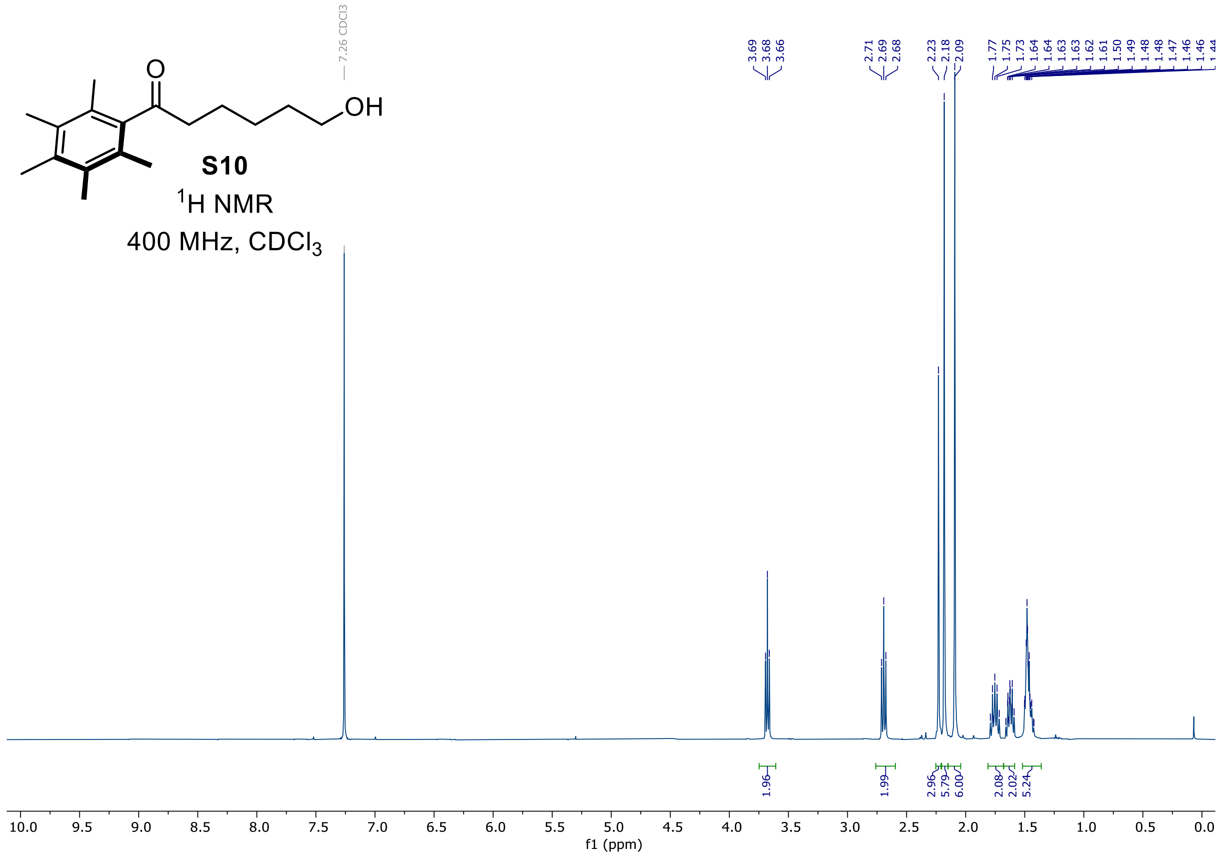

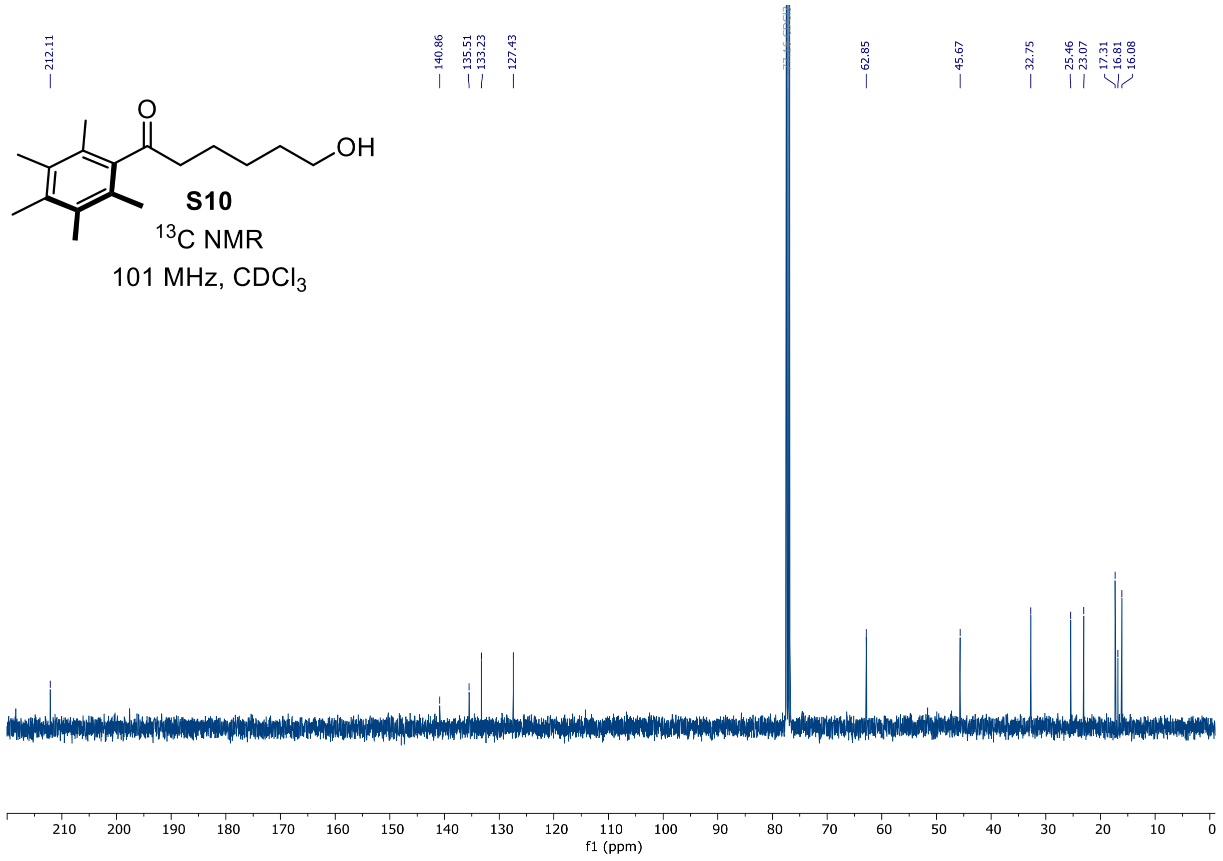

12 1-(2,3,4,5,6-Pentamethylphenyl)-6-phenoxyhexan-1-one

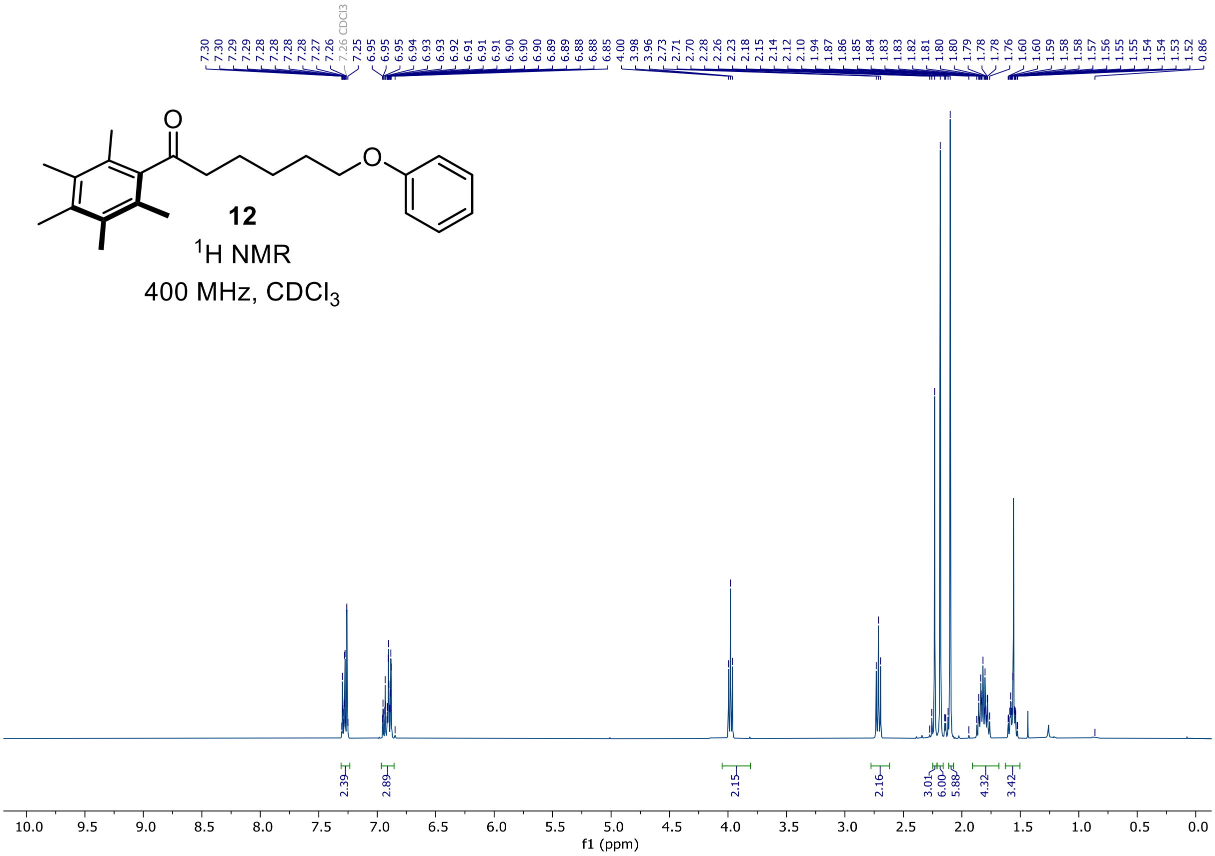

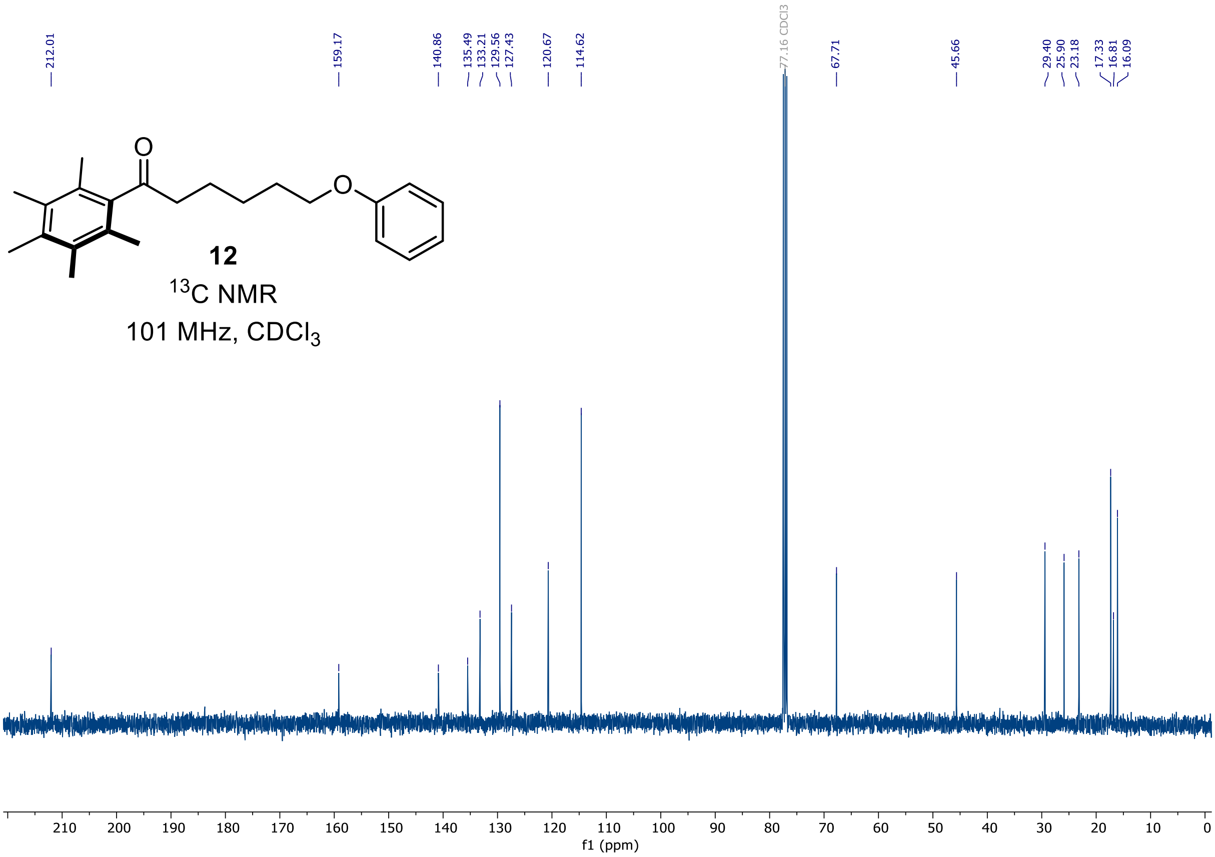

S11 2-Butyl-1-(2,3,4,5,6-pentamethylphenyl)-6-phenoxyhexan-1-one

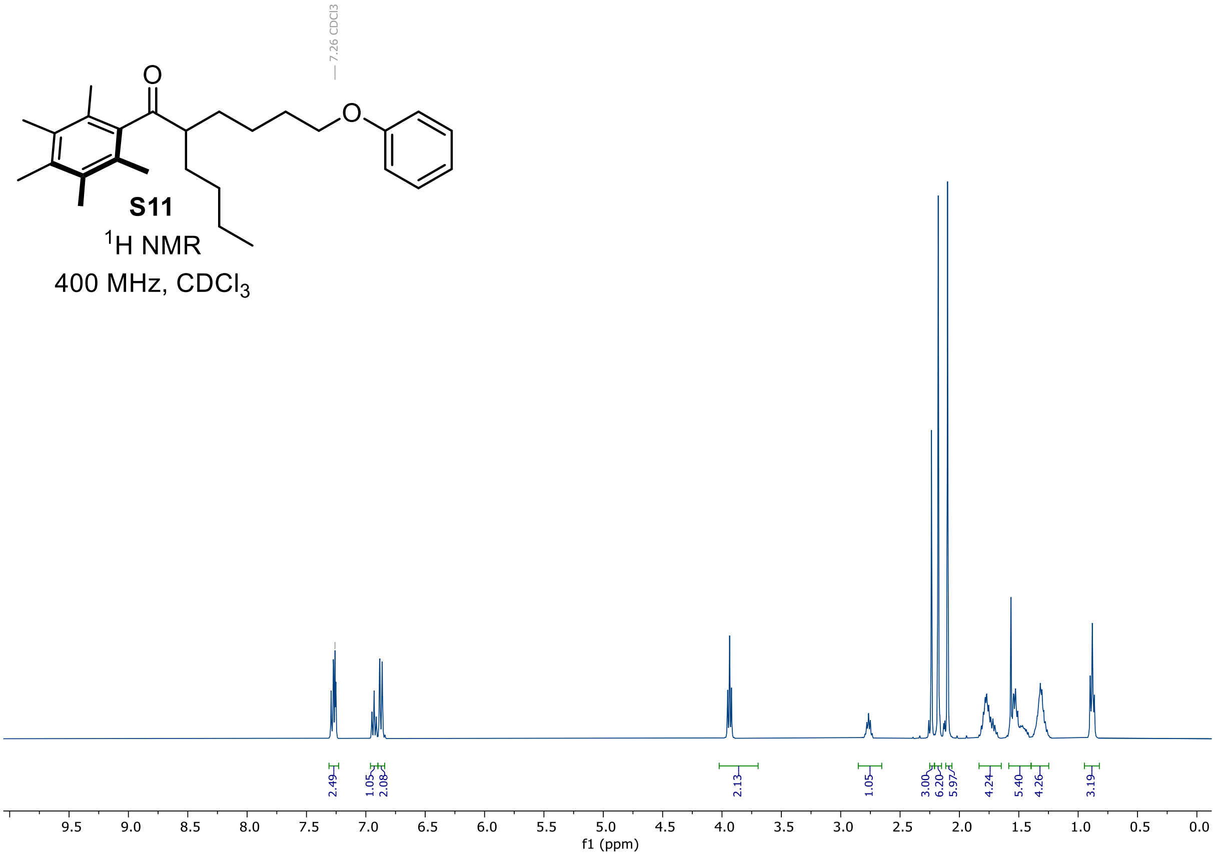

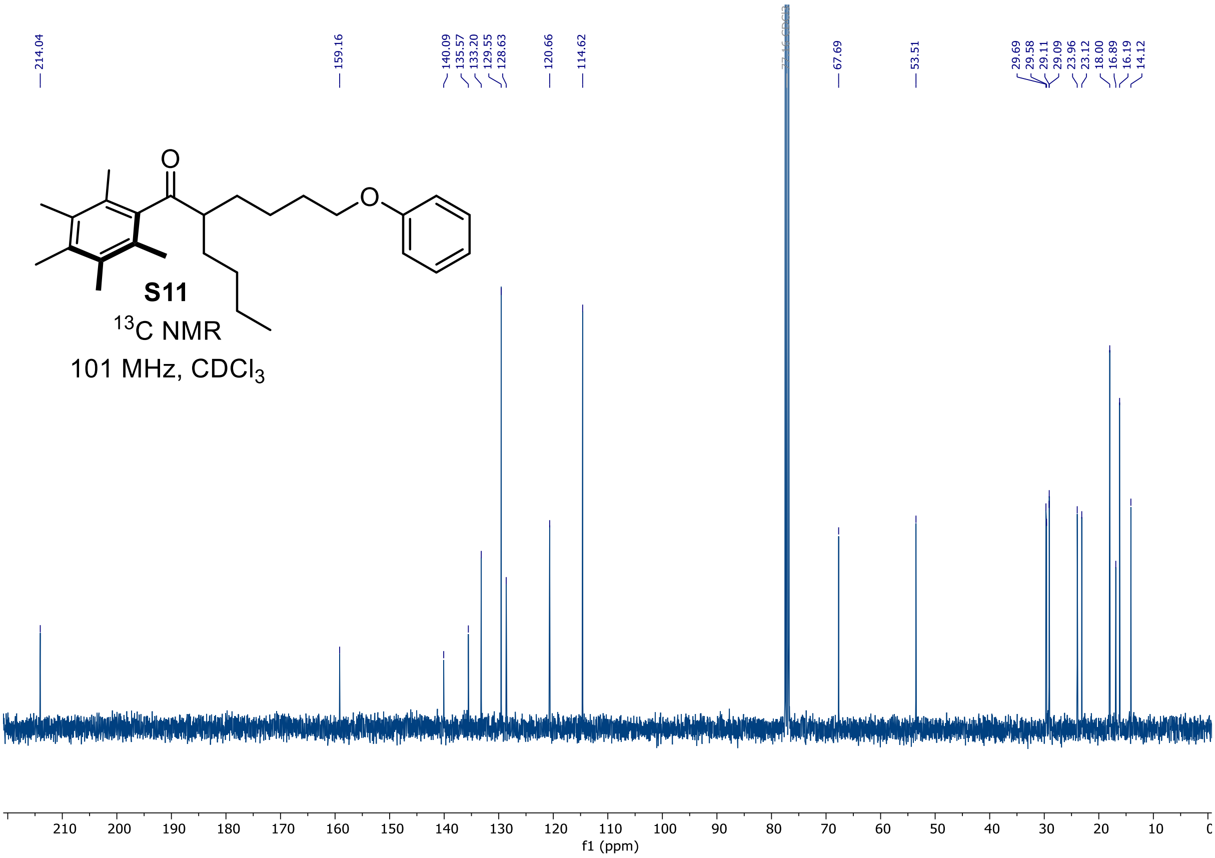

S12 6-Bromo-1-(2,3,4,5,6-pentamethylphenyl)hexan-1-one

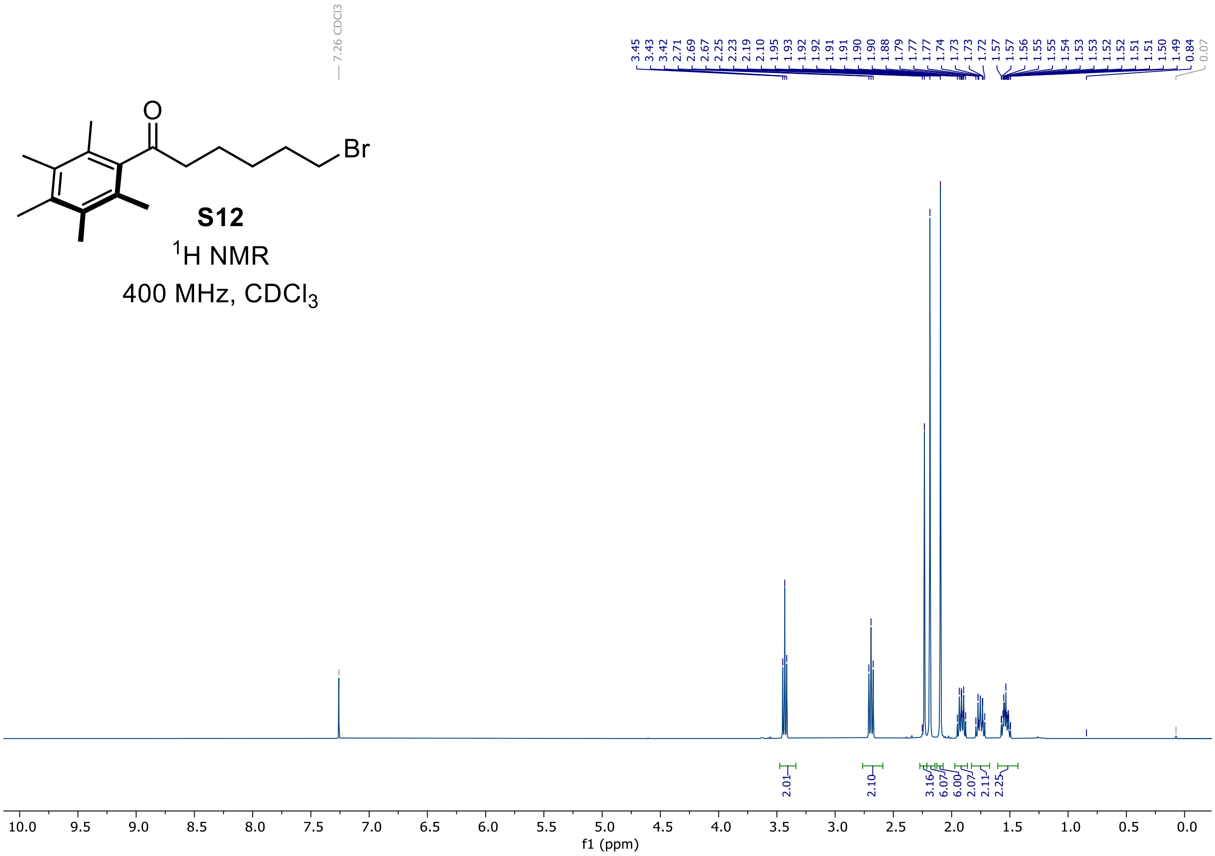

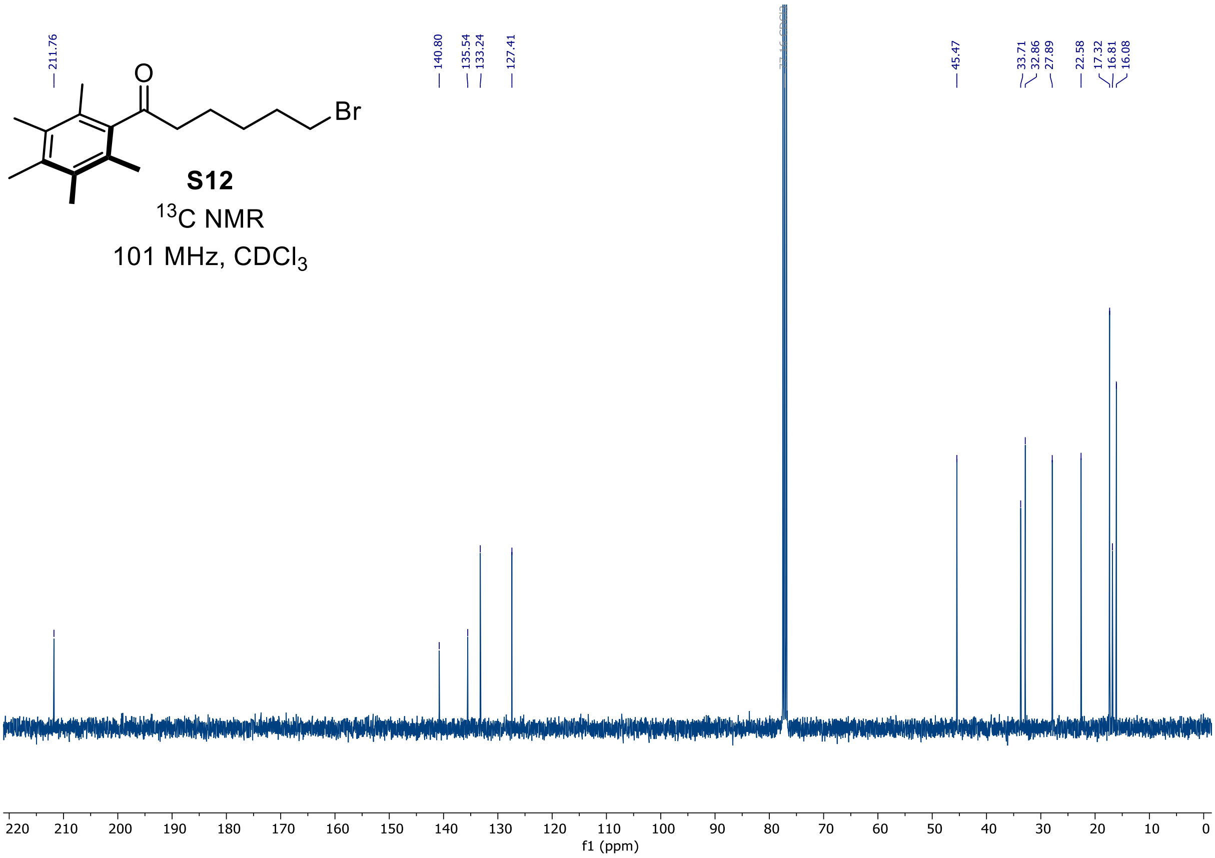

13 1-(2,3,4,5,6-Pentamethylphenyl)-6-(phenylthio)hexan-1-one

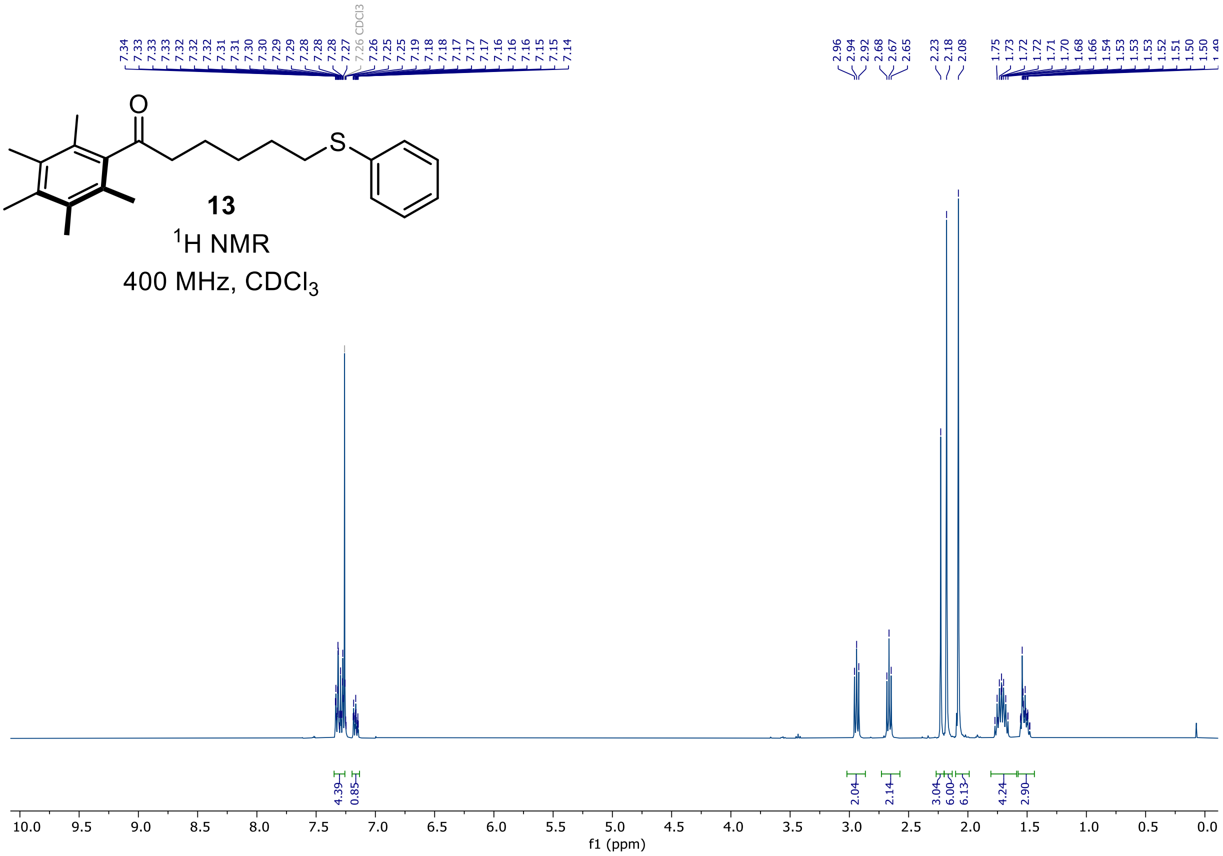

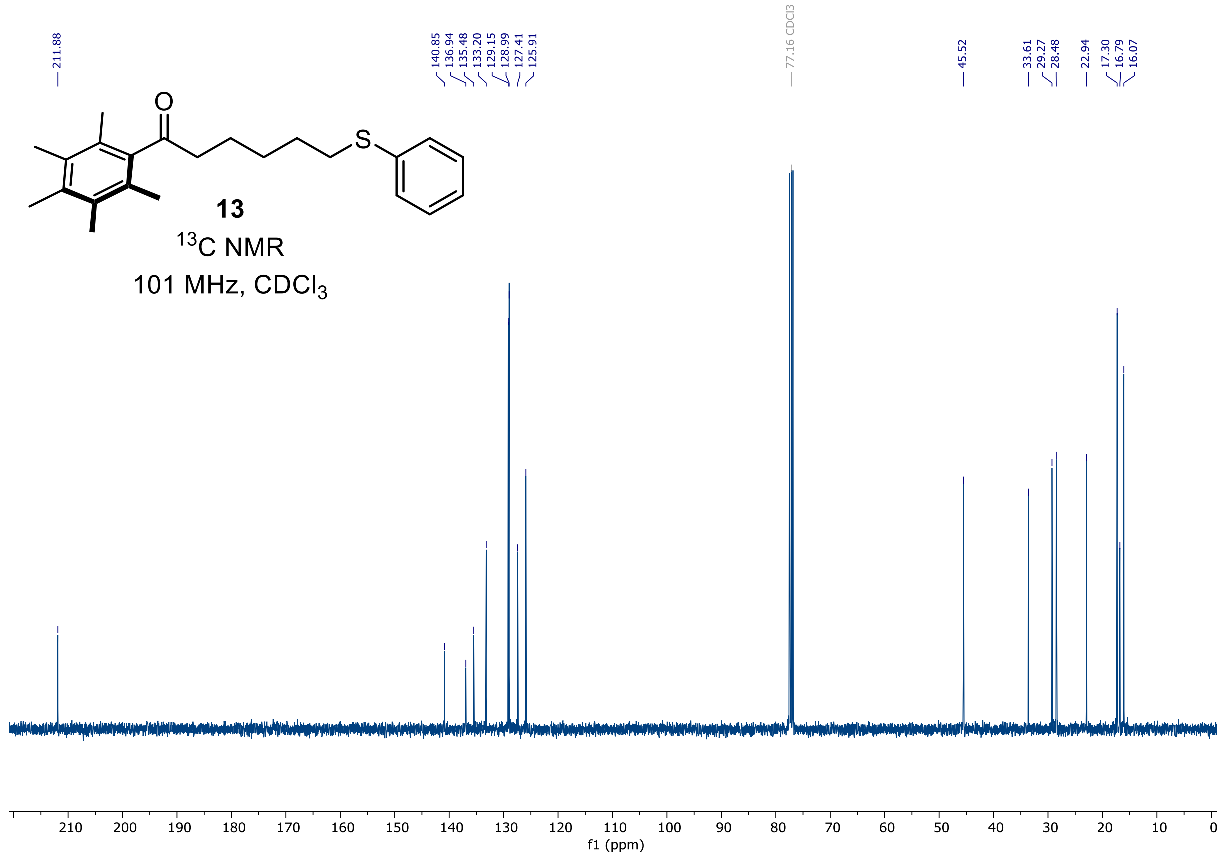

S13 2-Benzyl-1-(2,3,4,5,6-pentamethylphenyl)-6-(phenylthio)hexan-1-one

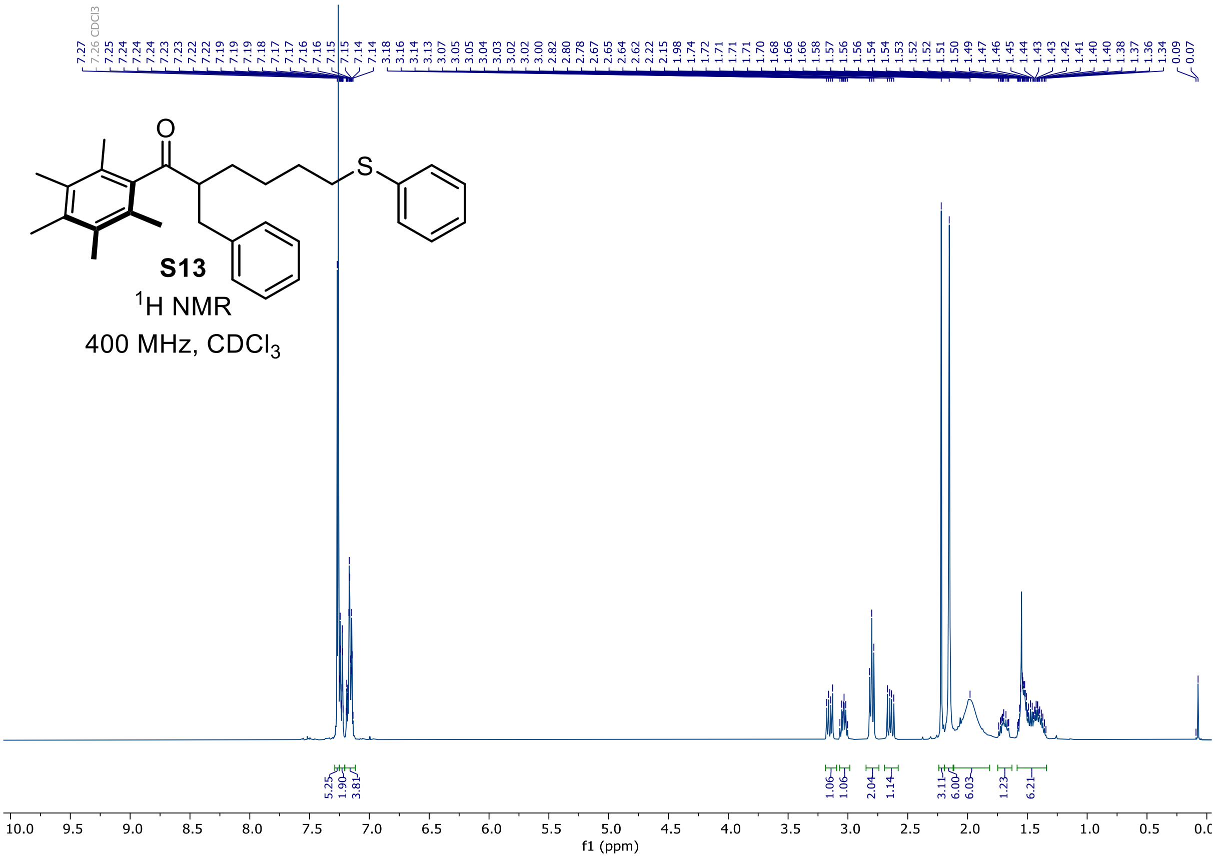

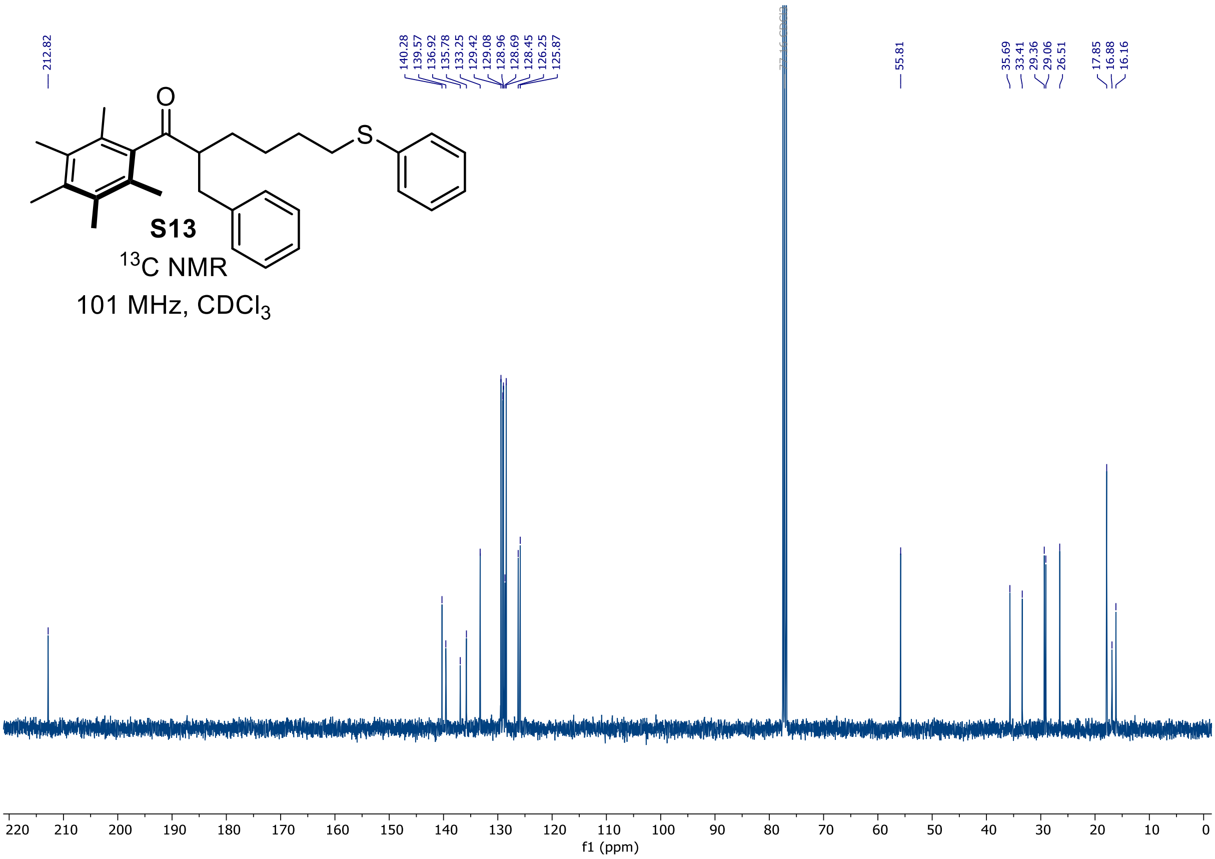

S15 5-Methyl-1-(2,3,5,6-tetramethylphenyl)hexan-1-one

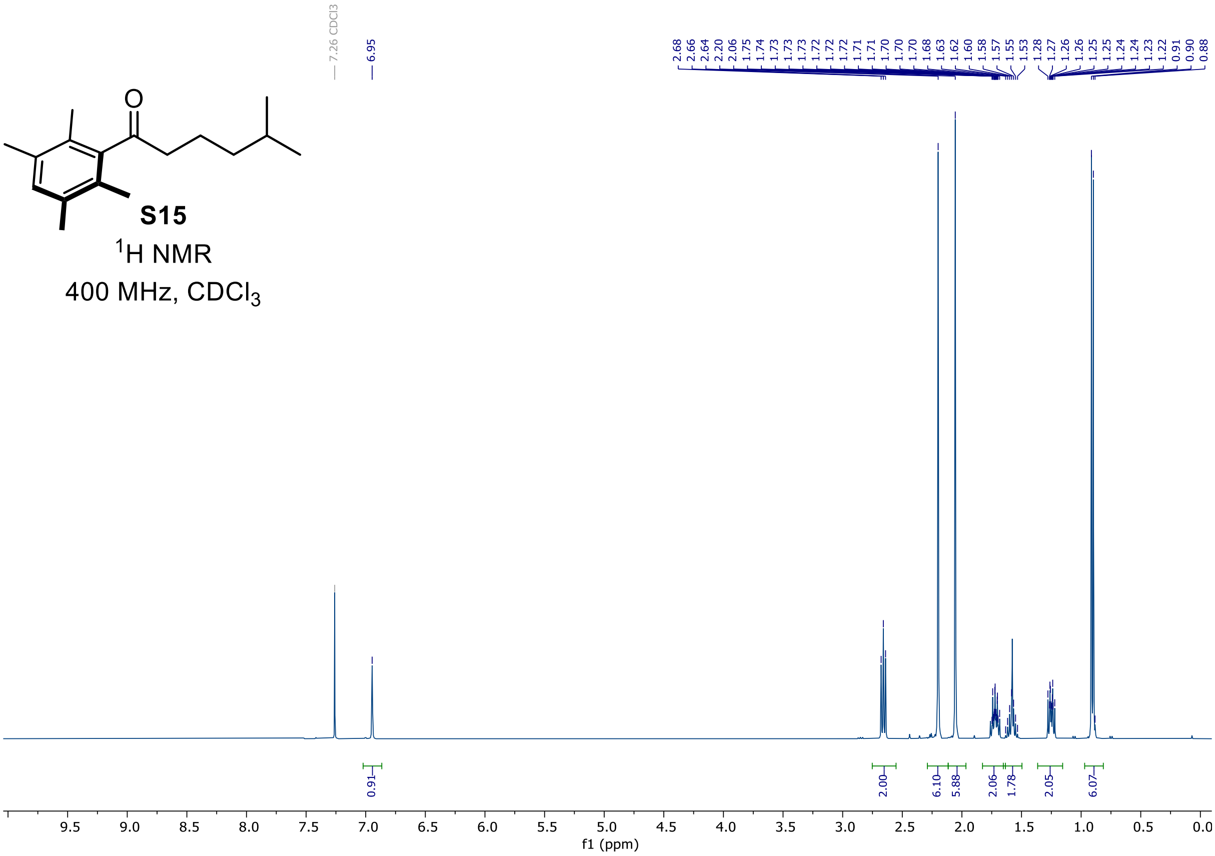

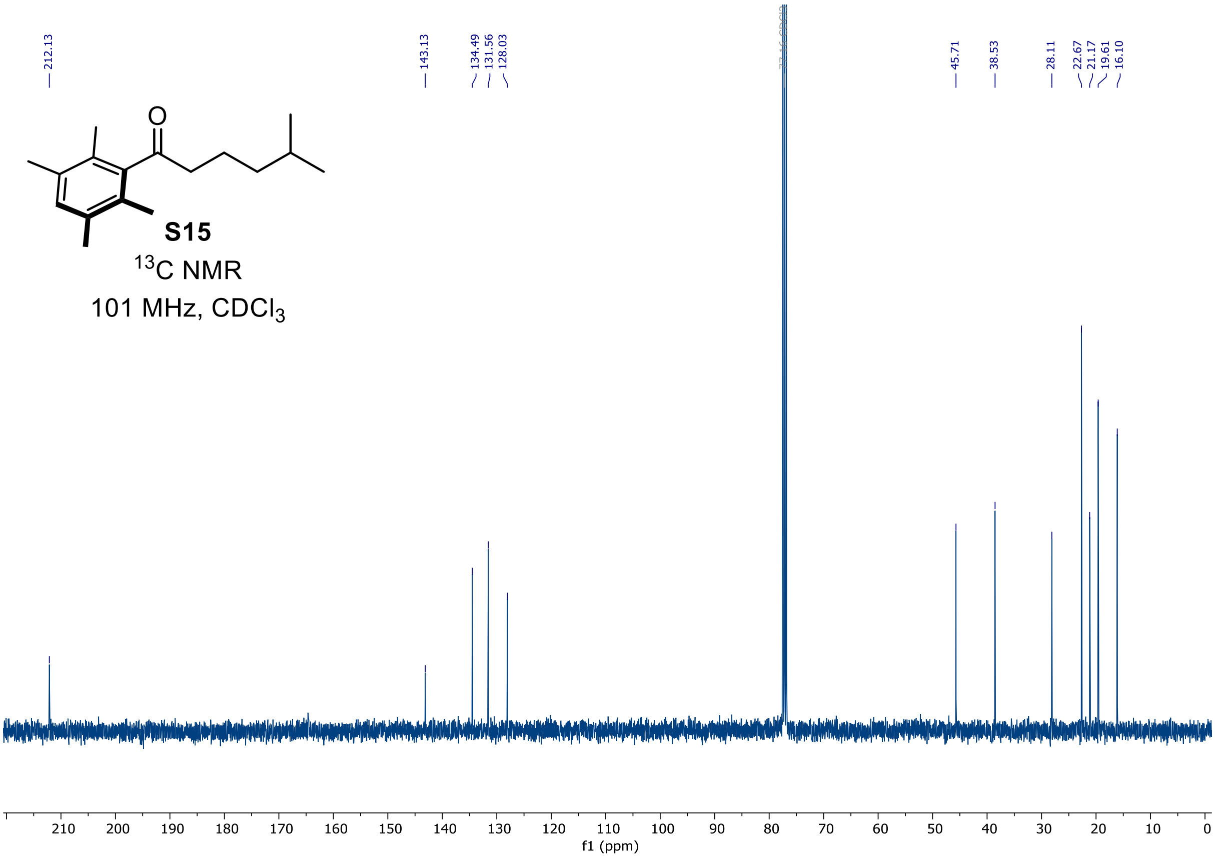

S16 2-Phenyl-1-(2,3,5,6-tetramethylphenyl)ethan-1-one

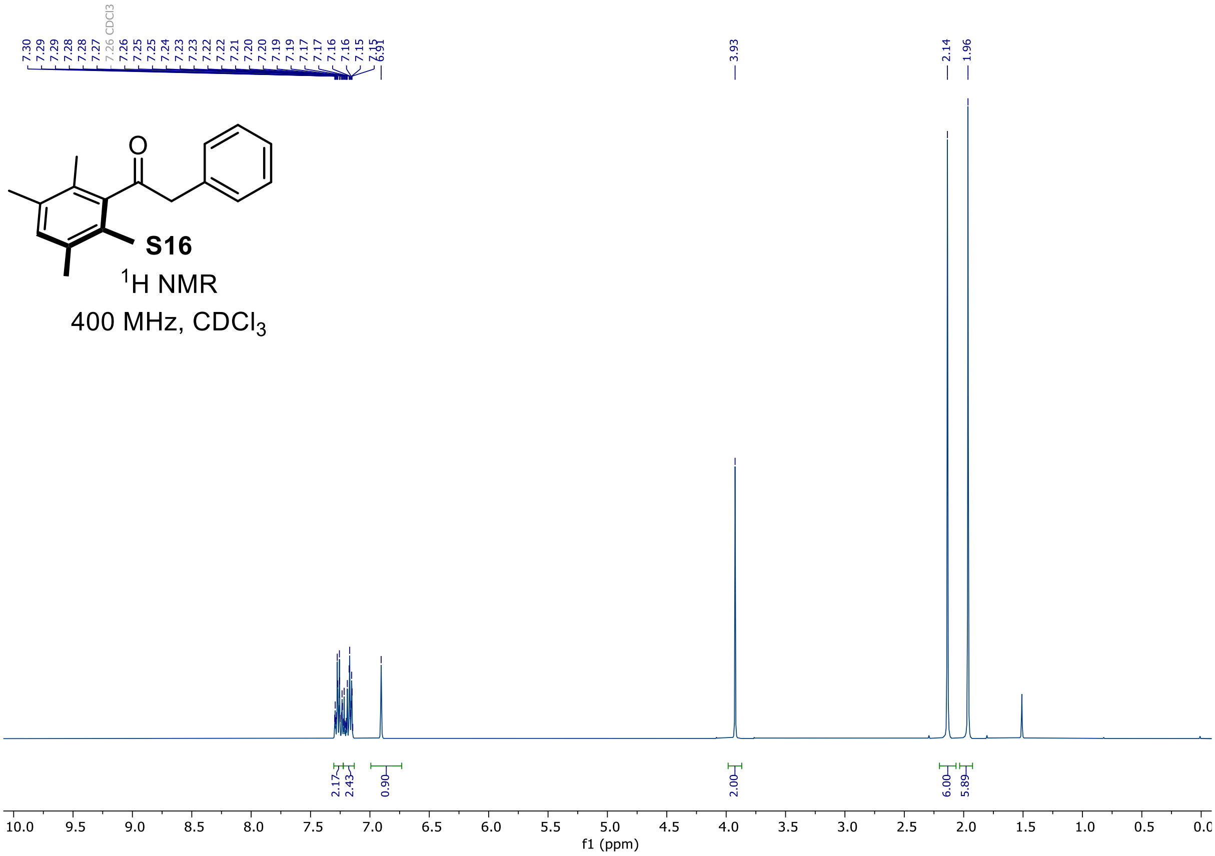

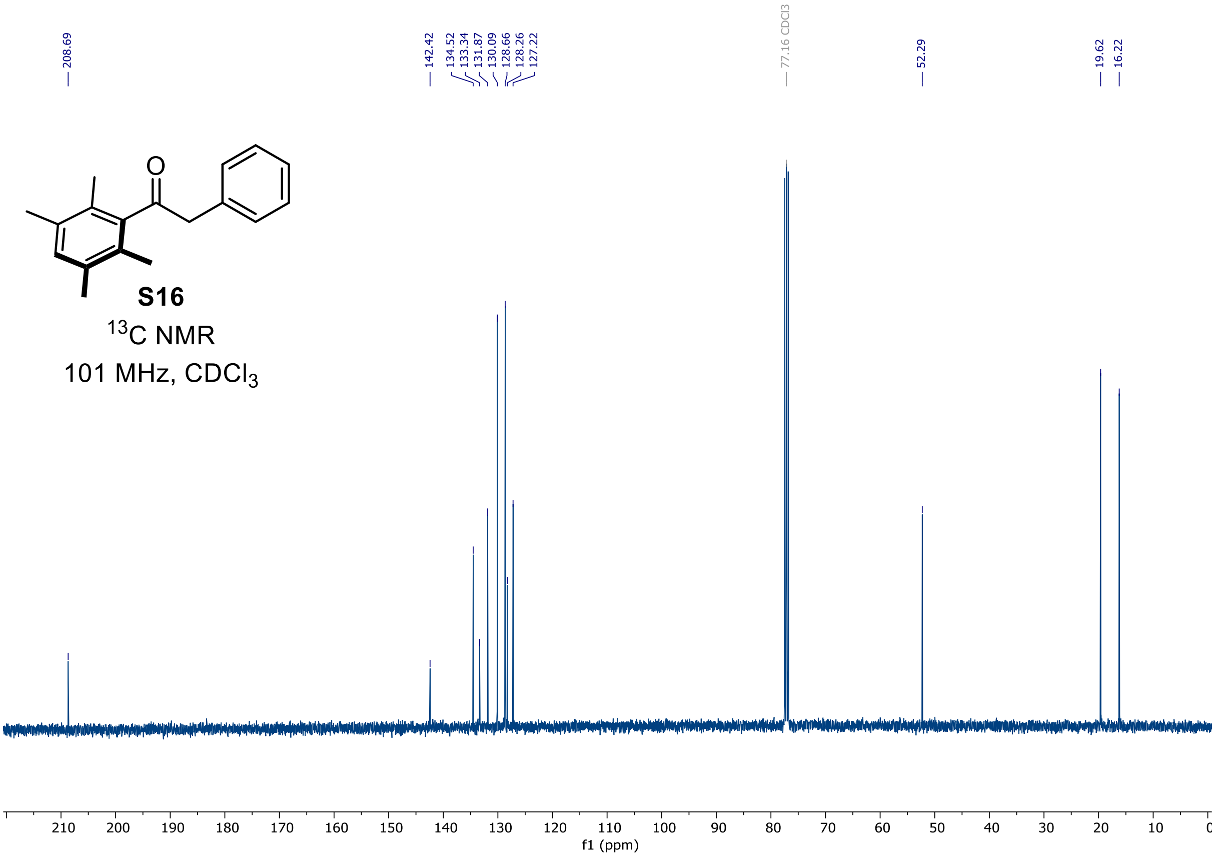

8k (1-Phenylcyclopropyl)(2,3,5,6-tetramethylphenyl)methanone

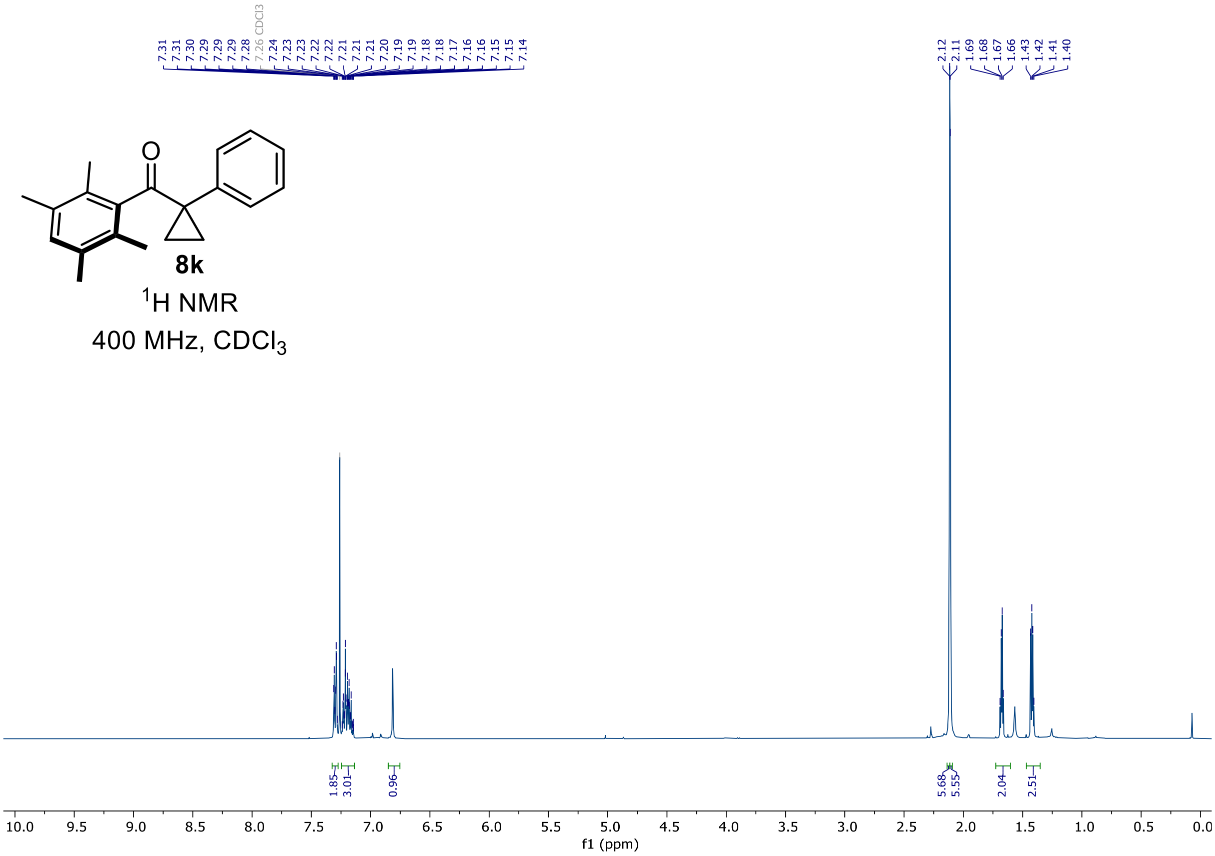

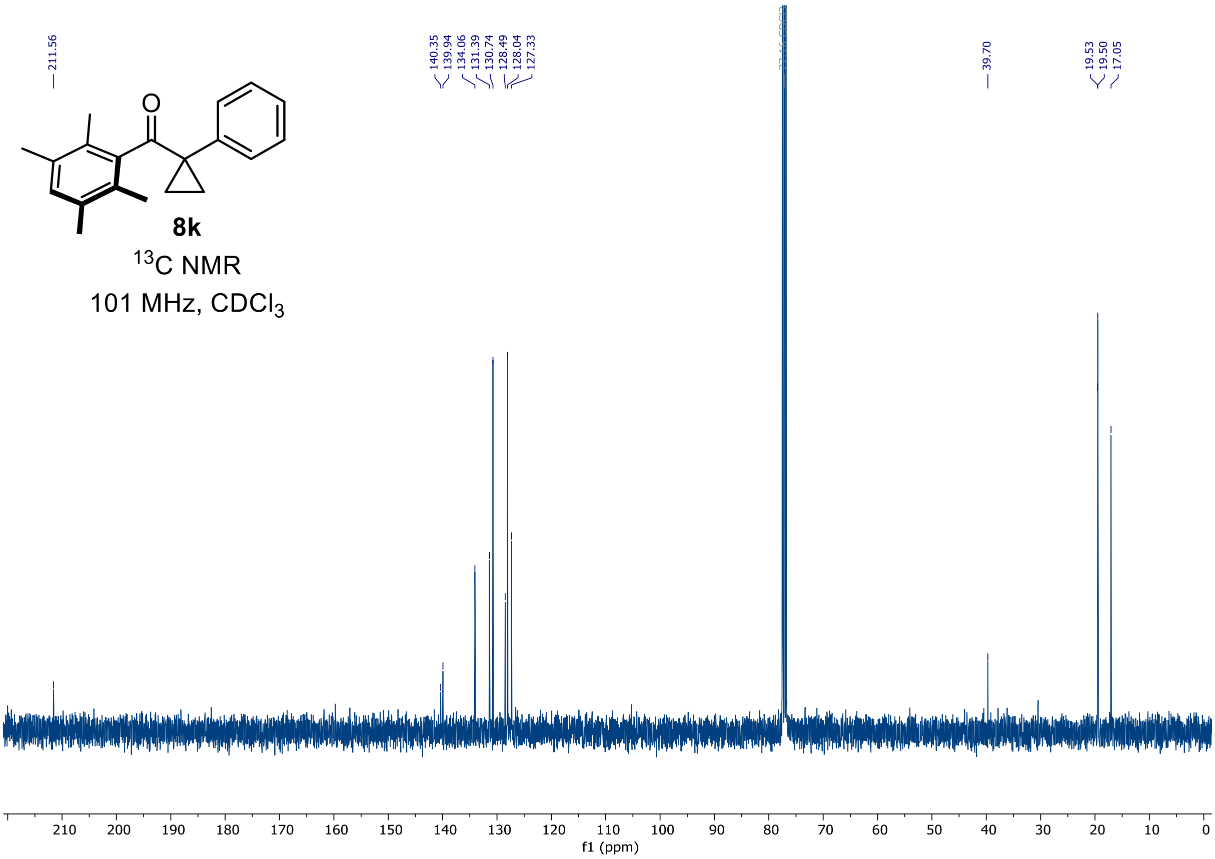

8k' 4-Phenyl-5-(2,3,5,6-tetramethylphenyl)-2,3-dihydrofuran

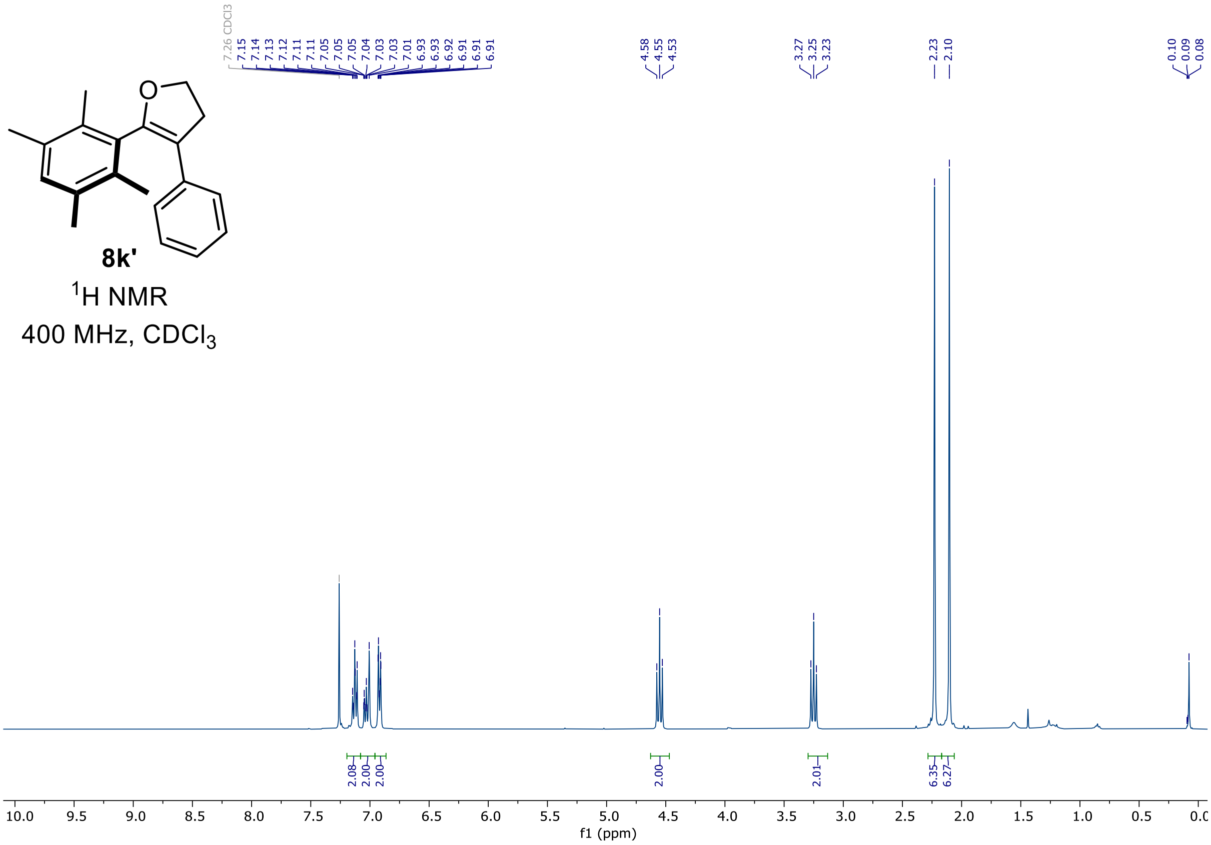

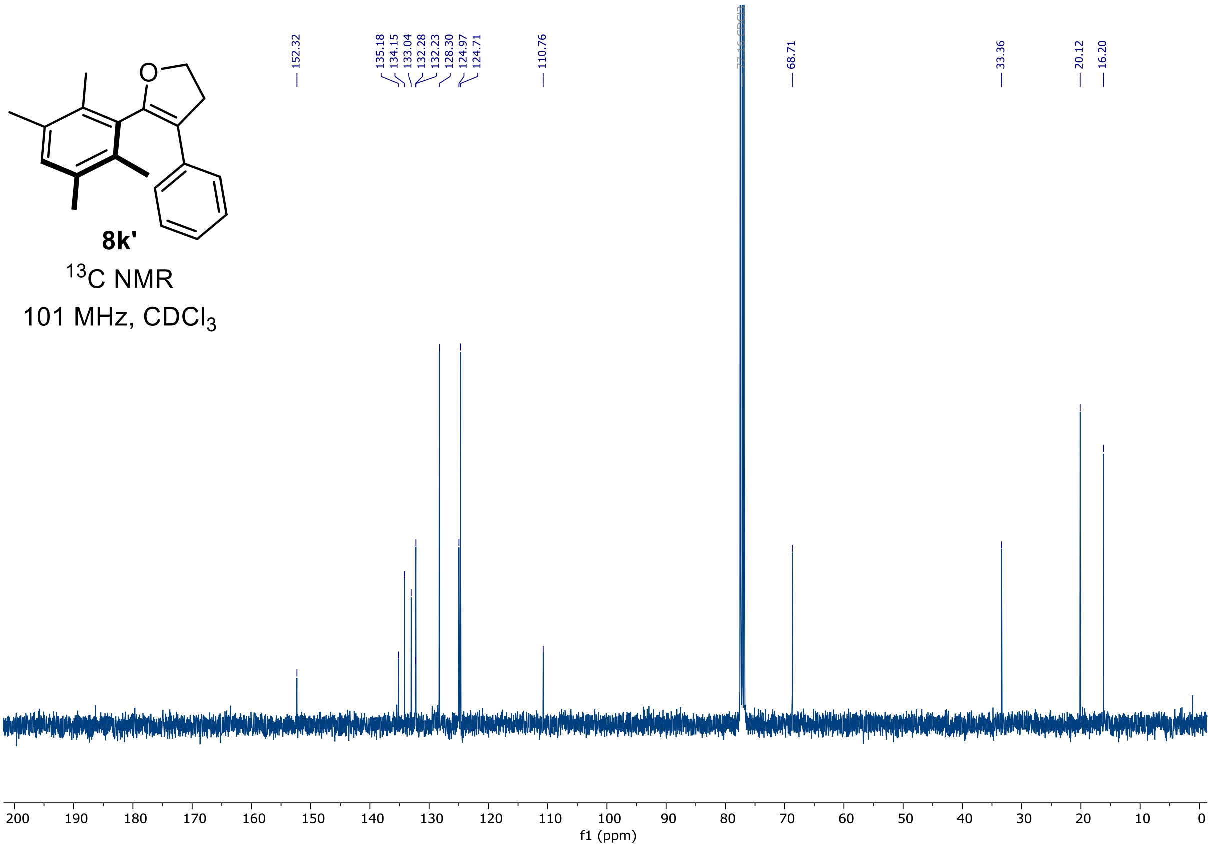

S17 4-Oxo-4-(2,3,5,6-tetramethylphenyl)butanoic acid

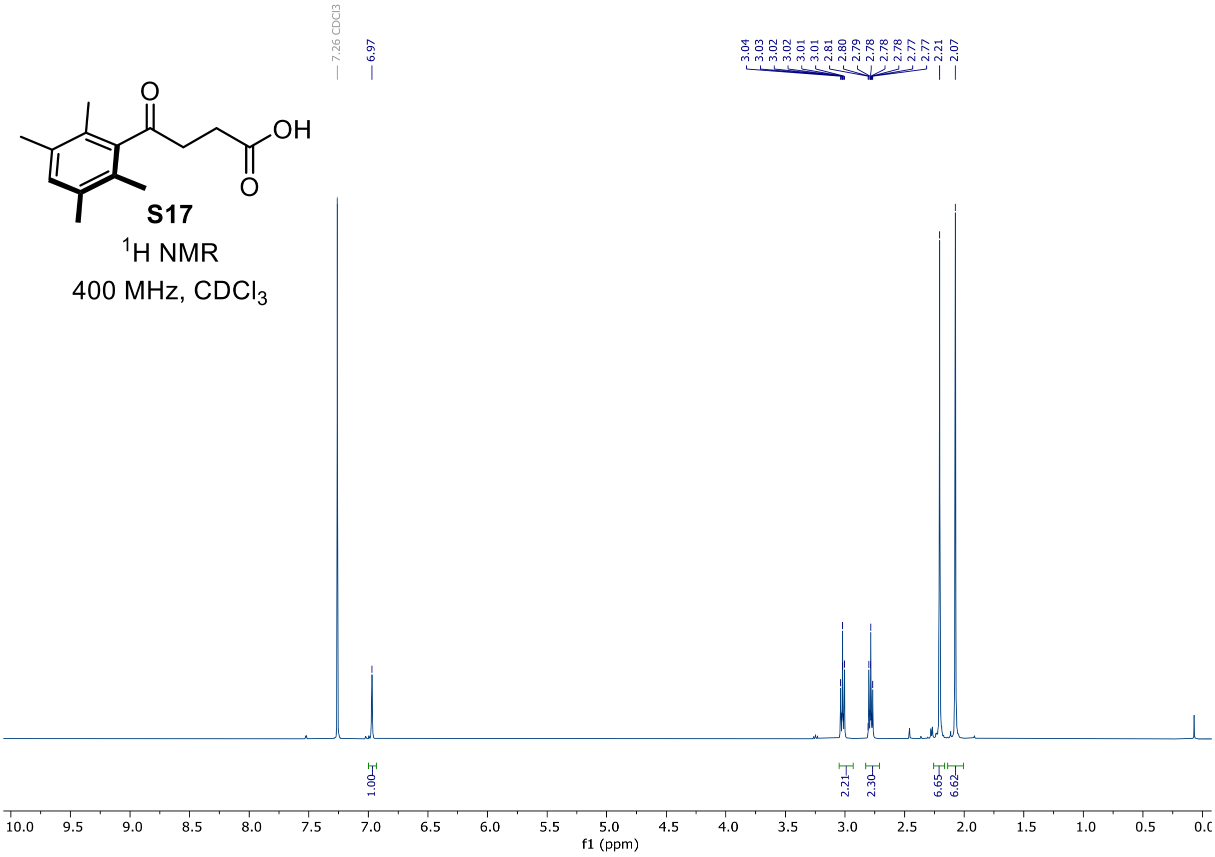

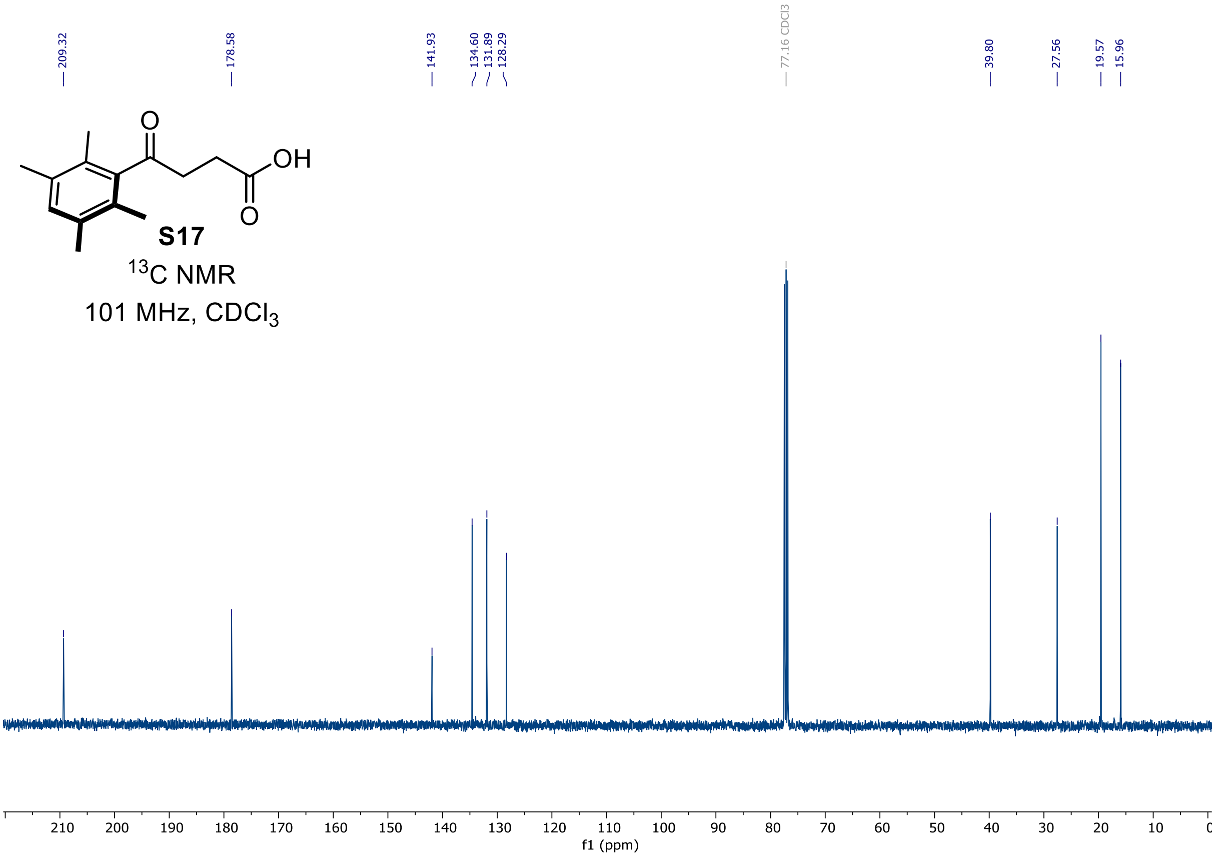

S18 4-Hydroxy-1-(2,3,5,6-tetramethylphenyl)butan-1-one

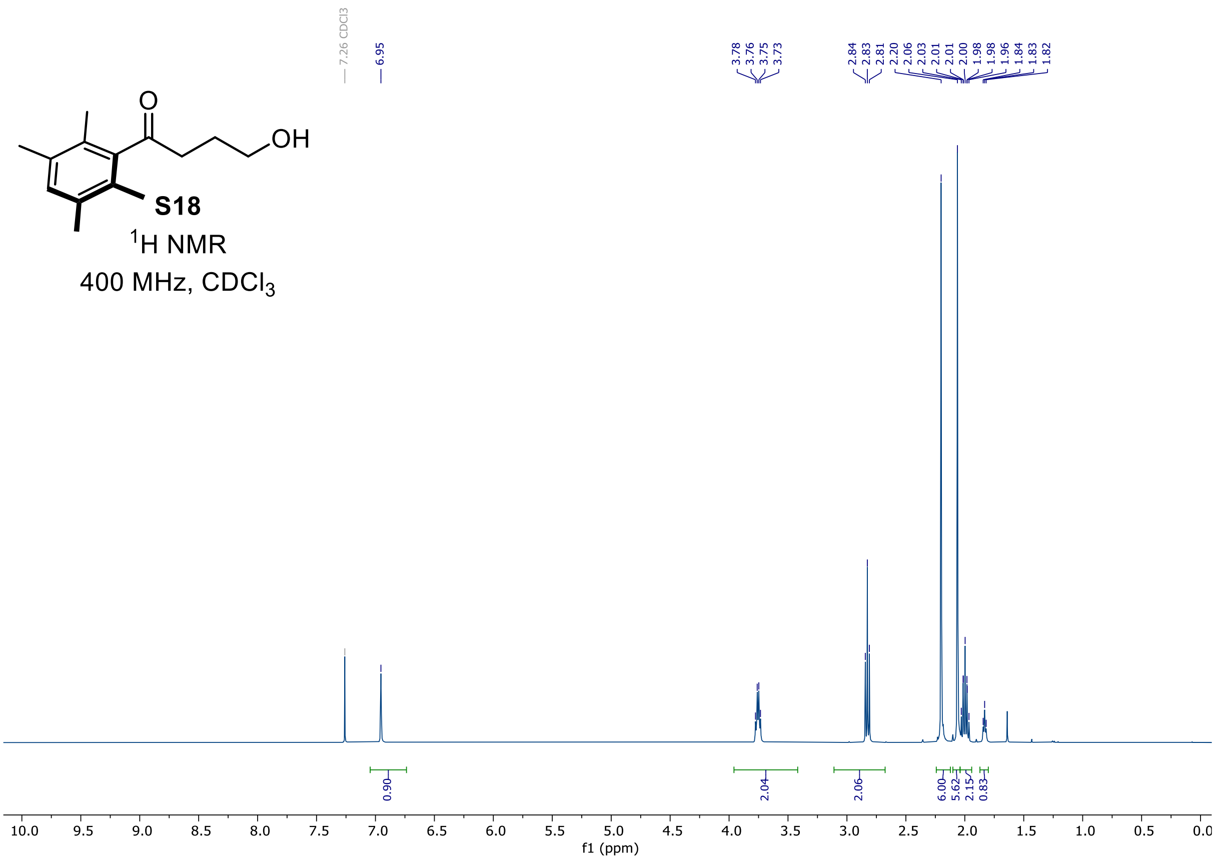

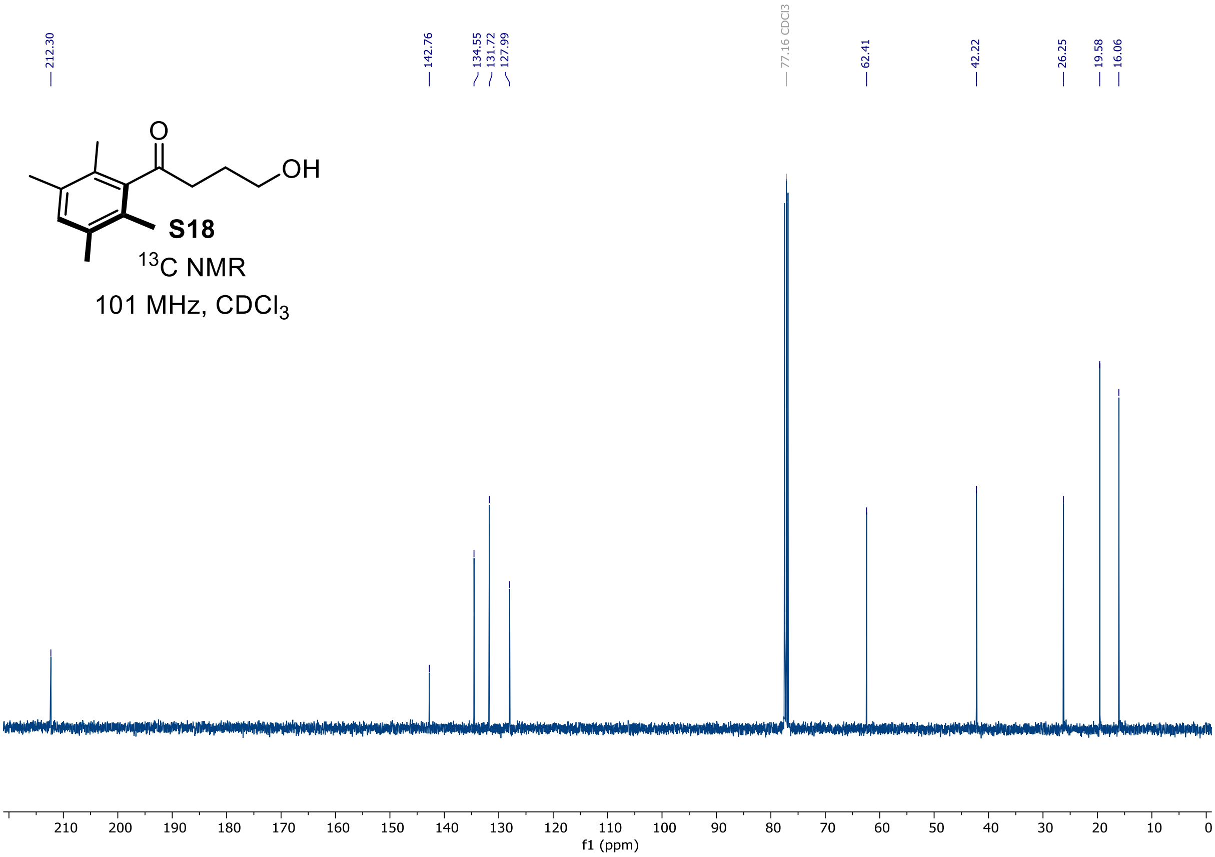

S19 4-((4-Methoxybenzyl)oxy)-1-(2,3,5,6-tetramethylphenyl)butan-1-one

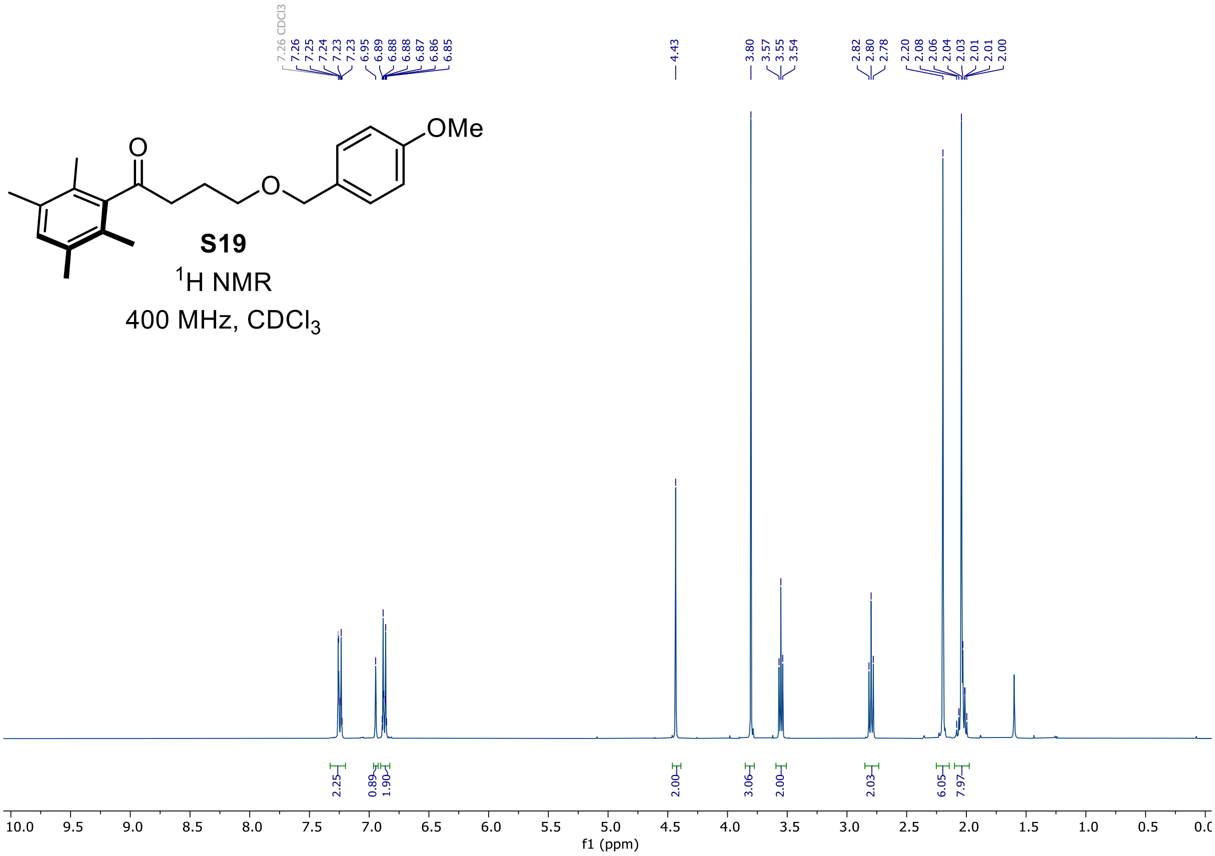

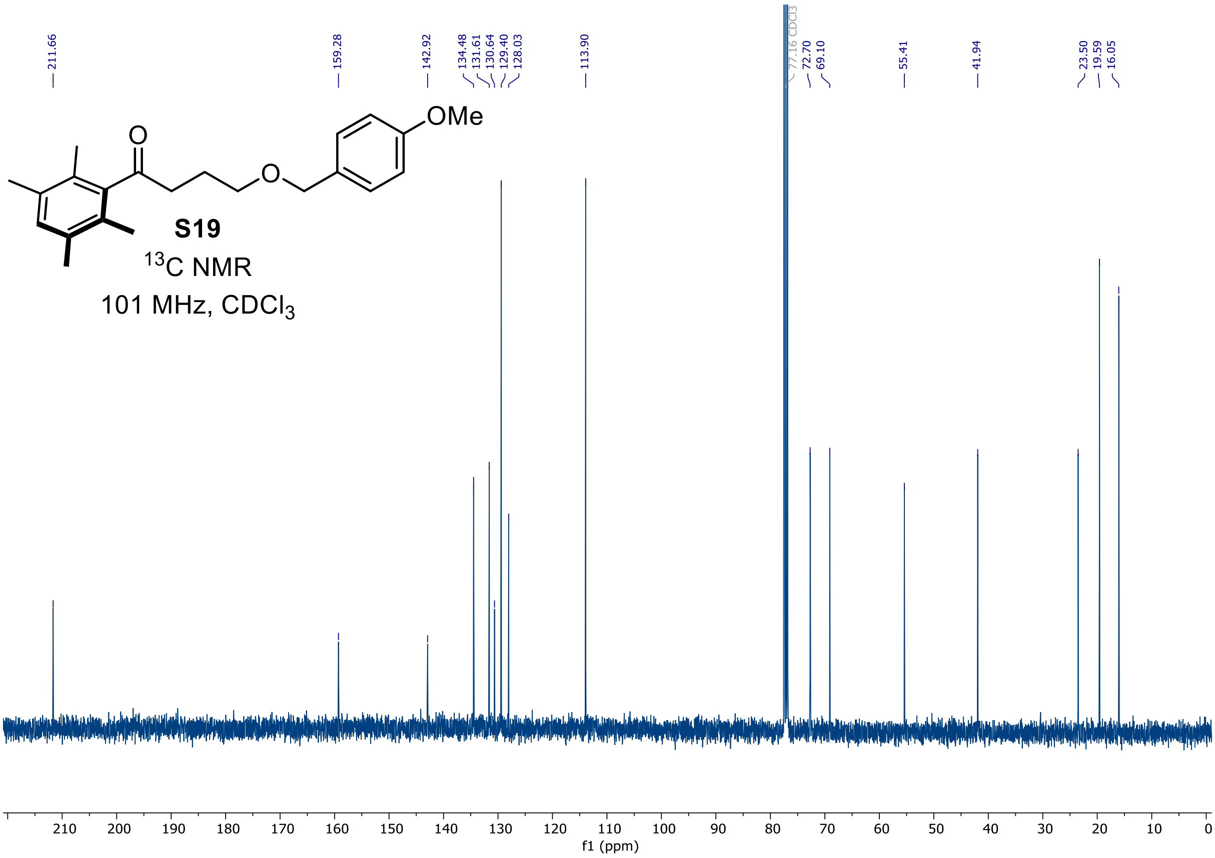

8l (1-(2-((4-Methoxybenzyl)oxy)ethyl)cyclopropyl)(2,3,5,6-tetramethylphenyl)methanone

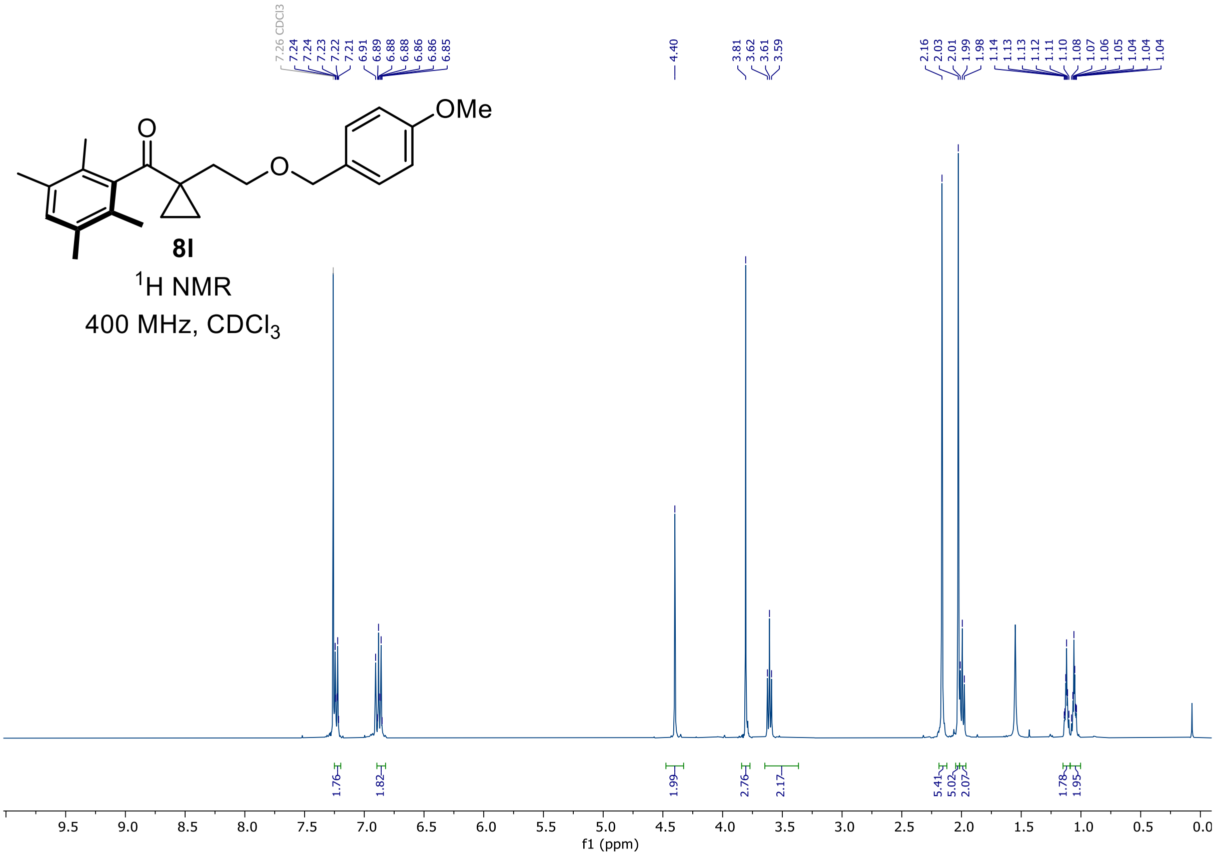

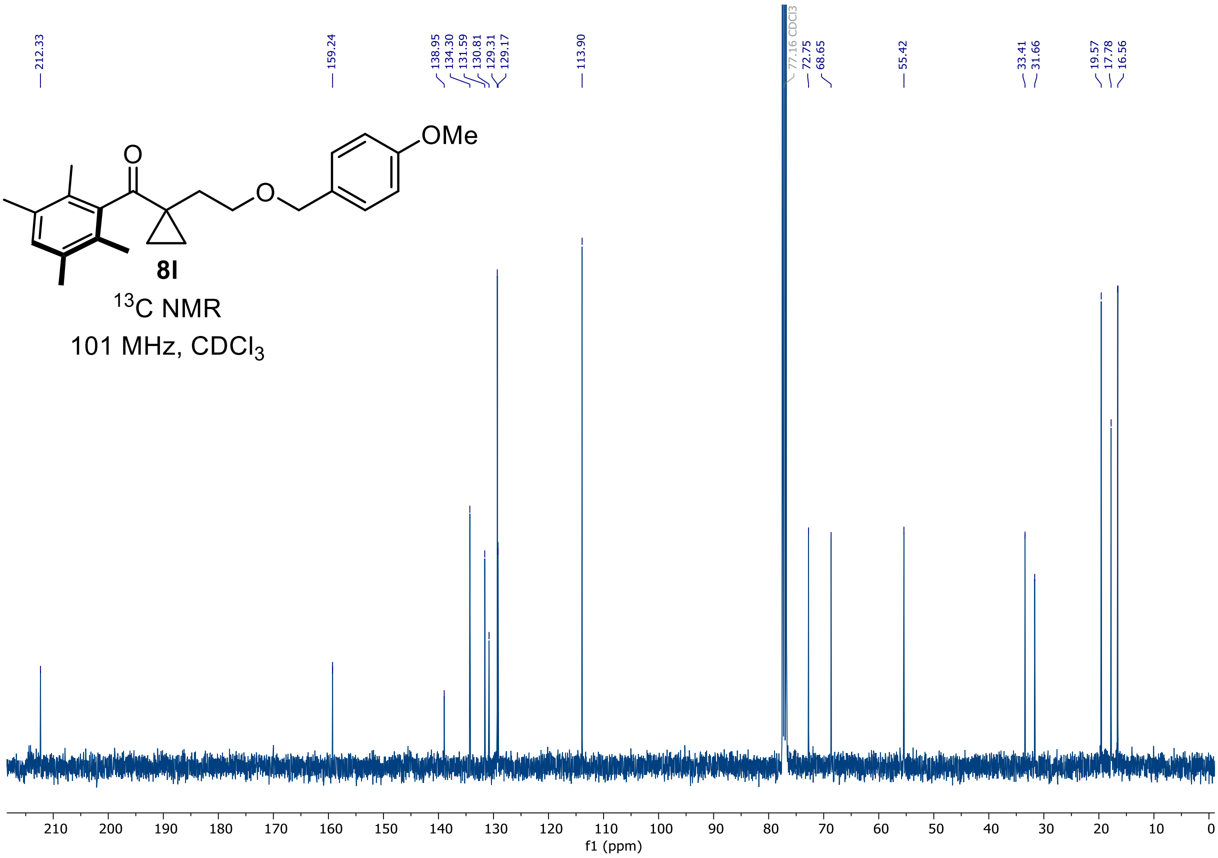

S20 4-Bromo-1-(2,3,5,6-tetramethylphenyl)butan-1-one

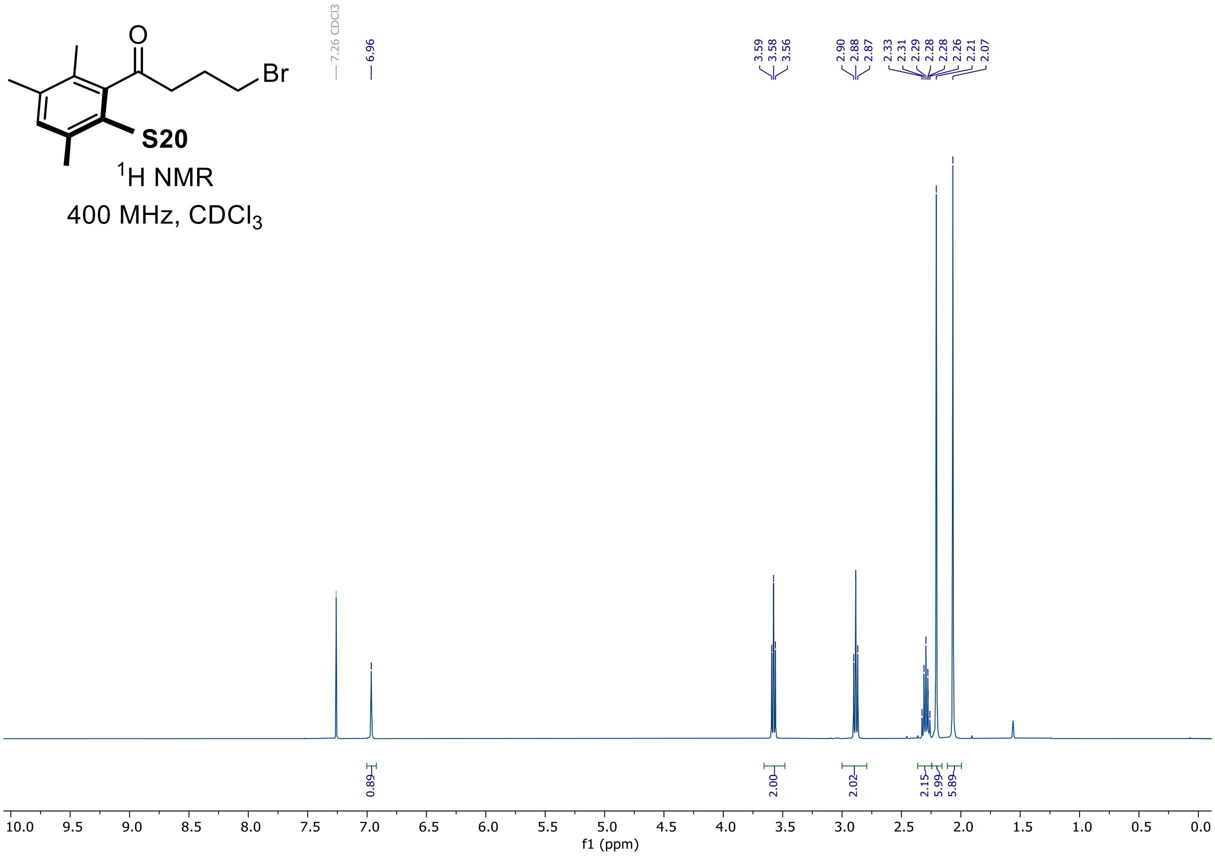

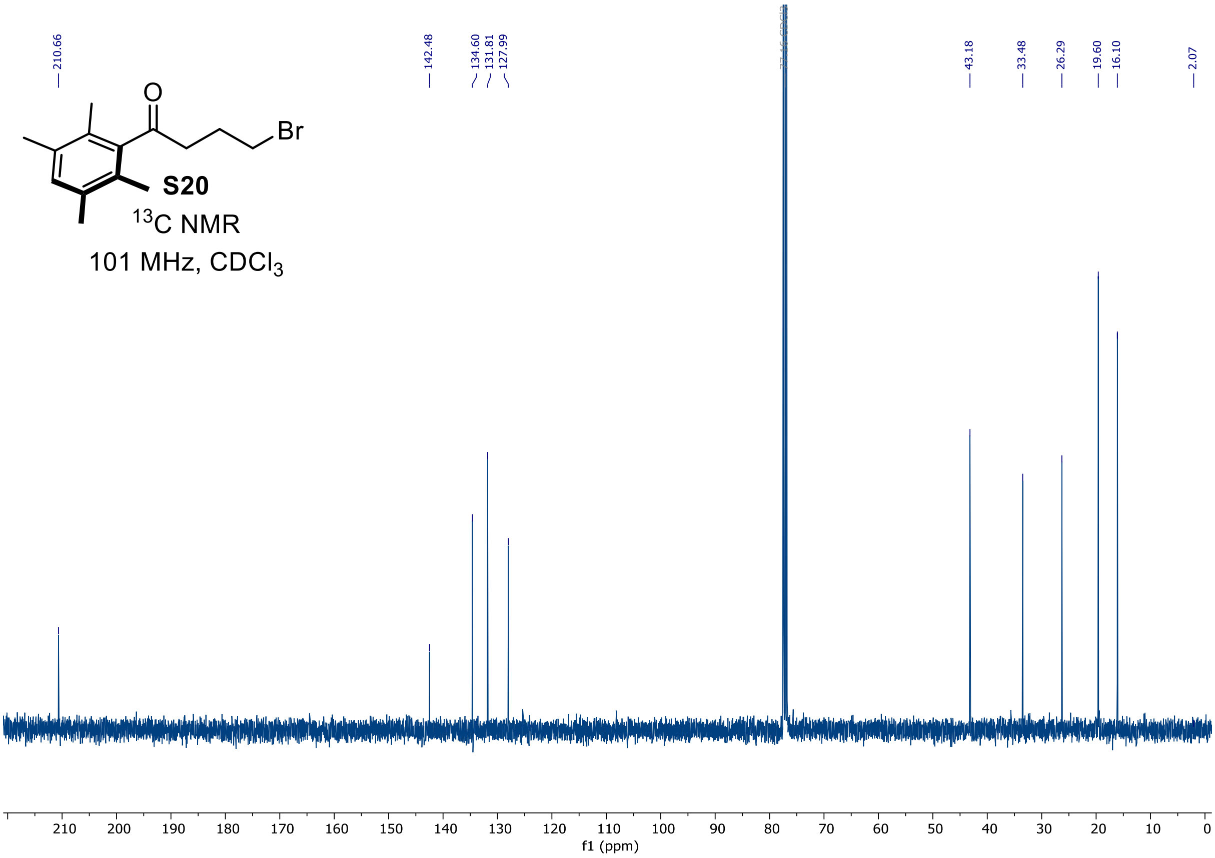

S21 4-(Dibenzylamino)-1-(2,3,5,6-tetramethylphenyl)butan-1-one

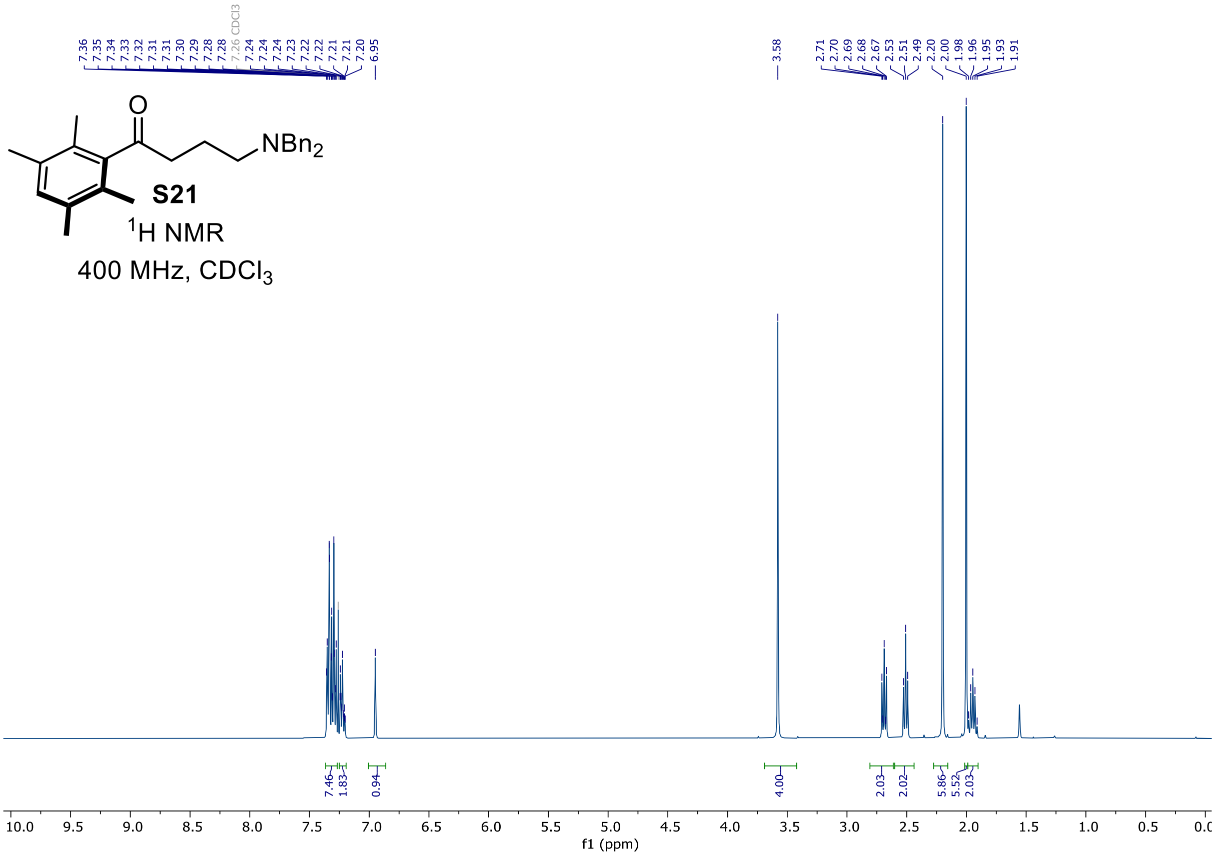

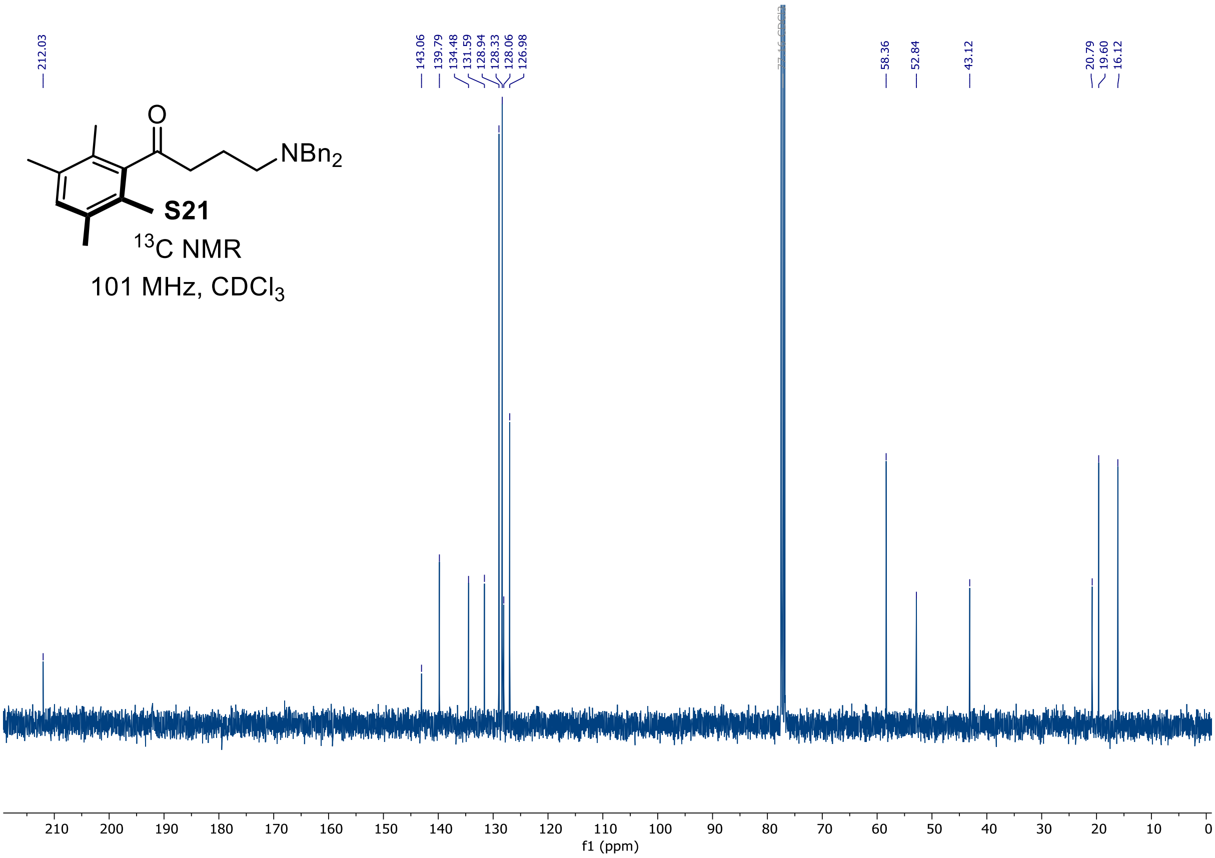

8m (1-(2-(Dibenzylamino)ethyl)cyclopropyl)(2,3,5,6-tetramethylphenyl)methanone

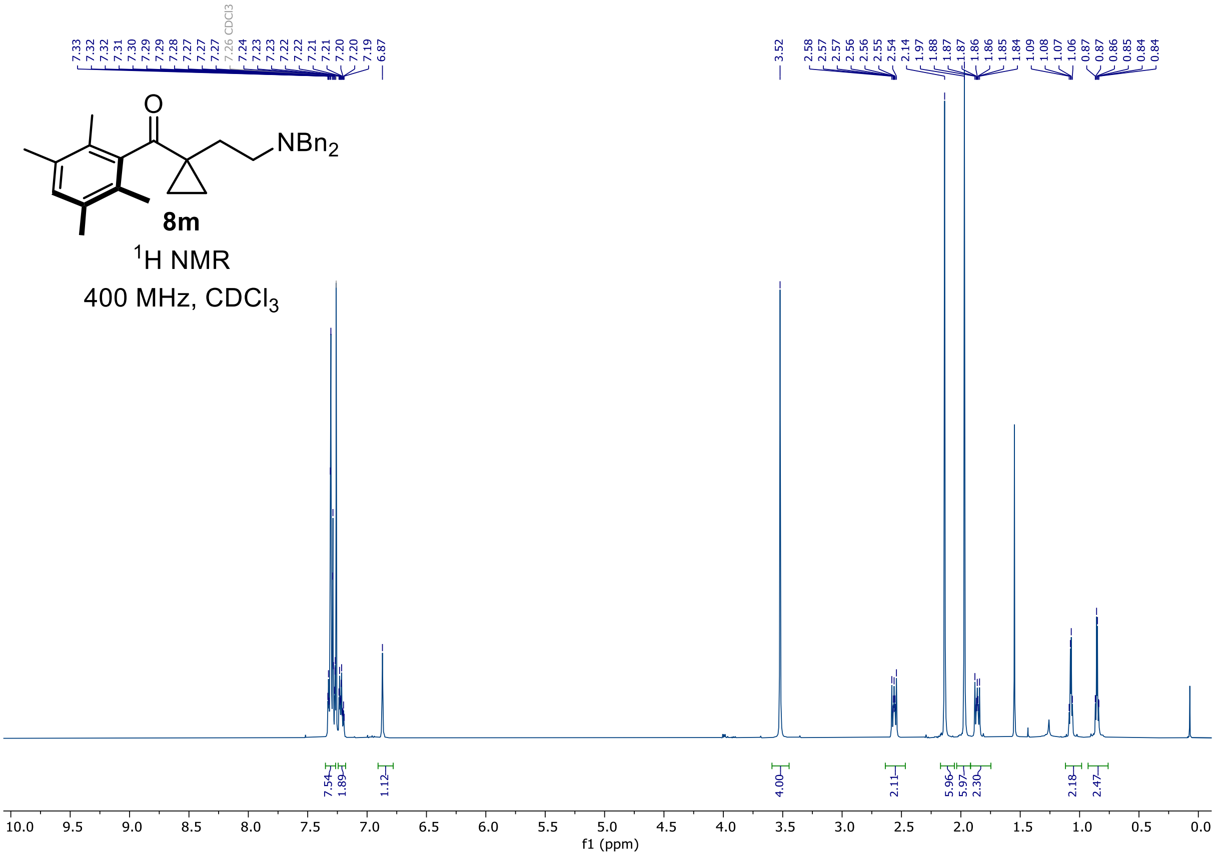

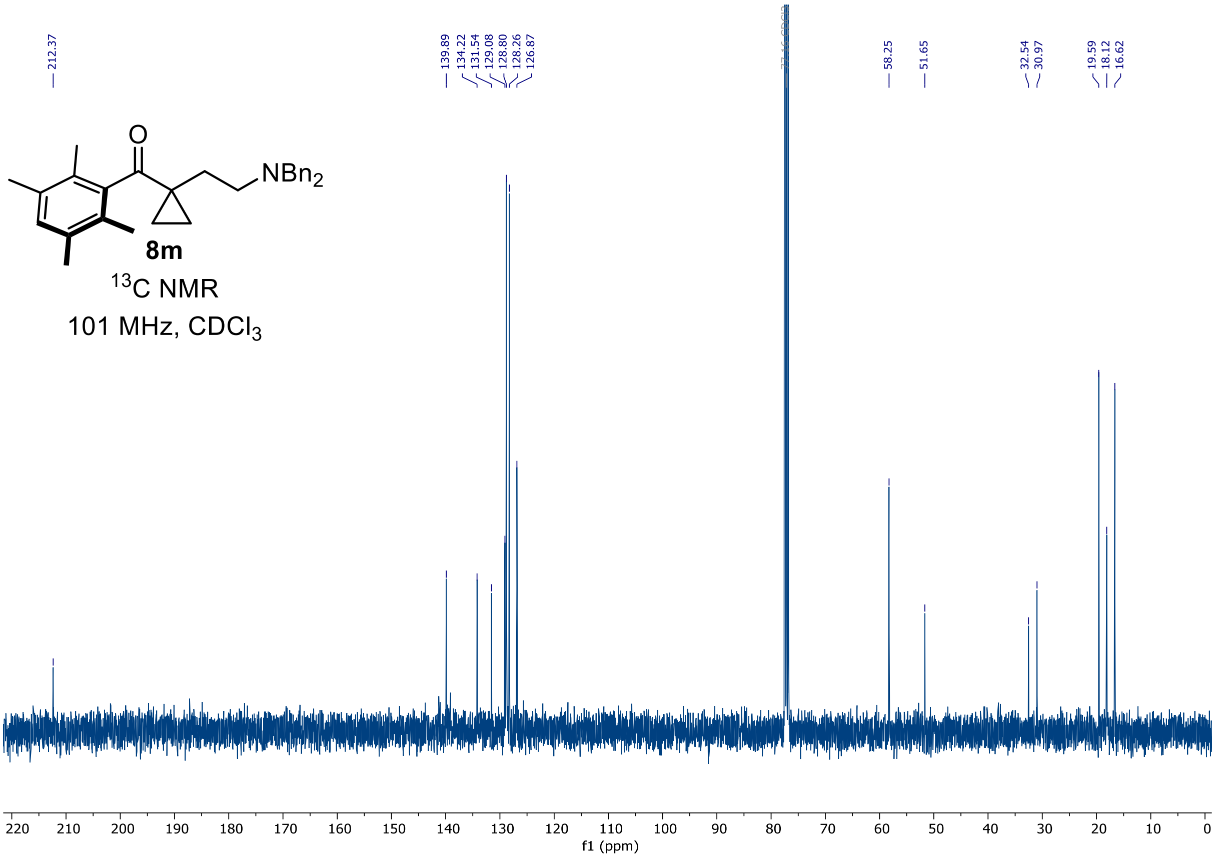

S22 5-Bromo-1-(2,3,5,6-tetramethylphenyl)pentan-1-one

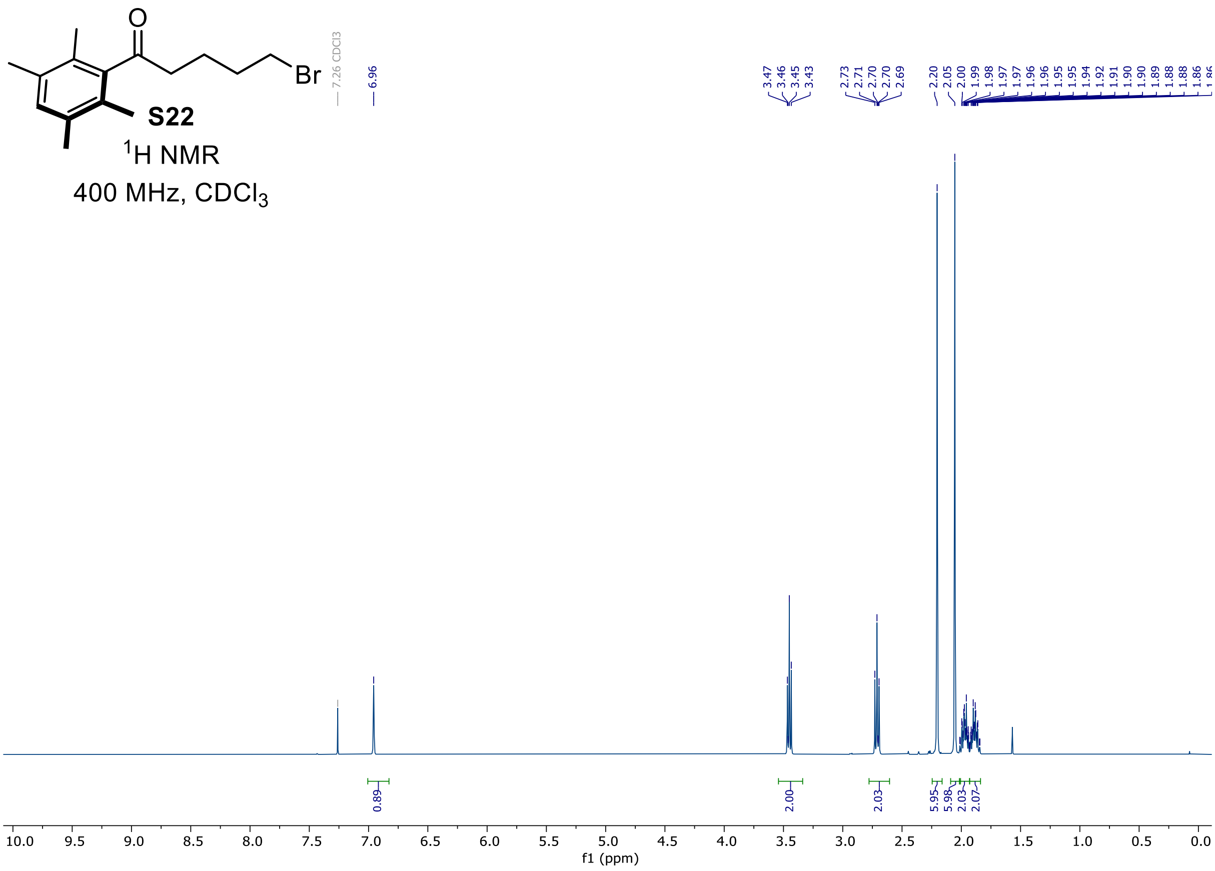

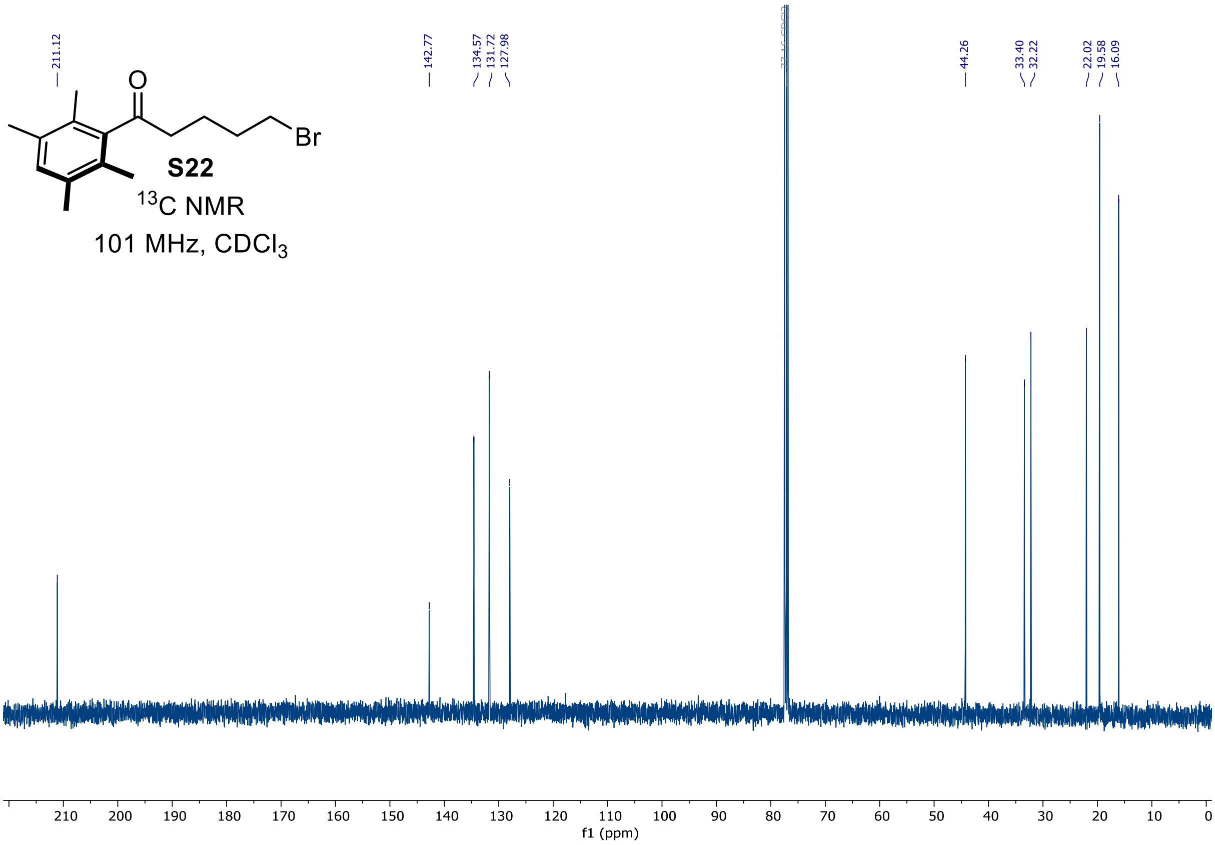

S23 1-(2,3,5,6-Tetramethylphenyl)-5-tosylpentan-1-one

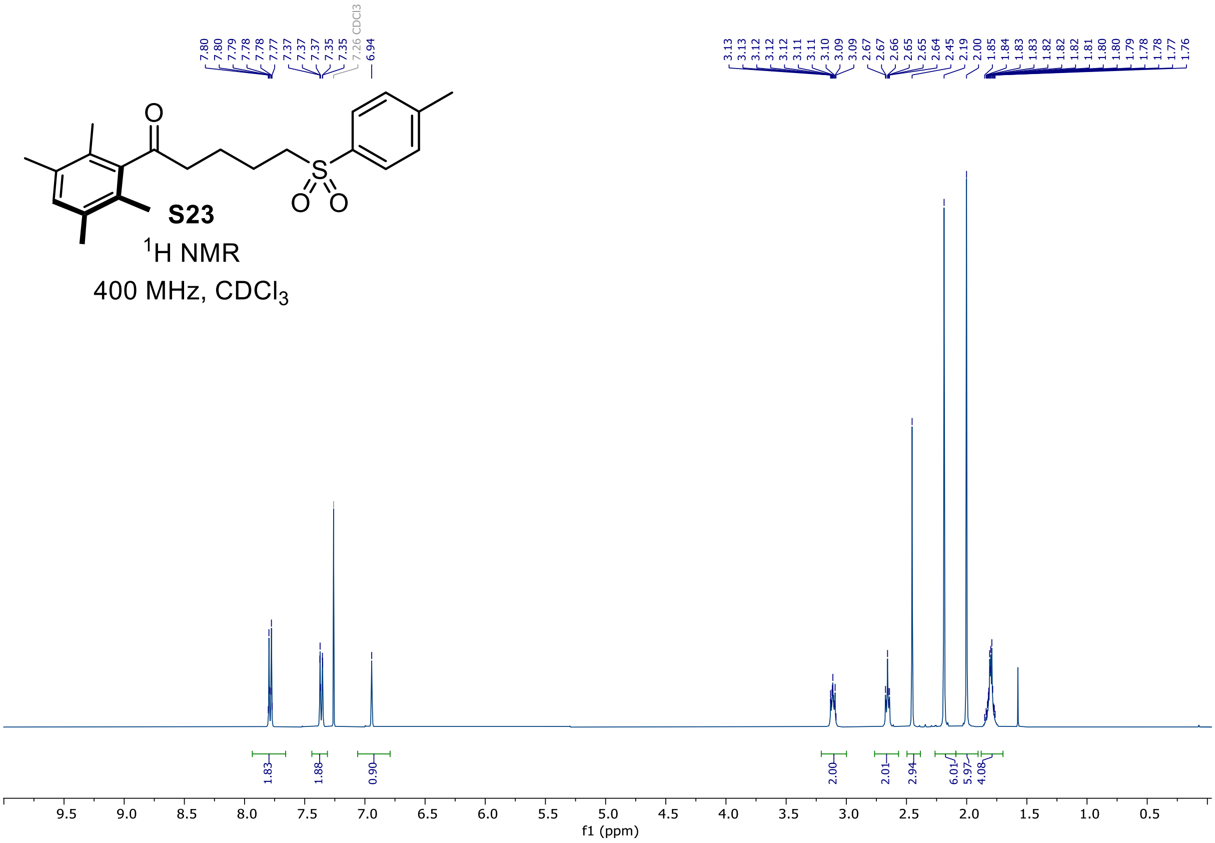

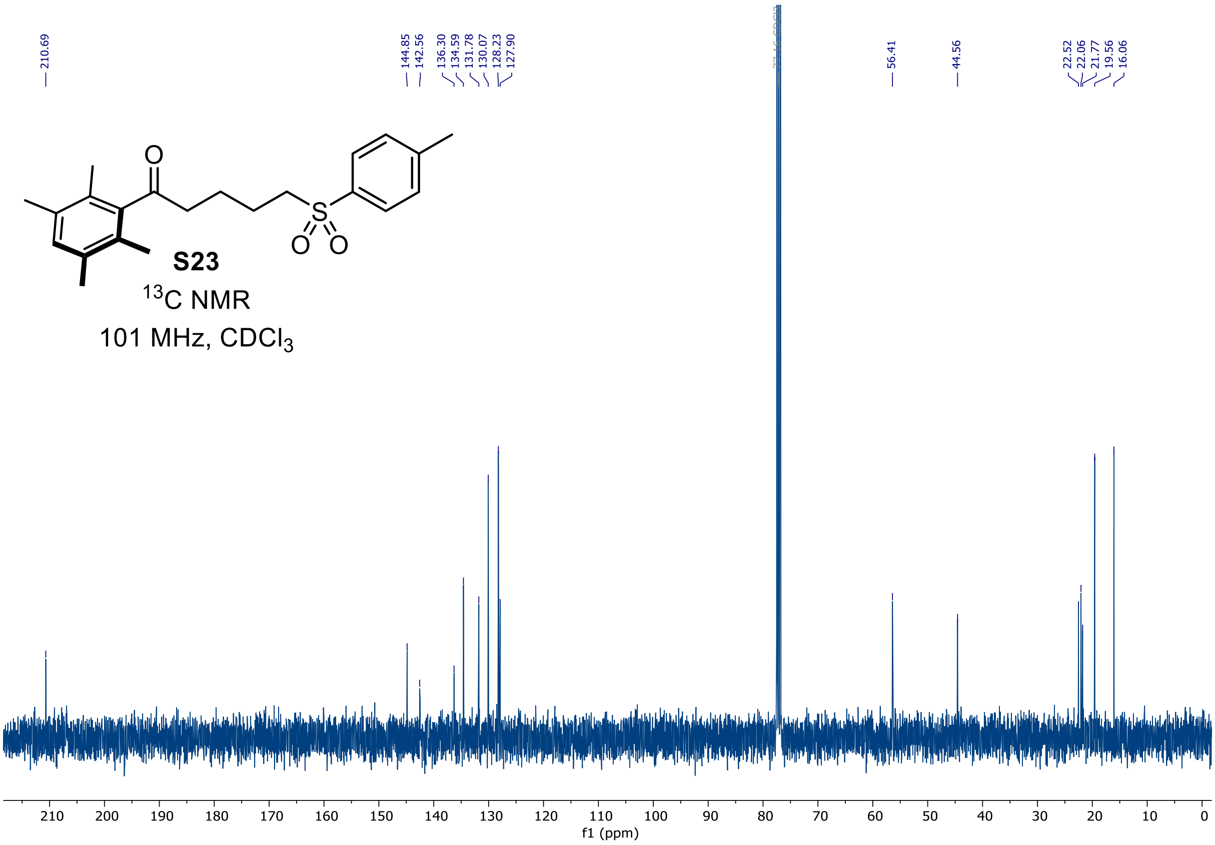

8n (2,3,5,6-Tetramethylphenyl)(1-(3-tosylpropyl)cyclopropyl)methanone

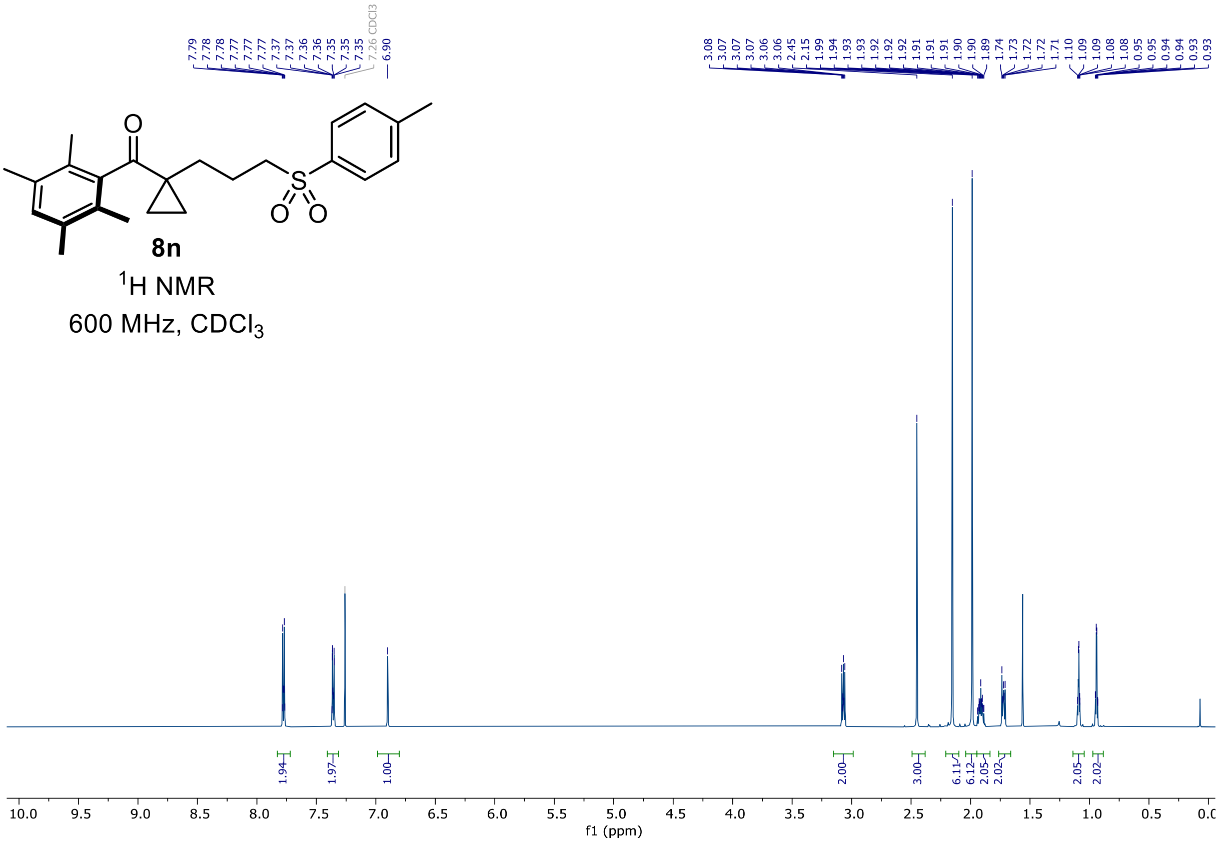

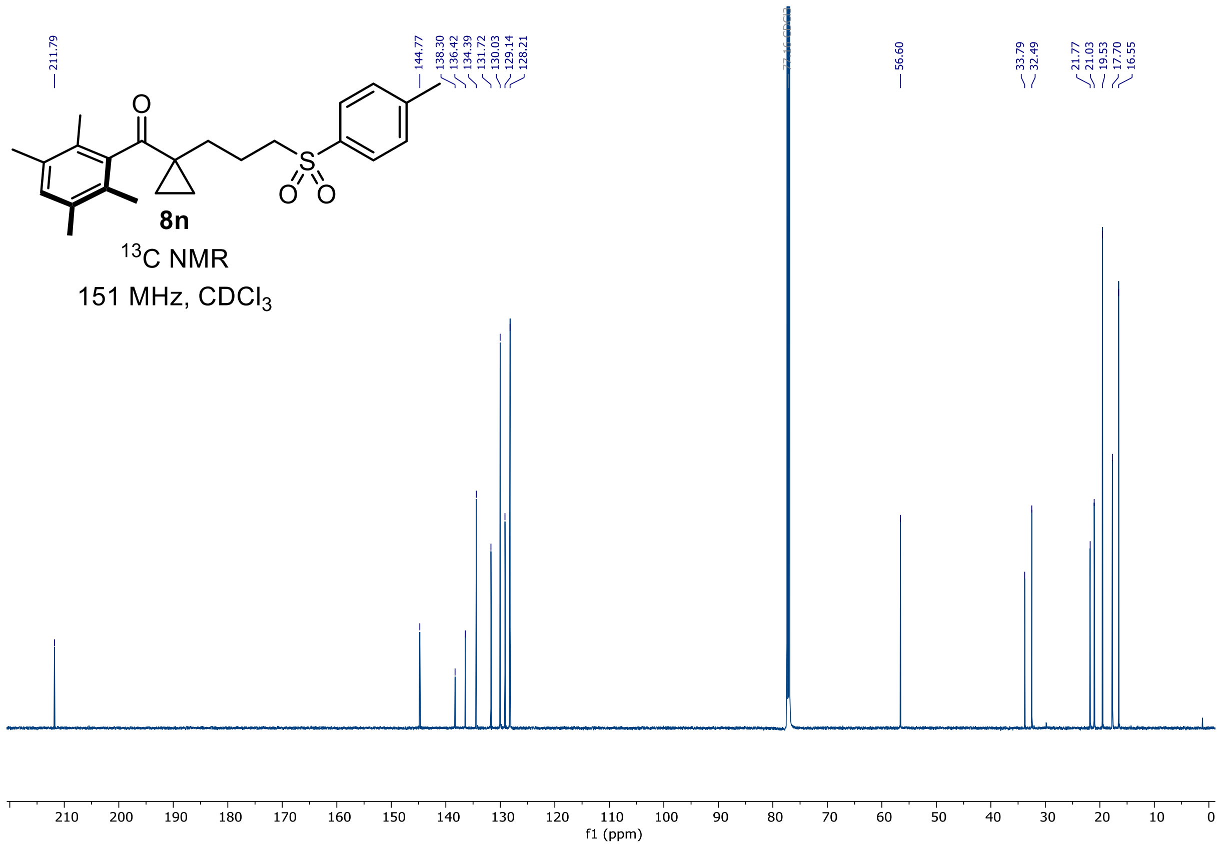

14 2-Benzyl-4-(4-methoxyphenoxy)-1-(2,3,4,5,6-pentamethylphenyl)butan-1-one

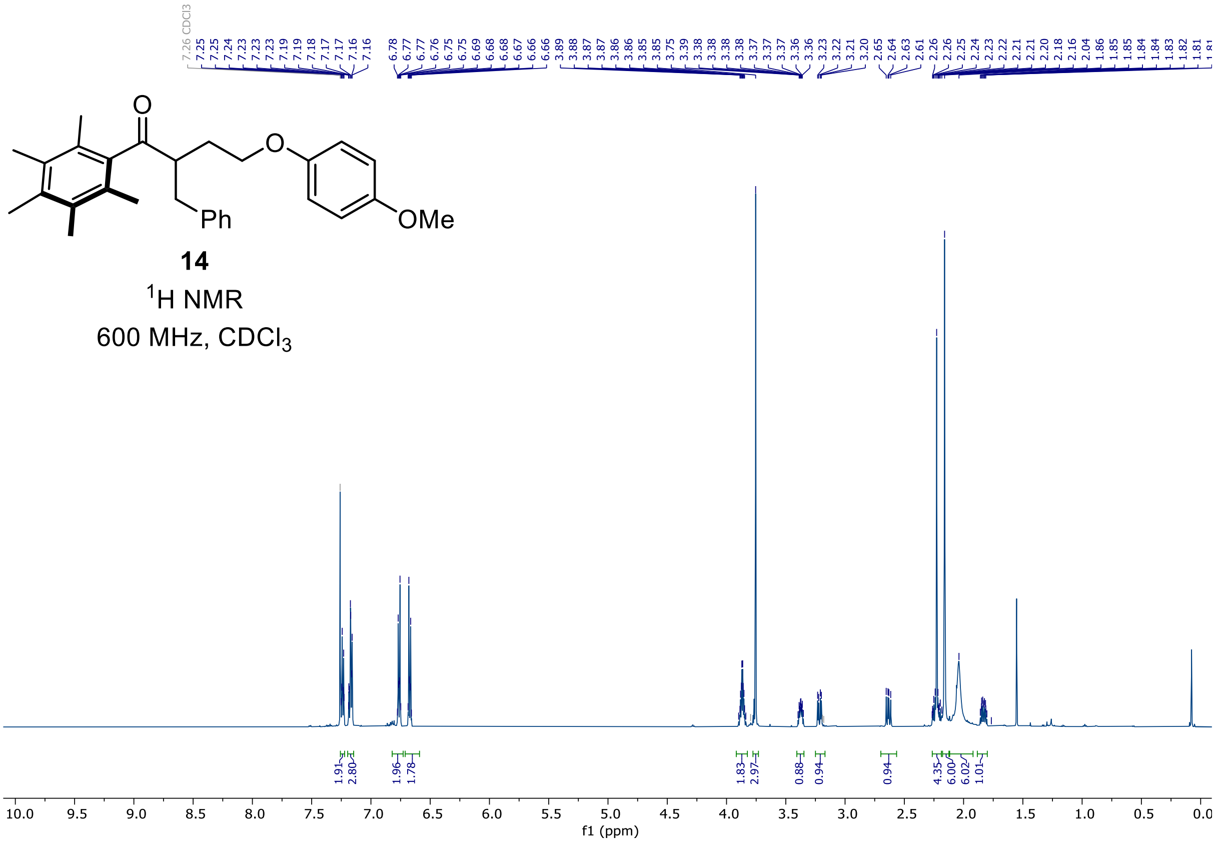

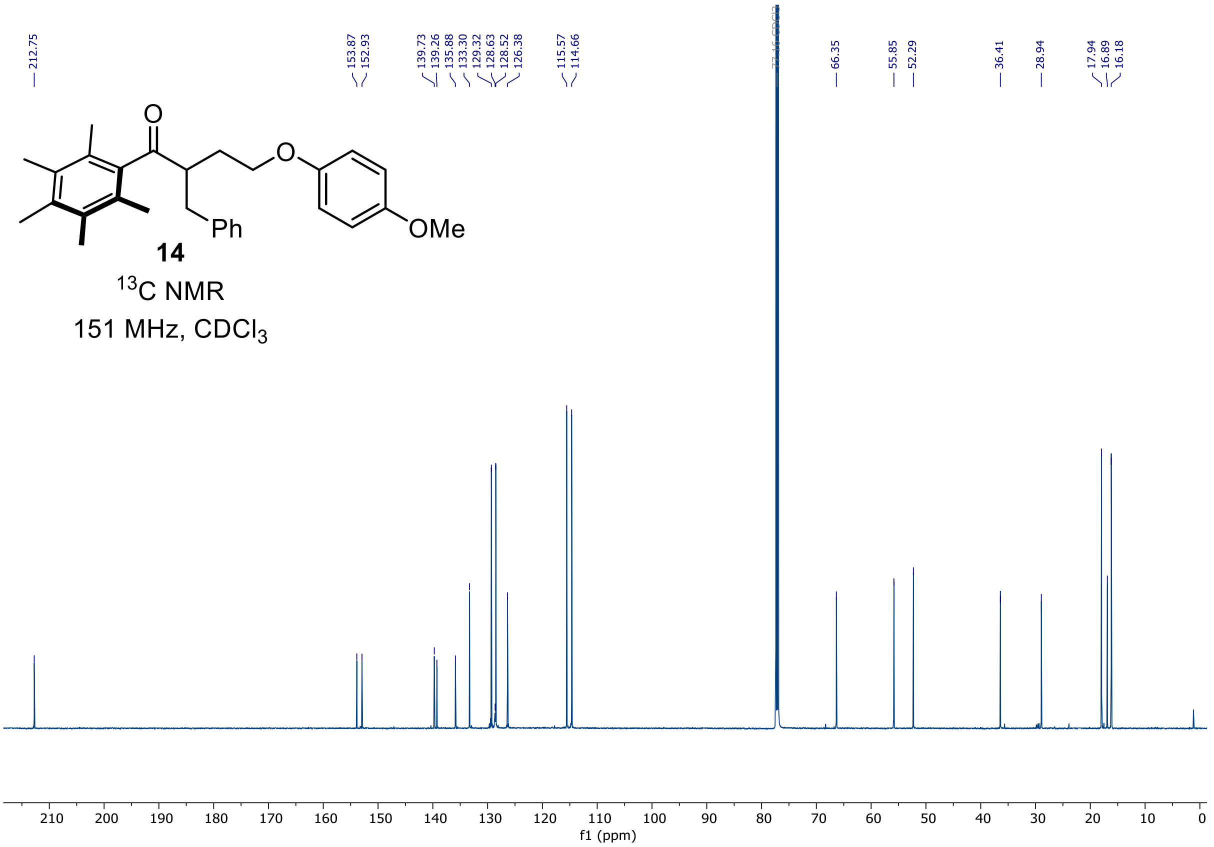

Derivatisation

15a (1-Butylcyclopropyl)(4-hydroxy-2,3,5,6-tetramethylphenyl)methanone

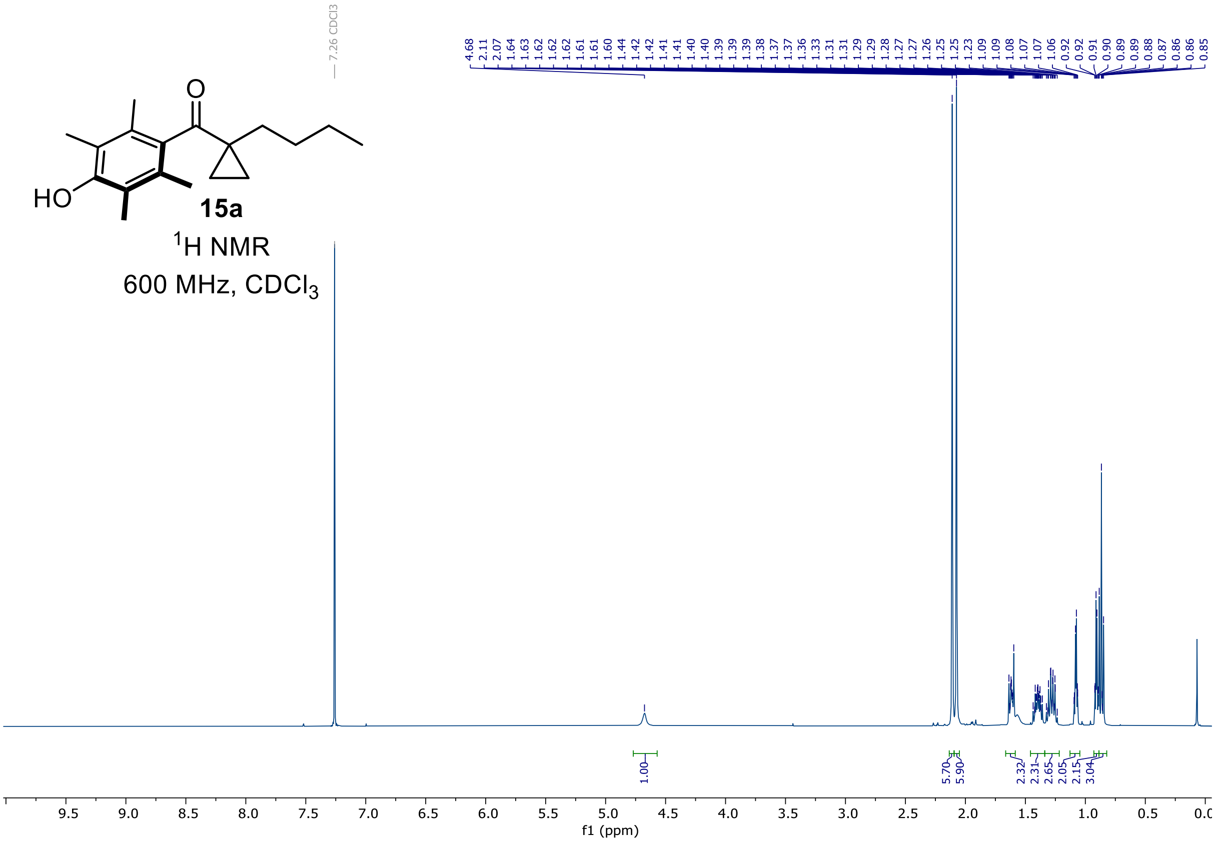

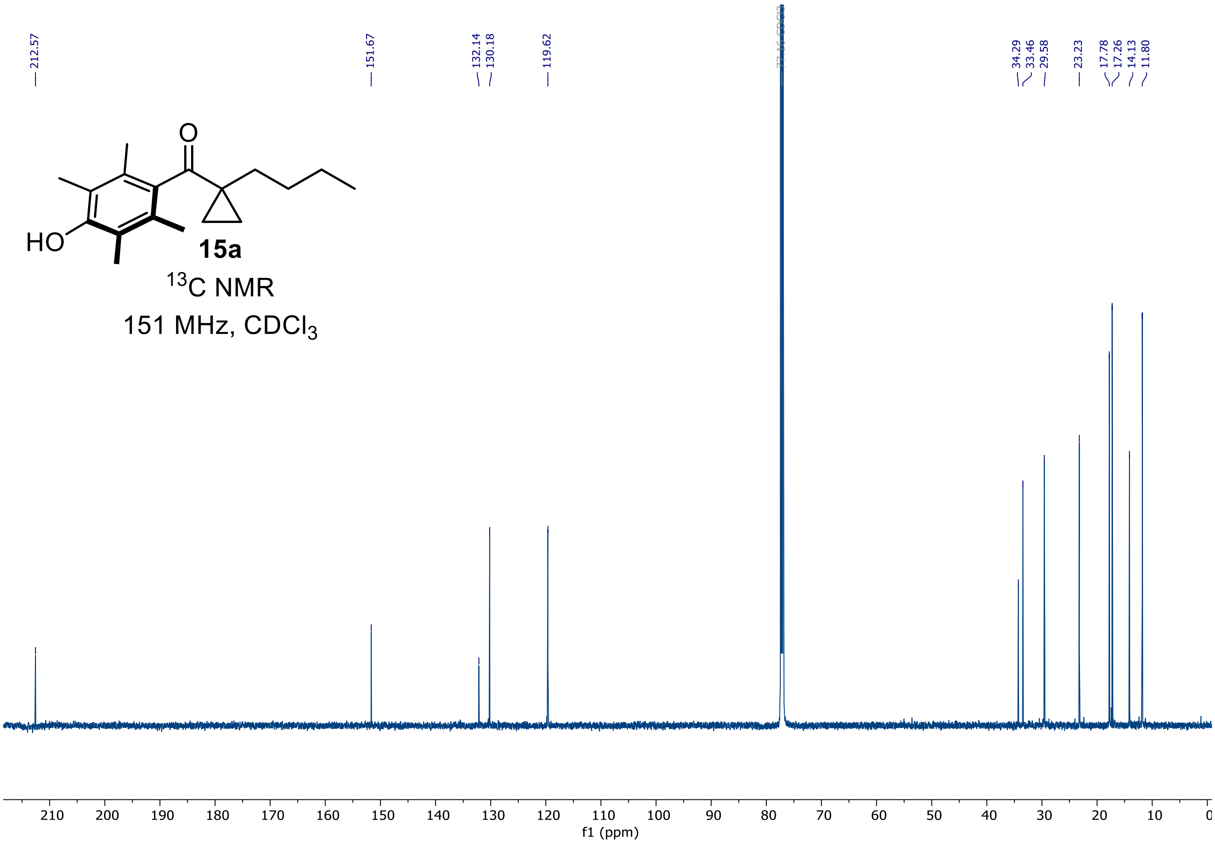

15b (4-Hydroxy-2,3,5,6-tetramethylphenyl)(1-isopentylcyclopropyl)methanone

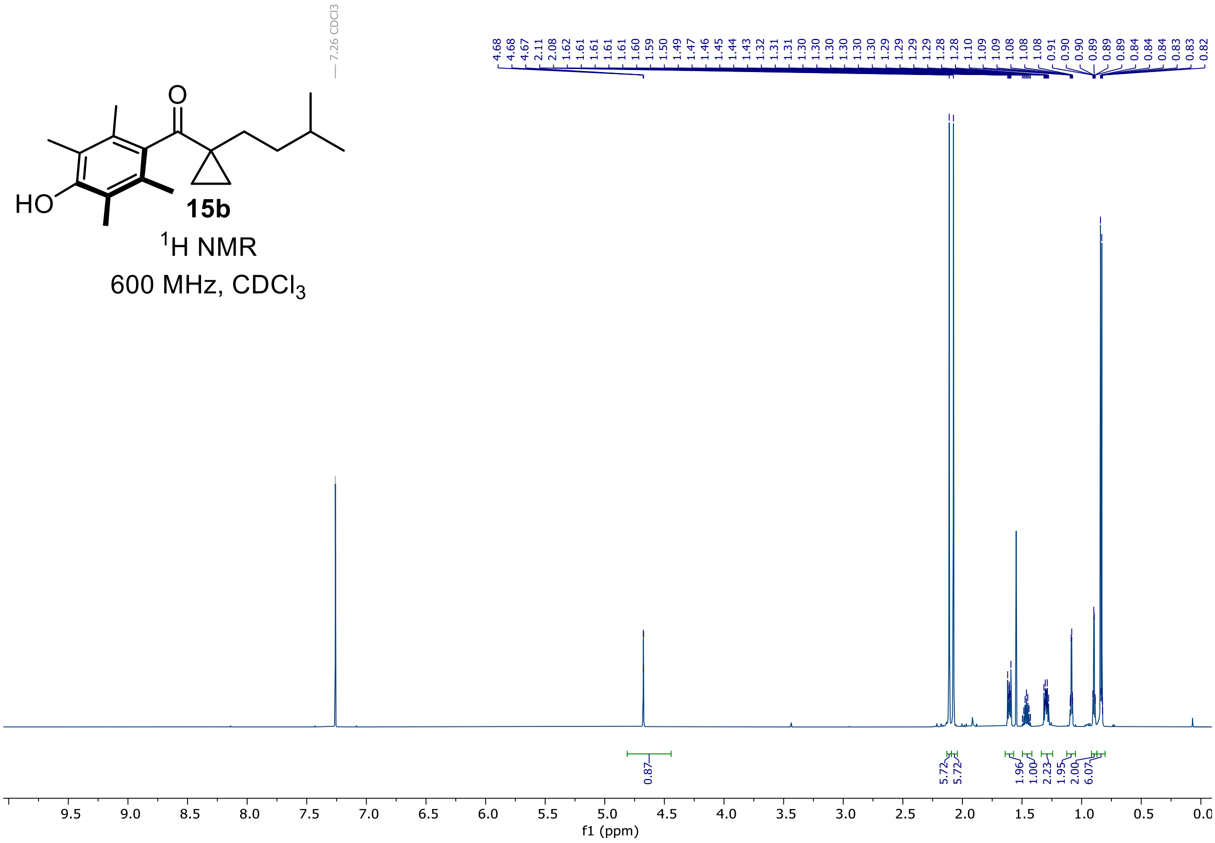

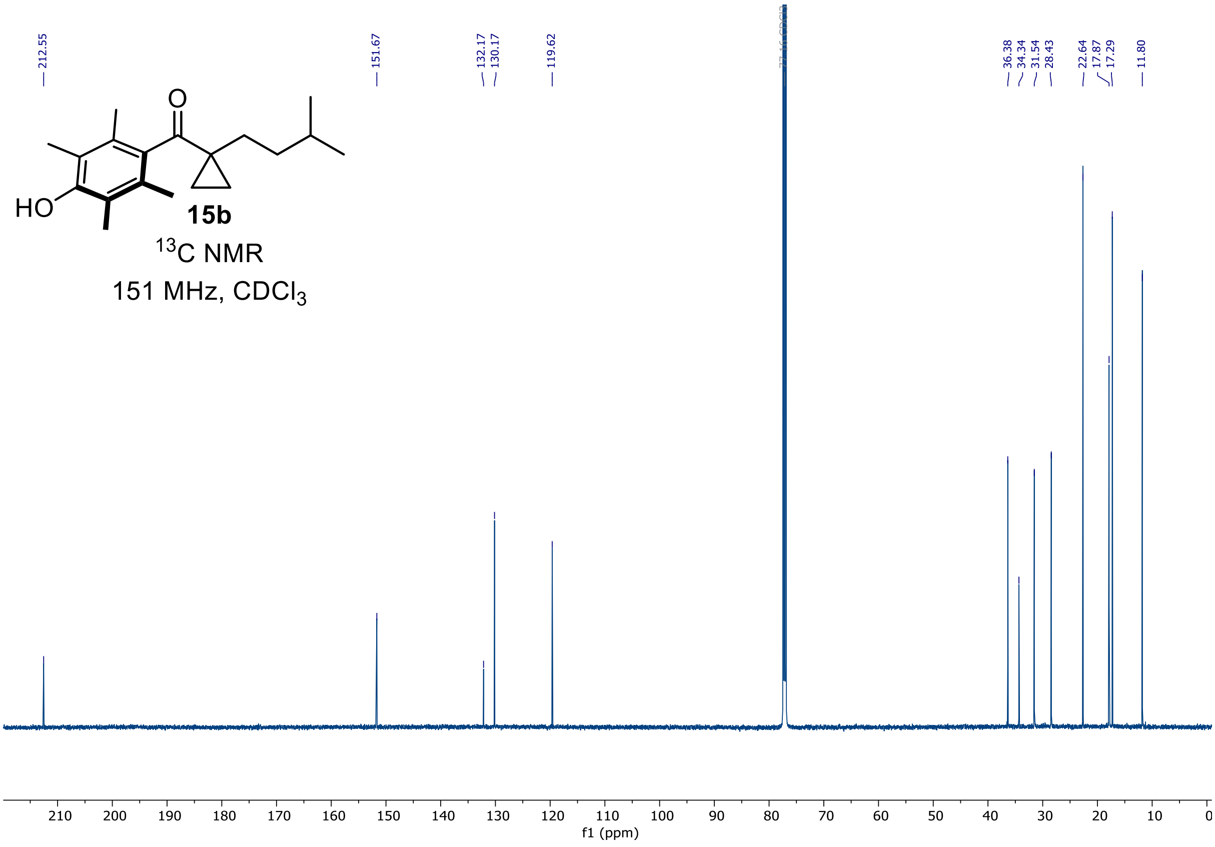

16b 1-Isopentylcyclopropane-1-carboxylic acid

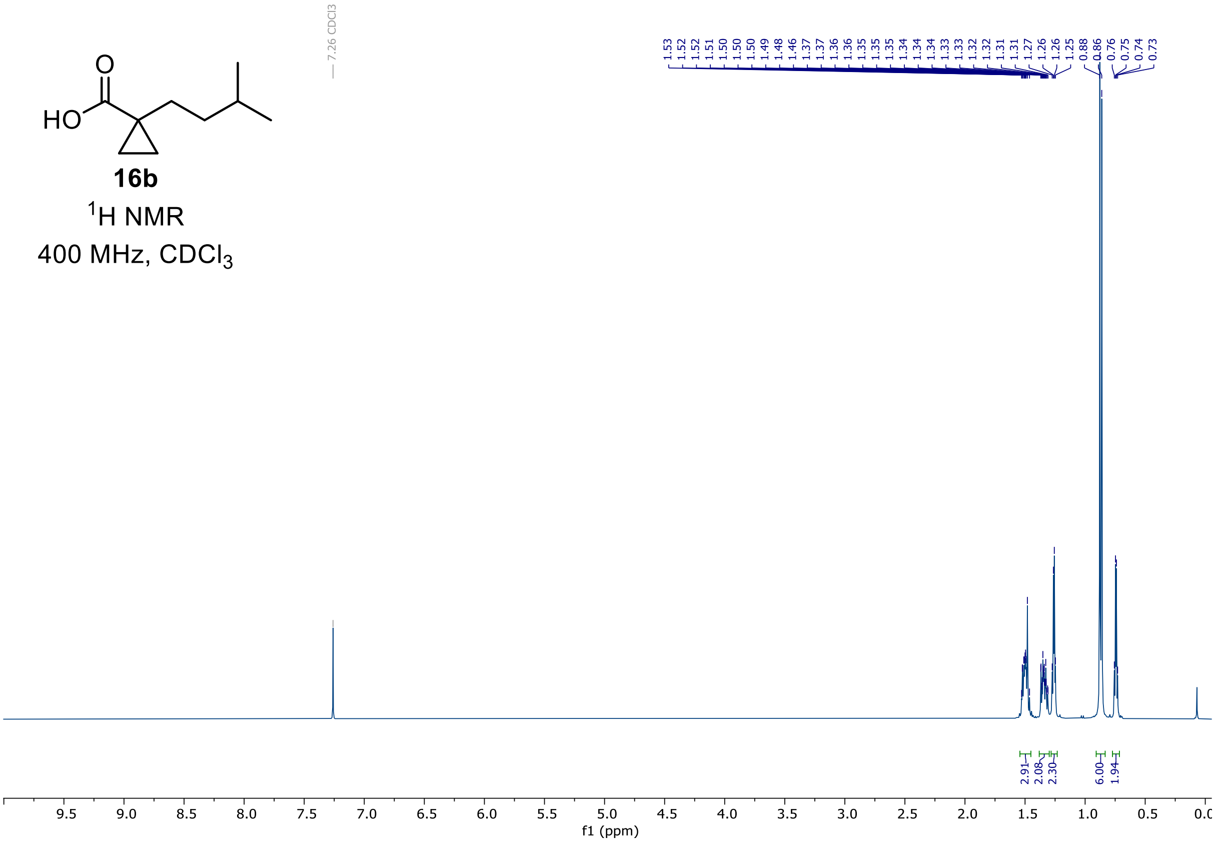

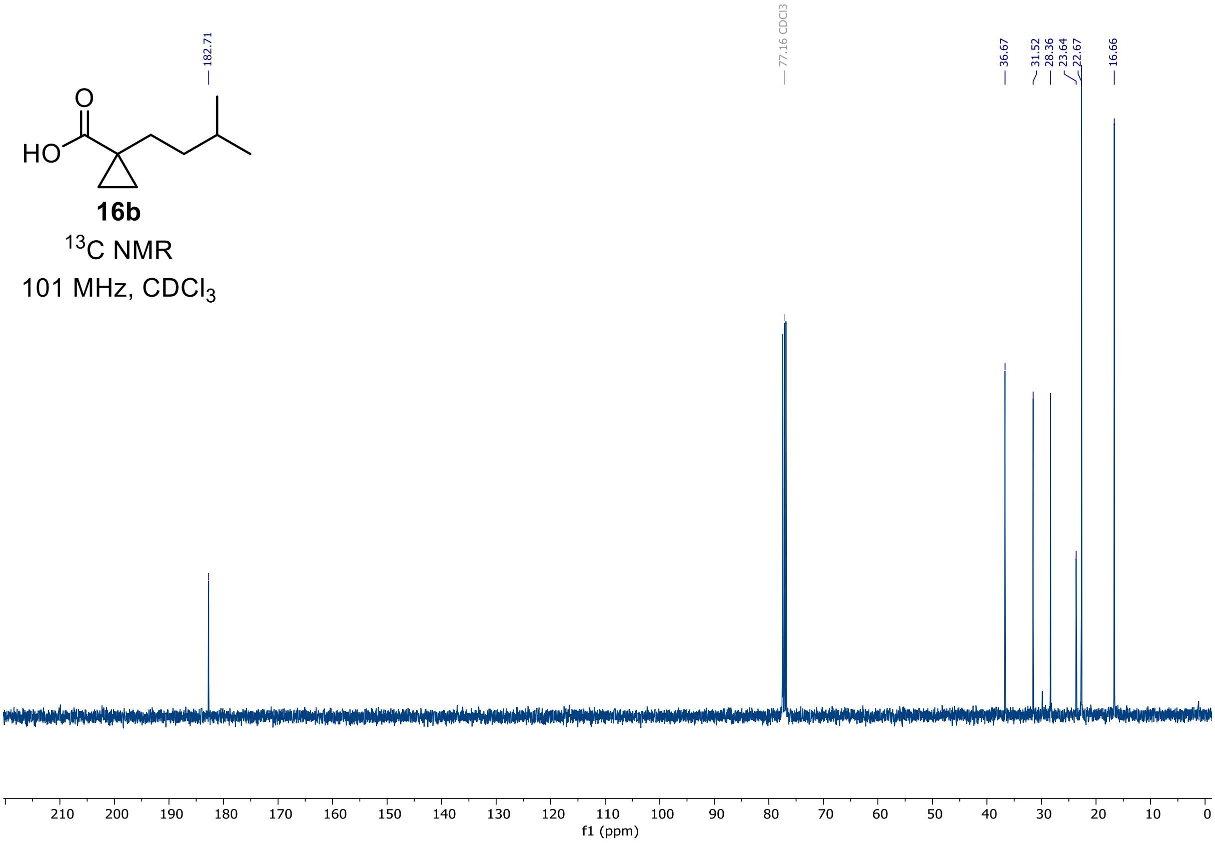

15c (1-(4-(Benzyloxy)butyl)cyclopropyl)(4-hydroxy-2,3,5,6-tetramethylphenyl)methanone

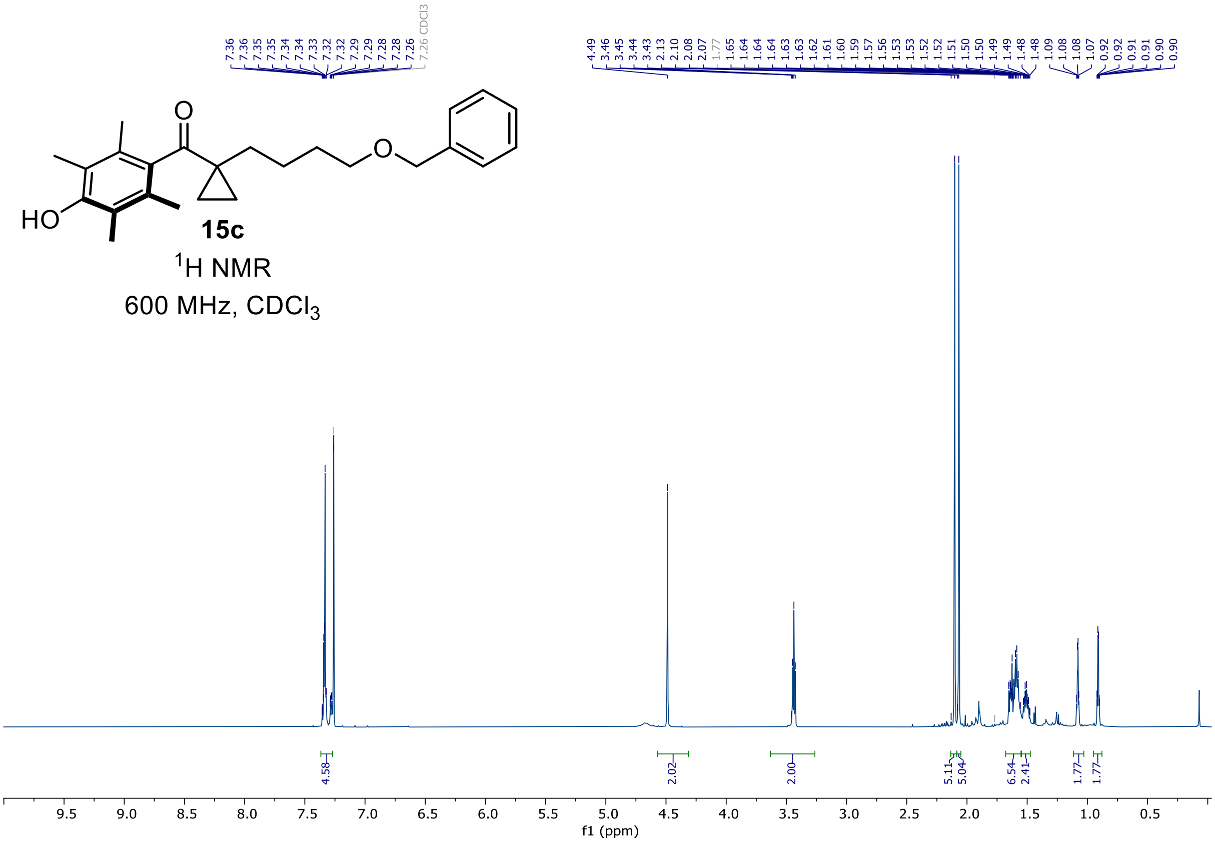

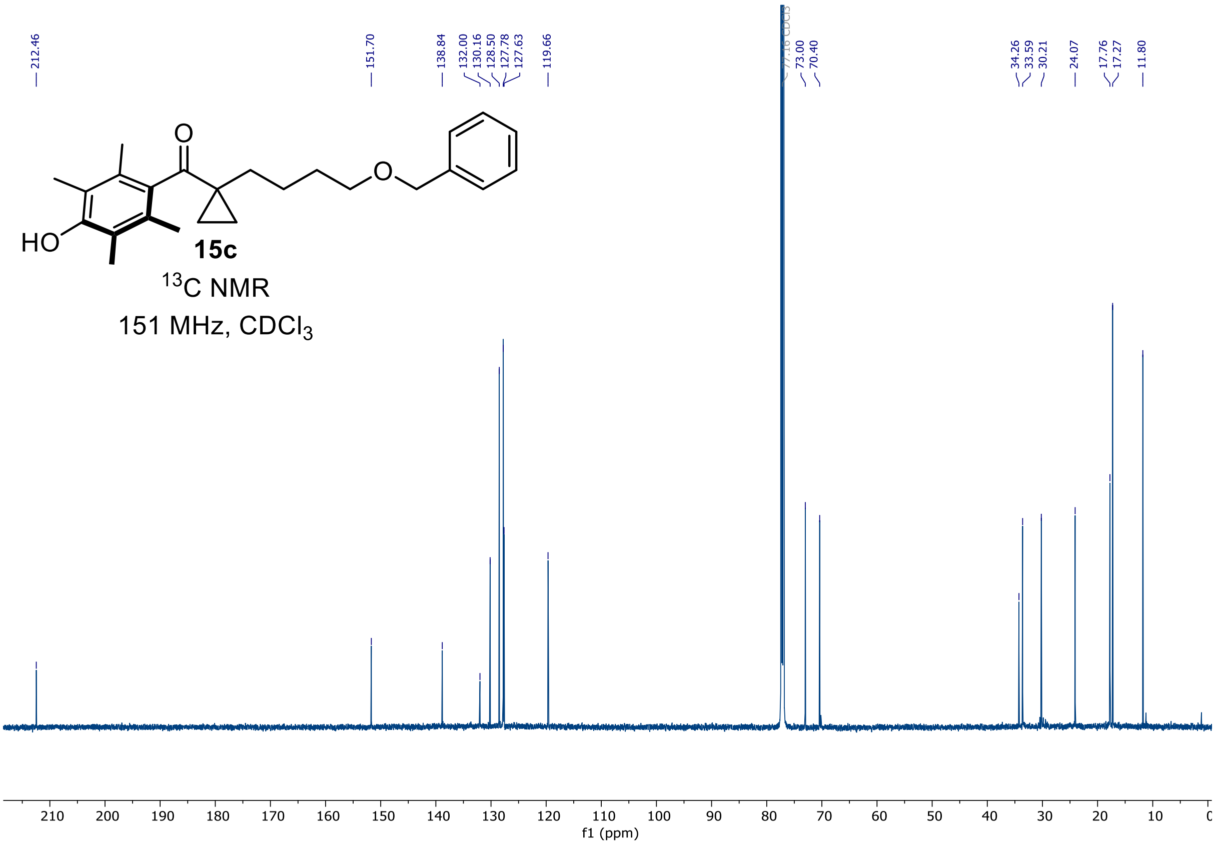

16c 1-(4-(Benzyloxy)butyl)cyclopropane-1-carboxylic acid

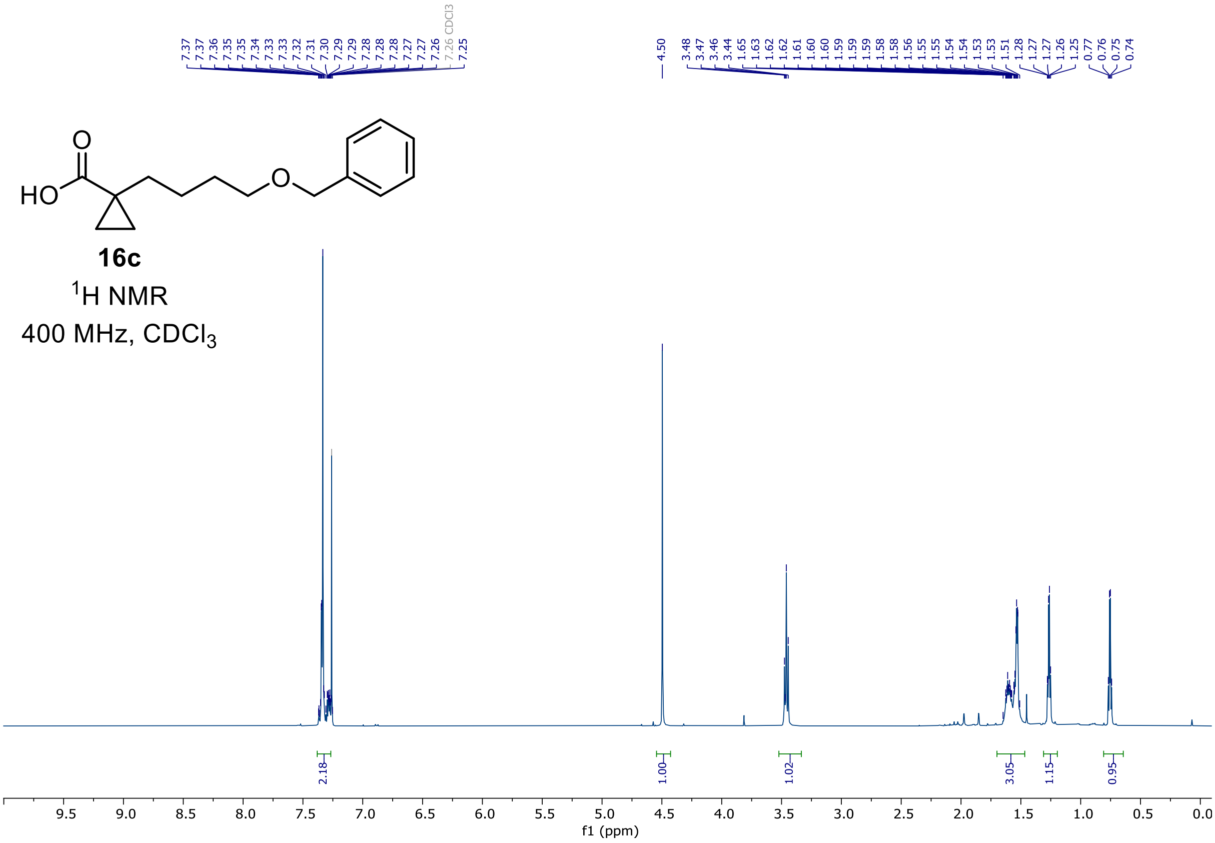

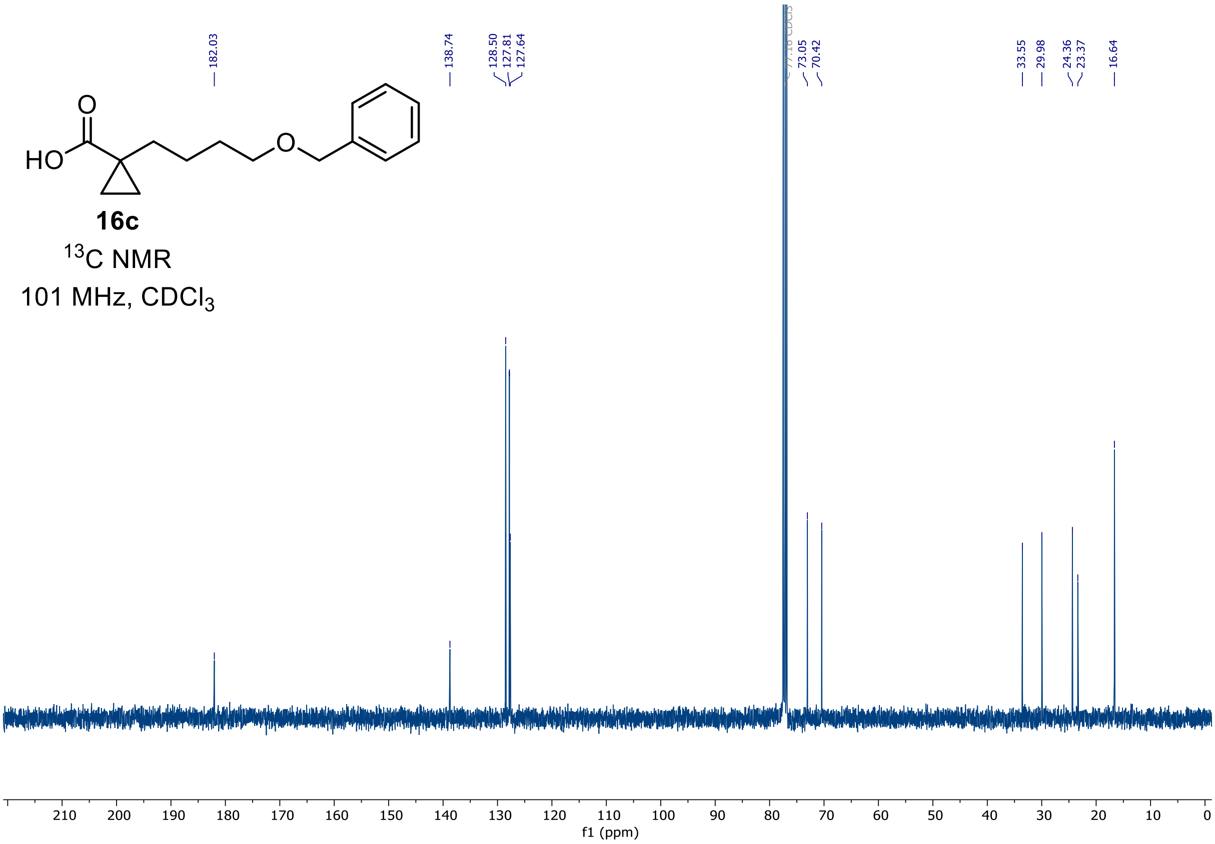

15d 1-(Cyclopropylmethyl)cyclopropyl)(4-hydroxy-2,3,5,6-tetramethylphenyl)methanone

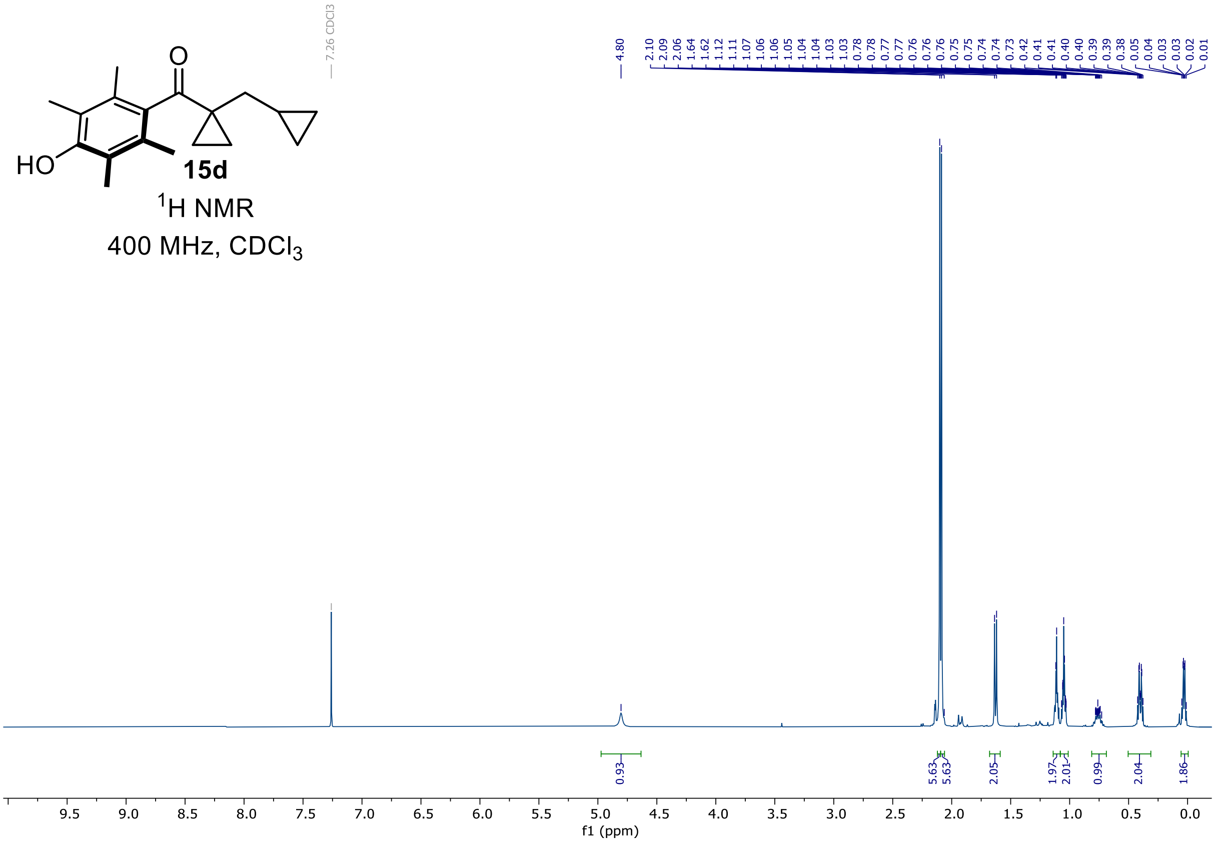

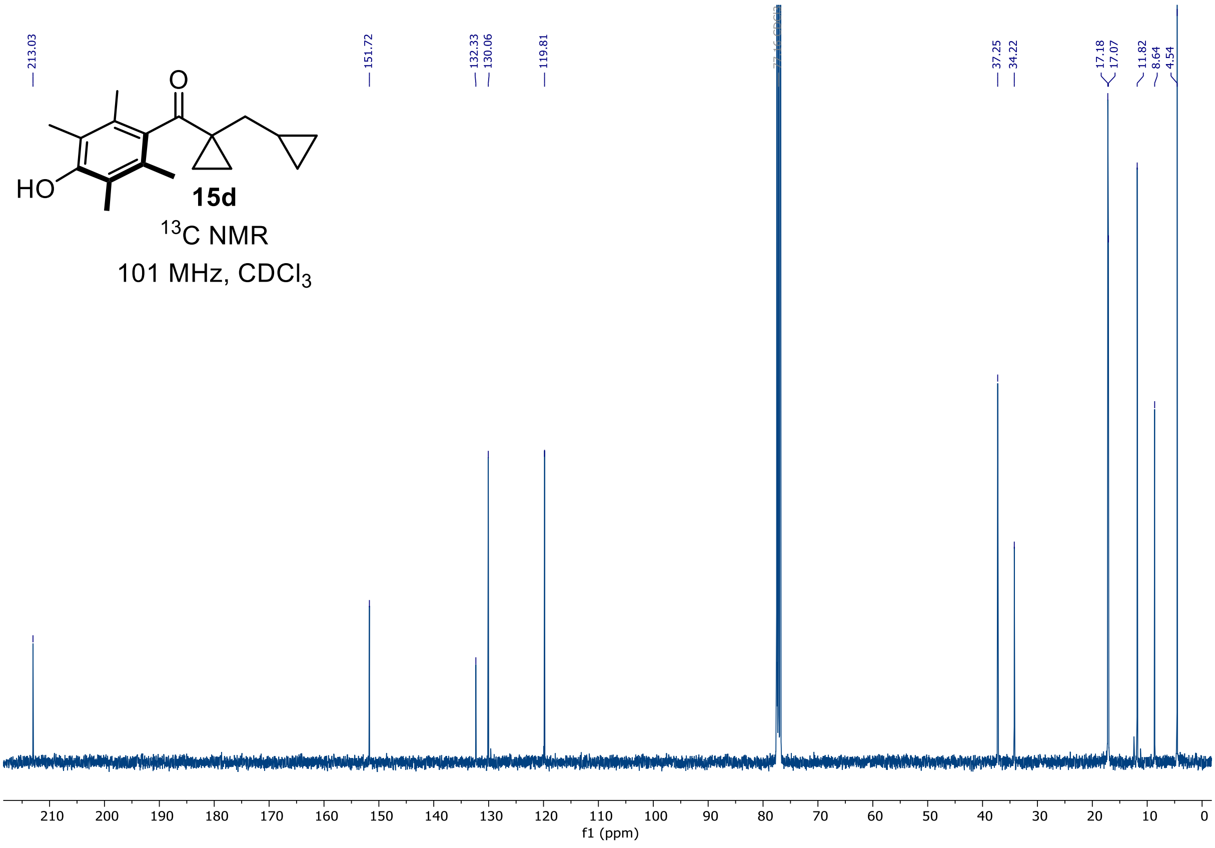

16d 1-(Cyclopropylmethyl)cyclopropane-1-carboxylic acid

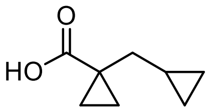

16d
^{1}H NMR
400 MHz, MeOD

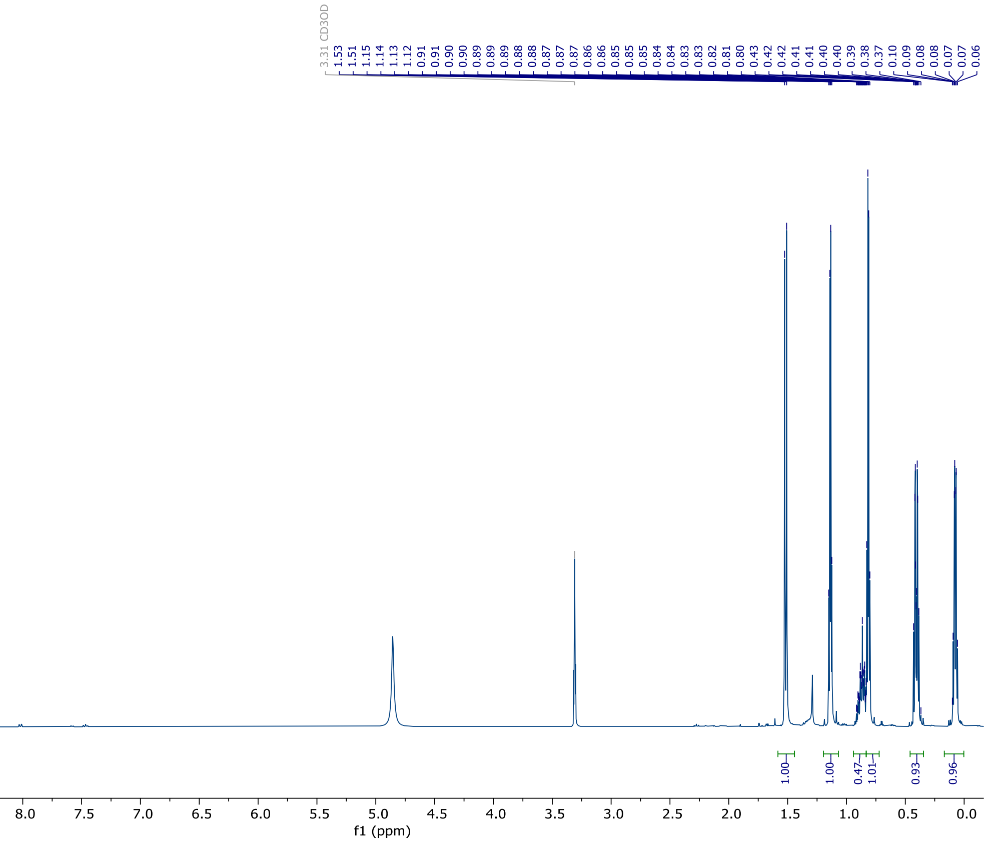

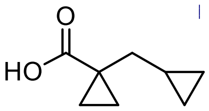

16d
^{13}C NMR
101 MHz, MeOD

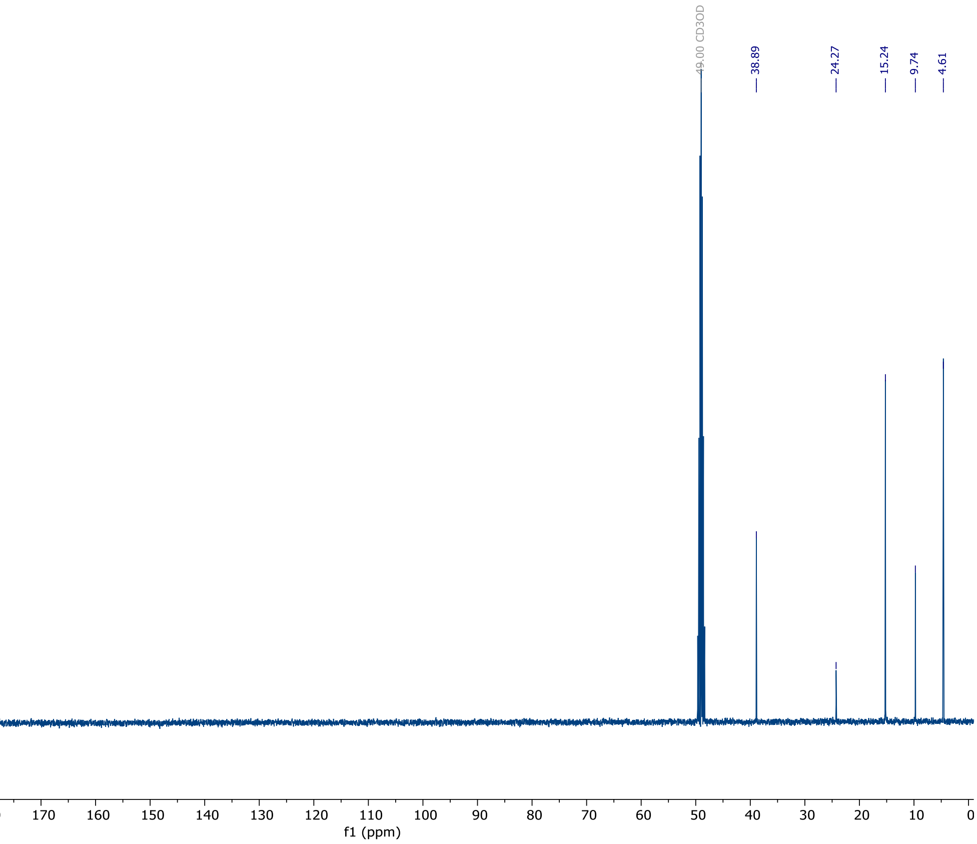

15e (1-Benzylcyclopropyl)(4-hydroxy-2,3,5,6-tetramethylphenyl)methanone

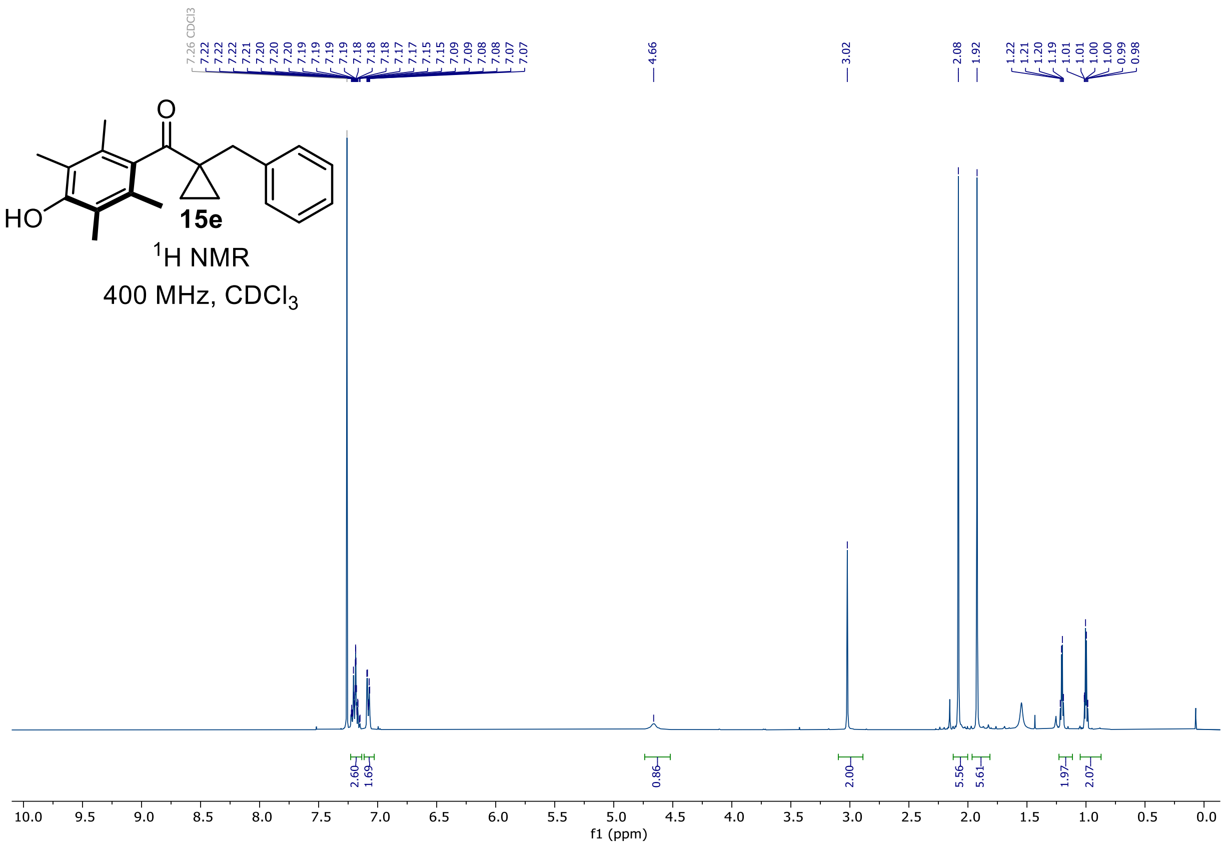

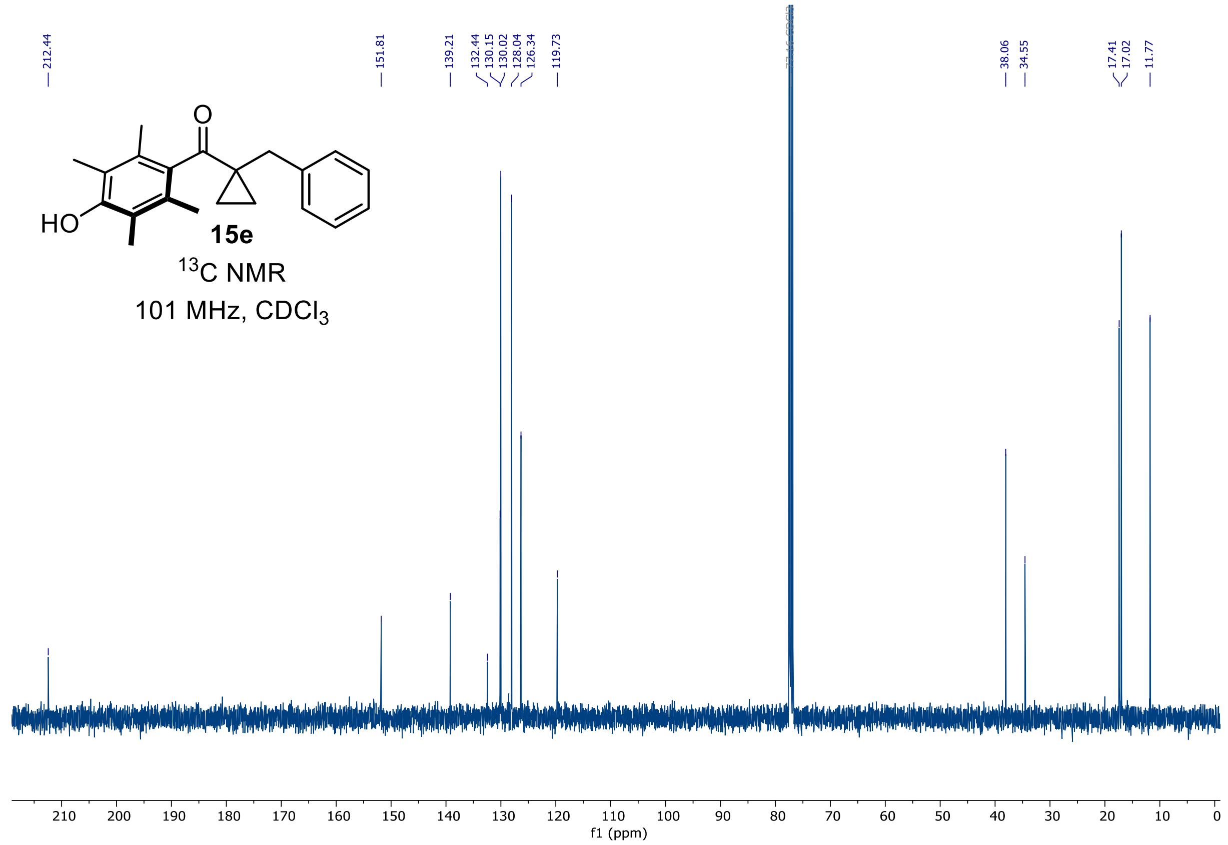

X-Ray Crystallography Supplementary Information

8a (1-Benzylcyclopropyl)(2,3,5,6-tetramethylphenyl)methanone

Crystals were grown over 1-2 weeks *via* vapour diffusion using CH_2Cl_2 as the solvent and ^{i}PrOH as the precipitant.

Single Crystal Data for **8a**: C21H24O, Mr =292.42. 100 K – monoclinic, P 2_1/n, a = 12.6727(4) Å, b = 8.6802(3) Å, c = 14.8889(4) Å, β = 91.672(3)°, V = 1637.11(9) Å^3, Data/restraints/parameters – 4056/0/199, Rint = 0.028, Final R1 = 0.0393, wR2 = 0.0924 (I>2σ(I)).

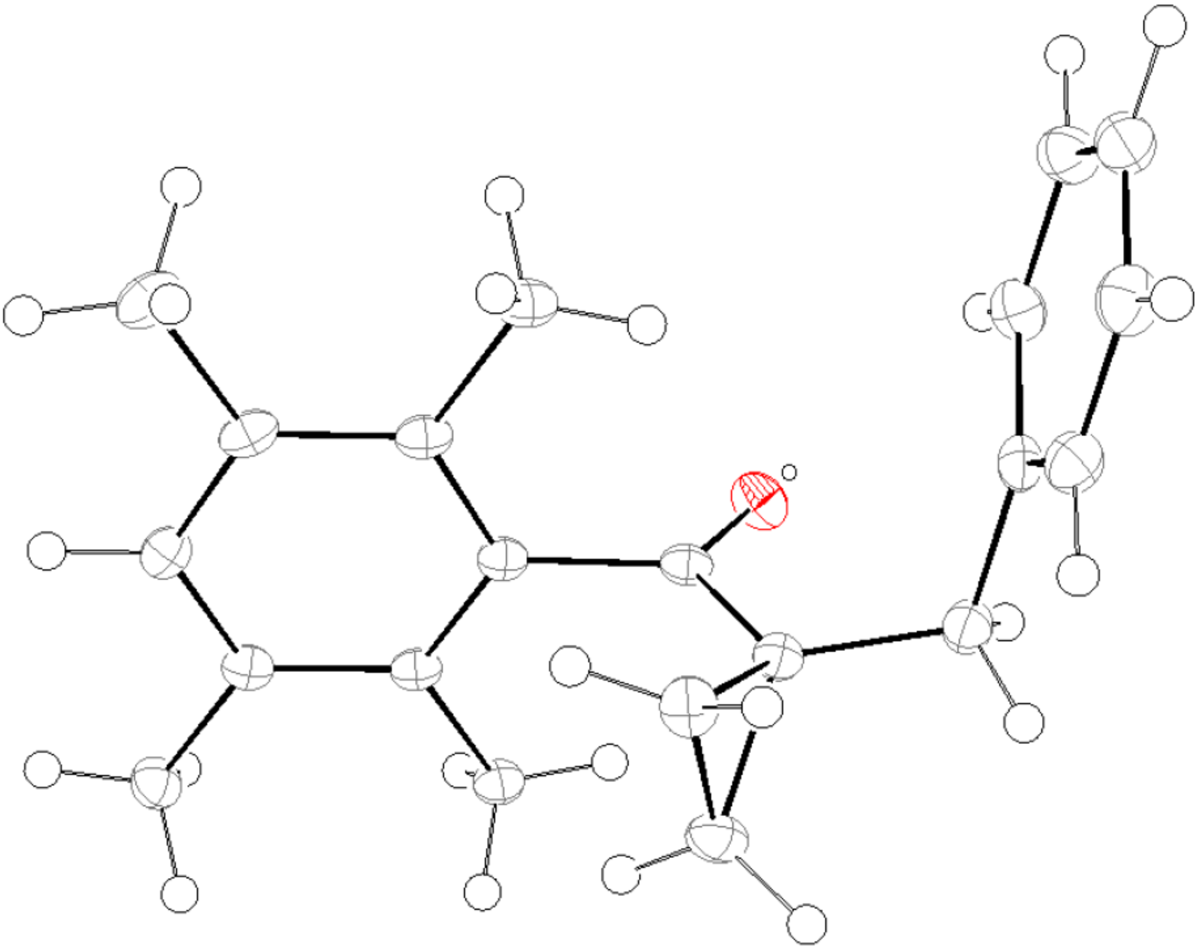

***Figure 1:** Thermal ellipsoid plot for compound **8a**. Thermal ellipsoids are shown at 50% probability.*

8m (1-(2-(Dibenzylamino)ethyl)cyclopropyl)(2,3,5,6-tetramethylphenyl)methanone

Crystals were grown over 1-2 weeks *via* vapour diffusion using a mixture of MTBE and CH_2Cl_2 as the solvent and hexane as the precipitant.

Single Crystal Data for **8m**: C30H35NO, Mr =425.61. 100 K – monoclinic, C 2/c, a = 19.4632(5) Å, b = 6.9774(2) Å, c = 35.9129(8) Å, β = 92.285(2)°, V = 4873.2(2) $Å^3$, Data/restraints/parameters – 6035/584/333, Rint = 0.050, Final R1 = 0.0418, wR2 = 0.0981 (I>2σ(I)).

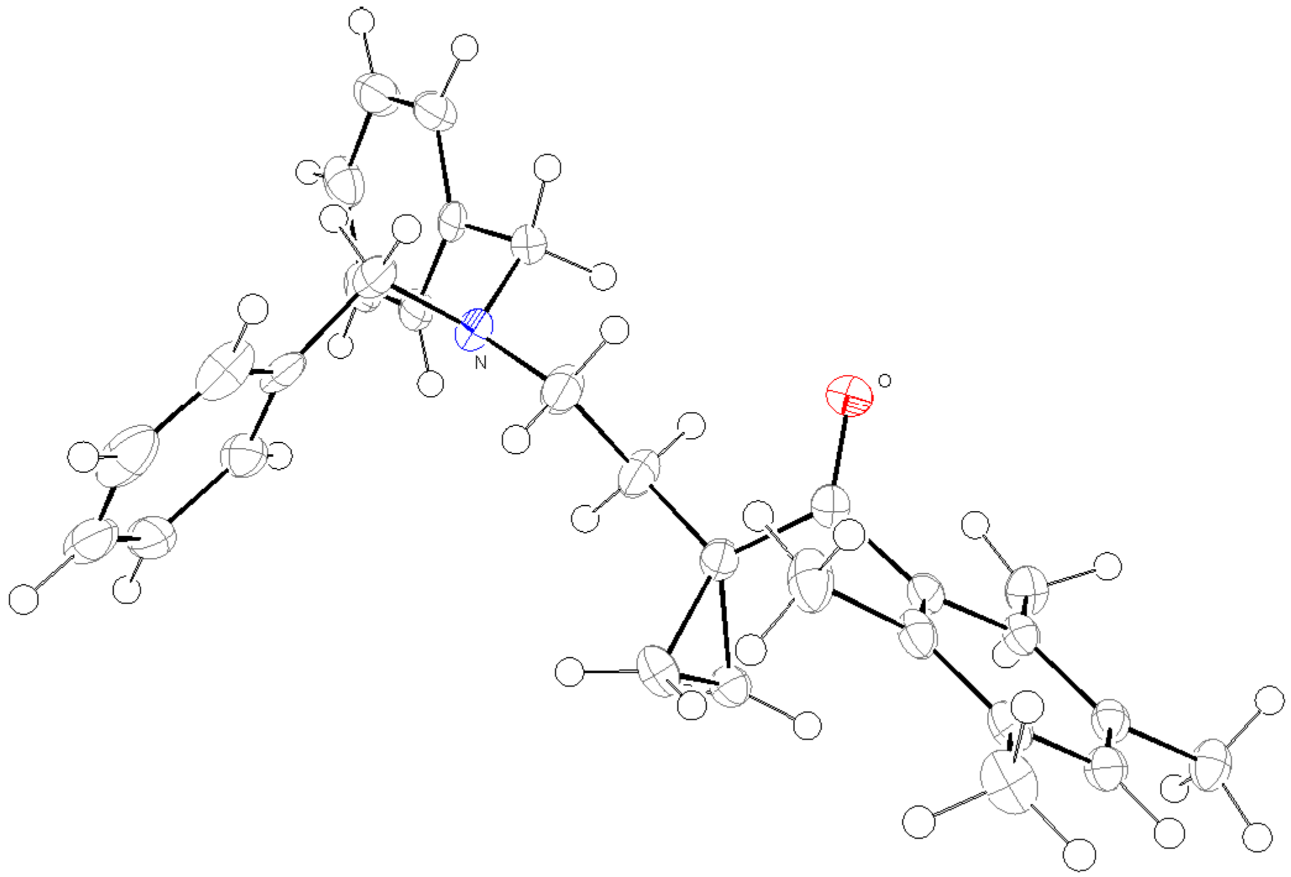

***Figure 2**: Thermal ellipsoid plot for **8m.** Thermal ellipsoids are shown at 50% probability. Disorder is omitted for clarity.*

References

1. Pangborn, A. B.; Giardello, M. A.; Grubbs, R. H.; Rosen, R. K.; Timmers, F. J., *Organometallics* **1996,** *15* (5), 1518-1520.
2. Palatinus, L.; Chapuis, G., *J. Appl. Cryst.* **2007,** *40*, 786-790.
3. Parois, P.; Cooper, R. I.; Thompson, A. L., *Chem. Cent. J.* **2015,** *9* (1), 30.
4. Cooper, R. I. T., A. L.; Watkin, D. J., *J. Appl. Cryst.* **2010,** *43*, 1100-1107.
5. Frost, J. R.; Cheong, C. B.; Akhtar, W. M.; Caputo, D. F.; Stevenson, N. G.; Donohoe, T. J., *J. Am. Chem. Soc.* **2015,** *137* (50), 15664-7.
6. Frost, J. R.; Cheong, C. B.; Akhtar, W. M.; Caputo, D. F.; Stevenson, N. G.; Donohoe, T. J., *J Am Chem Soc* **2015,** *137* (50), 15664-7.
7. Hong, F.-T.; Paquette, L. A., *J Org Chem* **1999,** *64* (10), 3783-3786.
8. Gan, S.; Yin, J.; Yu, Z.; Song, L.; Shi, L., *Green Chemistry* **2022,** *24* (5), 2232-2239.
9. Cheong, C. B.; Frost, J. R.; Donohoe, T. J., *Synlett* **2020,** *31* (18), 1828-1832.
10. Liu, Y.; Park, S. K.; Xiao, Y.; Chae, J., *Organic & Biomolecular Chemistry* **2014,** *12* (26), 4747-4753.
11. Yuan, C.; Eliasen, A. M.; Camelio, A. M.; Siegel, D., *Nature Protocols* **2014,** *9* (11), 2624-2629.
12. Shen, P.-X.; Hu, L.; Shao, Q.; Hong, K.; Yu, J.-Q., *J Am Chem Soc* **2018,** *140* (21), 6545-6549
13. Jahngen, E. G. E.; Phillips, D.; Kobelski, R. J.; Demko, D. M., *The Journal of Organic Chemistry* **1983,** *48* (15), 2472-2476.
